# Structural variation of the complete chloroplast genome and plastid phylogenomics of the genus *Asteropyrum* (Ranunculaceae)

**DOI:** 10.1038/s41598-019-51601-2

**Published:** 2019-10-25

**Authors:** Jian He, Min Yao, Ru-Dan Lyu, Le-Le Lin, Hui-Jie Liu, Lin-Ying Pei, Shuang-Xi Yan, Lei Xie, Jin Cheng

**Affiliations:** 1 Beijing Forestry University, Beijing, 100083 China; 2 Beijing Forestry University Forest Science Co. Ltd., Beijing, 100083 China; 3 Henan Agricultural University, Zhengzhou, 450002 China; 4 Institute of Botany, the Chinese Academy of Sciences, Beijing, 100093 China

**Keywords:** Phylogenetics, Plant evolution

## Abstract

Two complete chloroplast genome sequences of *Asteropyrum*, as well as those of 25 other species from Ranunculaceae, were assembled using both Illumina and Sanger sequencing methods to address the structural variation of the cp genome and the controversial systematic position of the genus. Synteny and plastome structure were compared across the family. The cp genomes of the only two subspecies of *Asteropyrum* were found to be differentiated with marked sequence variation and different inverted repeat-single copy (IR-SC) borders. The plastomes of both subspecies contains 112 genes. However, the IR region of subspecies *peltatum* carries 27 genes, whereas that of subspecies *cavaleriei* has only 25 genes. Gene inversions, transpositions, and IR expansion-contraction were very commonly detected in Ranunculaceae. The plastome of *Asteropyrum* has the longest IR regions in the family, but has no gene inversions or transpositions. Non-coding regions of the cp genome were not ideal markers for inferring the generic relationships of the family, but they may be applied to interpret species relationship within the genus. Plastid phylogenomic analysis using complete cp genome with Bayesian method and partitioned modeling obtained a fully resolved phylogenetic framework for Ranunculaceae. *Asteropyrum* was detected to be sister to *Caltha*, and diverged early from subfamily Ranunculoideae.

## Introduction

In recent years, the use of whole chloroplast (cp) genome data for plant phylogenetic reconstruction has been greatly improved our understanding of evolutionary relationships of angiosperms at a wide range of taxonomic levels^1–4^. Chloroplast genome shows uniparental inheritance in most angiosperm species and has a size ranging from 115 to 165 kb^5^. It usually has a conserved circular structure containing a large single copy (LSC) and a small single copy (SSC), which are separated by two copies of inverted repeat (IR) regions. Rates of nucleotide substitution in the cp genome are relatively slow and therefore can provide resolution of plant phylogeny at generic and familial levels^6–13^. The gene number and arrangement of the cp genome are often well conserved in angiosperms^14^. However, variations in the structure of the cp genome, including inversions, transpositions of certain regions, and expansion of IRs, are not uncommon in many families, including Ranunculaceae, Fabaceae, and Asteraceae^11,12,15–20^. Recent studies of the cp genomes of Ranunculales showed that inversions, transpositions, and IR expansions may provide strong phylogenetic information, and plastid phylogenomic analysis can yield infra-familial phylogeny with high resolution^3,11,12,15,20^.

**Figure 1:**
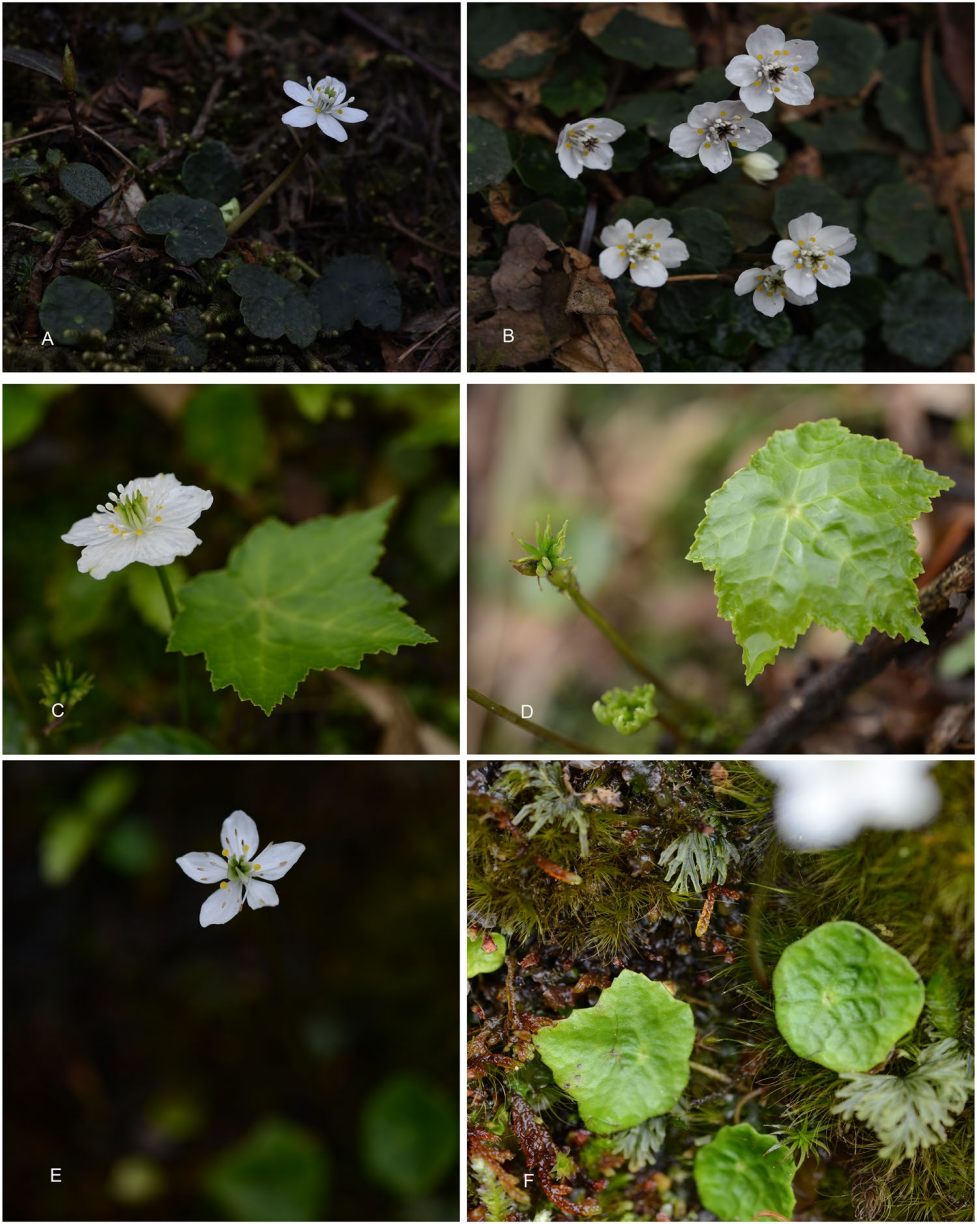
Field photographs of *Asteropyrum* taxa. (**A**,**B**) *Asteropyrum peltatum* ssp. *peltatum* (photos taken by L. Xie from Emei Mountain, Sichuan, China). (**C**,**D**) *Asteropyrum peltatum* ssp. *cavaleriei* (photos taken by L. Xie from Eryanping, Yibin, Sichuan, China). (**E**,**F**) An intermedium form of the two subspecies (photos taken by L. Xie from Laojunshan, Yibin, Sichuan, China).

*Asteropyrum* Drumm. et Hutch. is a small but very distinctive genus in Ranunculaceae distributed predominantly in China. It is famous for its great pharmaceutical value and can be used as substitutes of goldthread (*Coptis* Salisb.) for curing icterus, hydroncus, and diarrhea in rural areas of southern China^21^. The plants of *Asteropyrum* are small perennial herbs with simple peltate leaves, white sepals and small golden yellow petals (Fig. 1). Traditionally, the genus was considered to have two species, *A. peltatum* (Franch.) Drumm. et Hutch. and *A. cavaleriei* (Lévl. et Vant.) Drumm. et Hutch. They have different sizes and leaf shapes^21–23^. *Asteropyrum peltatum* is often smaller (usually less than 10 cm tall) than *A. cavaleriei* with suborbicular to inconspicuously 5-sided peltate leaves (Fig. 1A,B). Whereas, scape of *A. cavaleriei* is often 12–20 cm tall and the leaf is also much larger with five-angled blade (Fig. 1C,D). However, intermediate forms are common in the overlap zones of these two taxa (Fig. 1E,F). Thus, *A. cavaleriei* has often been treated as a subspecies of *A. peltatum*^24^.

The systematic position of *Asteropyrum* within Ranunculaceae has long been an interesting issue and disputed for almost a century. Drummond & Hutchinson^25^ separated this genus from *Isopyrum* L. and reported that *Asteropyrum* may be closely related to *Caltha* L. and *Trollius* L. However, later authors argued that this genus may be closely related to *Isopyrum*^26^, *Copis*^21,27^, or *Calathodes* Hook. f. & Thoms.^23^, and its chromosome type was also interpreted differently^24,28–31^.

Recent molecular phylogenetic studies also did not clearly resolve the position of *Asteropyrum*. It was found to be closely related to the (*Beesia*, (*Eranthis*, *Cimicifuga*)) clade by a study using *rbc*L and 26s rDNA^32^, or sister to *Caltha* by a study using three plastid regions, one nuclear region, and morphological data^33^, or sister to tribe Cimicifugeae by a study using five plastid and one nuclear regions^34^, and sister to *Callianthemum* C. A. Mey. in a study using four plastid, three nuclear, and one mitochondrial regions^35^. However, these studies did not obtain a robust phylogenetic framework of Ranunculaceae, and the systematic position of *Asteropyrum* in the family still remains to be clarified.

Plastid phylogenomic studies may be particularly suitable for resolving the generic relationship within Ranunculaceae, and structural variations of the cp genome, e.g., gene inversion, gene transposition, and IR expansion-contraction, may provide important systematic information about the family^3,11,12,15,20^. Zhai *et al*. carried out a plastid phylogenomic study focusing on Ranunculaceae based on a well sampling scheme^15^. They also discussed structural variation of plastid genome sequences of the family. However, their study focused on tribal relationship of Ranunculaceae and only included one sample of *Asteropyrum*. In their study, they detected and reported five types of plastome sequences of the family based on gene inversions and transpositions but left IR expansion-contraction (widely distributed and highly diverged in the family) issue unaddressed. For phylogenomic analyses, they used two matrices (coding region and complete plastome sequences) and three phylogenetic reconstruction methods (Parsimony, Maximum Likelihood, and Bayes) with no partitioned modeling applied the Bayesian analysis. The results gave a better resolution of the family. However, position of *Asteropyrum* was still not well supported.

In this study, we reported the complete cp genome sequences of both subspecies of *Asteropyrum*, as well as 25 plastome sequences from other genera in Ranunculaceae using genome skimming data. We then compared the synteny and plastome structure across the family and conducted a plastid phylogenomic study using partitioned modeling method to resolve deep-level relationships and explore plastome structural evolution across Ranunculaceae. The aims of these analyses were to clarify variation in the cp genome across the genus *Asteropyrum*, to detect structural variation in the *Asteropyrum* cp genome in comparison with other genera in the family, to infer the phylogenetic position of *Asteropyrum* within the family, and to try to compare and reconstruct the deep-level relationships of Ranunculaceae using different plastome partitions.

## Results

### Plastome organization and features of *Asteropyrum* and its relatives

We obtained 3.0 Gb and 3.2 Gb Next-Generation Sequencing (NGS) clean datasets for *Asteropyrum peltatum* ssp. *peltatum* (Franch.) Drumm. & Hutch. (16,552,494 reads) and *A. peltatum* ssp. *cavaleriei* (Lévl. & Vant.) Q. Yuan & Q. E. Yang (17,655,994 reads), respectively. Blat analysis was used to select 133,486 putative plastid reads for ssp. *peltatum* and 144,722 reads for ssp. *cavaleriei*. The reads from ssp. *peltatum* were used to obtain two large contigs from the *de novo* assembly (51,204 bp, and 81,421 bp). One gap was bridged using Sanger sequencing (with primers: LSCF: TGCGATGCTCTAACCTCTGAG; LSCR: AGAGCAATCCTAACCAGAATCATCT). For the ssp. *cavaleriei* data, only one large contig (131,836 bp), which included complete LSC, IR, and SSC regions was derived. Information regarding the cp genome assembly for all of the other newly sequenced samples from Ranunculaceae is presented in Supplementary Table S1.

**Figure 2:**
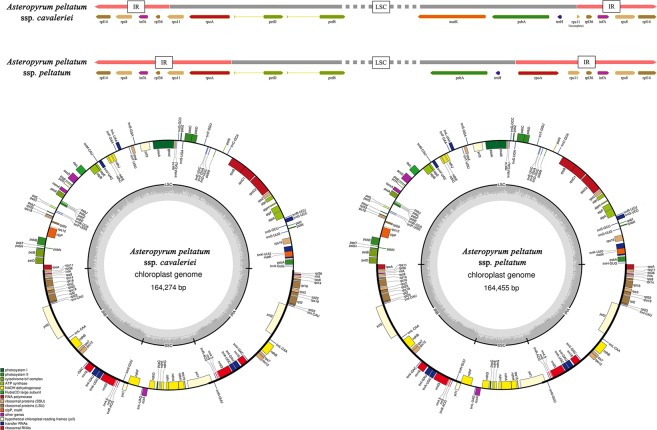
The complete plastid genomes of the two subspecies of *Asteropyrum*. Upper: Schematic representation of the two plastome sequences of *Asteropyrum* showing the different IR-SC boundaries of the two subspecies; Lower: Chloroplast genome maps of the two subspecies. The thick lines on the outer complete circle identify the inverted repeat regions (IRa and IRb). The innermost track of the plastome shows the G + C content. Genes on the outside of the map are transcribed in a clockwise direction, whereas genes on the inside of the map are transcribed in a counter clockwise direction.

The complete cp genome sequences of ssp. *peltatum* and ssp. *cavaleriei* are 164,455 bp and 164,274 bp, respectively (Fig. 2, Table 1), and the rate of identical sites between the two plastome sequences was 98.1%. The two complete cp genome sequences had similar GC content (38.0% for ssp. *cavaleriei* and 37.9% for ssp. *peltatum*). Within the circular plastid genome of *Asteropyrum*, the IR region had the richest GC content, followed by the LSC region, while the SSC region had the lowest GC content (Table 1).

**Table 1:** Summary of the chloroplast genomes of *Asteropyrum*.

Category	*Asteropyrum peltatum* ssp. *peltatum*	*Asteropyrum peltatum* ssp. *cavaleriei*
Total cp genome size (bp)	164455	164274
Length of large single copy region (bp)	81819	84284
Length of inverted repeat region (bp)	32659	31429
Length of small single copy region (bp)	17318	17132
Coding size (bp)	106585	105105
Intron size (bp)	15665	15730
Spacer size (bp)	42205	43439
Total GC content (%)	38.0	37.9
GC content of LSC (%)	36.1	36.0
GC content of IR (%)	41.7	42.1
GC content of SSC (%)	32.5	32.6
Total number of genes	112	112
Number of genes in LSC	73	74
Number of genes in SSC	12	12
Number of genes duplicated in IR	27	25
Number of protein encoding genes	78 (*rpl32* absent*)	78 (*rpl32* absent*)
Number of tRNA genes	30	30
Number of rRNA genes	4	4

*Comparing to *Amborella*.

The LSC region of ssp. *cavaleriei* was found to be longer than that of ssp. *peltatum* (84,284 bp vs. 81,819 bp), whereas the IR region of ssp. *cavaleriei* was significantly shorter than that of ssp. *peltatum* (31,429 bp vs. 32,659 bp), because the LSC region of ssp. *cavaleriei* carried two more genes (*rpoA* and *rps11*) at the border of the LSC and IR than that of ssp. *peltatum*. At the same time, the gene number of the IR region of ssp. *cavaleriei* was two less than that of ssp. *peltatum* (Fig. 2).

Because the gene numbers in the IR regions of the two subspecies differed, we designed two pairs of primers to assess the stability of the IR-SC boundary of both subspecies using additional samples. We also tested the IR-SC boundary of an intermediate form as well. The results showed that the IR-SC boundaries within each subspecies are stable. The intermediate individual collected from Yibin, Sichuan had an IR-SC boundary that was identical with that of ssp. *cavaleriei*. The features of the other 25 newly sequenced cp genomes and their gene maps are presented in Supplementary Table S2 and Supplementary Fig. S1.

### Structural variation of the chloroplast genome in Ranunculaceae

**Figure 3:**
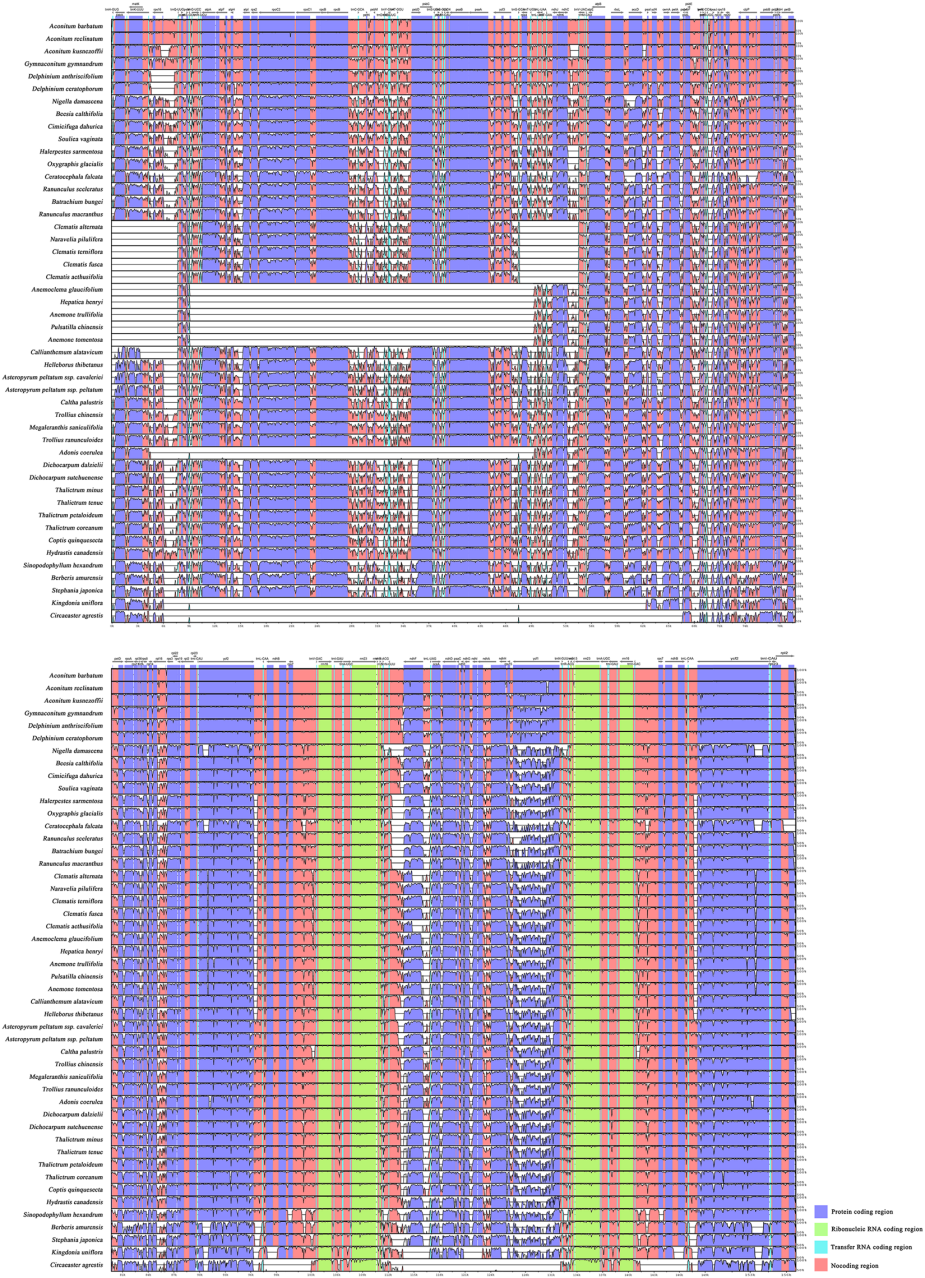
Sequence alignment of the complete plastome sequences of Ranunculaceae and outgroup samples compared in this study using the mVISTA program and LAGAN method. A cut-off of 70% similarity was used for the plot, and the Y-scale represents the percent similarity ranging from 50–100%. Blue represents coding regions, and pink represents non-coding regions.

When the LAGAN alignment program was applied, the mVISTA results showed a large proportion of un-matched area in tribe Anemoneae and the genus *Adonis* (Fig. 3), which indicated inversions and gene transpositions in the cp genomes of these plants. The shuffle-LAGAN method was used to obtain a well-matched global alignment for all plastome sequences of Ranunculales (Supplementary Fig. S2). Inversions and transpositions in cp genomes of tribe Anemoneae have been reported previously^11,12,15,20^, and the cp genome of *Adonis* was found to have a large inversion in the LSC region between *rps*16 and *trn*T-UGU, which contains 35 genes (43,411 bp in total length), including 21 protein-coding genes and 14 tRNA genes (Supplementary Fig. S1-*Adonis*). The inversion within *Adonis* showed no phylogenetic relationship with that in tribe Anemoneae.

IR expansion-contraction was found to be very common in Ranunculaceae and its relatives (Table 2, Supplementary Fig. S3). After comparing a wide range of angiosperm cp genomes, we identified the cp genome of *Amborella* as the standard IR composition (with 17 genes) that is commonly shared by most of angiosperms. In Ranunculaceae, the cp genomes of *Asteropyrum*, *Dichocarpum* W. T. Wang & P. K. Hsiao, *Hydrastis* L., and tribe Anemoneae (*Anemone* L., *Pulsatilla* Mill., *Hepatica* Mill., *Anemoclema* (Franch.) W. T. Wang, *Clematis* L., *Naravelia* DC., etc.) showed IR expansion, whereas IR contraction was detected in *Helleborus* L. and *Ceratocephala* Moen. (detailed IR information for all tested Ranunculaceae samples is presented in Supplementary Table S3). The longest IR in Ranunculales was found to be that of *Berberis amurensis* Rupr., which carries 32 genes. *Asteropyrum* showed the longest IR regions within the family Ranunculaceae (Fig. 2, Table 2).

**Table 2:** Information regarding IR expansion-contraction for all analyzed Ranunculales species using the *Amborella* plastome as the standard.

Species	Gene number in IR	length of IR	Additional (or absent) genes when IR expansion-contraction occurs (*Amborella* as the standard)
*Amborella trichopoda*	17	26651	
*Aconitum barbatum*	17	26090	
*Aconitum kuznezoffii*	17	26282	
*Aconitum reclinatum*	17	26061	
*Adonis coerulea*	17	26087	
*Anemoclema glaucifolium*	24	31256	*infA, rps8, rpl14, rpl16, rps3, rpl22, rps19*
*Anemone tomentosa*	24	31490	*infA, rps8, rpl14, rpl16, rps3, rpl22, rps19*
*Anemone trullifolia*	24	31022	*infA, rps8, rpl14, rpl16, rps3, rpl22, rps19*
*Asteropyrum peltatum spp. cavaleriei*	25	31429	*rpl36, infA, rps8, rpl14, rpl16, rps3, rpl22, rps19*
*Asteropyrum peltatum spp. peltatum*	27	32659	*rpoA, rps11, rpl36, infA, rps8, rpl14, rpl16, rps3, rpl22, rps19*
*Batrachium bungei*	17	25352	
*Beesia calthifolia*	17	26500	
*Berberis amurensis*	32	37152	*psbB, psbT, psbN, psbH, petB, petD, rps11, rpl36, infA, rps8, rpl14, rpl16, rps3, rpl22, rps19*
*Callianthemum alatavicum*	17	25978	
*Caltha palustris*	17	26421	
*Ceratocephala falcata*	16	24165	*rpl2* (absent)
*Cimicifuga dahurica*	17	26572	
*Circaeaster agrestis*	21	28023	*rps19, trnQ-UUG, trnL-UAG, rpl32*
*Clematis aethusifolia*	23	31041	*rps8, rpl14, rpl16, rps3, rpl22, rps19*
*Archiclematis alternata*	23	31037	*rps8, rpl14, rpl16, rps3, rpl22, rps19*
*Clematis fusca*	24	31039	*infA, rps8, rpl14, rpl16, rps3, rpl22, rps19*
*Clematis terniflora*	24	31045	*infA, rps8, rpl14, rpl16, rps3, rpl22, rps19*
*Coptis quinquesecta*	17	26442	
*Delphinium anthriscifolium*	17	25977	
*Delphinium ceratophorum*	17	26560	
*Dichocarpum dalzielii*	17	26535	
*Dichocarpum sutchuenense*	19	27622	*rpl22, rps19*
*Gymnaconitum gymnandrum*	17	26140	
*Halerpestes sarmentosa*	17	25057	
*Helleborus thibetanus*	16	24999	*rpl2* (absent)
*Hepatica henryi*	24	31039	*infA, rps8, rpl14, rpl16, rps3, rpl22, rps19*
*Hydrastis canadensis*	18	27032	*rps19*
*Kingdonia uniflora*	19	31109	*ndhB* (loss); *rps19, ycf1, rps15*
*Megaleranthis saniculifolia*	17	26608	
*Naravelia pilulifera*	23	31054	*rps8, rpl14, rpl16, rps3, rpl22, rps19*
*Nigella_damascena*	17	25167	
*Oxygraphis glacialis*	17	25094	
*Pulsatilla chinensis*	24	31115	*infA, rps8, rpl14, rpl16, rps3, rpl22, rps19*
*Ranunculus macranthus*	17	25791	
*Ranunculus sceleratus*	17	25302	
*Sinopodophyllum hexandrum*	17	25950	
*Souliea vaginata*	17	26533	
*Stephania japonica*	17	24340	
*Thalictrum coreanum*	17	26403	
*Thalictrum minus*	17	26482	
*Thalictrum petaloideum*	17	26480	
*Thalictrum tenue*	17	26504	
*Trollius chinensis*	17	26627	
*Trollius ranunculoides*	17	26500	

### Plastid phylogenomic analyses

Seven datasets and three methods were used to obtain 21 phylogenetic frameworks for the family Ranunculaceae. All of these frameworks were largely similar to each other (presented in Supplementary Fig. S4). Topology conflicts among different datasets or methods were usually not well supported statistically.

The parameters of the parsimony analyses are presented in Table 3. This analysis yielded less resolved phylogenies in comparison with the ML and Bayesian methods. The phylogenetic inferences using the parsimony method showed largely congruent results among the seven datasets. The complete cp genome dataset obtained the best resolved and supported phylogenetic tree, in which *Asteropyrum* was sister to *Nigella* with weak bootstrap support (BS = 62, Supplementary Fig. S4). This result was different from ML and Bayesian analyses (*Asteropyrum* sister to *Caltha*).

**Table 3:** Characteristics of the seven datasets used for parsimony analysis.

Dataset^a^	No. of taxa	Aligned length^b^	Variable sites (percentage of aligned length)	Informative sites (percentage of aligned length)	No. of MP trees	Tree length	Consistency index	Retention index
Large single copy	48	77968 bp	36926 bp (47.36%)	24041 bp (30.83%)	1	93269	0.5850	0.6891
Small single copy	48	18551 bp	11030 bp (60.93%)	7645 bp (41.21%)	2	32922	0.5409	0.6496
Inverted repeats	48	24318 bp	5713 bp (23.49%)	2066 bp (8.50%)	3	8245	0.8018	0.7851
Coding regions	48	74772 bp	24741 bp (33.09%)	15204 bp (20.33%)	2	58454	0.5803	0.6834
Intergenic spacers	48	31036 bp	21476 bp (69.20%)	14081 bp (45.37%)	1	58731	0.5841	0.6781
Introns	48	14373 bp	6755 bp (47.00%)	3979 bp (27.68%)	1	15648	0.6182	0.7007
Complete cp genome	48	120181 bp	52972 bp (44.08%)	33264 bp (27.68%)	1	120181	0.5860	0.6825

^a^Only one IR region was used for analyses.

^b^With removal of ambiguous alignments.

**Figure 4:**
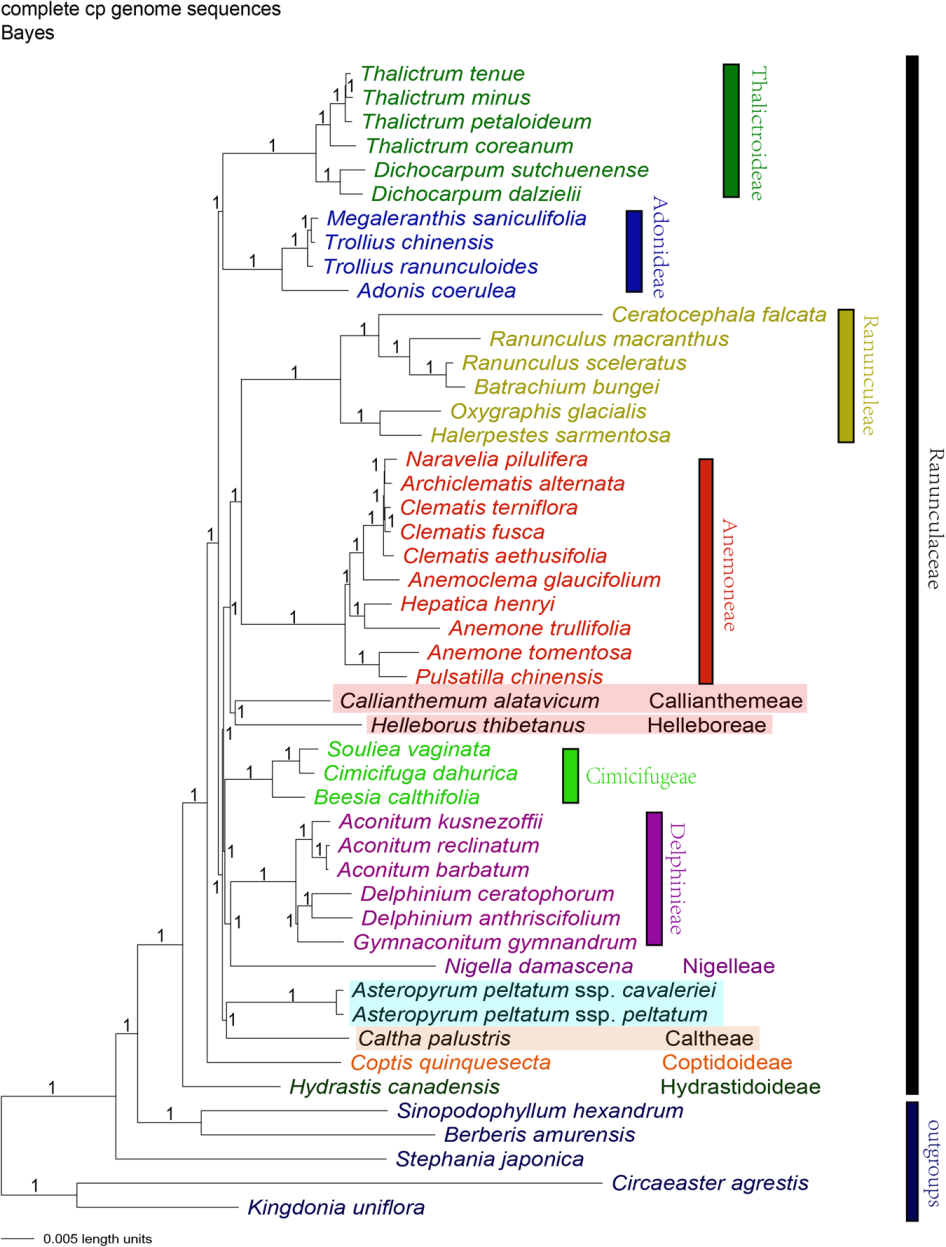
Phylogeny of Ranunculaceae species inferred from complete plastome sequences using Bayesian methods with partitioned modeling. The Bayesian phylograms show the posterior probability (PP) values on each node.

The topologies from the Bayesian and ML analyses were almost the same for each dataset, but the ML analyses also yielded less resolved and supported trees than the Bayesian method. Among all the 21 phylogenetic trees, the one obtained from the complete plastome sequence dataset with Bayesian method showed a fully resolved phylogeny with all the branches supported by PP value of 1 (Fig. 4). Therefore, the phylogenetic relationships discussed below were mainly based on this result.

## Discussions

After comparing the first diverged *Amborella*^36^ and other angiosperm species, we determined that a total number of 113 genes (with 17 genes in IR region) in the cp genome can be set as a primitive cp genome structure based on its prevalence in angiosperms. Then, we summarized the structural variation of the cp genomes (including gene composition, gene inversion and transposition, and IR expansion-contraction) across Ranunculaceae based on all the available data.

Structural variations of the cp genome within Ranunculaceae have been reported^11,12,15,20,37,38^. Using restriction site mapping, Johansson^38^ reported large inversions and gene loss in plastomes of *Adonis* species. In this study, one inversion in *A. coerulea*, which was identical with the *A. vernalis* inversion described by Johansson^38^, was confirmed and located in detail. Zhai *et al*. reported the same inversion in *A. sutchuenensis*^15^. We also detected one gene loss (*rpl32*) in *A. coerulea* (Supplementary Table S3). The inversion within *Adonis* plastomes could be a synapomorphy within the genus. Structural variations of cp genome have been reported within tribe Anemoneae by previous studies^11,12,15,20,37^. In comparison with *Adonis*, those cp genome structural variations of tribe Anemoneae differed in size and number. No evidence indicates that these inversions in the cp genomes of *Adonis* and tribe Anemoneae are phylogenetically related.

IR expansion-contraction is also an important process involved in cp genome variation within Ranunculaceae. Based on a broad comparison with other angiosperm species, ten genera (*Asteropyrum*, *Clematis*, *Archiclematis*, *Naravelia*, *Pulsatilla*, *Anemone*, *Anemoclema*, *Hepatica*, *Dichocarpum*, and *Hydrastis*) were found to exhibit IR expansion, whereas two genera (*Helleborus* and *Ceratocephala*) showed IR contraction. Some of these IR expansion events may be phylogenetically informative. For example, within the well supported tribe Anemoneae, a single IR-expansion, which may be a synapomorphy of the plastome variation in the tribe, was detected^12^. However, in other case, IR expansion-contraction may occur independently in some genera. For example, the cp genomes from two species of *Dichocarpum* showed different IR regions. One species, *D. dalzielii*, had a normal 17-gene IR, whereas the other, *D. sutchuenense*, carried expanded IR regions (19 genes) (Table 2). On the other hand, IR contraction and gene loss in *Helleborus* and *Ceratocephala* (Table 2) also evolved independently within Ranunculaceae because these two genera were separated phylogenetically (Fig. 4).

The genus *Asteropyrum* did not have gene inversions or transpositions in their cp genomes, but they showed significant IR expansion and carried the longest IR regions in the family (Table 2). Different gene compositions in IR regions of the two subspecies were detected (Fig. 2, Table 2). Two more genes (*rpoA* and *rps11*) were present in the IR region of ssp. *peltatum*, and this variation is stable within the subspecies. These phenomena suggested that plastome structural variation can occur not only among genera but also within a single species.

In the present study, an intermediate sample showed the same IR-SC boundary with *A. peltatum* ssp. *cavaleriei*. Morphologically, ssp. *peltatum* and ssp. *cavaleriei* can be easily distinguishable by their size and shape of leaves. Intermediate forms between the two subspecies in morphology and palynology are sometimes present at their overlapping zones^24^ and can be either more similar to ssp. *peltatum* or to ssp. *cavaleriei*. The intermediate individual sampled in this study was small in its leaf and scape size but with slightly five-angled leaves (Fig. 1E,F). According to Yuan & Yang^24^, it can be recognized as an intermediate form but more similar to ssp. *cavaleriei*. The IR-SC boundary indicated that this intermediate individual could be a hybrid one between the two subspecies with ssp. *cavaleriei* as its maternal parent.

A large number of molecular phylogenetic as well as several phylogenomic analyses have been conducted for Ranunculaceae^33–35,39–45^. However, these studies have suffered from poor resolution largely due to an insufficient phylogenetic signal or insufficient sampling. Zhai *et al*. carried out a well sampled plastid phylogenomic study on Ranunculaceae^15^, and they obtained a better phylogenetic framework of the family. However, this study did not separate the plastid sequence in detail and the substitution model was tested without partitioning. Thus, we still do not know the diversification and phylogenetic resolving ability of different partition of the plastid genome sequences.

In this study, phylogenetic relationships within Ranunculaceae were inferred from six separate datasets, as well as a complete plastome sequence data. Among all the six separate datasets, intergenic spacer data showed the highest rate of informative sites (45.37%). Whereas, the IR region showed the lowest rate of informative sites (8.50%), indicating that it is the most conserved region in the entire plastid genome (Table 3). The phylogenetic resolution of IR region was also shown to be the worst among the seven datasets (Supplementary Fig. S4) because of its small number of phylogenetic signals.

All the phylogenetic trees inferred by different datasets were largely congruent (Supplementary Fig. S4). *Hydrastis* and *Coptis* consistently appeared as the earliest diverged lineages in the family. Major clades, including subfamily Thalictroideae, tribe Adonideae, tribe Ranunculeae, tribe Anemoneae, tribe Cimicifugeae, and tribe Delphinieae were resolved in all the analyses. However, the systematic positions of *Asteropyrum*, *Caltha*, *Helleborus*, *Callianthemum* and *Nigella* were found to be unstable and often had low statistical support values using the six separate datasets. These genera may have undergone unusual evolutionary processes, such as ancient hybridization or rapid radiation, coupled with their origin and evolution process.

Because of the different rates and patterns of nucleotide substitutions among the cp genome sequences^46^, data partitioning methods are required for phylogenetic reconstruction to ensure the accuracy of the analysis^2,47–50^. Our results showed that Bayesian analyses with partitioned models always obtained the best resolved Ranunculaceae phylogeny for each dataset. Using Bayesian analysis, the complete plastome dataset obtained a fully resolved phylogeny for Ranunculaceae (Fig. 4), which was better resolved than all the previous molecular and phylogenomic studies^15,33–35,39–45^.

In general, the Bayesian phylogeny inferred from the complete cp genome sequences is largely congruent with the phylogeny obtained by Cossard *et al*.^35^ Using eight DNA fragments from the chloroplast, mitochondrial, and nuclear genomes, Cossard *et al*. identified the sister relationship of subfamilies Thalictroideae and Adonideae with insignificant statistical support (PP = 0.80)^35^. This relationship was not resolved by most previous molecular phylogenetic studies^32,33,40–42,44,51,52^. In the present study, our plastid phylogenomic analysis resolved the sister relationship between subfamilies Thalictroideae and Adonideae (Fig. 4), and supported the hypothesis of Cossard *et al*.

From the Bayesian analysis, we detected the sister relationship of *Callianthemum* and *Helleborus* (Fig. 4) which was not resolved by all the previous studies of Ranunculaceae phylogeny^32,33,40–42,44,51,52^. The clade of *Callianthemum* and *Helleborus* was found to be related to a well-supported clade of tribe Ranunculeae + tribe Anemoneae. The sister relationship of tribe Cimicifugaea and the tribe Delphinieae + tribe Nigellaea clade (Fig. 4) was resolved by our analysis. This relationship was previously reported by Hoot but without sufficient statistical support^41^.

The phylogenetic position of *Asteropyrum* has been disputed for a century and still remains to be resolved by now^20,24,32,35,53^. In this study, the sister relationship of *Asteropyrum* and *Caltha* was discovered using the complete cp genome datasets with ML and Bayesian methods. This clade was further found to be first diverged from subfamily Ranunuloideae (Fig. 4). Wang *et al*. also proposed sister relationship of *Asteropyrum* and *Caltha* by combining molecular and morphological data, but their results was not statistically supported^32^. It is noteworthy that Cossard *et al*. resolved a sister relationship of *Asteropyrum* and *Callianthemum* with a strong support value using a combination of chloroplast, mitochondrial, and nuclear genes^35^. This unexpected result was solely contributed by the nuclear gene RanaCYL1 dataset. The contradictory results by our cp genome analysis and nuclear RanaCYL1 data may be caused by either an ancient hybridization event or incomplete lineage sorting of nuclear RanaCYL1. Further studies focused on this issue may be conducted using additional markers from nuclear genome.

In the Bayesian phylogram, many major clades of Ranunculaceae had a very short branch length, including the *Asteropyrum* + *Caltha* clade, Cimicifugeae + Delphinieae + Nigelleae clade, and Ranunculeae to Nigelleae clade (Fig. 4). This indicated ancient lineage radiation of the subfamily Ranunculoideae, which was also proposed by Zhai *et al*.^15^ Thus, it is not surprising that previous molecular phylogenetic analyses using limited molecular markers failed to resolve the phylogenetic framework of Ranunculaceae. Further phylogenomic studies using additional evidence, such as mitochondrial and nuclear genomic data, may help to deeper our understanding of the evolution of Ranunculaceae.

## Conclusion

The two subspecies of *Asteropyrum* carried quite different plastid genomes with different IR-SC borders and much sequence variation. The plastome sequence of *Asteropyrum* has the longest IR regions in the family. Unlike *Adonis* and species from tribe Anemoneae, no gene inversions and transpositions were detected in the *Asteropyrum* cp genome. The complete cp genome showed excellent suitability for drawing phylogenetic inferences within Ranunculaceae. The complete cp genome sequence, as well as its structural variation (gene inversions-transpositions and IR expansion-contraction), can provide abundant phylogenetic information for the family. In contrast, non-coding regions have excessive variations and a high level of noise, so they are not suitable for resolving the generic relationships within the family. As mentioned by Ma *et al*., analyses in plastid genomic studies should always be conducted using partitioned datasets, and parsimony analysis often obtains unsatisfactory results^2^. Our plastid phylogenomic inferences, which were obtained using the complete cp genome sequence and Bayesian analysis, provided a better resolved phylogenetic framework for Ranunculaceae in comparison with all the previous studies. *Asteropyrum* was detected to be closely related to *Caltha*, and unusual ancient evolutionary processes for *Asteropyrum* was also suggested by our findings.

## Methods

### Taxon sampling and sequencing

Fresh leaves were collected from plants from field by the authors (Table 4) and the samples of *Nigella* and *Helleborus* were from cultivated plants (seeds were obtained from Mr. Fothergill’s Seeds Limited Company). The leaf samples were dried with tell-tale silica-gel. Samples from both subspecies of *Asteropyrum* were collected from different populations. We also sequenced 25 other species from Ranunculaceae and mined 21 accessions of complete cp genomes (including five outgroups from Ranunculales) from GenBank for comparative analysis. The samples covered 31 genera and represented most tribes of Ranunculaceae, but did not include the basal clades of Trib. Glaucidieae and Trib. Xanthorhizeae^33^ (Table 4). For all leaf tissue samples, total genomic DNA was isolated using the CTAB method^54^ and assessed by agarose gel electrophoresis.

**Table 4:** Information regarding the sequenced *Asteropyrum* materials and other samples from Ranunculaceae*.

Species	Sample locality	Voucher (Herbarium)	Genbank accession	Reference
*Aconitum barbatum*	Songshan, Beijing, China	*L. Xie* 20140820 (BJFC)	MK253470	This study
*Aconitum kuznezoffii*	Donglingshan, Beijing, China	*L. Xie* 20150709 (BJFC)	MK253471	This study
*Aconitum reclinatum*	NA	NA	MF186593	Kong *et al*.^69^
*Adonis coerulea*	Xiaojin, Sichuan, China	*H. J. Liu* I-1109 (BJFC)	MK253469	This study
*Anemoclema glaucifolium*	Shangri-la, Yunnan, China	*B. Xu-*M417-090 (SWFC)	MH205609	Liu *et al*.^12^
*Anemone tomentosa*	Barkam, Sichuan, China	*H. J. Liu* I”-1080 (BJFC)	MG001339	Liu *et al*.^12^
*Anemone trullifolia*	Dinggye, Xizang, China	*PE2013* Tibet 2588 (PE)	MH205608	Liu *et al*.^12^
*Archiclematis alternata*	Nyalam, Xizang, China	*PE2010* Tibet 963 (PE)	MG675221	Liu *et al*.^11^
*Asteropyrum peltatum* ssp. *peltatum*	Emei, Sichuan, China	*L. Xie* 20150094 (BJFC)	MG734862	This study
*A. peltatum* ssp. *cavaleriei*	YiBin, Sichuan, China	*L. Xie* 2014-YB013 (BJFC)	MG734861	This study
*Batrachium bungei*	Ali, Xizang, China	*Tibet2013* 4048 (PE)	MK253468	This study
*Beesia calthifolia*	Emei, Sichuan, China	*L. Xie* 2015-EM19(BJFC)	MK253467	This study
*Berberis amurensis*	NA	NA	KM057374	Unpublished
*Callianthemum alatavicum*	Urumqi, Xinjiang, China	*Z. Z. Yang* 0524 (BJFC)	MK253466	This study
*Caltha palustris*	Emei, Sichuan, China	*L. Xie* 2015-EM25 (BJFC)	MK253465	This study
*Ceratocephala falcata*	Altay, Xinjiang, China	*L. Xie* 2016003 (BJFC)	MK253464	This study
*Cimicifuga dahurica*	Xiaowutai, Hebei, China	*XWT2011033*	MK253463	This study
*Circaeaster agrestis*	Shennongjia, Hubei, China	*Y. X. Sun* 1510 (HIB)	KY908400	Sun *et al*.^3^
*Clematis aethusifolia*	Donglingshan, Beijing, China	*L. Xie* 2015014 (BJFC)	MK253462	This study
*Clematis fusca* var. *coreana*	NA	NA	KM652489	Park & Park^70^
*Clematis terniflora*	Huzhou, Zhejiang, China	Unknown number (HZU)	KJ956785	Li *et al*.^71^
*Coptis quinquesecta*	Jinping, Yunnan, China	*LP*174738 (HZU)	MG585353	Zhang *et al*.^72^
*Delphinium ceratophorum*	Binchuan, Yunnan, China	*Q. He* 2017091301 (BJFC)	MK253460	This study
*Delphinium anthriscifolium*	Huixian, Henan, China	*L. Xie* 20160402 (BJFC)	MK253461	This study
*Dichocarpum dalzielii*	Yibin, Sichuan, China	*L. Xie* sc2014008 (BJFC)	MK253459	This study
*Dichocarpum sutchuenense*	Taibai, Shaanxi, China	*H. J. Liu* BE05 (BJFC)	MK253458	This study
*Gymnaconitum gymnandrum*	NA	NA	KT964697	Unpublished
*Halerpestes sarmentosa*	Shidu, Beijing, China	FS2015001	MK253457	This study
*Helleborus thibetanus*	Cult. in Beijing Forest. Univ.	*J. He* C2018001 (BJFC)	MK253456	This study
*Hepatica_henryi*	Emei, Sichuan, China	*L. Xie* 2015EM039 (BJFC)	MG001340	Liu *et al*.^12^
*Hydrastis canadensis*	NA	NA	KY085918	Unpublished
*Kingdonia uniflora*	Meixian, Shaanxi, China	*Y. X. Sun* 1606 (HIB)	KY908401	Sun *et al*.^3^
*Megaleranthis saniculifolia*	Mt. Sobaek, Korea	Unknown number (Korea University Herbarium)	FJ597983	Kim *et al*.^73^
*Naravelia pilulifera*	Longzhou, Guangxi, China	*L. Xie* 201511 (BJFC)	MK253455	This study
*Nigella_damascena*	Cult. in Beijing Forest. Univ.	*J. He* C2018002 (BJFC)	MK253454	This study
*Oxygraphis glacialis*	Urumqi, Xinjiang, China	*Z. Z. Yang* 0422 (BJFC)	MK253453	This study
*Pulsatilla chinensis*	Songshan, Beijing, China	*L. Xie* 2015YQ002 (BJFC)	NC_039452	Liu *et al*.^12^
*Ranunculus macranthus*	NA	NA	DQ359689	Raubeson *et al*.^74^
*Ranunculus sceleratus*	Mentougou, Beijing, China	*L. Xie* 2014098 (BJFC)	MK253452	This study
*Sinopodophyllum hexandrum*	NA	NA	MG593048	Ye *et al*.^75^
*Souliea vaginata*	Shangri-la, Yunnan, China	*L. Xie* 2012-X110 (BJFC)	MK253451	This study
*Stephania japonica*	Wuhan, Hubei, China	*Y. X. Sun* 1405 (HIB)	KU204903	Sun *et al*.^76^
*Thalictrum coreanum*	Gangwon-do, Korea	NA	KM206568	Park *et al*.^77^
*Thalictrum minus*	Wulingshan, Hebei, China	*L. Xie* 20171102 (BJFC)	MK253450	This study
*Thalictrum petaloideum*	Donglingshan, Beijing, China	*L. Xie* 20150705 (BJFC)	MK253449	This study
*Thalictrum tenue*	Mentougou, Beijing, China	*L. Xie* 20160502 (BJFC)	MK253448	This study
*Trollius chinensis*	NA	NA	KX752098	Unpublished
*Trollius ranunculoides*	Shangri-la, Yunnan, China	*L. Xie* 2012-X137 (BJFC)	MK253447	This study

*All the newly sequenced samples used in this study are not endangered species and were not obtained in conserved area.

The total DNA samples of the 27 newly sequenced species were sent to Novogene (http://www.novogene.com, China) for library construction and next-generation sequencing. Short-insert (350 bp) paired-end read library preparation and 2 × 150 bp sequencing were performed on an Illumina (HiSeq4000) genome analyzer platform. Approximately 2–4 Gb of raw data for each species were first filtered using the FASTX-Toolkit to obtain high-quality clean data by removing adaptors and low-quality reads (http://hannonlab.cshl.edu/fastx_toolkit/download.html). The remaining clean reads (high-quality reads) were sent to the authors for further analysis.

### Chloroplast genome assembly and annotation

For the clean reads, BLAT analysis was used to exclude nuclear and mitochondrial reads using published plastid genome sequences from Ranunculaceae as references^55^. Next, *de novo* assembly was performed using Geneious R11 with a medium-low sensitivity setting^56^ to assemble plastid genome sequences. For most of the tested samples, only one contig (approximately 130 kb) was obtained by de novo assembly. If more than one smaller contig was obtained, the whole-genome reads were mapped to those contigs using the Fine Tuning program in Geneious R11 (iterating up to 100 times) to fill gaps. Contigs were connected by overlapping their terminal sequences using the Repeat Finder program implemented in Geneious R11. Sanger sequencing was also used to bridge gaps when necessary. When a 130 kb contig (including SSC, IR, and LSC) was built for each sample, the IR region was determined using the Repeat Finder program, after which the IR region was inverted and copied manually to construct the complete cp genome sequence. Because great variation was found at the IR-SC boundaries in the two *Asteropyrum* plastomes, we subjected these regions to Sanger sequencing. Broader population sampling of the two *Asteropyrum* subspecies, as well as an intermediate individual, was applied to assess the stability of the variation at IR-SC boundaries using Sanger sequencing (Supplementary Tables S4 and S5).

Complete plastid genomes were annotated using the Unix program Plann 1.1.2^57^ and manually verified using Geneious Annotate R11 and the online program Blast^58^. The cp genome sequences and annotations were uploaded to GenBank using Bankit (https://www.ncbi.nlm.nih.gov/books/NBK63590/). Accession numbers are shown in Table 4. Illustrations of all the newly sequenced plastomes were obtained using the Organellar Genome DRAW tool^59^.

### Comparative chloroplast genomic analyses for two subspecies of *Asteropyrum* and their relatives

The IR/SC boundaries of *Asteropyrum* and the other tested species were illustrated and compared with other outgroups to address IR expansion-contraction. MAFFT v7.309^60^ was used to align the plastome sequences, whereas mVISTA^61^ was used to export visual results to allow evaluation of the structural similarity of plastomes. The alignment programs applied in mVISTA were LAGAN, which produces true multiple alignments regardless of whether they contain inversions or not, and Shuffle-LAGAN, which can detect rearrangements and inversions^62,63^. The sequence diversification of the two subspecies of *Asteropyrum* was assessed by comparing the two aligned sequences. The rate of identical sites was calculated using Geneious R11.

### Plastid phylogenomic analysis

In this study, 48 cp genome sequences, including those of five outgroups from Ranunculales, were aligned for phylogenomic analysis. The gene orders for the cp genome sequences of Tribe Anemoneae and *Adonis* (in which inversions and/or transposition regions were present) were shuffled in the same order with other Ranunculaceae and outgroup species. The following data sets were applied for phylogenetic reconstruction: complete cp genome sequence (with only one IR region), LSC, SSC, IR, coding regions (CDs), intron, and intergenic spacer regions. All datasets were aligned using MAFFT v7.309^60^. Ambiguous alignments and sites with more than 80% missing data^4^ were deleted automatically using a Python script (https://github.com/HeJian151004/get_homology).

Maximum Parsimony (MP) analysis was conducted for all the seven datasets using PAUP v4.0b10^64^. Characters were treated as unordered and equally weighted, whereas gaps were treated as missing data. Branch-and-Bound or a 1000-replicate heuristic search was applied with simple addition, and tree bisection reconnection branch swapping with MUL-trees was utilized to search the MP tree(s). Statistical support was assessed by 1000 bootstrap replicates with 1000 random taxon addition replicates and 10 trees held at each step.

Maximum likelihood (ML) analyses were carried out with RAxML-HPC2 v8.2.10^65^ performed on the online server (https://www.phylo.org/). The GTR + G model was applied for all datasets as suggested by the software instructions (see RAxML manual). The statistical value was inferred using the combined rapid bootstrap method (1000 replicates).

Bayesian inference (BI) was performed with MrBayes v3.2.3^66^ using partitioned substitution models tested by PartitionFinder v2.1.1^67^ with a minimum subset size of 5000, because excessively parameter-rich models often cause calculation problems in Bayesian analysis and fail to converge^2,50^. The best substitution models and data partition schemes were selected by Akaike information criterion (AIC)^68^. Two parallel independent Markov chain Monte Carlo (MCMC) chains were run, each of which consisted of three hot chains and one cold chain for 5,000,000 generations. The trees were sampled and saved every 100 generations. The MCMC convergence was tested by calculating the standard deviation value of split frequencies (less than 0.01) and by assessing the convergence of the parameter values of the two runs. The first 25% of trees were discarded as burn-in, and the remaining trees were used to generate the consensus tree.

## Supplementary information

Supplementary information

Supplementary dataset

## Data Availability

The new sequenced plastome sequenced are all deposited at NCBI and seen in Table 4.
