# Supplementary material for: Structural variation of the complete chloroplast genome and plastid phylogenomics of the genus Asteropyrum (Ranunculaceae)
Source: Sci Rep. 2019 Oct 25;9:15285. doi: 10.1038/s41598-019-51601-2 (PMC6814708; doi:10.1038/s41598-019-51601-2)
Supplement: Supplementary file 1 — Supplementary information [file 41598_2019_51601_MOESM1_ESM.pdf]

Structural variation of the complete chloroplast genome and plastid phylogenomics of the genus *Asteropyrum* (Ranunculaceae)

Jian He, Min Yao, Ru-Dan Lyu, Le-Le Lin, Hui-Jie Liu, Lin-Ying Pei, Shuang-Xi Yan, Lei Xie, Jin Cheng

Supplementary information

Supplementary Table S1: Information regarding plastid genome sequence assembly for all newly sequenced Ranunculaceae species.

Supplementary Table S2: Summary of the plastome features of newly sequenced Ranunculaceae species.

Supplementary Table S3: Detailed IR information of all tested Ranunculaceae and outgroup species.

Supplementary Table S4: *Asteropyrum* materials used for IR-SC boundary checking.

Supplementary Table S5: Primers designed for IR-SC boundary checking using Sanger sequencing.

Supplementary Figure S1: Chloroplast genome maps of newly sequenced Ranunculaceae species. The inversion on the plastome of *Adonis* is marked. Gene inversions and transpositions in the species of tribe Anemoneae were provided in Liu et al. [11]and are not shown here.

Supplementary Figure S2: Sequence alignment of the complete plastome sequences of Ranunculaceae and outgroup samples compared in this study using the mVISTA program and Shuffle-LAGAN method. A cut-off of 70% similarity was used for the plot, and the Y-scale represents the percent similarity ranging from 50–100%. Blue represents coding regions, and pink represents non-coding regions.

Supplementary Figure S3: Comparison of the LSC, IRs and SSC boundary regions for all tested Ranunculaceae and outgroup species.

Supplementary Figure S4: Twenty-one phylogenetic trees inferred by seven data sets and three methods of parsimony, maximum likelihood, and Bayesian methods.

Supplementary Table S1 Information regarding plastid genome sequence assembly for all newly sequenced Ranunculaceae species.

Species	Clean data (Gb)	Clean reads	Filtered cp reads	Contigs before iteration map	Contigs after iteration map	Length of contigs	PCR primer for gap filling
Aconitum barbatum	2.3	6344204	51642	2	2	69256+61414	20abF:TACCGAACTGAACTAAGAGCGC; 20abR:AGTCAAGAACAAAACAAAGAAGGGG
Aconitum kuznezoffii	3.2	8826707	72148	2	1	129650	
Adonis coerulea	2.3	6349523	51914	4	2	47911+82932	102rps16lF:CCCTGGAGACGCCCTTGTAT; 102rps16lR:GGGACGAGCTTGCTTCTTGA
Batrachium bungei	2.4	6619957	54112	5	1	130830	
Beesia calthifolia	3.1	8556436	69987	4	2	54030+76587	103psblcF:AGAAGAAATAATACTCTACCCCGATCT; 103psblcR:ATCTCTTTCACCAGGCCTCG
Callianthemum alatavicum	5.6	15449964	125266	3	1	130660	
Caltha palustris	2.6	7177618	58442	5	2	6017+122672	46trnQKF:CCAAAACCCGTTGCCTTACC; 46trnQKR:GGCTAAGAGCAAAAAGAACAAAGG
Ceratocephala falcata	3	8271707	66872	6	1	126756	
Cimicifuga dahurica	2.4	6622797	53583	4	1	132890	
Clematis aethusifolia	2.8	7720067	62559	1	1	128657	
Delphinium ceratophorum	2.7	7444709	60392	2	1	127785	
Delphinium anthriscifolium	3.5	9656901	78466	1	1	129200	
Dichocarpum dalzielii	2.1	5788694	47418	2	1	126674	
Dichocarpum sutchuenense	3.3	9105961	73902	2	1	127868	
Halerpestes sarmentosa	2.4	6623494	53898	5	1	132342	
Helleborus thibetanus	2.9	7999831	65434	1	1	130045	
Naravelia pilulifera	3	8272986	67427	1	1	128559	
Nigella damascena	2.2	6068894	49725	2	1	130155	
Oxygraphis glacialis	6.2	17104519	139004	2	1	131509	
Ranunculus sceleratus	3.6	9930175	80925	4	1	131122	
Souliea vaginata	3.1	8548566	69272	2	1	131924	
Thalictrum minus	2.3	6343047	51845	3	1	129819	
Thalictrum petaloideum	3.2	8832639	71601	2	1	129496	
Thalictrum tenue	3.2	8825760	71851	2	1	129699	
Trollius ranunculoides	2.3	6343823	51663	3	1	133266	

Supplementary Table S2 Summary of the plastome features of newly sequenced Ranunculaceae species.

Category	*Aconitum barbatum*	*Aconitum kuznezoffii*	*Adonis coerulea*	*Batrachium bungei*	*Beesia calthifolia*
Total cp genome size (bp)	156761	155832	157033	156082	158117
Length of large single copy region (bp)	87638	86339	86545	85436	87564
Length of inverted repeat region (bp)	26090	26282	26087	25352	26500
Length of small single copy region (bp)	16943	16929	18314	19942	17553
Coding size (bp)	99572	99504	99738	101265	100586
Intron size (bp)	15405	14592	15451	14760	15409
Spacer size (bp)	41784	41736	41844	40057	42122
Total GC content (%)	38.0	38.1	37.9	37.8	38.2
GC content of LSC (%)	36.0	36.3	36.1	36.0	36.4
GC content of IR (%)	43.0	43.0	43.1	43.5	43.2
GC content of SSC (%)	32.8	32.7	31.3	31.3	32.4
Total number of genes	112	111	112	112	112
Number of protein encoding genes	78(comparing to *Amborella*, lack rpl32)	77(lack rps16 and rpl32)	78(lack rpl32)	78(lack infA)	78(lack rpl32)
Number of tRNA genes	30	30	30	30	30
Number of rRNA genes	4	4	4	4	4
Number of genes duplicated in IR	17	17	17	17	17

Supplementary Table S2 Summary of the plastome features of newly sequenced Ranunculaceae species (continued).

Category	*Callianthemum alatavicum*	*Caltha palustris*	*Ceratocephala falcata*	*Cimicifuga dahurica*	*Clematis aethusifolia*
Total cp genome size (bp)	156538	155325	150821	159362	159598
Length of large single copy region (bp)	86901	84123	83576	88465	79518
Length of inverted repeat region (bp)	25978	26421	24165	26572	31041
Length of small single copy region (bp)	17681	18360	18915	17753	17998
Coding size (bp)	99964	100845	97081	100862	104607
Intron size (bp)	14127	15515	13731	15180	15313
Spacer size (bp)	42447	38965	40009	43320	39678
Total GC content (%)	38.2	38.1	38.4	38.1	37.9
GC content of LSC (%)	36.5	36.4	36.8	36.3	36.3
GC content of IR (%)	43.2	43.0	43.7	43.1	42.0
GC content of SSC (%)	31.9	32.0	32.3	32.3	31.4
Total number of genes	112	113	113	112	112
Number of protein encoding genes	78(lack rps16)	79	78(lack infA)	78(lack rpl32)	79
Number of tRNA genes	30	30	31(double trnfM)	30	29(lack trnT-UGU)
Number of rRNA genes	4	4	4	4	4
Number of genes duplicated in IR	17	17	16	17	23

Supplementary Table S2 Summary of the plastome features of newly sequenced Ranunculaceae species (continued).

Category	*Delphinium ceratophorum*	*Delphinium anthriscifolium*	*Dichocarpum dalzielii*	*Dichcarpum sutchuenense*
Total cp genome size (bp)	154245	155077	153109	155390
Length of large single copy region (bp)	84801	85871	82694	82716
Length of inverted repeat region (bp)	26560	25977	26535	27622
Length of small single copy region (bp)	16324	17252	17345	17430
Coding size (bp)	99225	99334	100441	101626
Intron size (bp)	14549	14608	14485	14584
Spacer size (bp)	40471	41135	38183	39180
Total GC content (%)	38.3	38.1	38.5	38.4
GC content of LSC (%)	36.3	36.2	36.8	36.6
GC content of IR (%)	43.0	43.2	43.2	43.0
GC content of SSC (%)	32.9	32.6	32.4	32.2
Total number of genes	111	111	112	112
Number of protein encoding genes	77(lack rpl32, rps16)	77(lack rpl32, rps16)	78(lack rpl32)	78(lack rpl32)
Number of tRNA genes	30	30	30	30
Number of rRNA genes	4	4	4	4
Number of genes duplicated in IR	17	17	17	19

Supplementary Table S2 Summary of the plastome features of newly sequenced Ranunculaceae species (continued).

Category	*Halerpestes sarmentosa*	*Helleborus thibetanus*	*Naravelia pilulifera*	*Nigella damascena*	*Oxygraphis glacialis*
Total cp genome size (bp)	157299	154944	159513	155222	156503
Length of large single copy region (bp)	85663	85962	79312	87107	86298
Length of inverted repeat region (bp)	25057	24999	31054	25167	25094
Length of small single copy region (bp)	21522	18984	18093	17781	20017
Coding size (bp)	100315	100363	104592	99821	100283
Intron size (bp)	15609	15133	17025	15502	15497
Spacer size (bp)	41375	39448	37896	39899	40723
Total GC content (%)	37.9	37.8	37.9	38.8	37.8
GC content of LSC (%)	36.1	35.9	36.3	37.2	35.9
GC content of IR (%)	43.4	43.4	42.0	43.5	43.3
GC content of SSC (%)	32.2	31.6	31.2	33.5	31.8
Total number of genes	112	113	112	112	112
Number of protein encoding genes	78(lack infA)	79	79	78(lack rpl32)	78(lack infA)
Number of tRNA genes	30	30	29(lack trnT-UGU)	30	30
Number of rRNA genes	4	4	4	4	4
Number of genes duplicated in IR	17	16	23	17	17

Supplementary Table S2 Summary of the plastome features of newly sequenced Ranunculaceae species (continued).

Category	*Ranunculus sceleratus*	*Souliea vaginata*	*Thalictrum minus*	*Thalictrum petaloideum*	*Thalictrum tenue*	*Trollius ranunculoides*
Total cp genome size (bp)	156324	158357	156201	155876	156103	159666
Length of large single copy region (bp)	85835	87686	85685	85326	85507	88194
Length of inverted repeat region (bp)	25302	26533	26482	26480	26504	26500
Length of small single copy region (bp)	19885	17605	17579	17590	17588	18472
Coding size (bp)	101072	100187	99844	99854	99890	98114
Intron size (bp)	15648	15151	15328	15268	15341	15430
Spacer size (bp)	39604	43019	41029	40754	40872	46122
Total GC content (%)	37.9	38.0	38.4	38.4	38.4	38.0
GC content of LSC (%)	36.1	36.2	36.6	36.6	36.6	36.3
GC content of IR (%)	43.5	43.0	43.2	43.2	43.2	43.1
GC content of SSC (%)	31.7	32.3	32.5	32.5	32.4	31.8
Total number of genes	112	112	112	112	112	113
Number of protein encoding genes	78(lack infA)	78(lack rpl32)	78(lack rpl32)	78(lack rpl32)	78(lack rpl32)	79
Number of tRNA genes	30	30	30	30	30	30
Number of rRNA genes	4	4	4	4	4	4
Number of genes duplicated in IR	17	17	17	17	17	17

Supplementary Table S3 Detailed IR information of all tested Ranunculaceae and outgroup species.

Species	Gene number in IR	Protein-coding genes number in IR	tRNA genes number in IR	rRNA genes number in IR	Border of IRa with LSC	Border of IRa with SSC	Border of IRb with SSC	Border of IRb with LSC	Length of IR	Comparing to Amborella
Aconitum barbatum	17	6	7	4	rps19	trnN-GUU — ndhF	ycf1	rpl2 — trnH-GUG	26090	
Aconitum kuznezoffii	17	6	7	4	rps19	trnN-GUU — ndhF	ycf1	rpl2 — trnH-GUG	26282	
Aconitum reclinatum	17	6	7	4	rps19 — rpl2	trnN-GUU — ndhF	ycf1	rpl2 — trnH-GUG	26061	
Adonis coerulea	17	6	7	4	rps19 — rpl2	trnN-GUU — ndhF	ycf1	rpl2 — trnH-GUG	26087	
Amborella trichopoda	17	6	7	4	rps19 — rpl2	trnN-GUU — ndhF	ycf1	rpl2 — trnH-GUG	26651	
Anemoclema glaucifolium	24	13	7	4	rpl36	ndhF	ycf1	infA — rps4	31256	add infA rps8 rpl14 rpl16 rps3 rpl22 rps19
Anemone tomentosa	24	13	7	4	rpl36	trnN-GUU — ndhF	ycf1	rps4	31490	add infA rps8 rpl14 rpl16 rps3 rpl22 rps19
Anemone trullifolia	24	13	7	4	rpl36 — infA	trnN-GUU — ndhF	ycf1	infA — rps4	31022	add infA rps8 rpl14 rpl16 rps3 rpl22 rps19
Asteropyrum peltatum spp. cavaleriei	25	14	7	4	rps11	ndhF	ycf1	rpl36 — trnH-GUG	31429	add rpl36 infA rps8 rpl14 rpl16 rps3 rpl22 rps19
Asteropyrum peltatum spp. peltatum	27	16	7	4	petD — rpoA	ndhF	ycf1	rpoA — trnH-GUG	32659	add rpoA rps11 rpl36 infA rps8 rpl14 rpl16 rps3 rpl22 rps19
Batrachium bungei	17	6	7	4	rps19 — rpl2	trnN-GUU — ndhF	ycf1 — trnN-GUU	rpl2 — trnH-GUG	25352	
Beesia calthifolia	17	6	7	4	rps19 — rpl2	trnN-GUU — ndhF	ycf1	rpl2 — trnH-GUG	26500	
Berberis amurensis	32	21	7	4	clpP — psbB	trnN-GUU — ndhF	ycf1	psbB — trnH-GUG	37152	add psbB psbT psbN psbH petB petD rps11 rpl36 infA rps8 rpl14 rpl16 rps3 rpl22 rps19
Callianthemum alatavicum	17	6	7	4	rps19	ndhF	ycf1	rpl2 — trnH-GUG	25978	
Caltha palustris	17	6	7	4	rps19	ndhF	ycf1	rpl2 — trnH-GUG	26421	
Ceratocephala falcata	16	5	7	4	rpl2	trnN-GUU — ndhF	ycf1 — trnN-GUU	trnH-GUG	24165	loss rpl2
Cimicifuga dahurica	17	6	7	4	rps19	trnN-GUU — ndhF	ycf1	rpl2 — trnH-GUG	26572	
Circaeaster agrestis	21	8	9	4	rpl22 — rps19	trnQ-UUG — ndhF	ycf1	rps19 — trnH-GUG	28023	add rps19 trnQ-UUG trnL-UAG rpl32
Clematis aethusifolia	23	12	7	4	infA	ndhF	ycf1	rps4	31041	add rps8 rpl14 rpl16 rps3 rpl22 rps19
Clematis alternata	23	12	7	4	infA	ndhF	ycf1	rps8 — rps4	31037	add rps8 rpl14 rpl16 rps3 rpl22 rps19
Clematis fusca	24	13	7	4	rpl36 — infA	ndhF	ycf1	rps4	31039	add infA rps8 rpl14 rpl16 rps3 rpl22 rps19
Clematis terniflora	24	13	7	4	rpl36 — infA	ndhF	ycf1	infA — rps4	31045	add infA rps8 rpl14 rpl16 rps3 rpl22 rps19
Coptis quinquesecta	17	6	7	4	rps19	ndhF	ycf1	rpl2 — trnH-GUG	26442	
Delphinium anthriscifolium	17	6	7	4	rps19 — rpl2	trnN-GUU — ndhF	ycf1	rpl2 — trnH-GUG	25977	
Delphinium ceratophorum	17	6	7	4	rps19	ndhF	ycf1	rpl2 — trnH-GUG	26560	
Dichocarpum dalzielii	17	6	7	4	rps19	ndhF	ycf1	rpl2 — trnH-GUG	26535	
Dichocarpum sutchuenense	19	8	7	4	rps3	ndhF	ycf1	rpl22 — trnH-GUG	27622	add rpl22 rps19
Gymnaconitum gymnandrum	17	6	7	4	rps19	trnN-GUU — ndhF	ycf1	rpl2 — trnH-GUG	26140	
Halerpestes sarmentosa	17	6	7	4	rps19	trnN-GUU — ndhF	ycf1 — trnN-GUU	rpl2 — trnH-GUG	25057	
Helleborus thibetanus	16	5	7	4	rpl2	ndhF	ycf1	rpl23 — trnH-GUG	24999	loss rpl2
Hepatica henryi	24	13	7	4	rpl36 — infA	trnN-GUU — ndhF	ycf1	rps4	31039	add infA rps8 rpl14 rpl16 rps3 rpl22 rps19
Hydrastis canadensis	18	7	7	4	rpl22	trnN-GUU — ndhF	ycf1	rps19 — trnH-GUG	27032	add rps19
Kingdonia uniflora	19	8	7	4	rpl22 — rps19	rps15 — trnL-UAG	rpl32 — rps15	rps19 — trnH-GUG	31109	loss ndhB add rps19 ycf1 rps15
Megaleranthis saniculifolia	17	6	7	4	rps19	trnN-GUU — ndhF	ycf1	rpl2 — trnH-GUG	26608	
Naravelia pilulifera	23	12	7	4	infA	trnN-GUU — ndhF	ycf1	rps8 — rps4	31054	add rps8 rpl14 rpl16 rps3 rpl22 rps19
Nigella damascena	17	6	7	4	rps19 — rpl2	ndhF	ycf1 — trnN-GUU	rpl2 — trnH-GUG	25167	
Oxygraphis glacialis	17	6	7	4	rps19	trnN-GUU — ndhF	ycf1	rpl2 — trnH-GUG	25094	
Pulsatilla chinensis	24	13	7	4	rpl36 — infA	trnN-GUU — ndhF	ycf1	rps4	31115	add infA rps8 rpl14 rpl16 rps3 rpl22 rps19
Ranunculus macranthus	17	6	7	4	rps19 — rpl2	trnN-GUU — ndhF	ycf1	trnH-GUG	25791	
Ranunculus sceleratus	17	6	7	4	rps19 — rpl2	trnN-GUU — ndhF	ycf1 — trnN-GUU	rpl2 — trnH-GUG	25302	
Sinopodophyllum hexandrum	17	6	7	4	rps19	trnN-GUU — ndhF	ycf1	rpl2 — trnH-GUG	25950	
Souliea vaginata	17	6	7	4	rps19	trnN-GUU — ndhF	ycf1	rpl2 — trnH-GUG	26533	
Stephania japonica	17	6	7	4	rps19	trnN-GUU — ndhF	ycf1	rpl2 — trnH-GUG	24340	
Thalictrum coreanum	17	6	7	4	rps19	trnN-GUU — ndhF	ycf1	rpl2 — trnH-GUG	26403	
Thalictrum minus	17	6	7	4	rps19	trnN-GUU — ndhF	ycf1	rpl2 — trnH-GUG	26482	
Thalictrum petaloideum	17	6	7	4	rps19	trnN-GUU — ndhF	ycf1	rpl2 — trnH-GUG	26480	
Thalictrum tenue	17	6	7	4	rps19	trnN-GUU — ndhF	ycf1	rpl2 — trnH-GUG	26504	
Trollius chinensis	17	6	7	4	rps19	trnN-GUU — ndhF	ycf1	rpl2 — trnH-GUG	26627	
Trollius ranunculoides	17	6	7	4	rps19	trnN-GUU — ndhF	ycf1	rpl2 — trnH-GUG	26500	

Supplementary Table S4 *Asteropyrum* materials used for IR-SC boundary checking.

Species	Sample locality	No. of individuals	Voucher (Herbarium)
Asteropyrum peltatum ssp. *peltatum*	Medog, Xizang, China	1	*X.X. Zhu* s.n. (KUN)
A. peltatum ssp. *peltatum*	Baoxing, Sichuan, China	2	*L. Xie* 2015-BX024 (BJFC)
A. peltatum ssp. *cavaleriei*	YiBin, Sichuan, China	2	*L. Xie* 2014-YB016 (BJFC)
A. peltatum ssp. *cavaleriei*	Dujiangyan, Sichuan, China	1	*L. Xie* 2012-DJ002 (BJFC)
A. peltatum ssp. *cavaleriei*	Nanchuan, Chongqing, China	1	*L. Xie* 2006-CQ041 (BJFC)
Intermediate form	YiBin, Sichuan, China	1	*L. Xie* 2014-YB019 (BJFC)

Supplementary Table S5 Primers designed for IR-SC boundary checking using Sanger sequencing.

Position	Region length	Primer name	Primers
ssp. *cavaleriei*: rps11-trnH	1008 bp	RIR2F	CAGCCGCAAATGTGATTCGT
		RIR2R	ATTGTGAATCCACCATGCGC
ssp. *peltatum*: rpoA-trnH	487 bp	92RIR2F	CCAATCAGAATTGCCTTCCAGG
		92RIR2R	ATTGTGAATCCACCATGCGC

Supplementary Figure S1

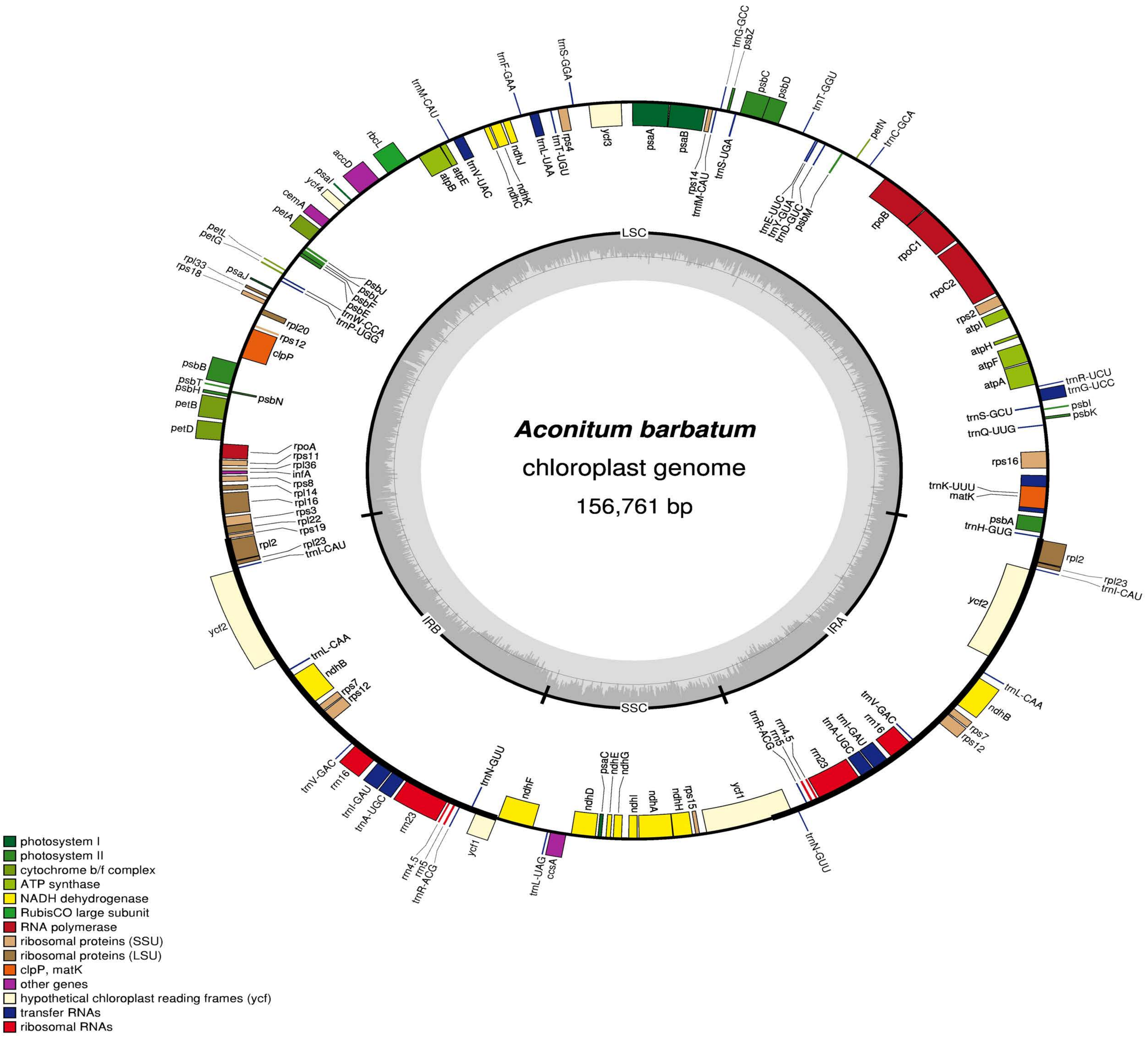

Supplementary Figure S1 (continue)

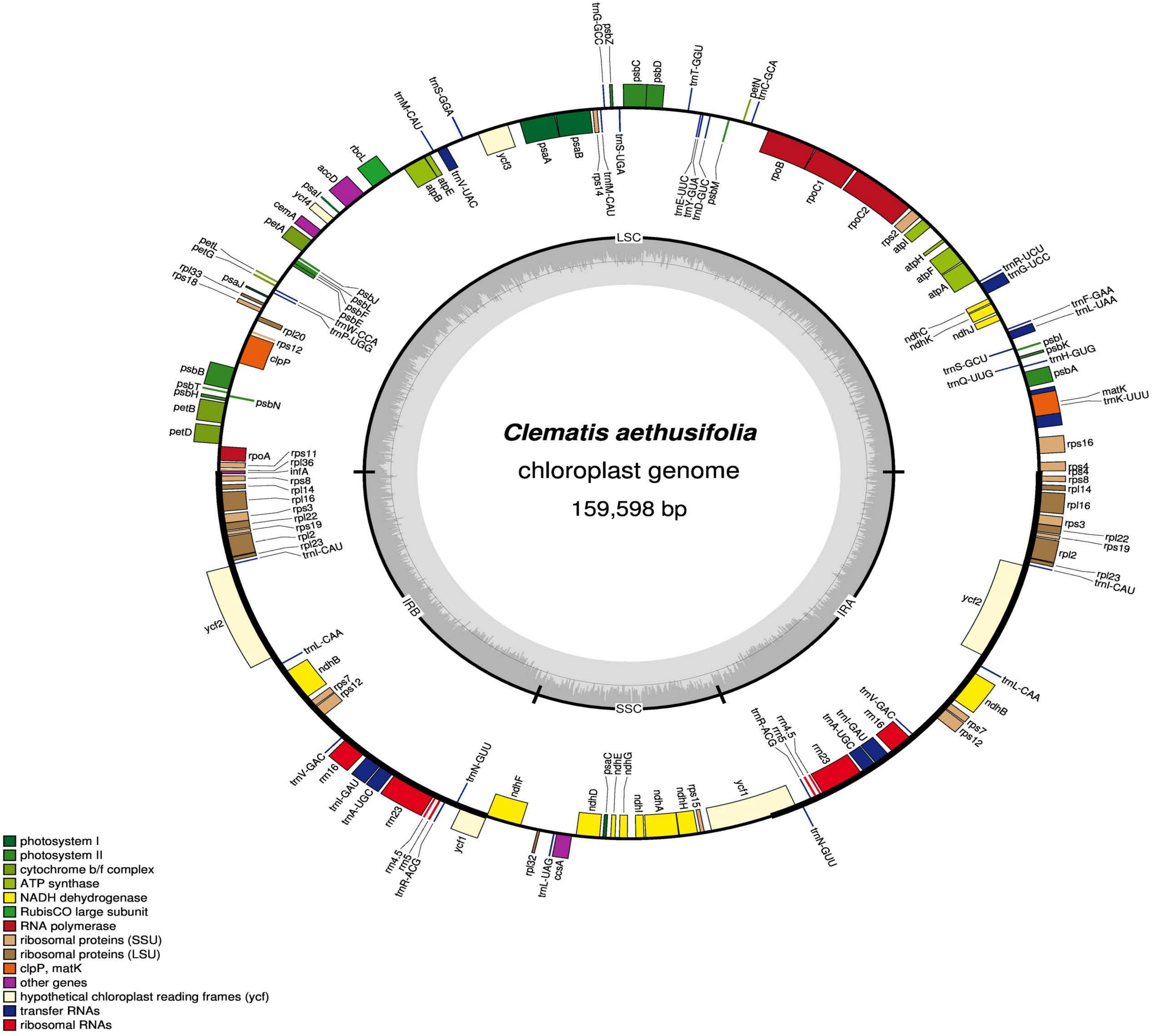

Supplementary Figure S1 (continue)

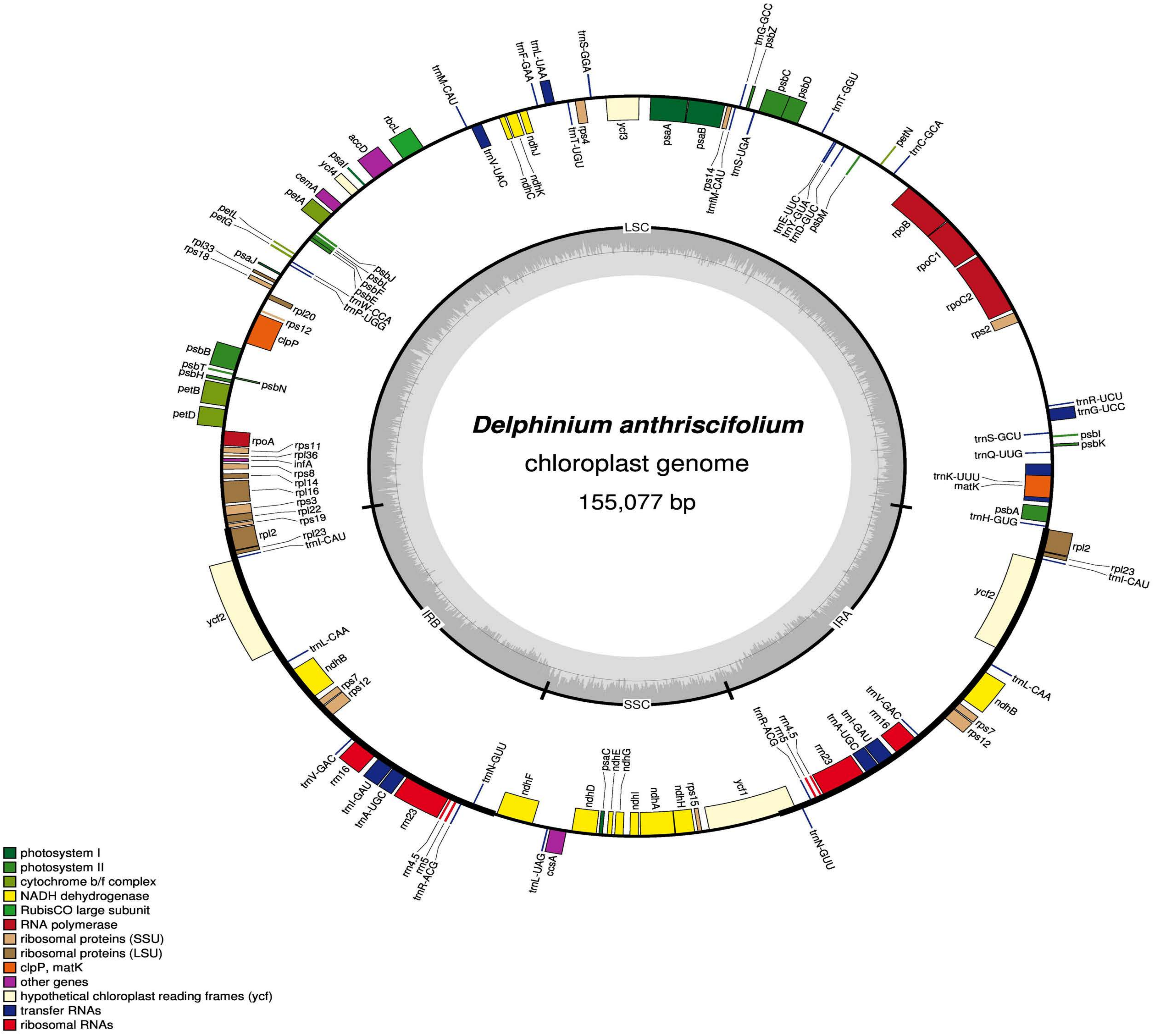

Supplementary Figure S1 (continue)

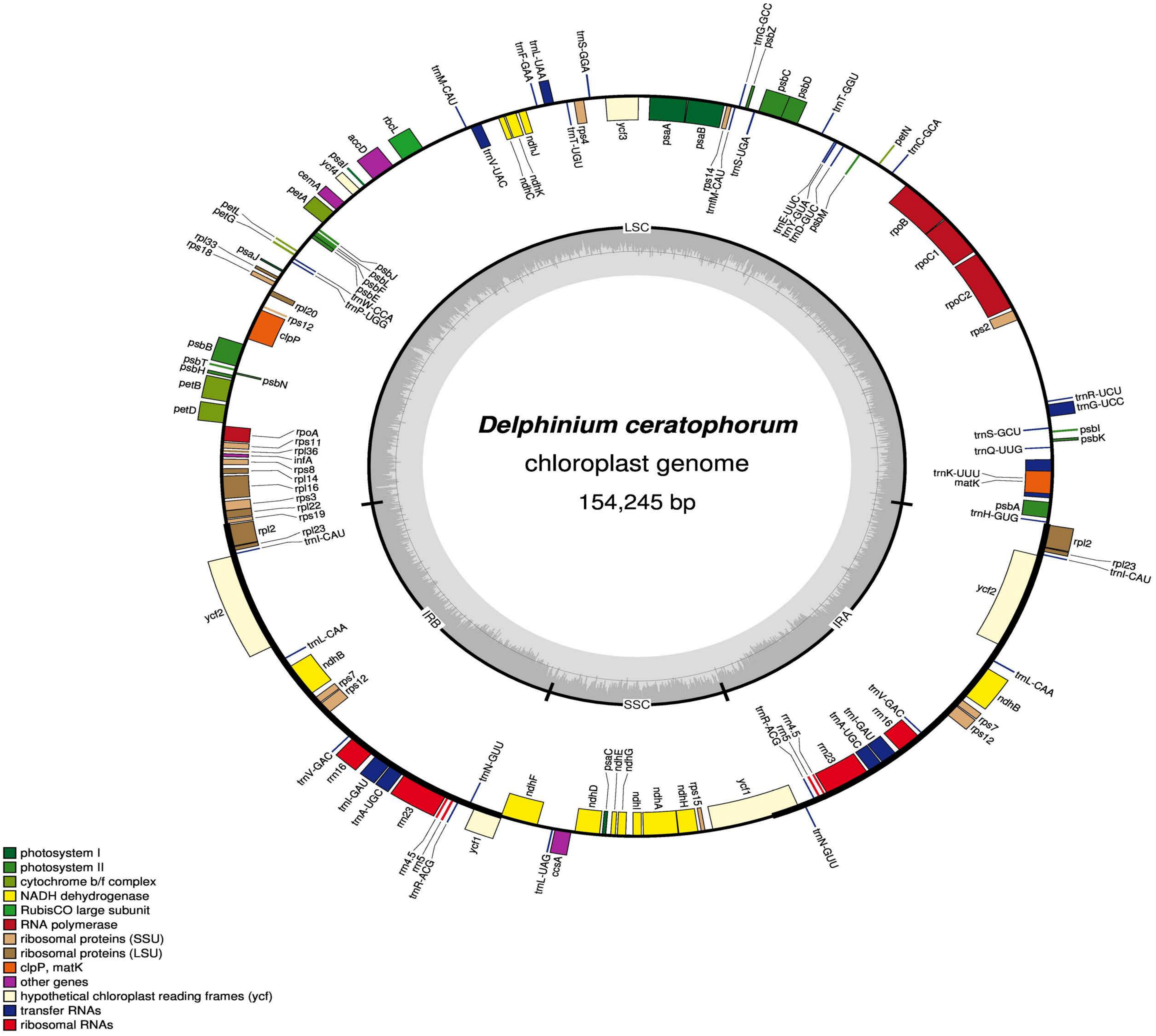

Supplementary Figure S1 (continue)

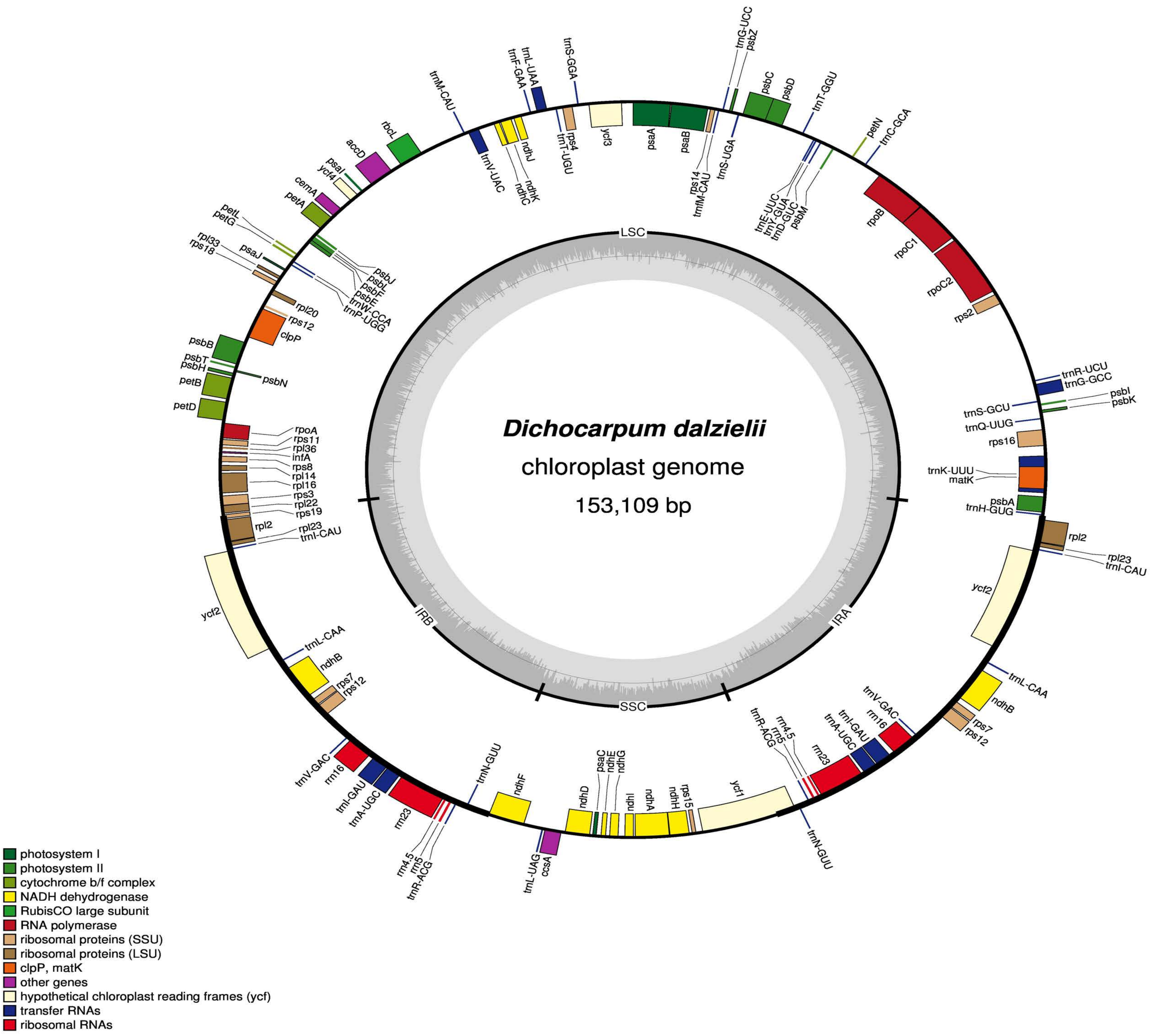

Supplementary Figure S1 (continue)

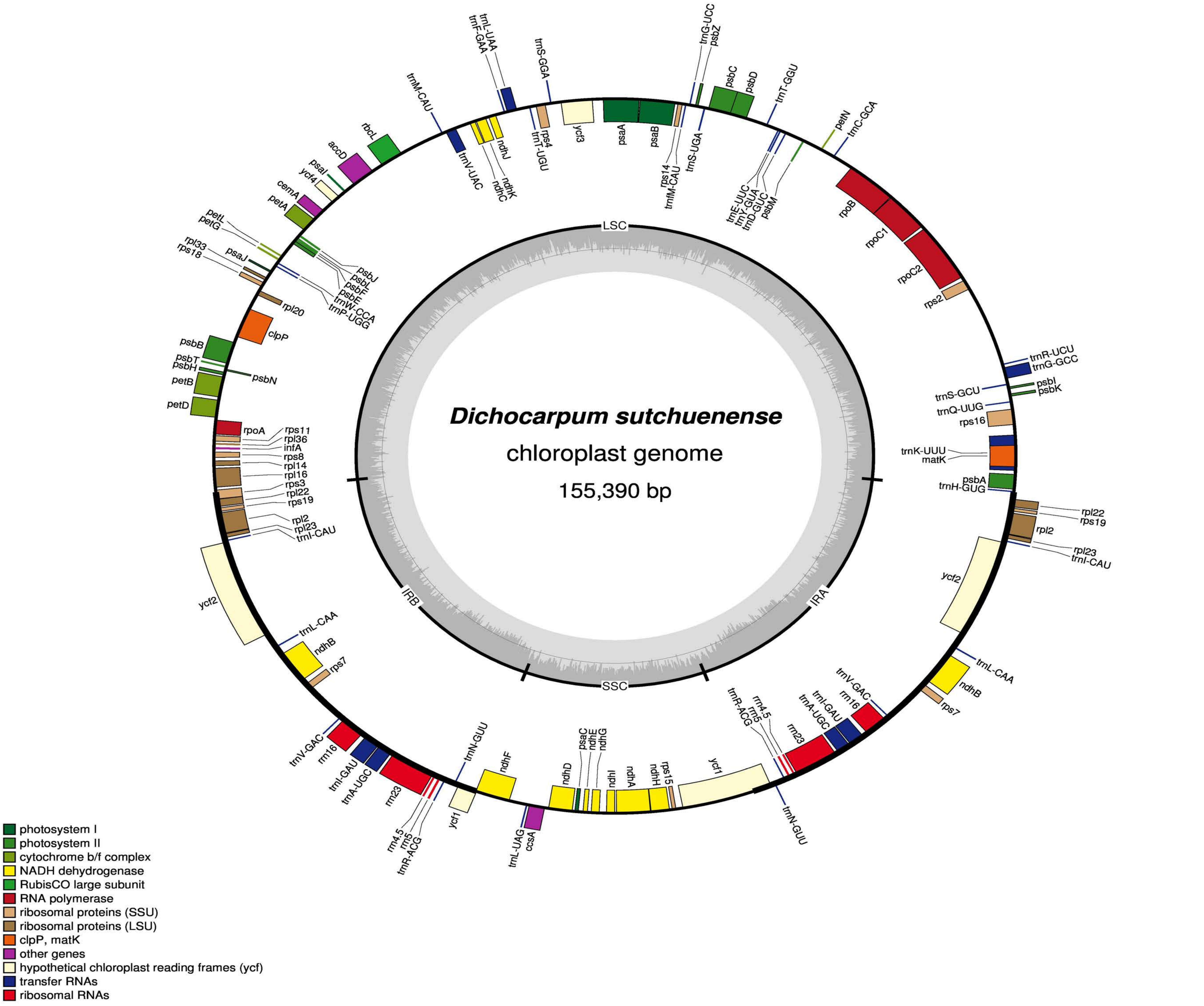

Supplementary Figure S1 (continue)

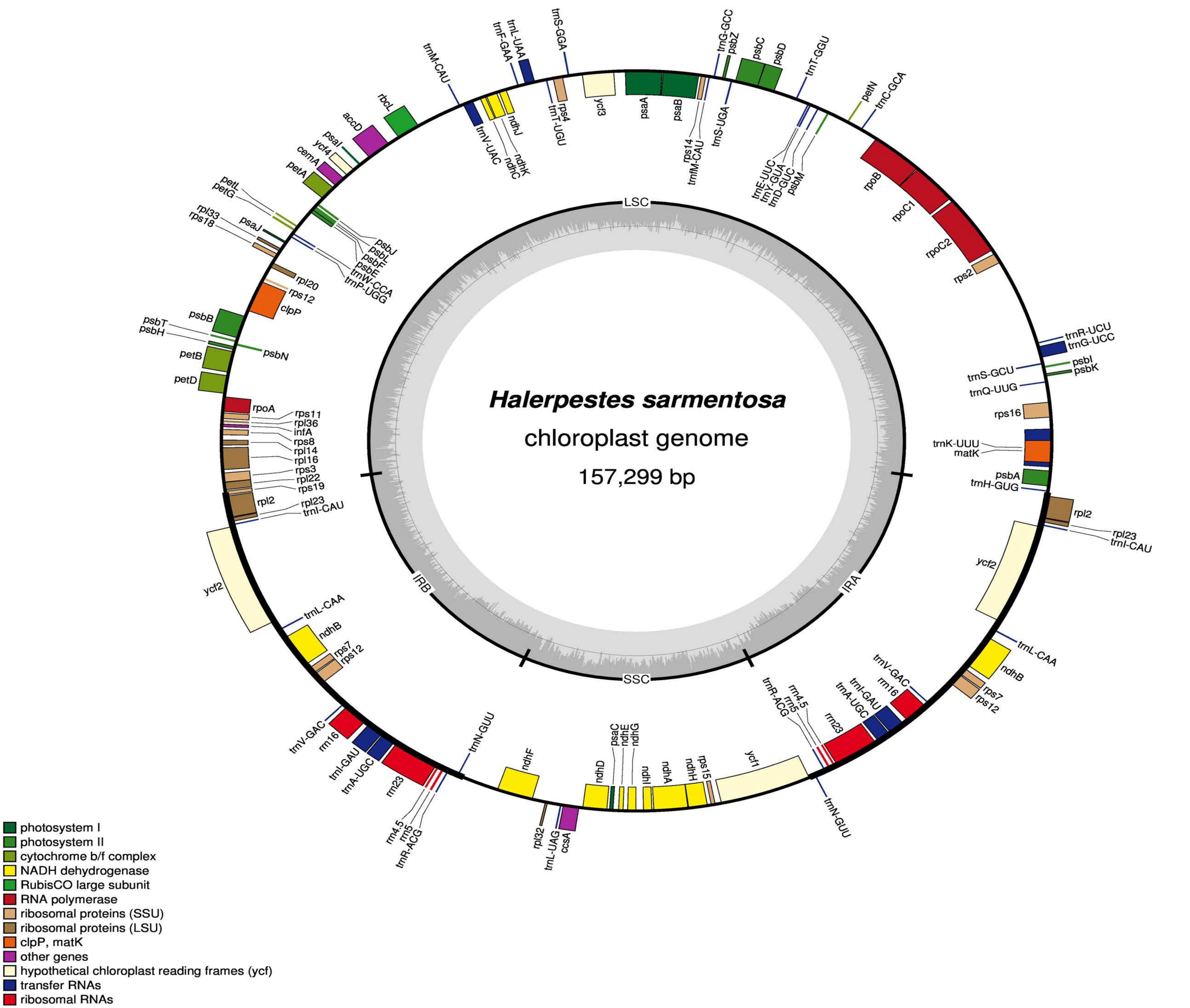

Supplementary Figure S1 (continue)

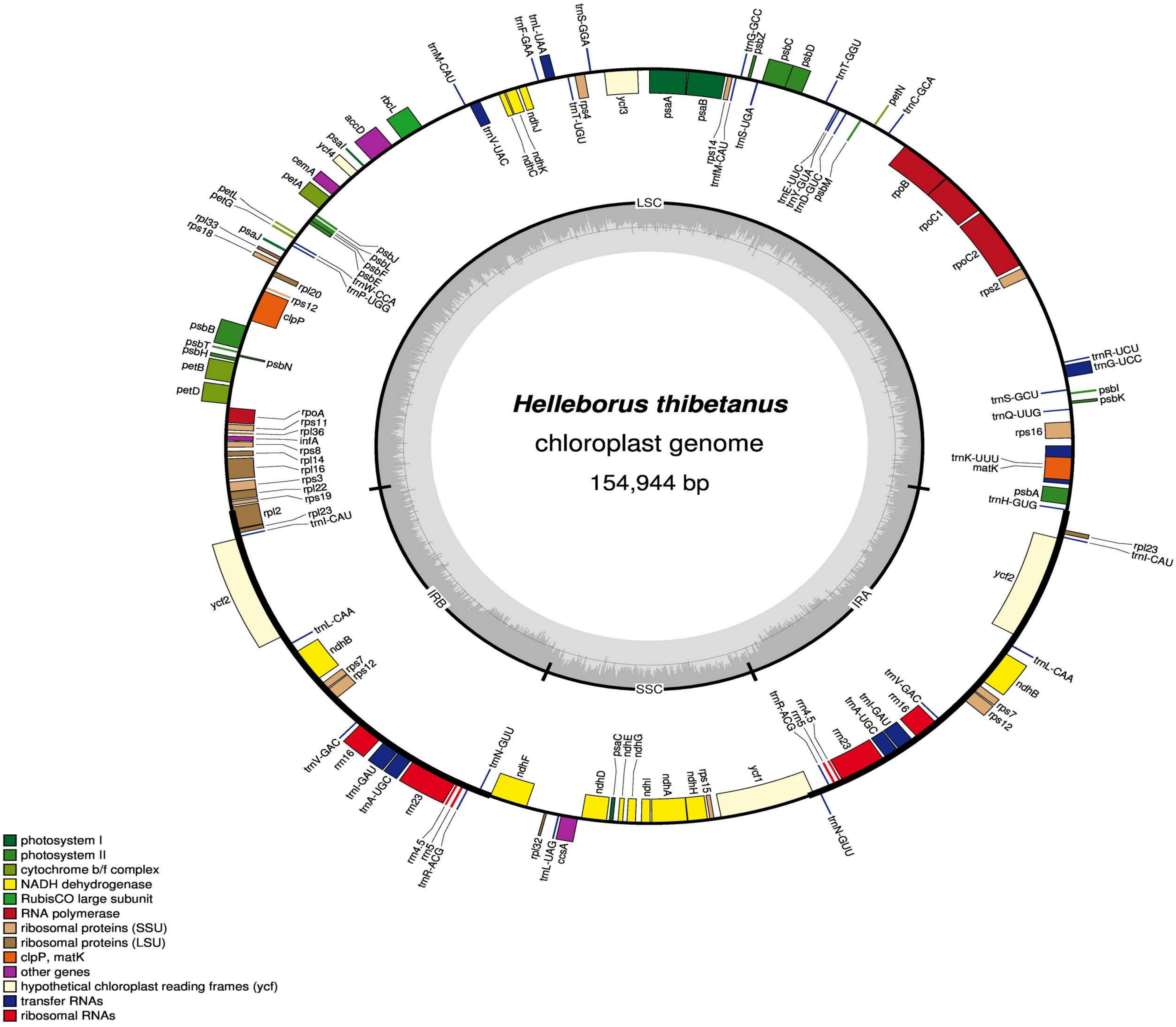

Supplementary Figure S1 (continue)

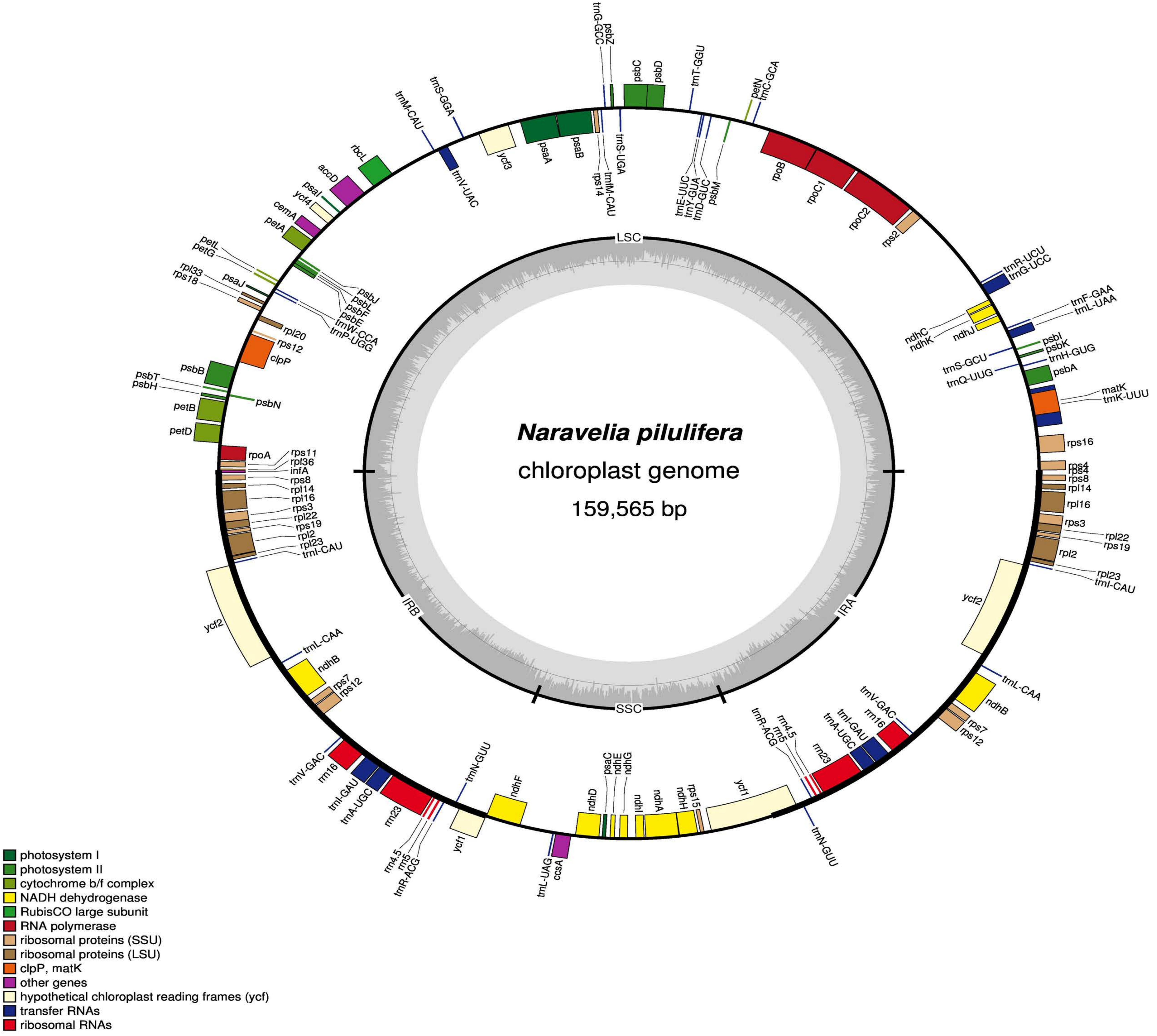

Supplementary Figure S1 (continue)

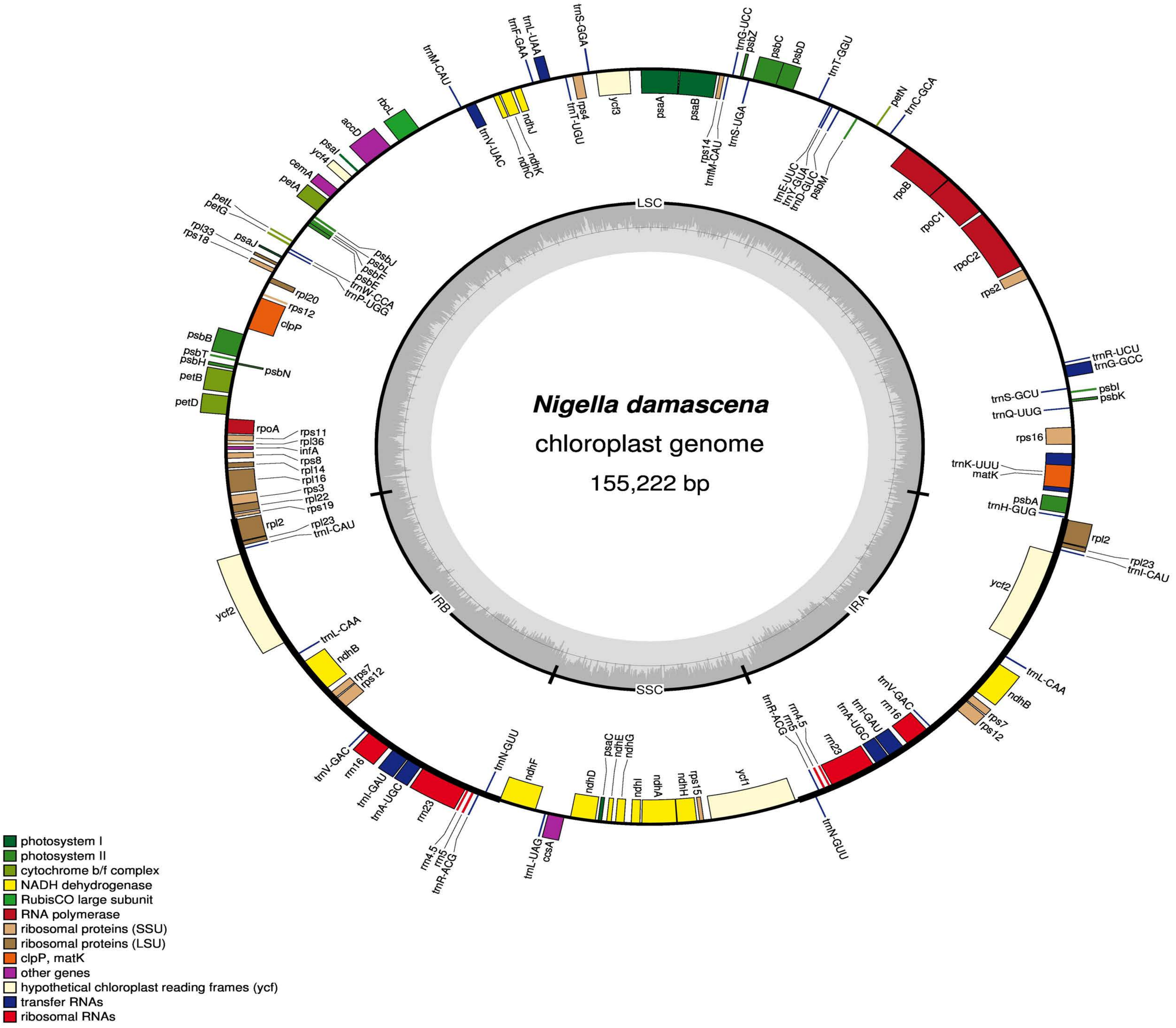

Supplementary Figure S1 (continue)

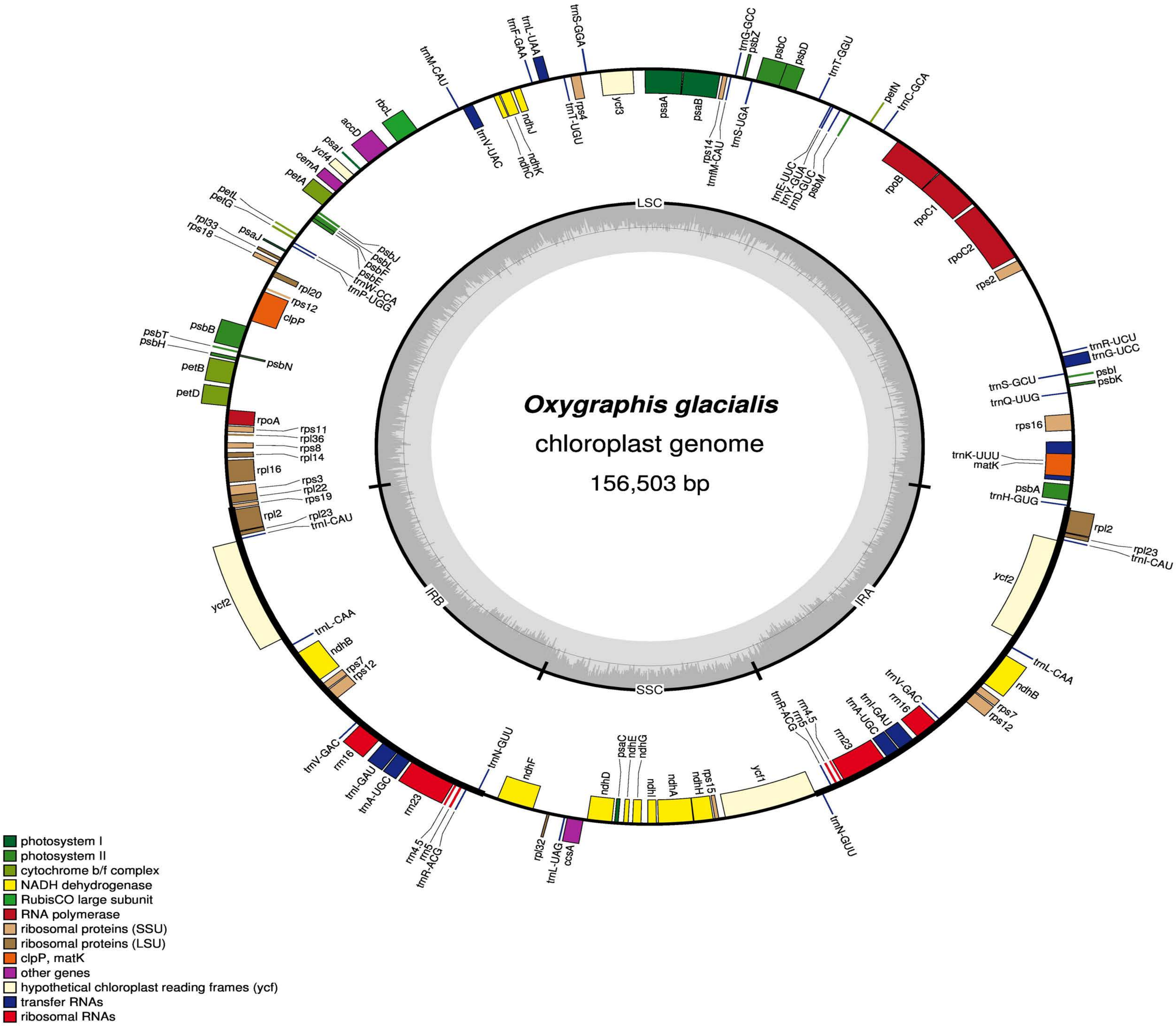

Supplementary Figure S1 (continue)

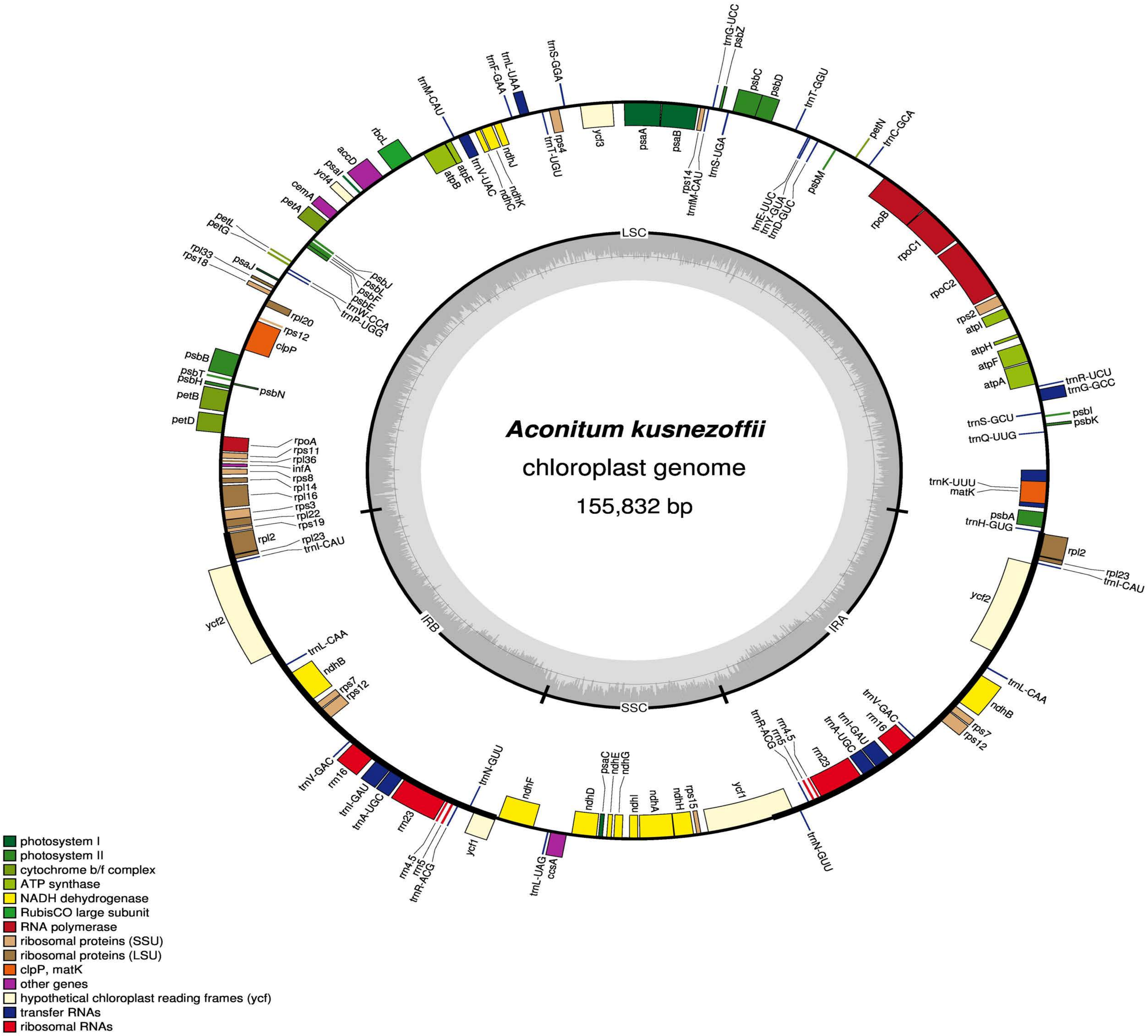

Supplementary Figure S1 (continue)

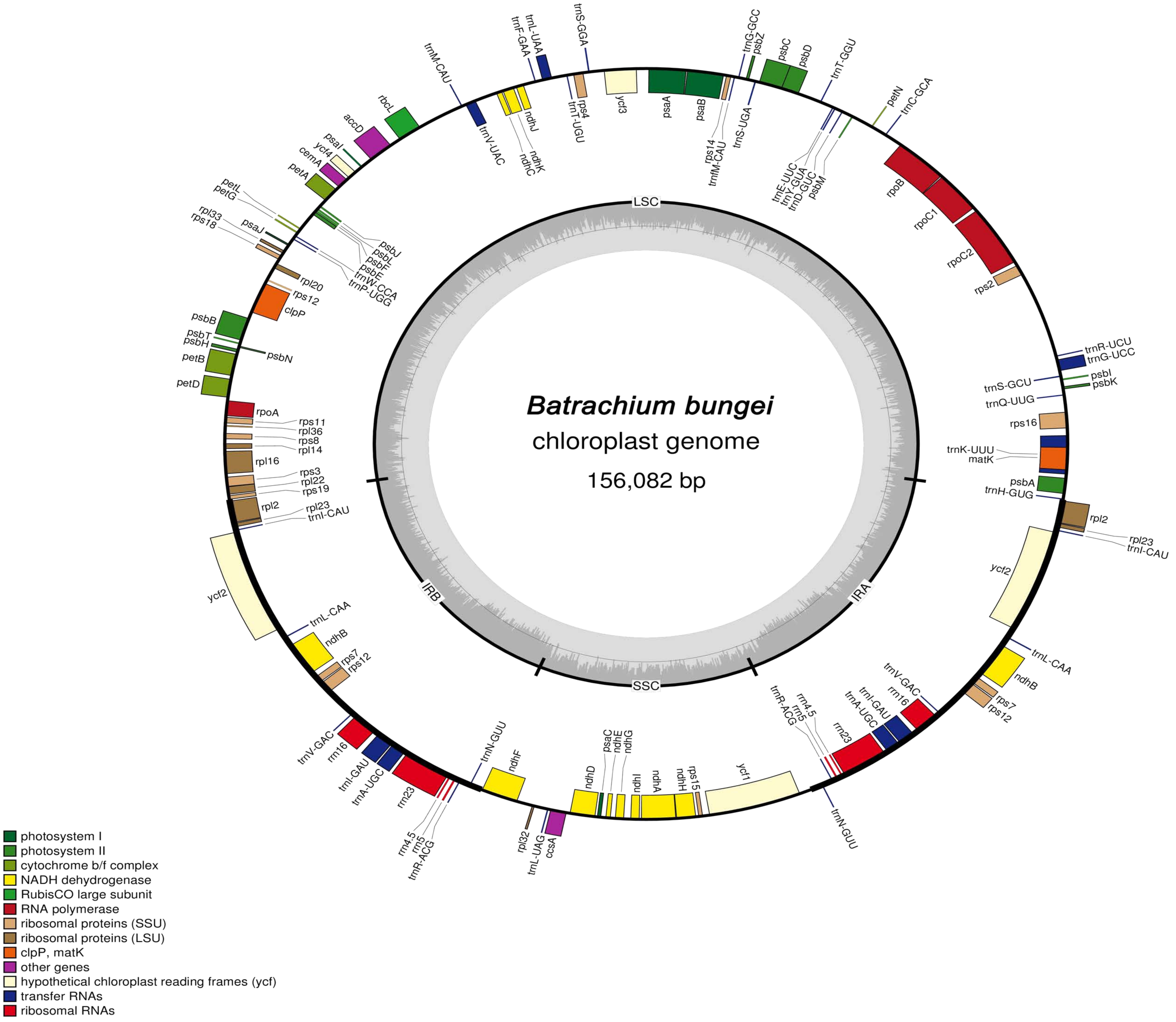

Supplementary Figure S1 (continue)

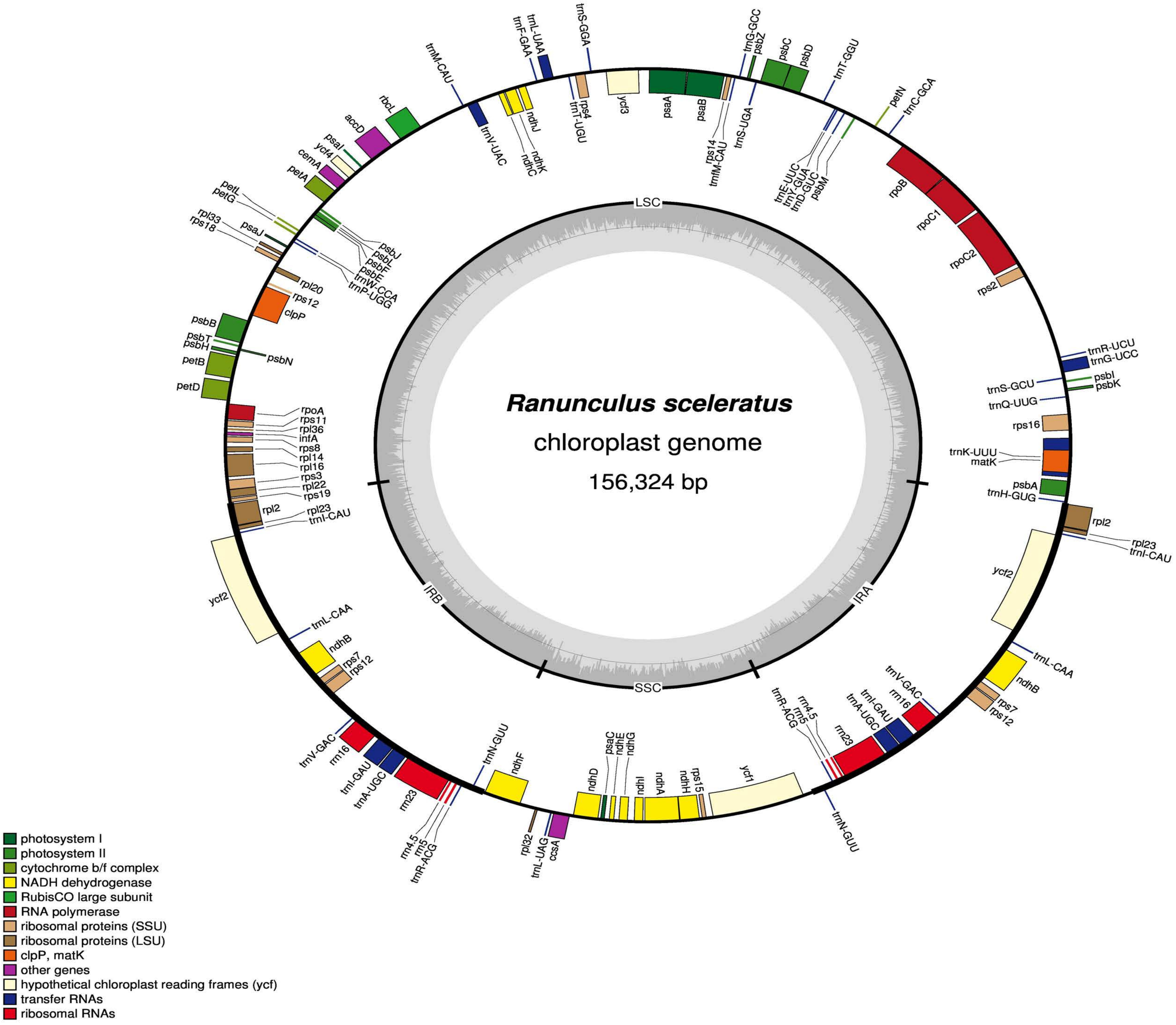

Supplementary Figure S1 (continue)

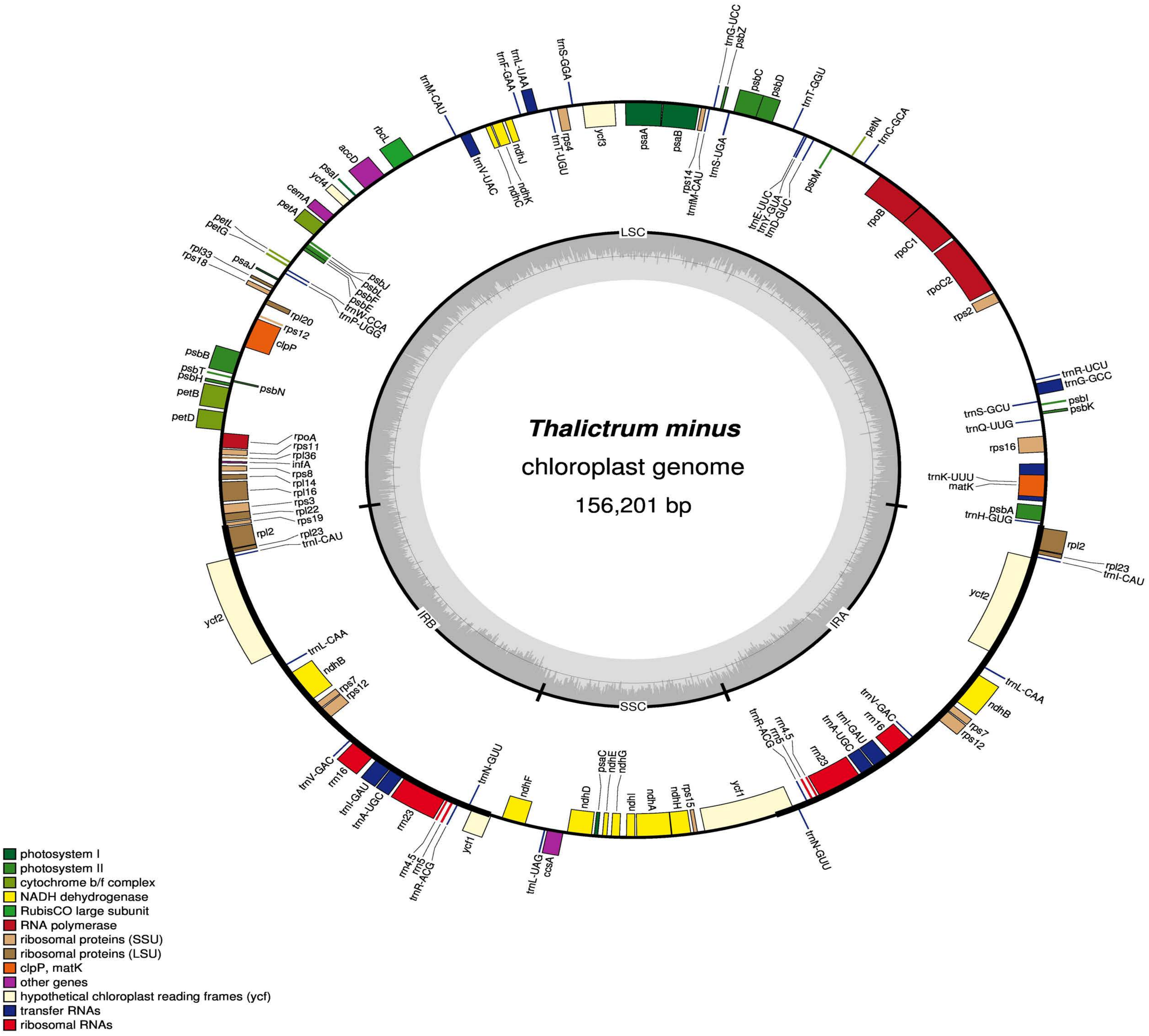

Supplementary Figure S1 (continue)

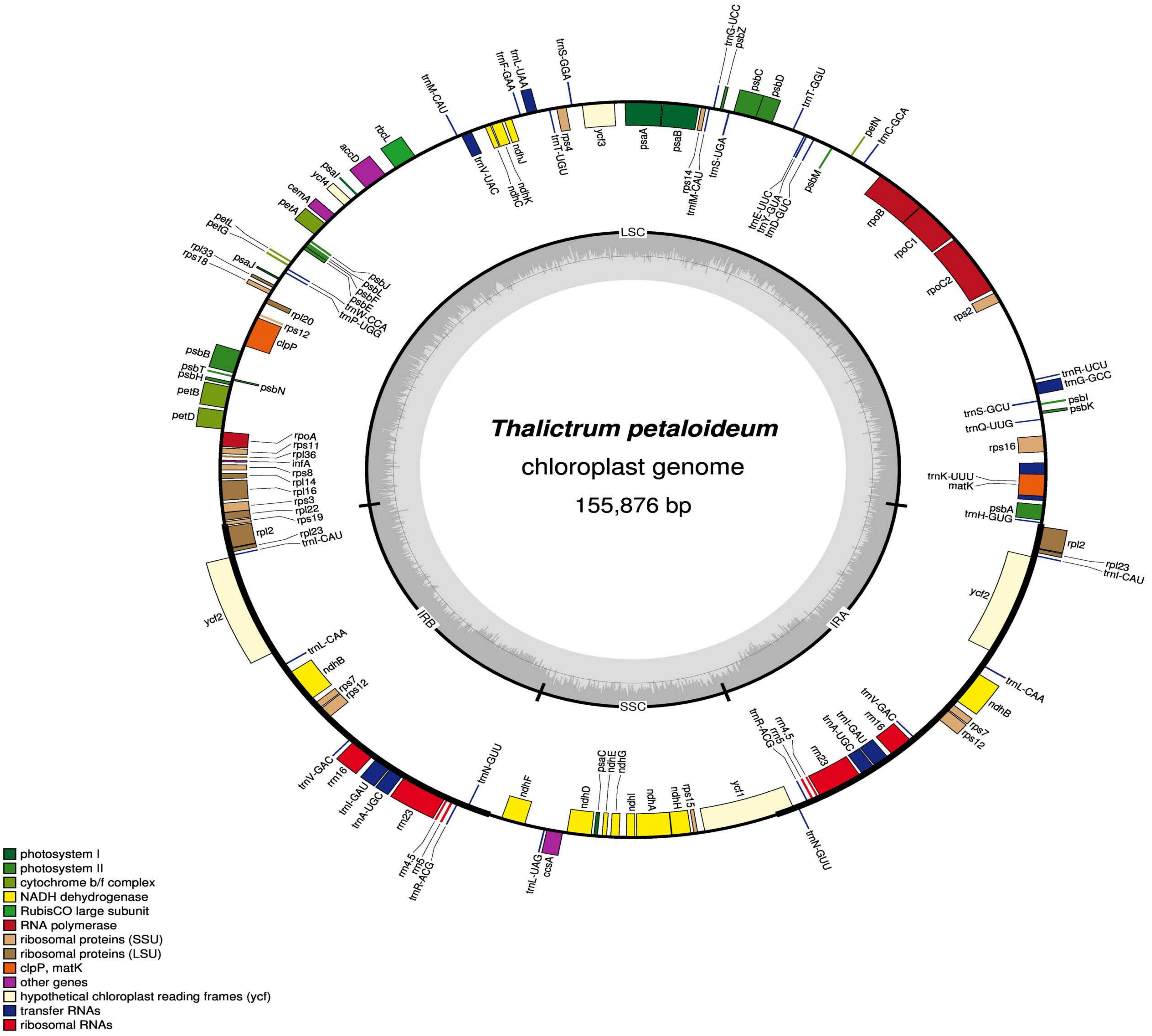

Supplementary Figure S1 (continue)

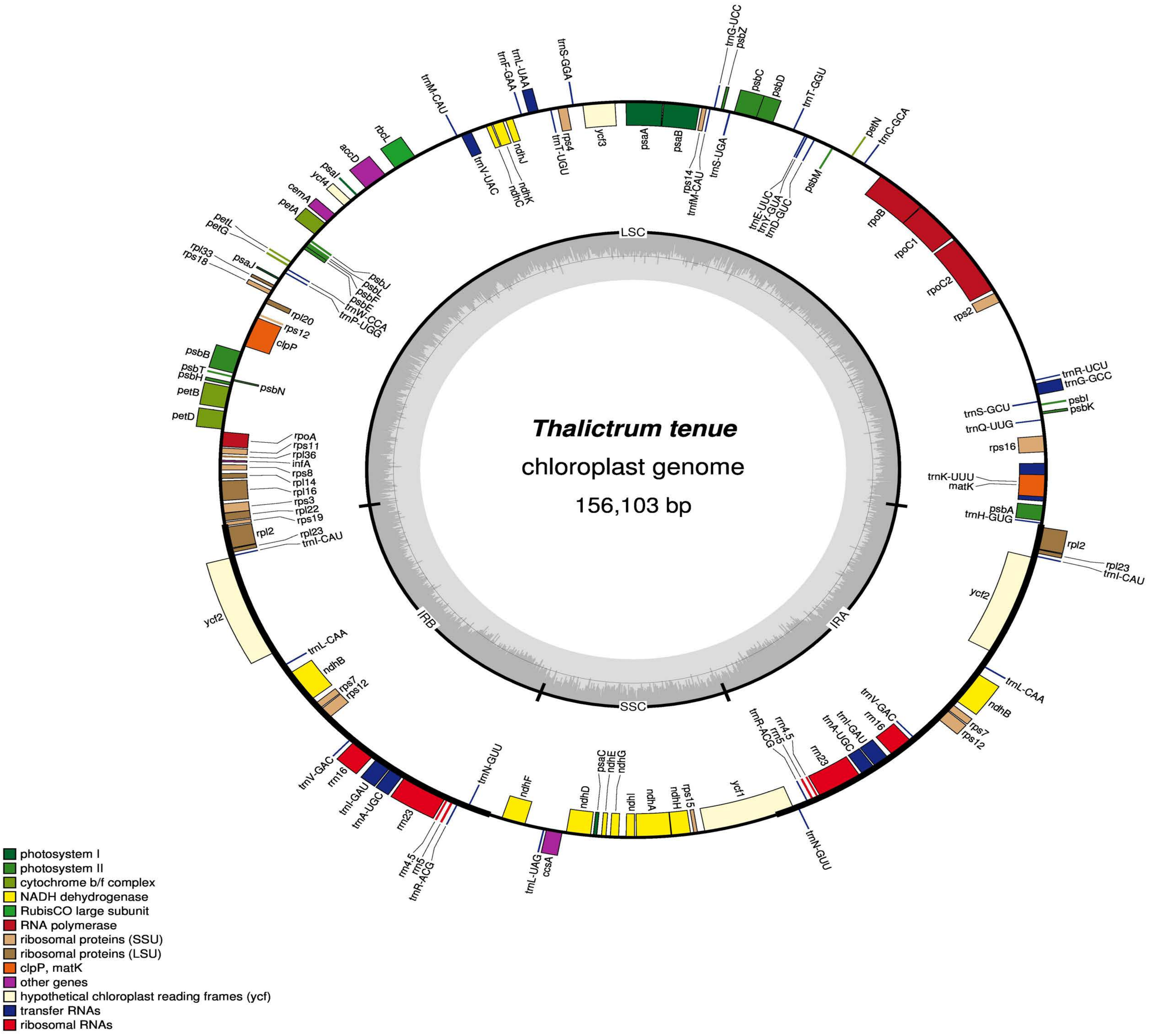

Supplementary Figure S1 (continue)

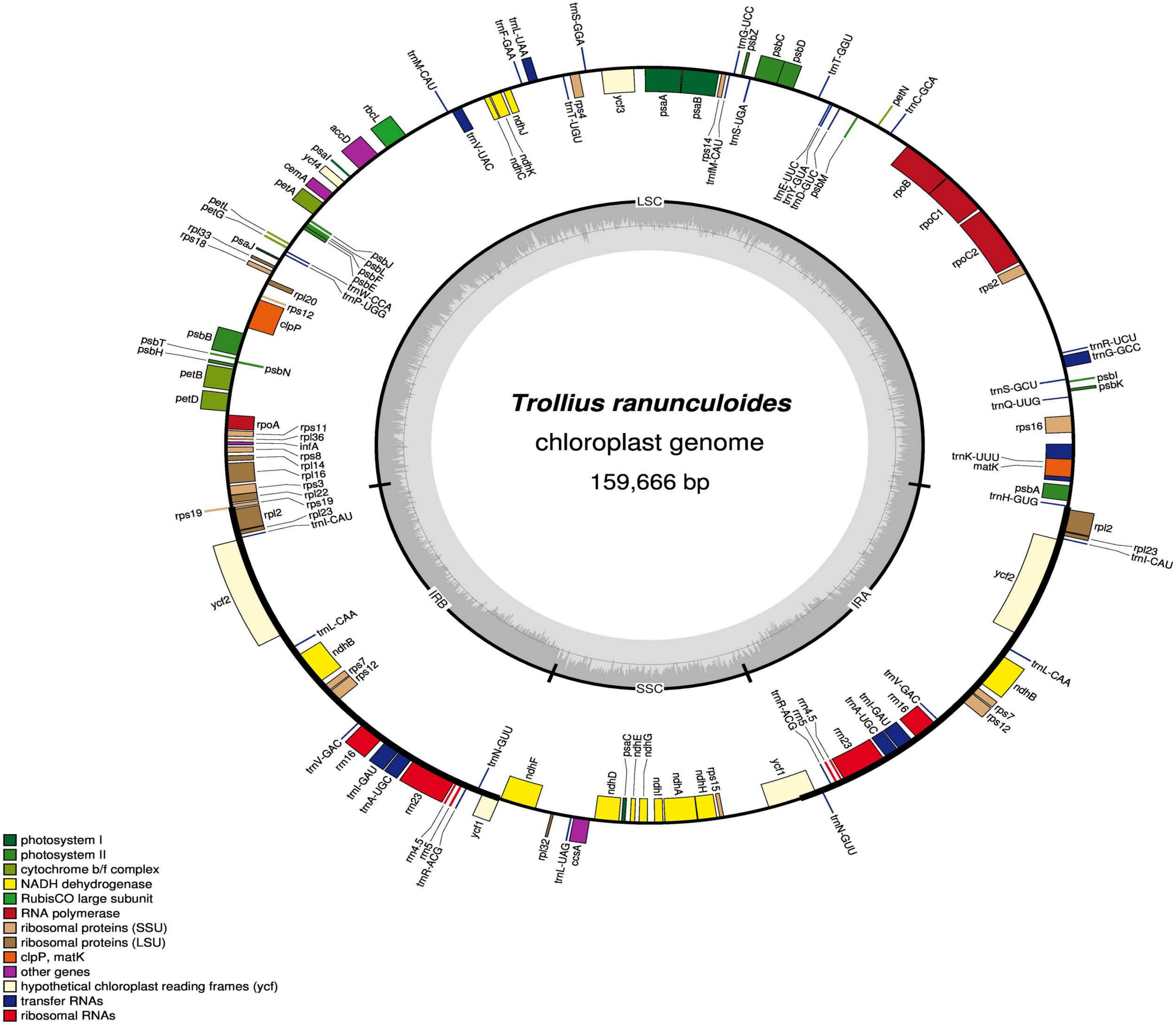

Supplementary Figure S1 (continue)

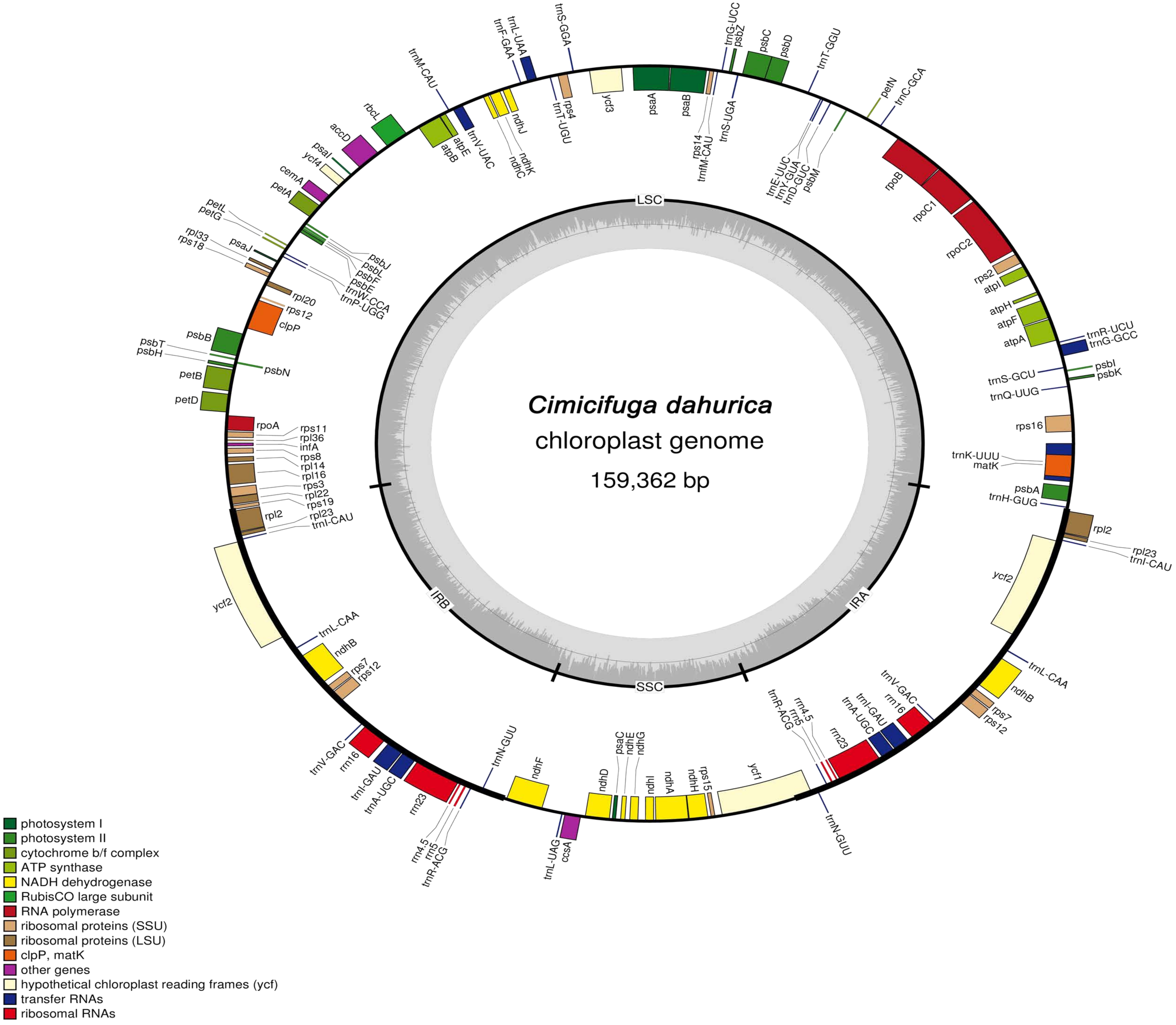

Supplementary Figure S1 (continue)

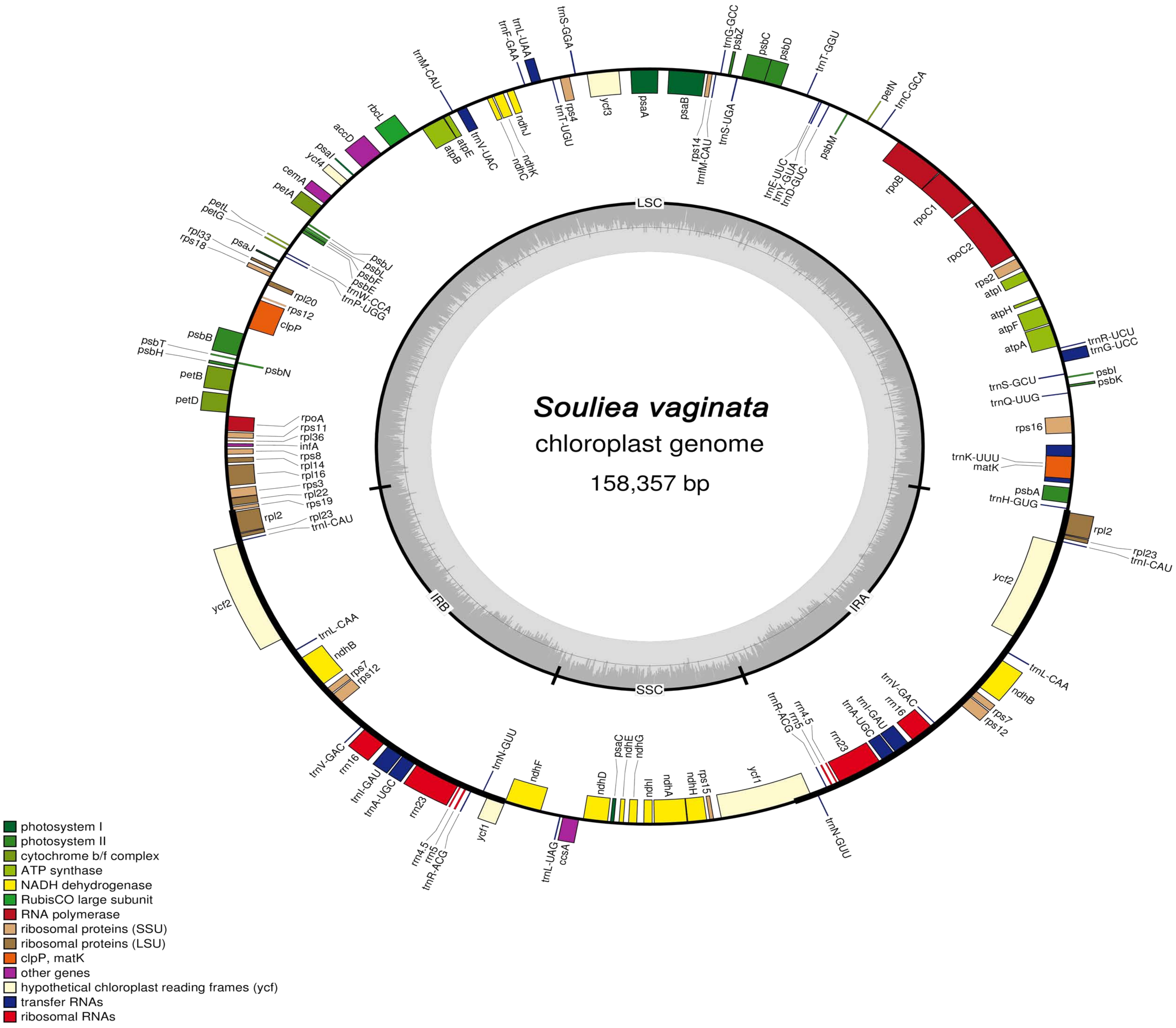

Supplementary Figure S1 (continue)

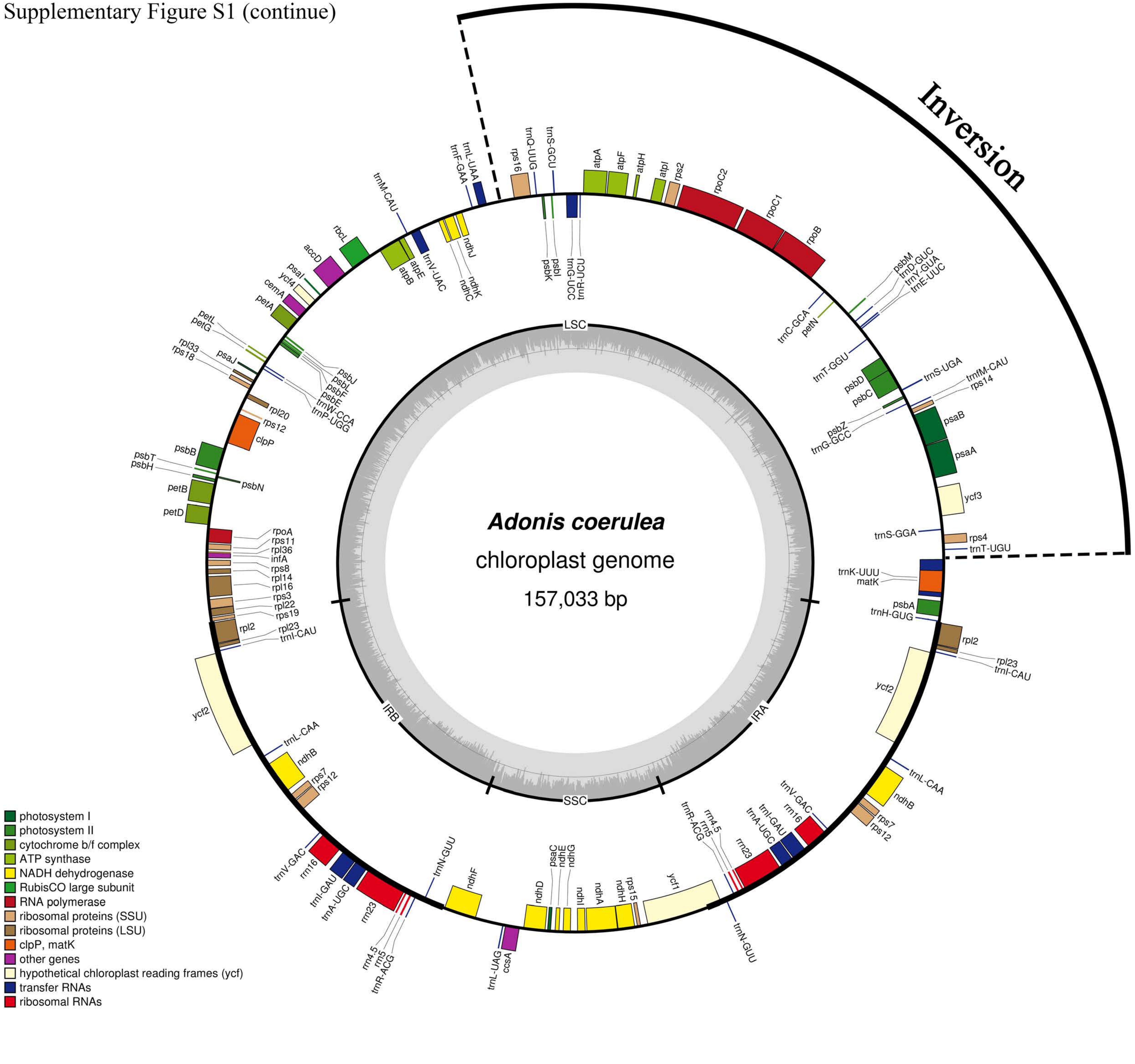

Supplementary Figure S1 (continue)

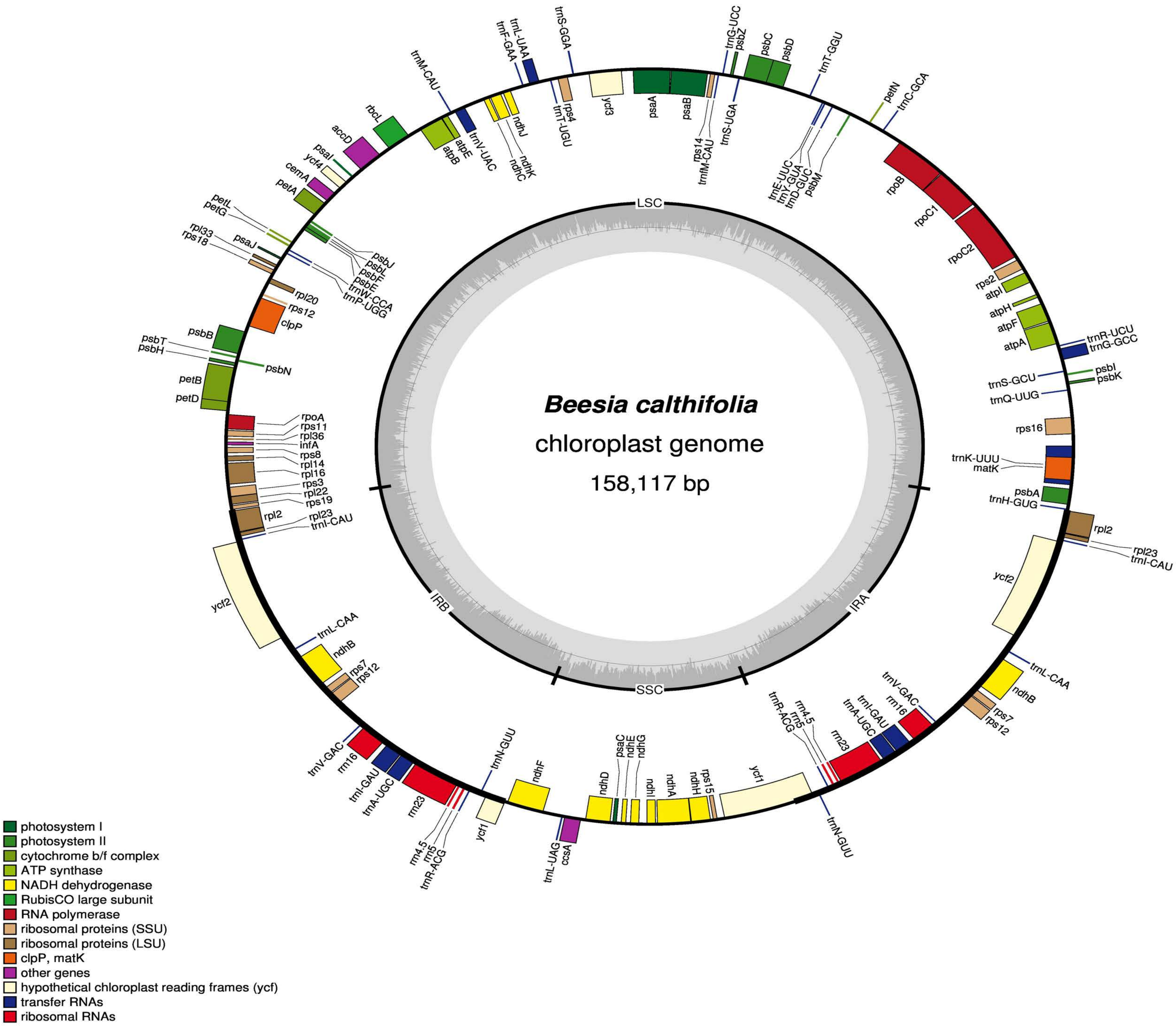

Supplementary Figure S1 (continue)

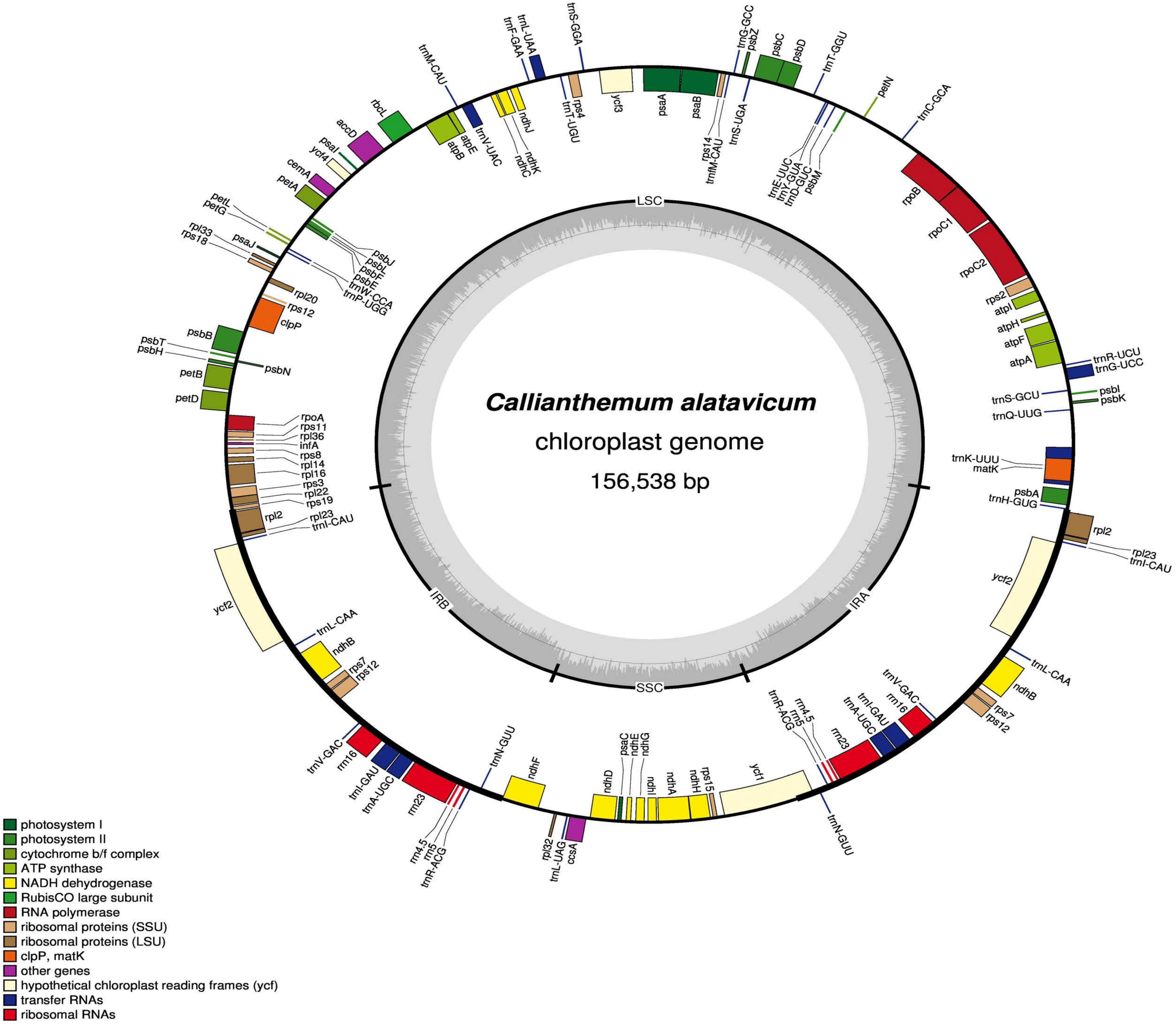

Supplementary Figure S1 (continue)

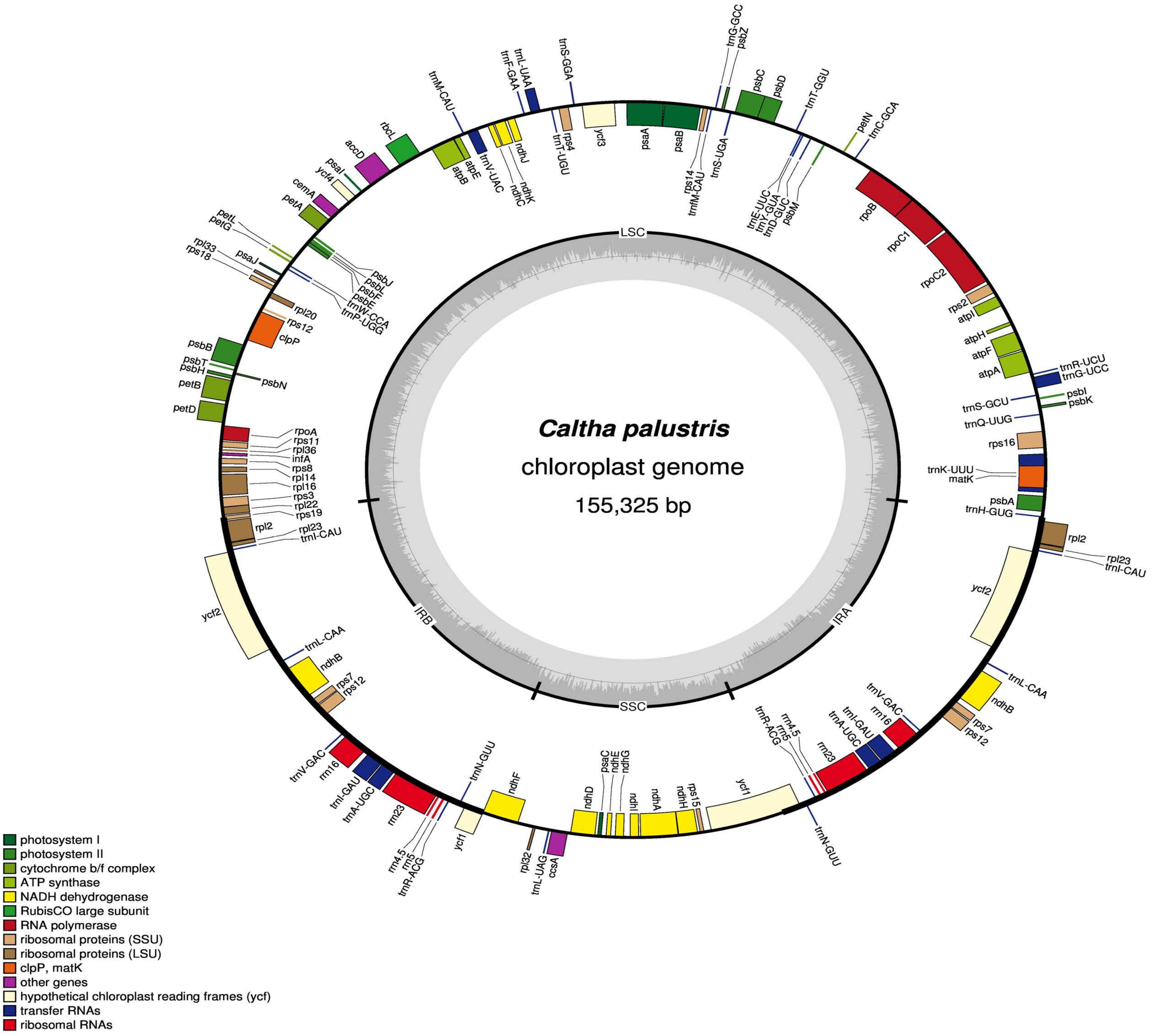

Supplementary Figure S1 (continue)

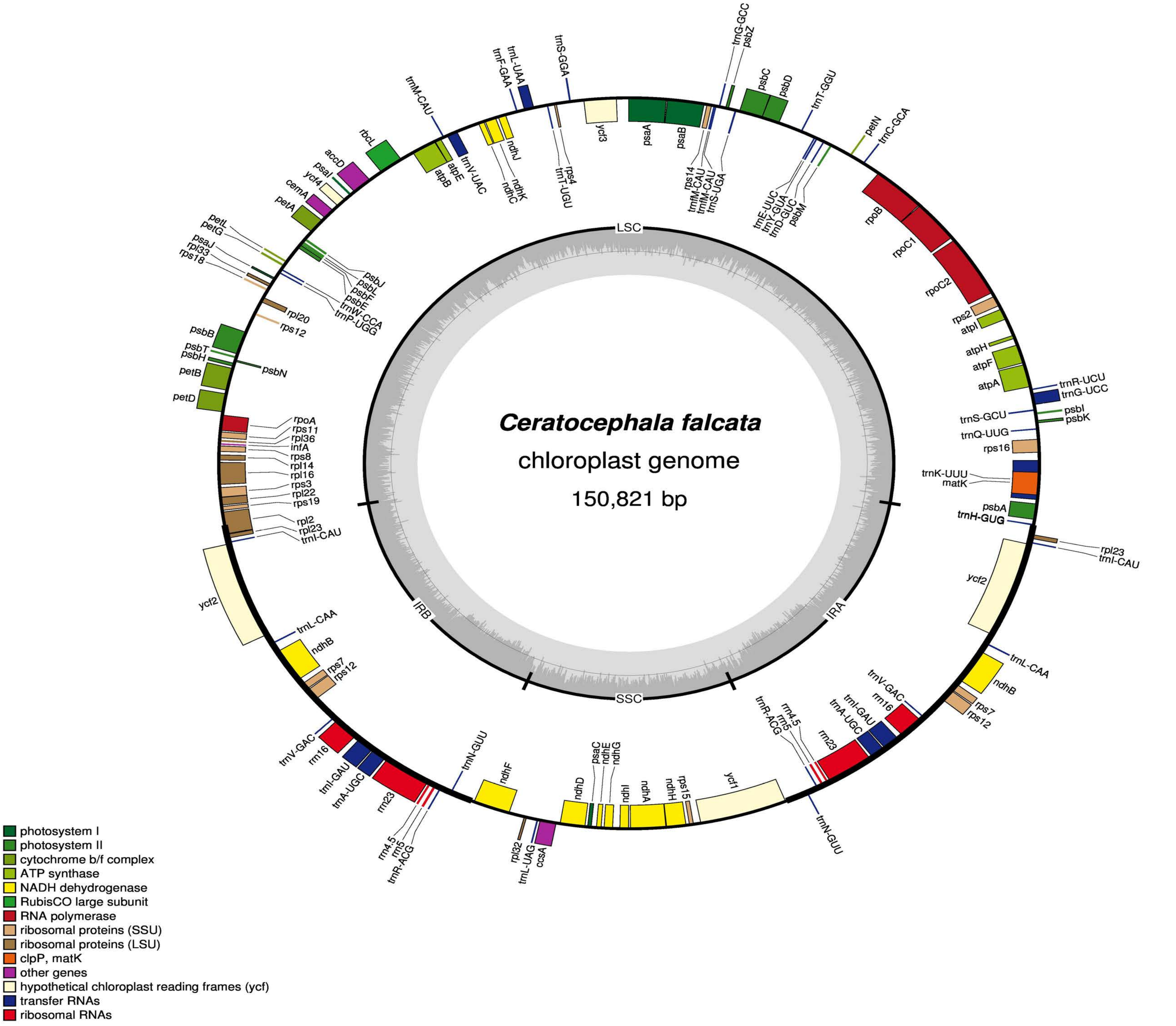

Supplementary Figure S2

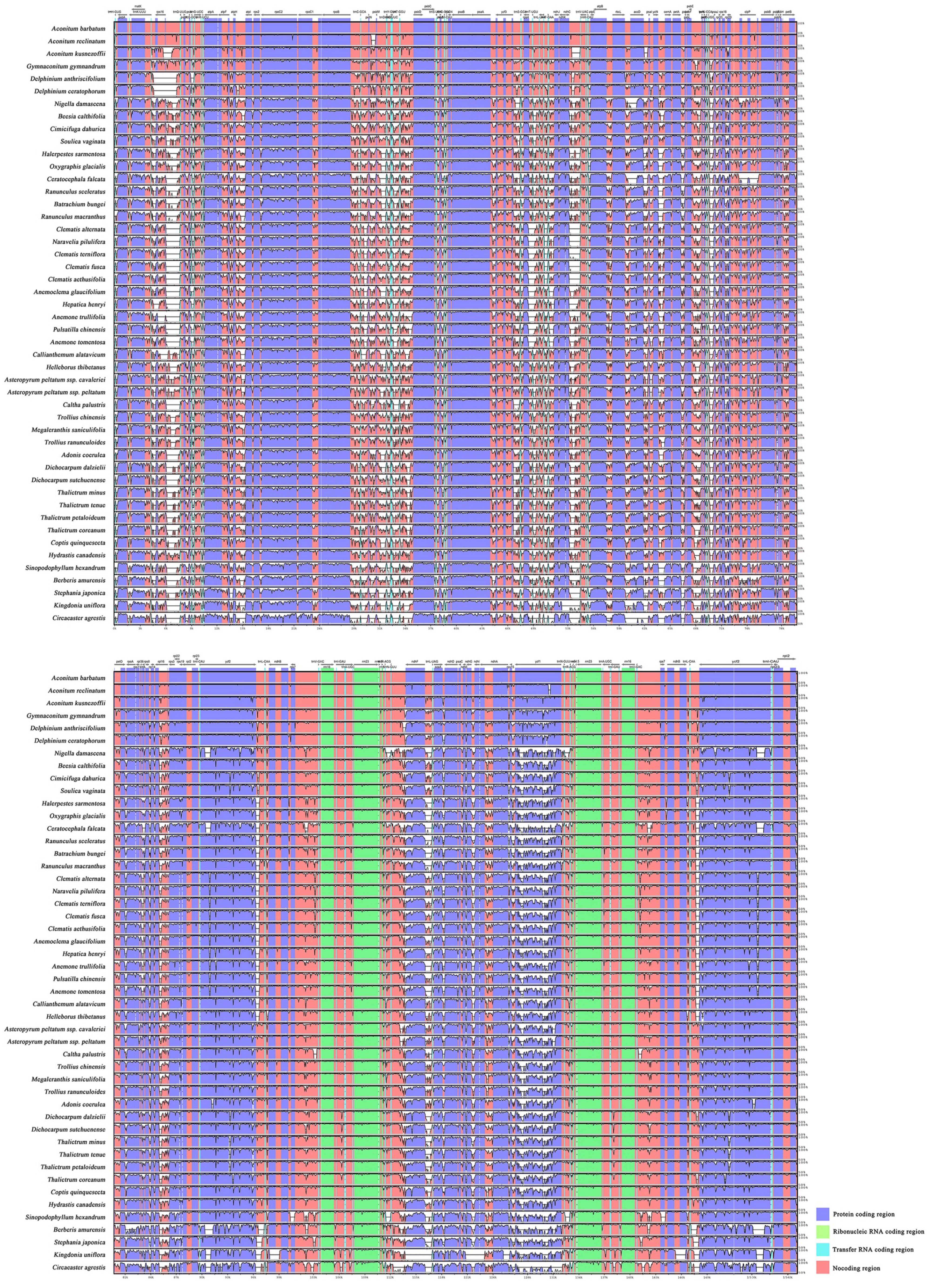

Supplementary Figure S3

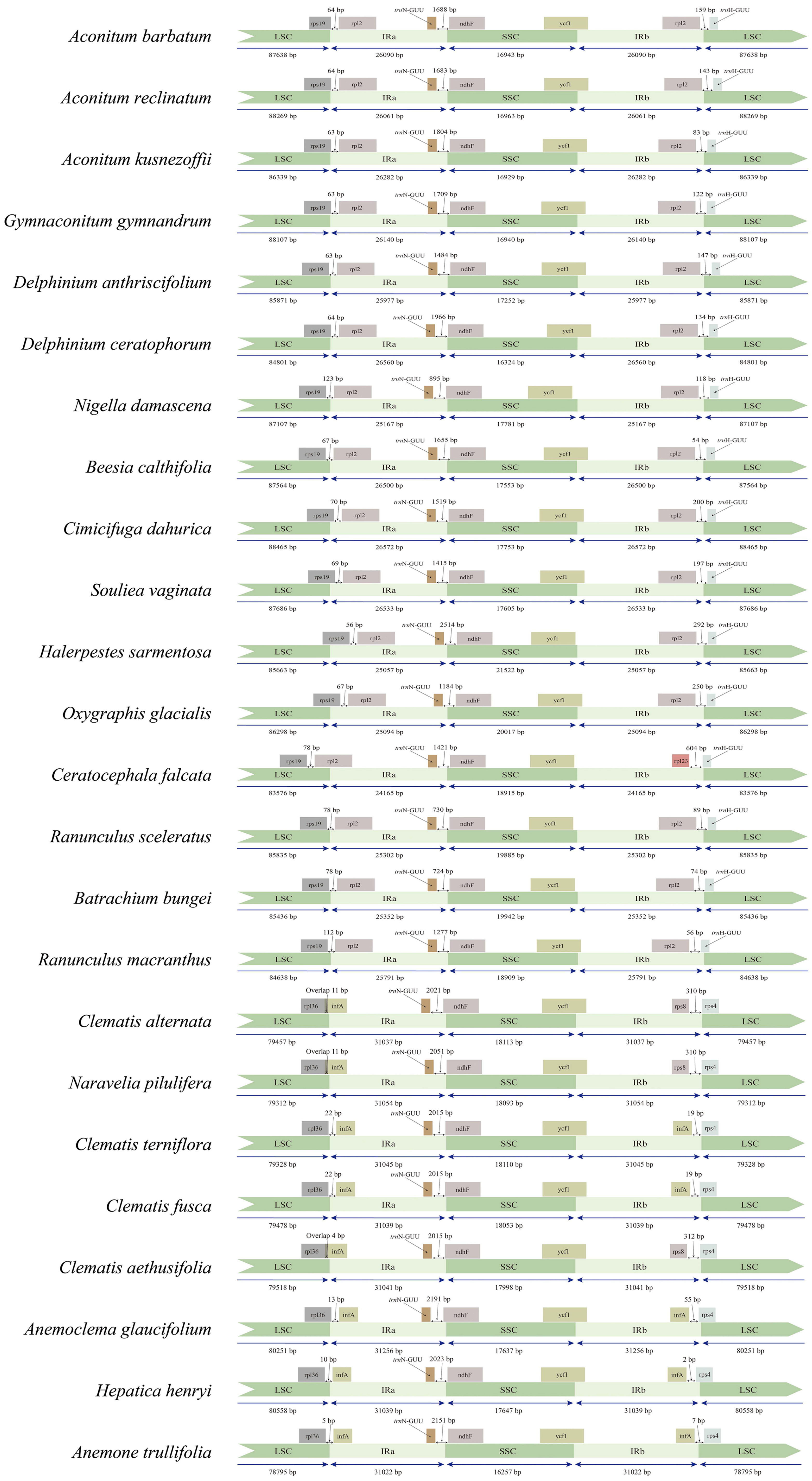

Supplementary Figure S3 (continue)

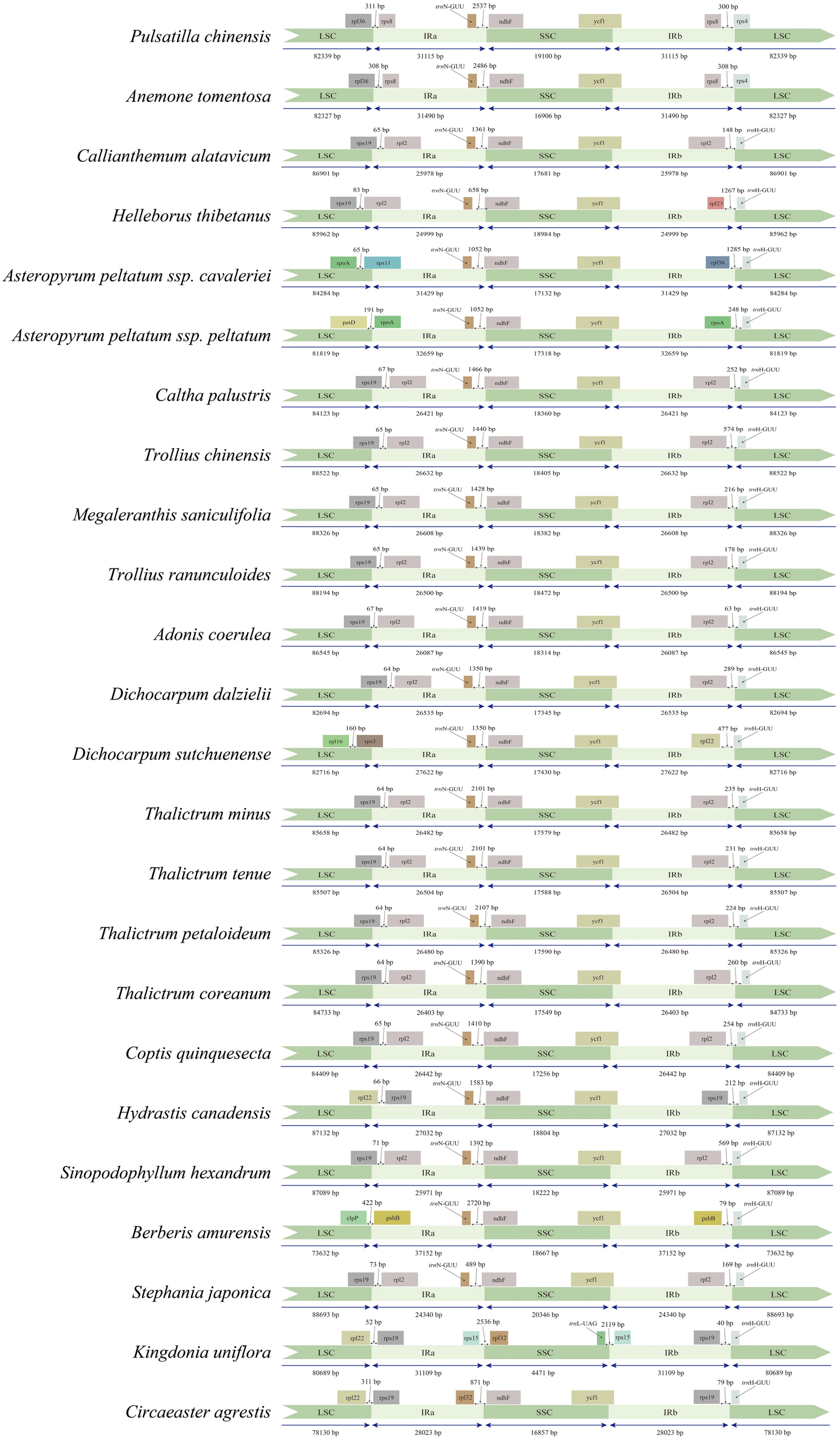

Supplementary Figure S4

Complete cp genome

Mrbayes

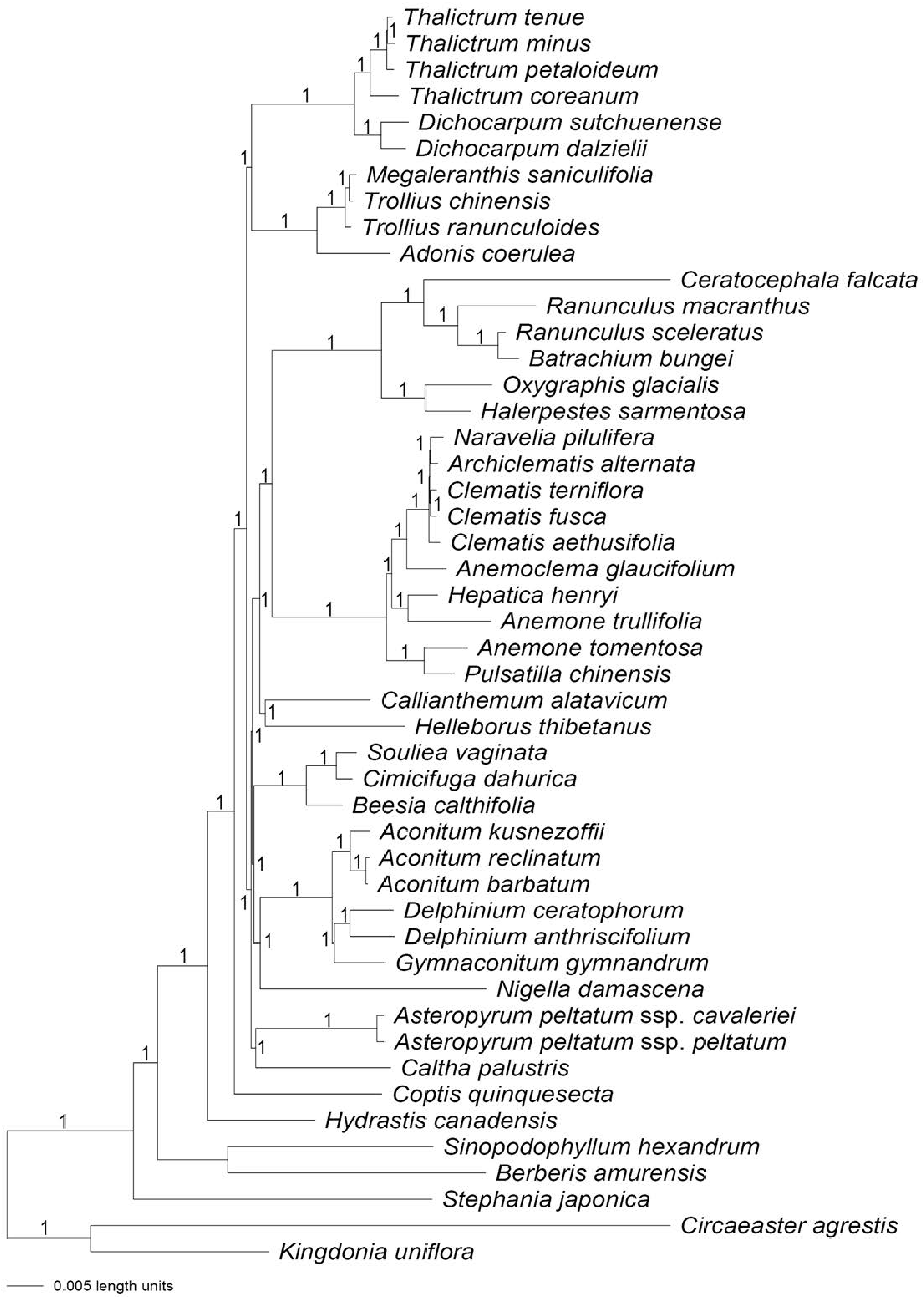

Supplementary Figure S4 (continue)

Cp CDs

Mrbayes

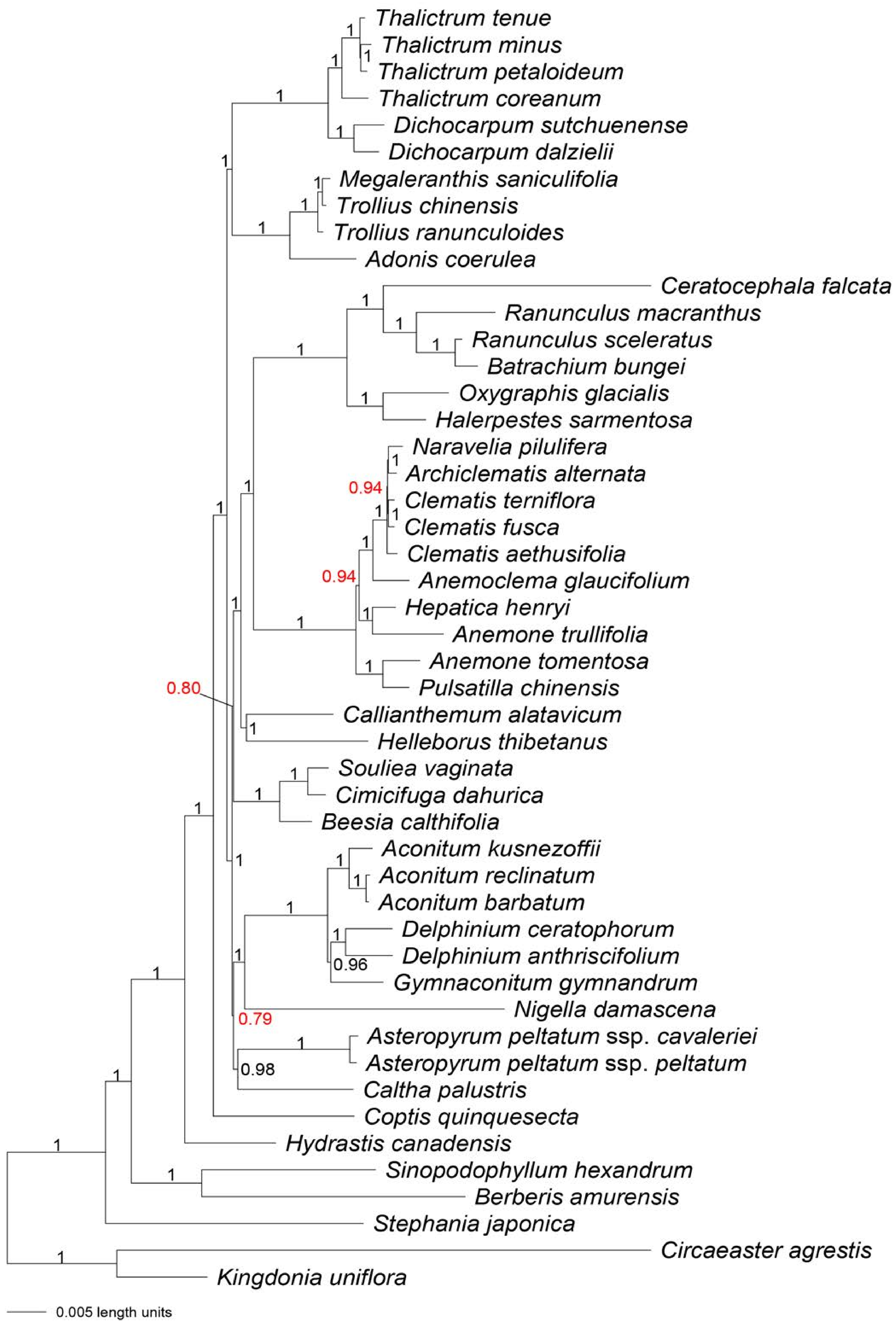

Supplementary Figure S4 (continue)

Cp IGS

Mrbayes

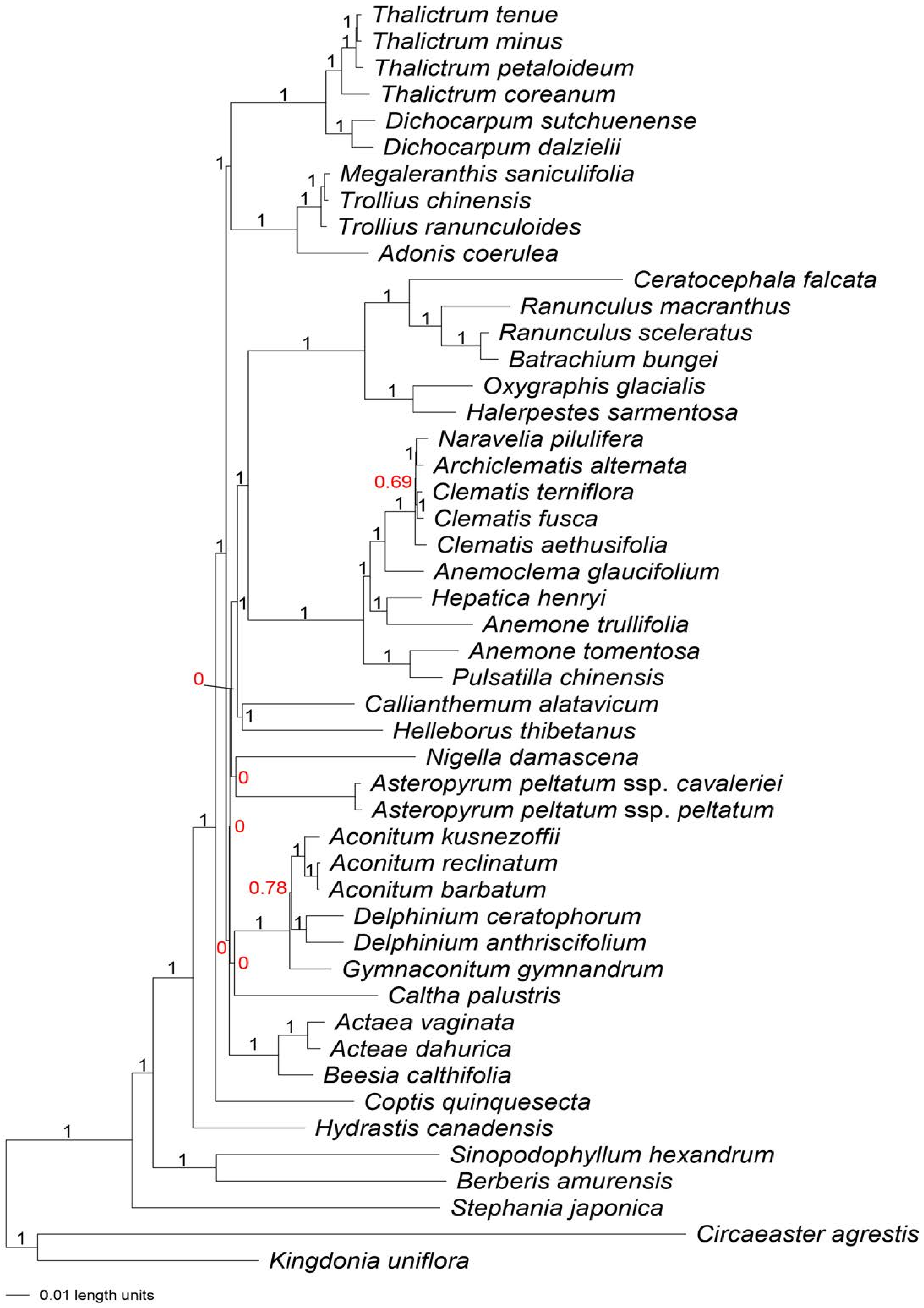

Supplementary Figure S4 (continue)

Cp Intron

Mrbayes

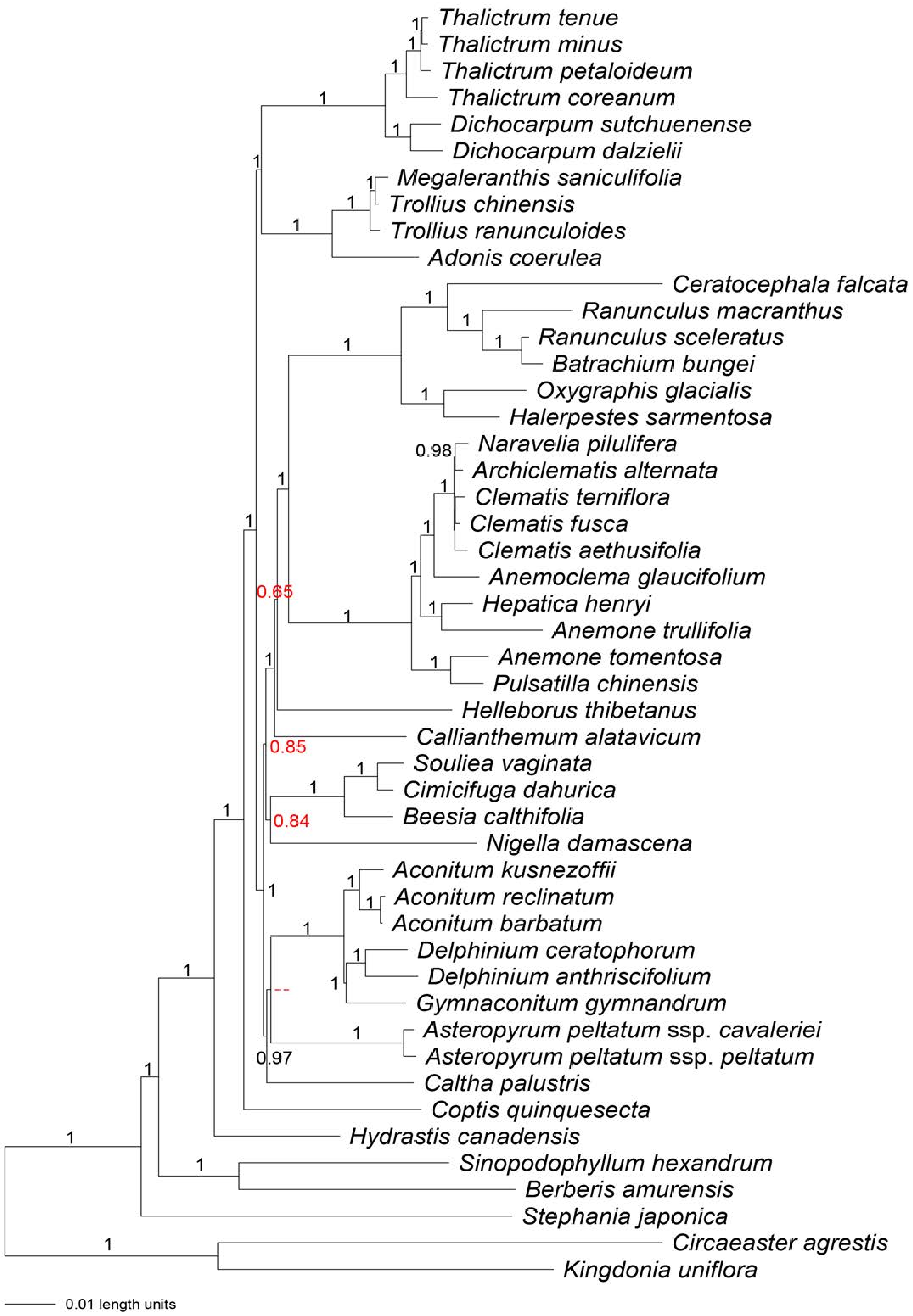

Supplementary Figure S4 (continue)

Cp LSC

Mrbayes

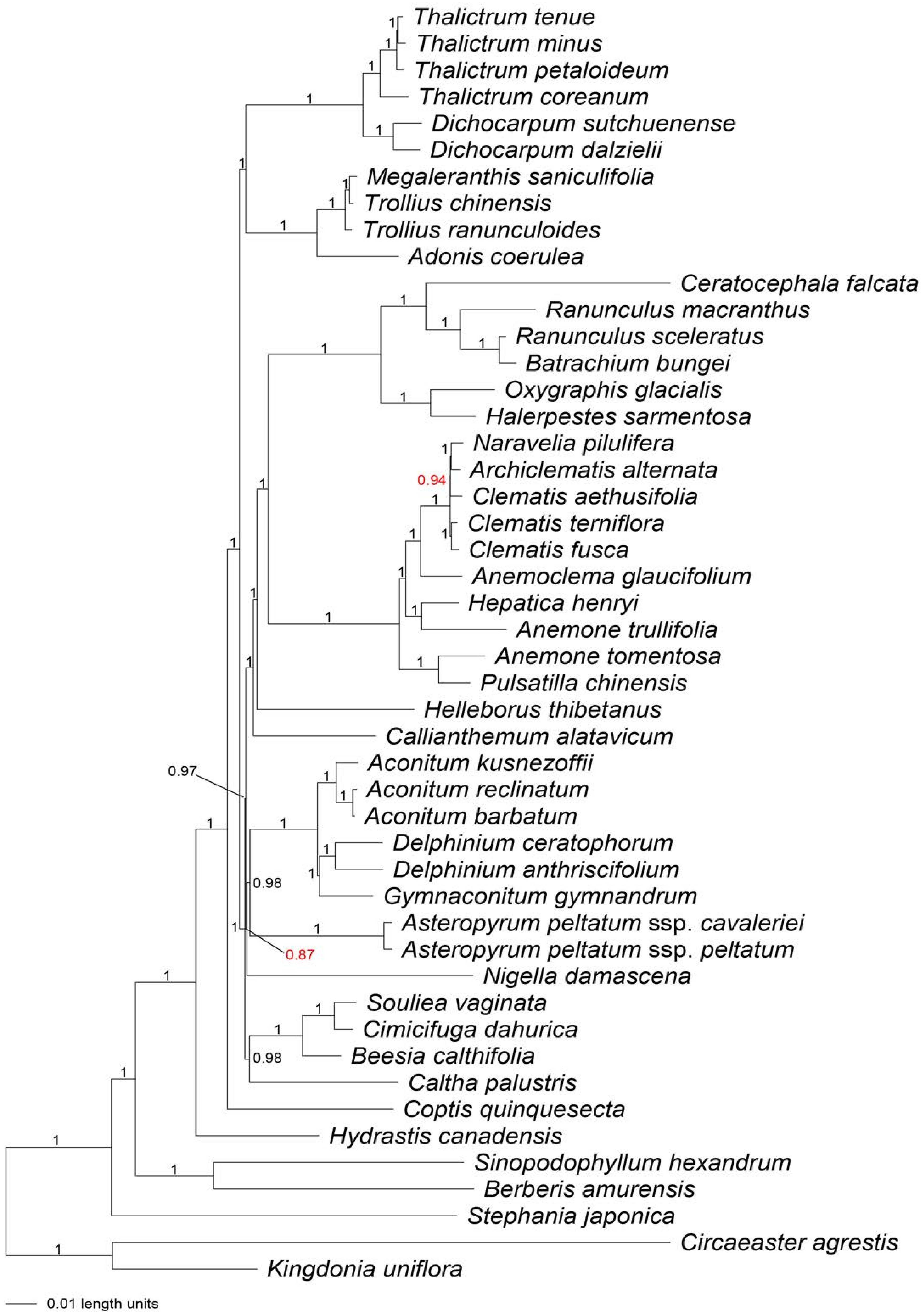

Supplementary Figure S4 (continue)

Cp SSC

Mrbayes

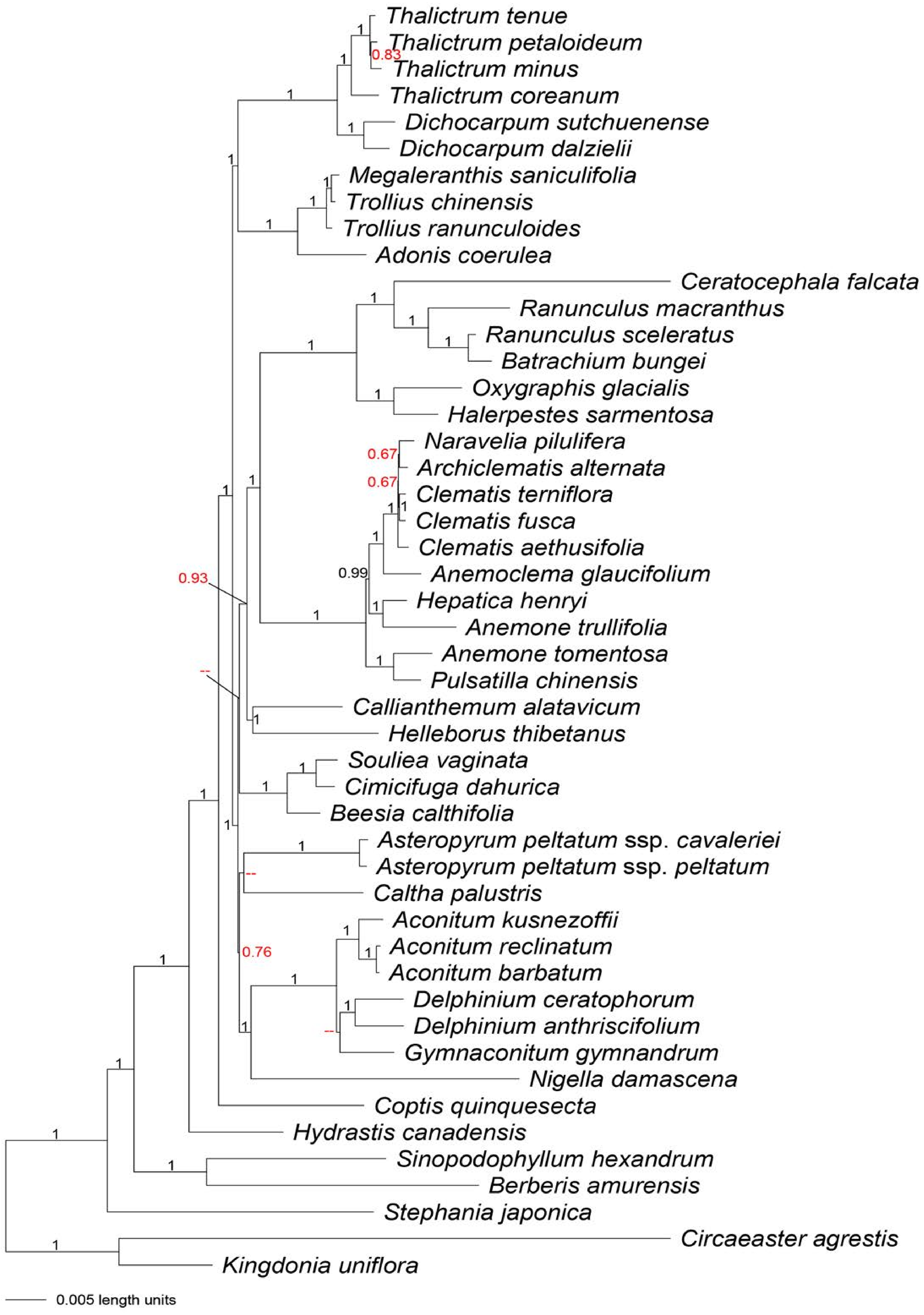

Supplementary Figure S4 (continue)

Cp IR

Mrbayes

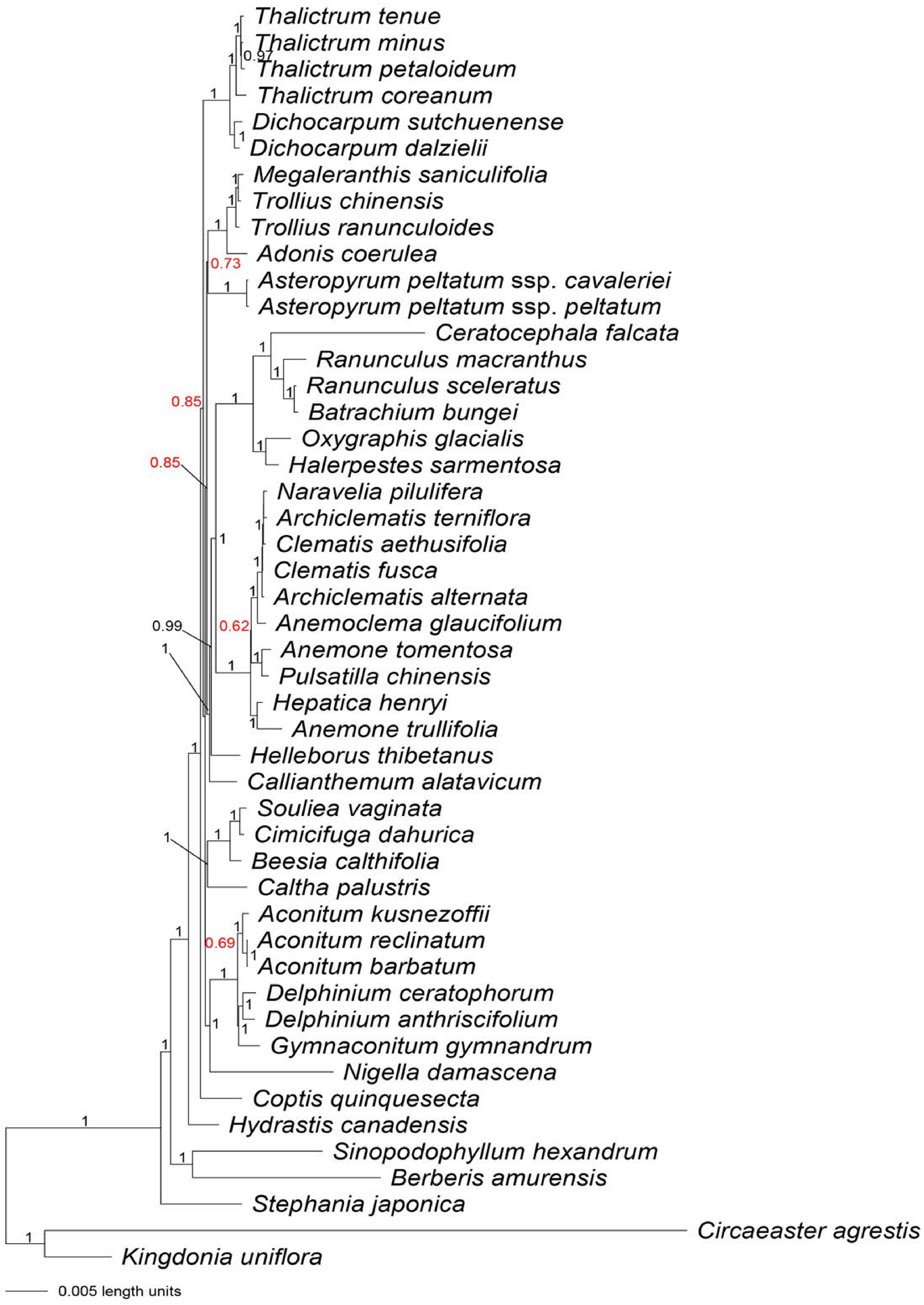

Supplementary Figure S4 (continue)

Complete cp genome

Raxml

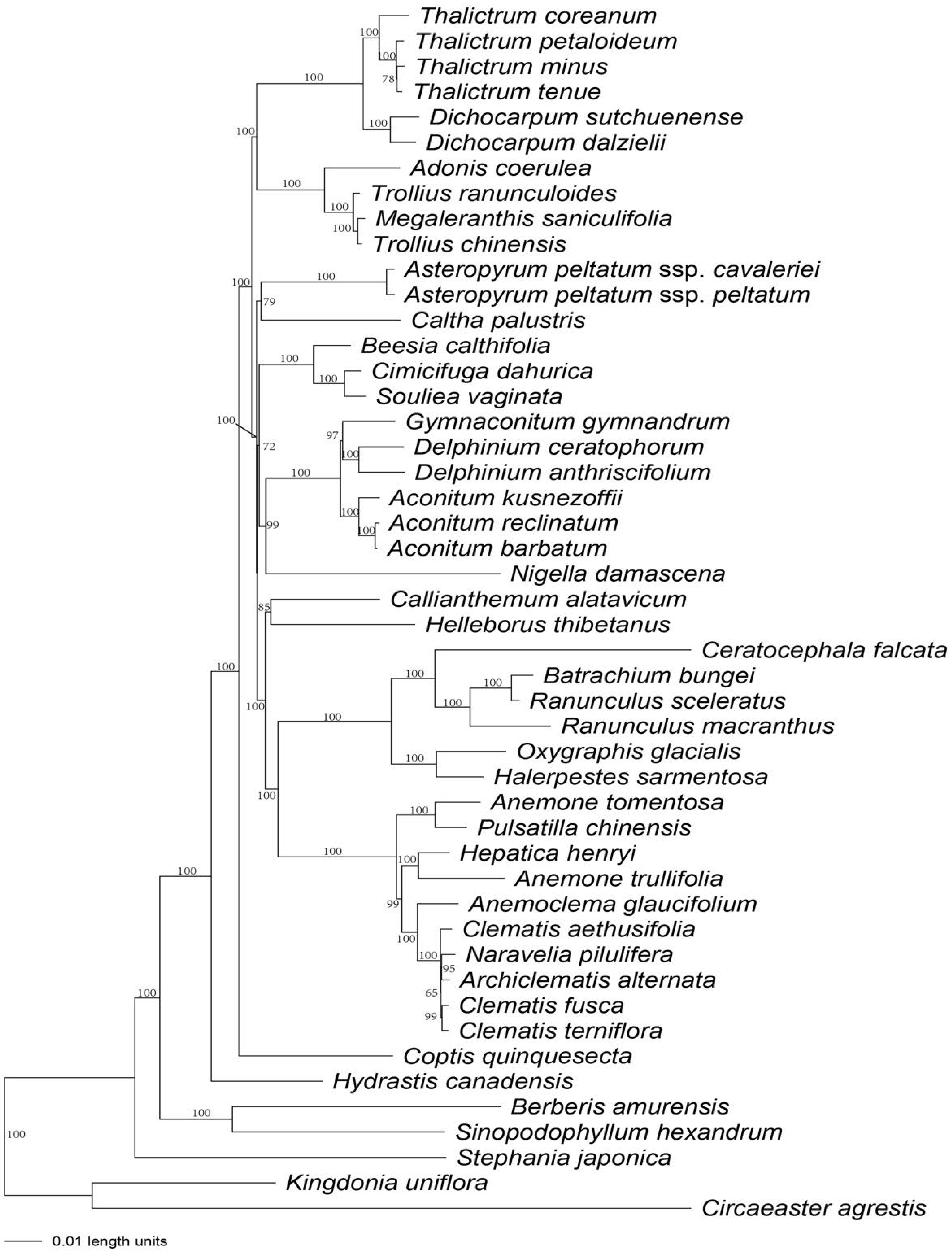

Supplementary Figure S4 (continue)

Cp CDs

Raxml

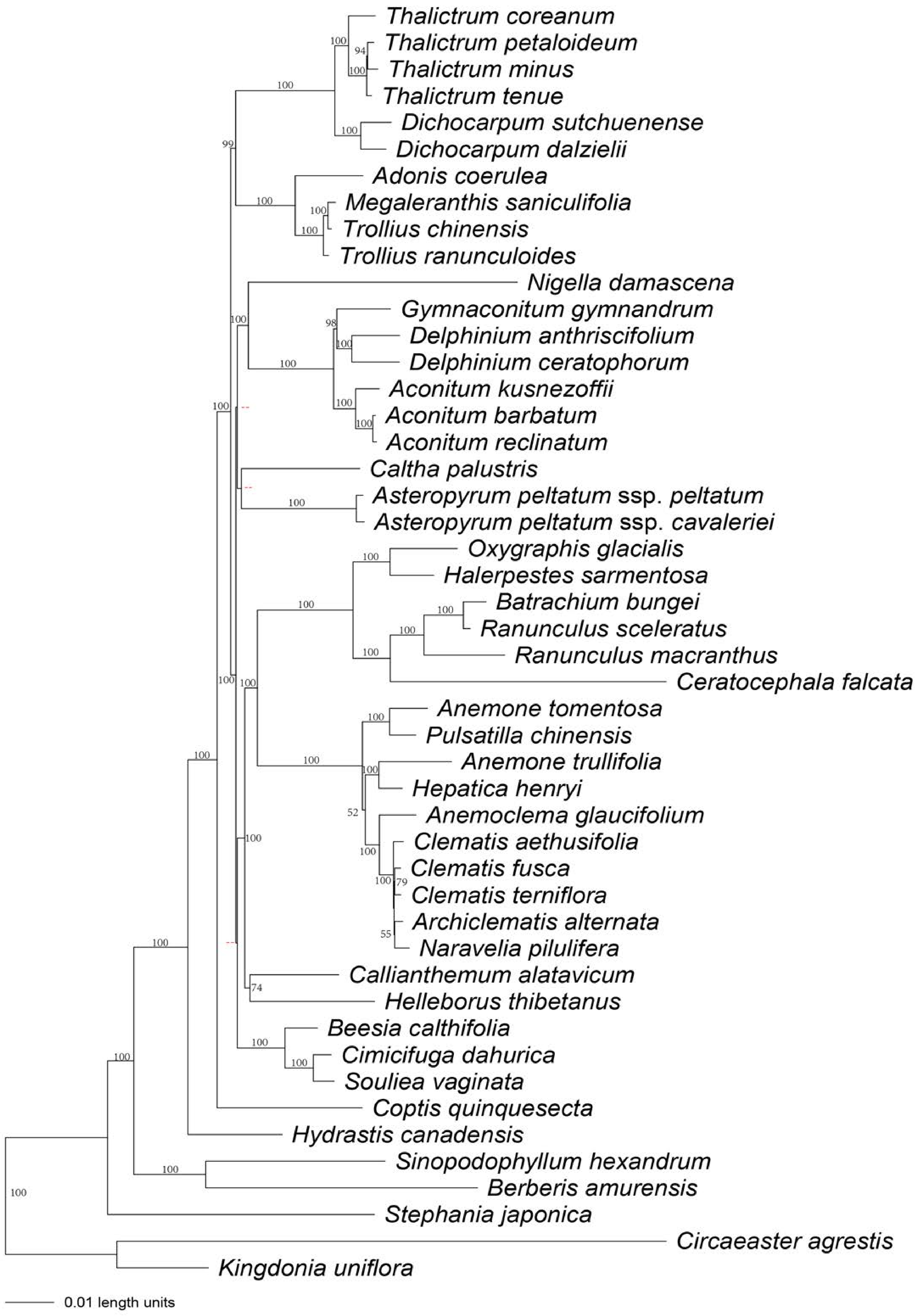

Supplementary Figure S4 (continue)

Cp IGS

Raxml

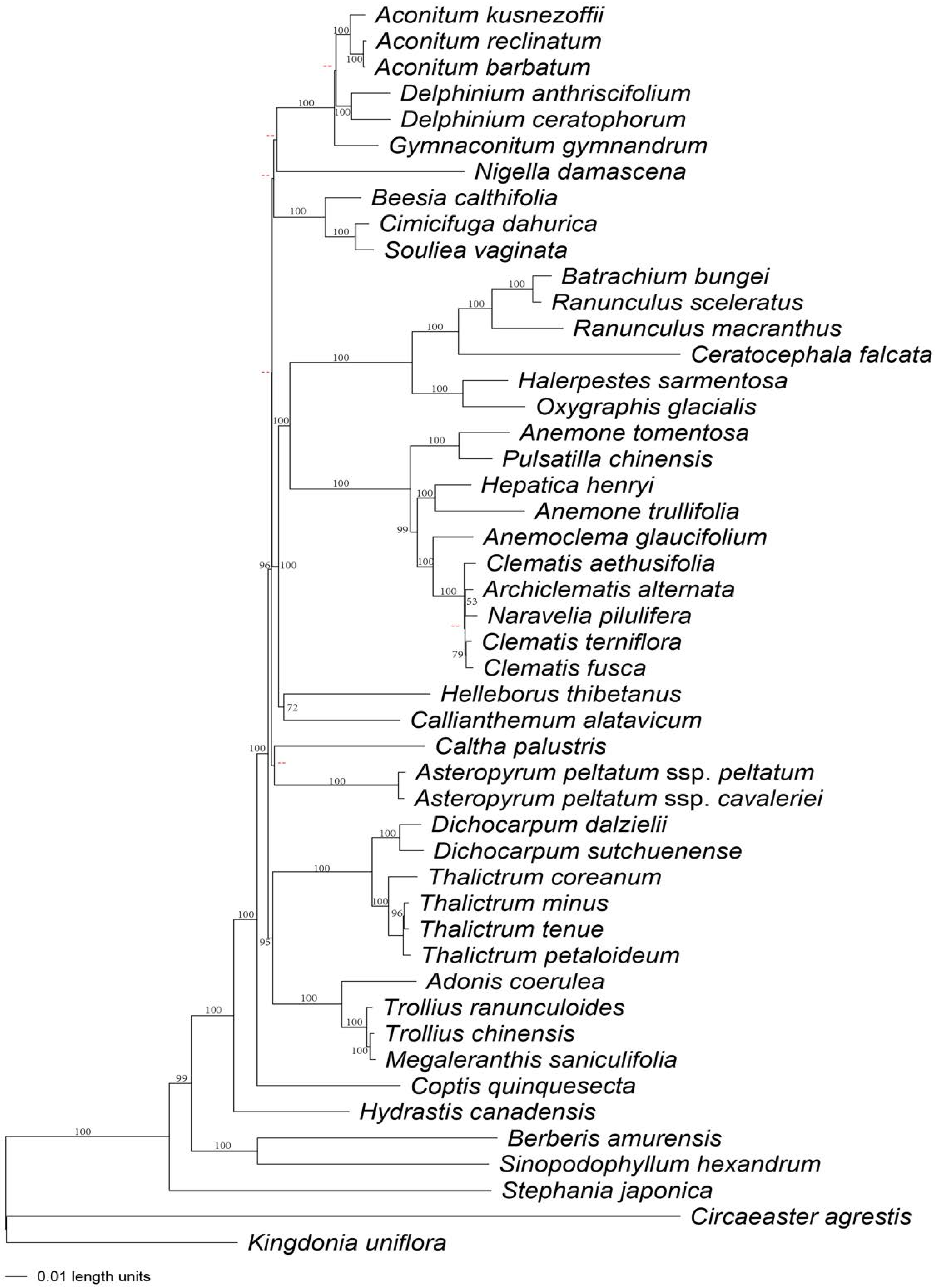

Supplementary Figure S4 (continue)

Cp Intron

Raxml

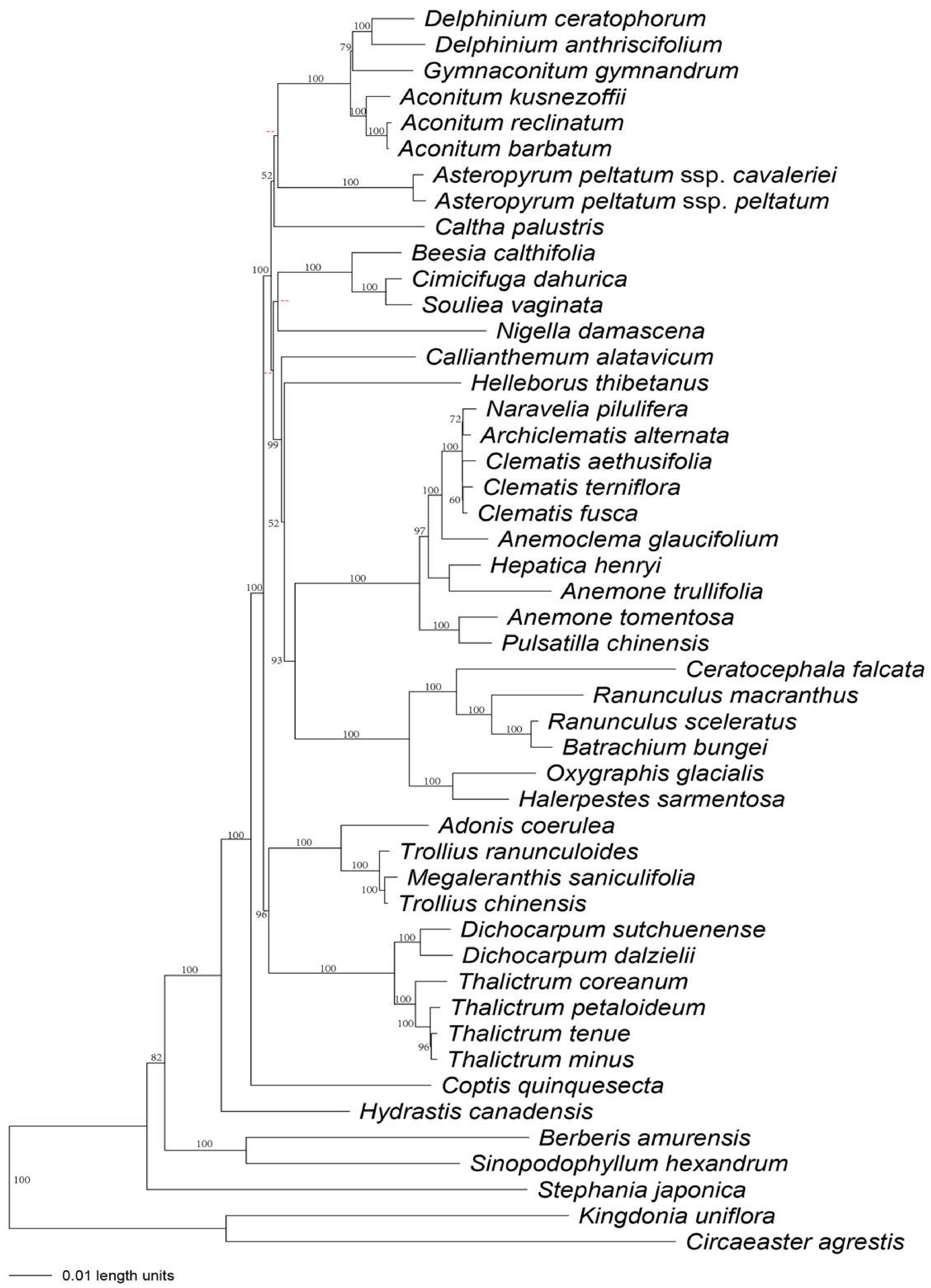

Supplementary Figure S4 (continue)

Cp LSC

Raxml

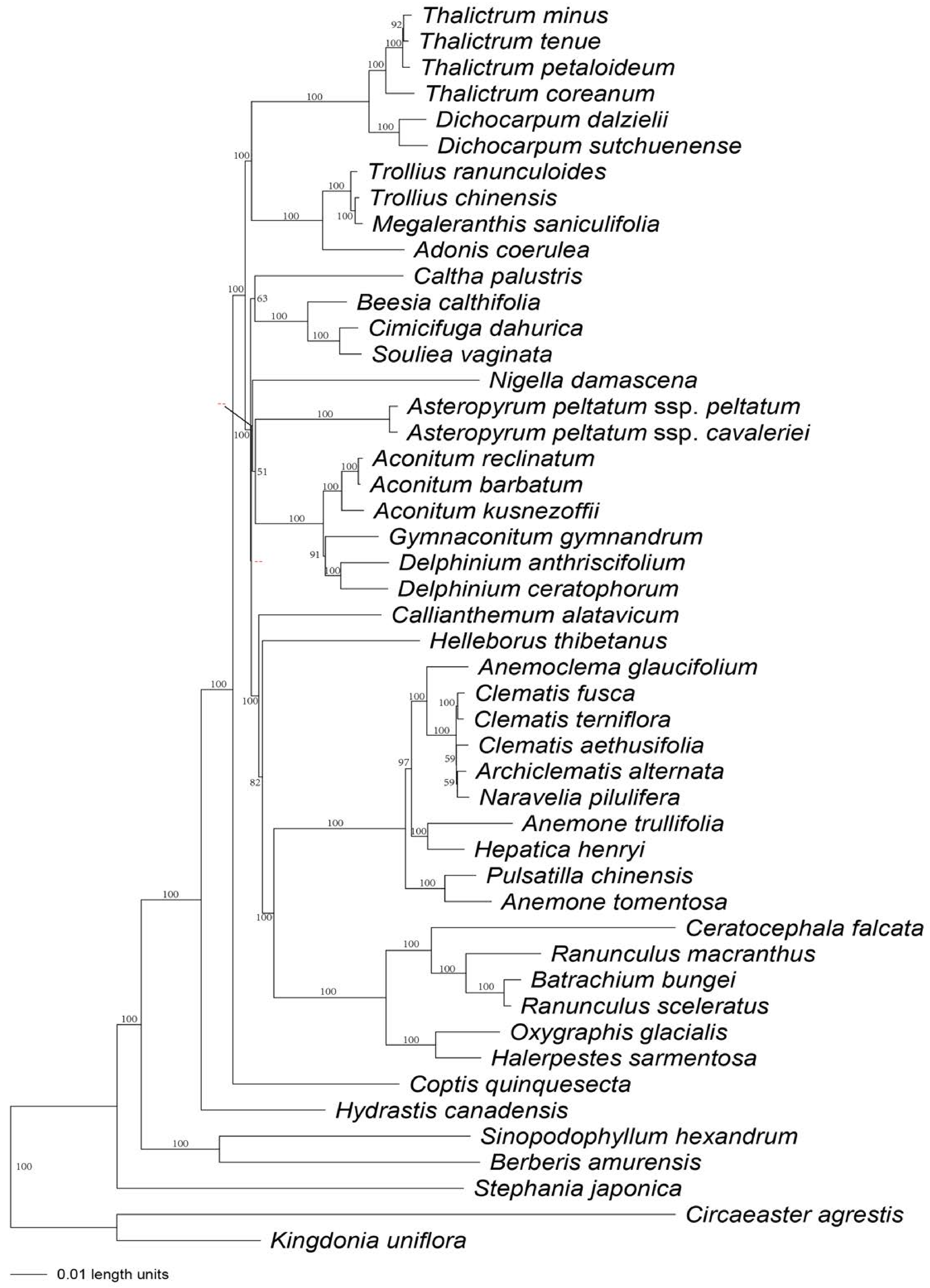

Supplementary Figure S4 (continue)

Cp SSC

Raxml

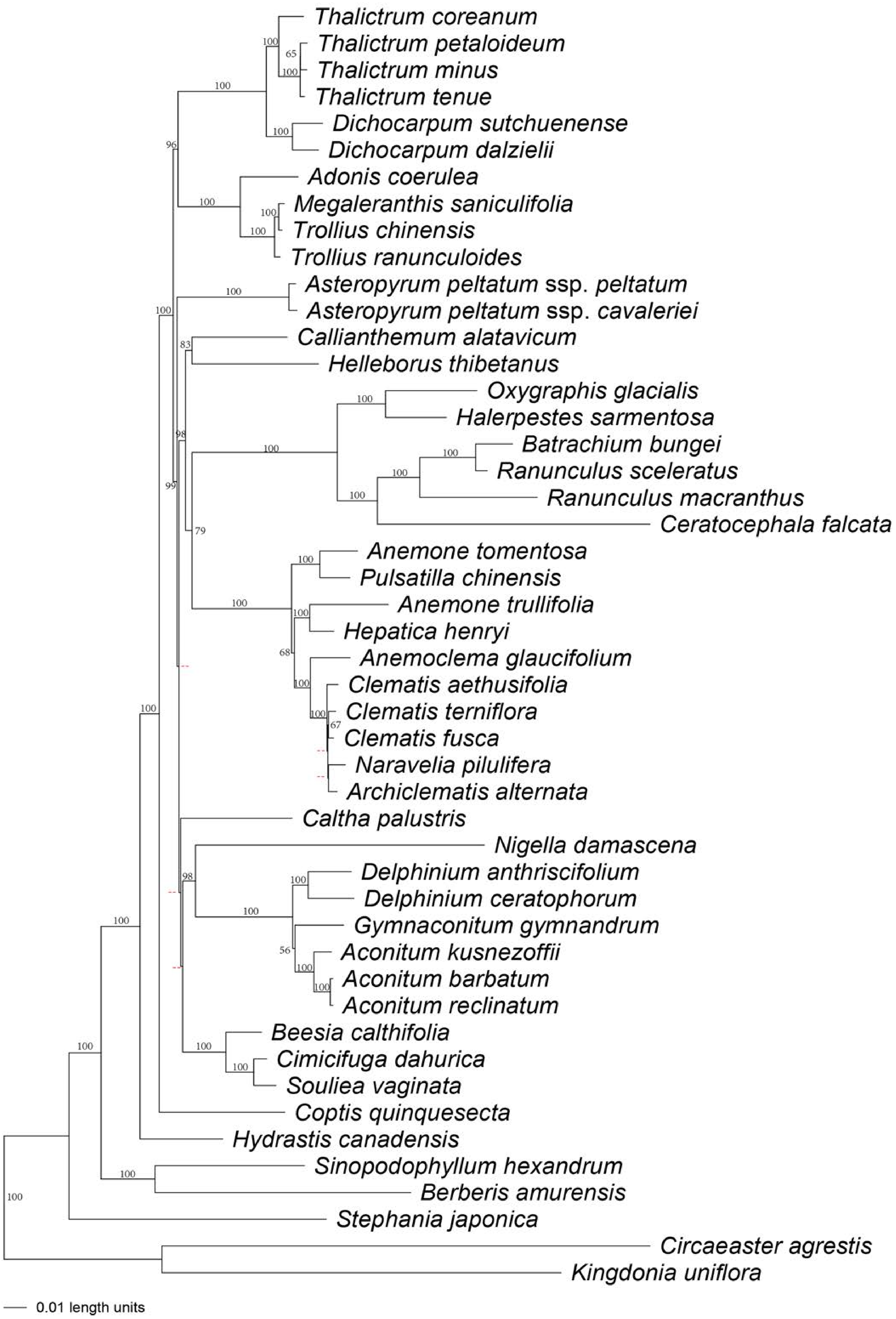

Supplementary Figure S4 (continue)

Cp IR

Raxml

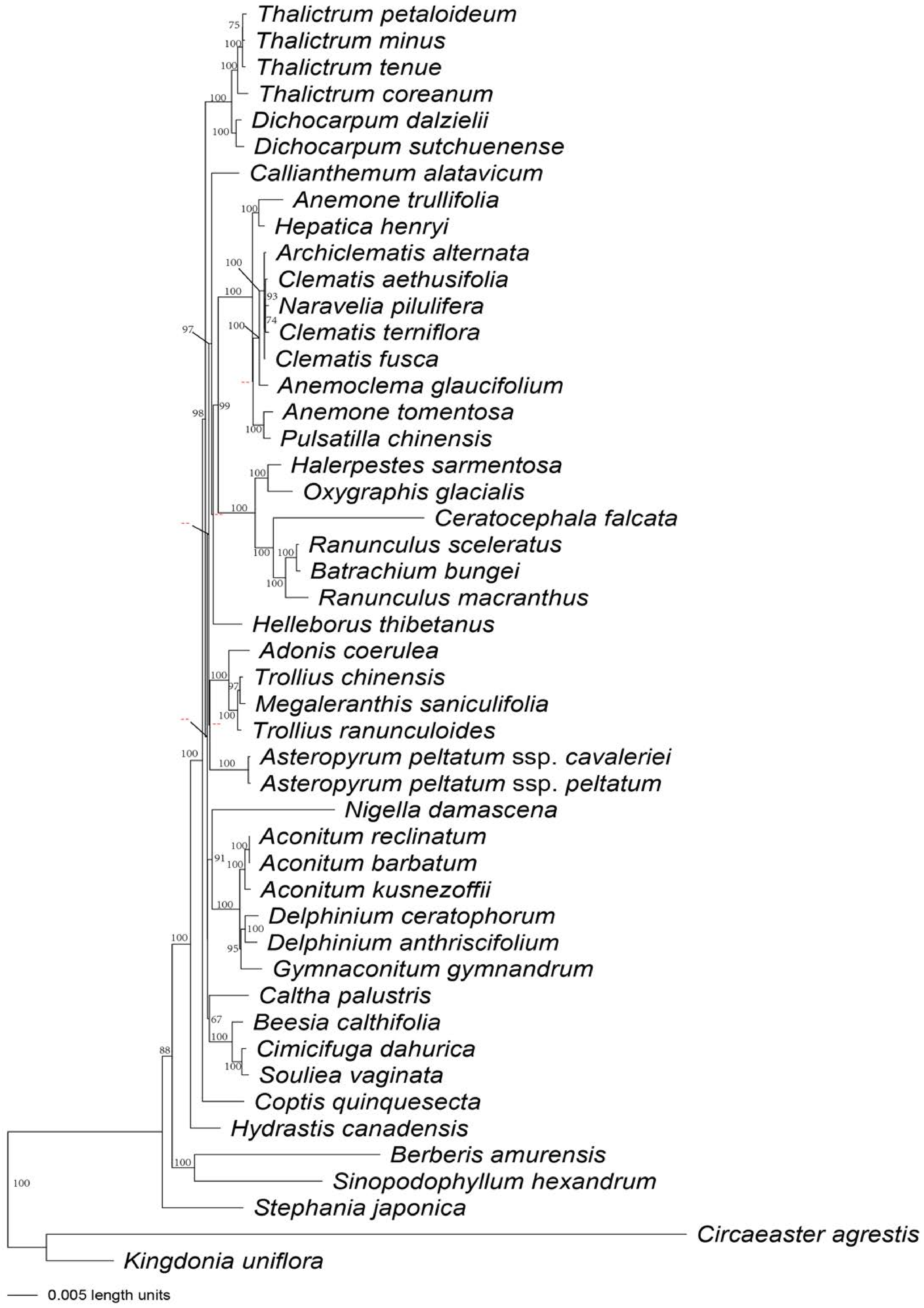

Supplementary Figure S4 (continue)

Complete cp genome

Paup-MP

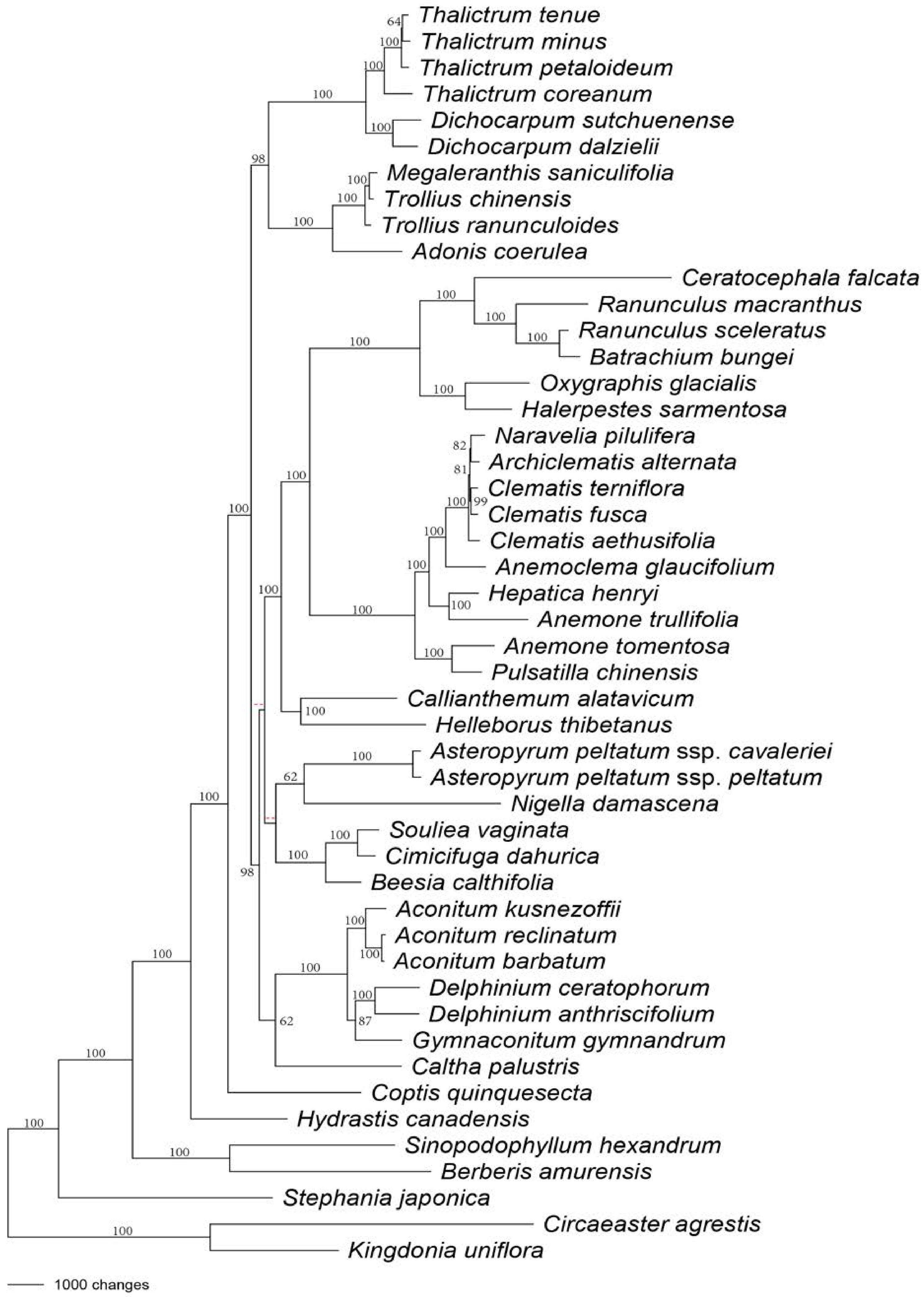

Of 120181 total characters:
All characters are of type 'unord'
All characters have equal weight
67209 characters are constant (proportion = 0.559231)
19708 variable characters are parsimony-uninformative
Number of parsimony-informative characters = 33264

Tree length = 132930
Consistency index (CI) = 0.5860
Homoplasy index (HI) = 0.4140
CI excluding uninformative characters = 0.4976
HI excluding uninformative characters = 0.5024
Retention index (RI) = 0.6825
Rescaled consistency index (RC) = 0.4000

Supplementary Figure S4 (continue)

Cp CDs

Paup-MP

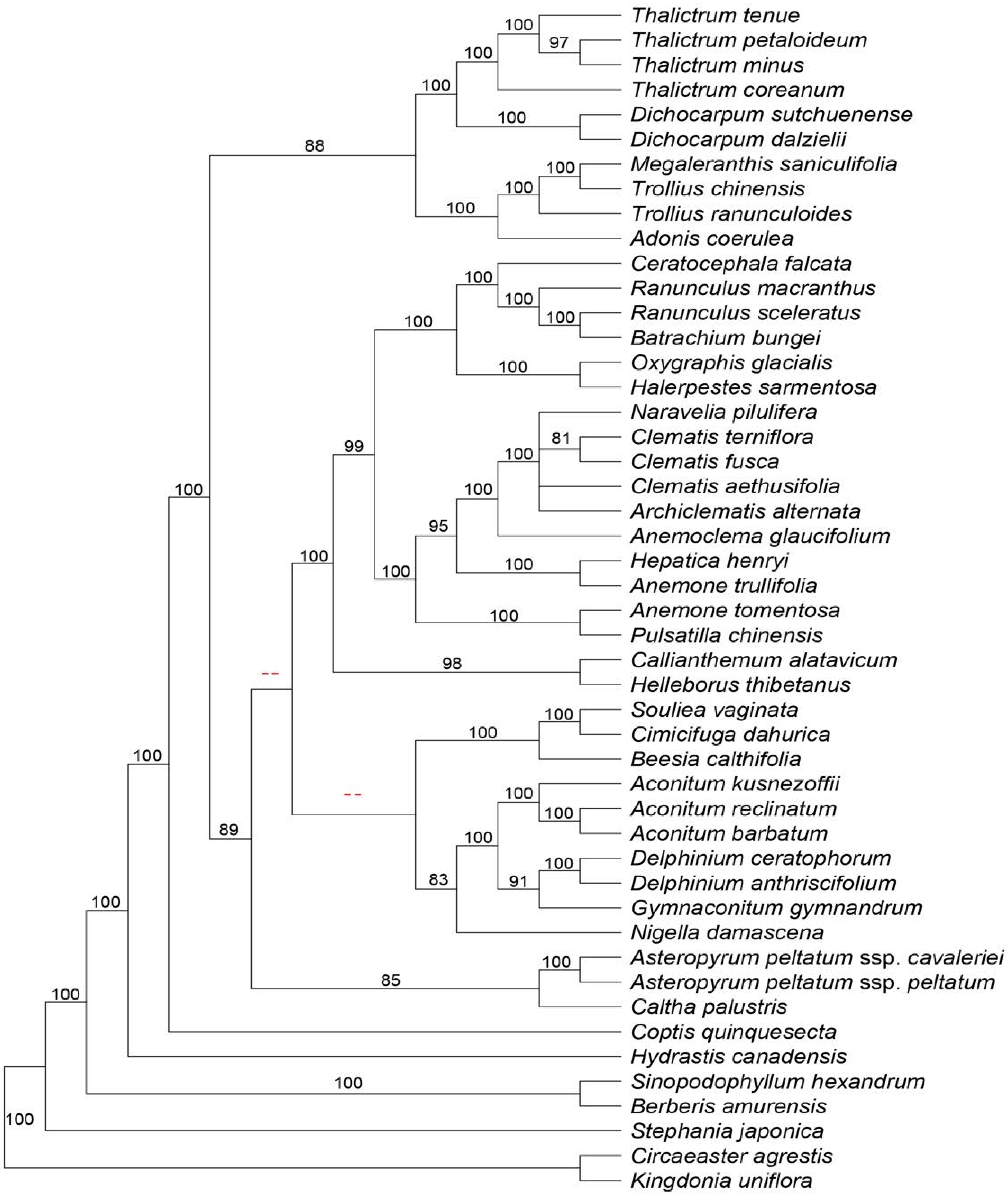

Of 74772 total characters:
All characters are of type 'unord'
All characters have equal weight
50031 characters are constant (proportion = 0.669114)
9537 variable characters are parsimony-uninformative
Number of parsimony-informative characters = 15204

Tree length = 58454
Consistency index (CI) = 0.5803
Homoplasy index (HI) = 0.4197
CI excluding uninformative characters = 0.4839
HI excluding uninformative characters = 0.5161
Retention index (RI) = 0.6834
Rescaled consistency index (RC) = 0.3966
2 trees

Supplementary Figure S4 (continue)

Cp IGS

Paup-MP

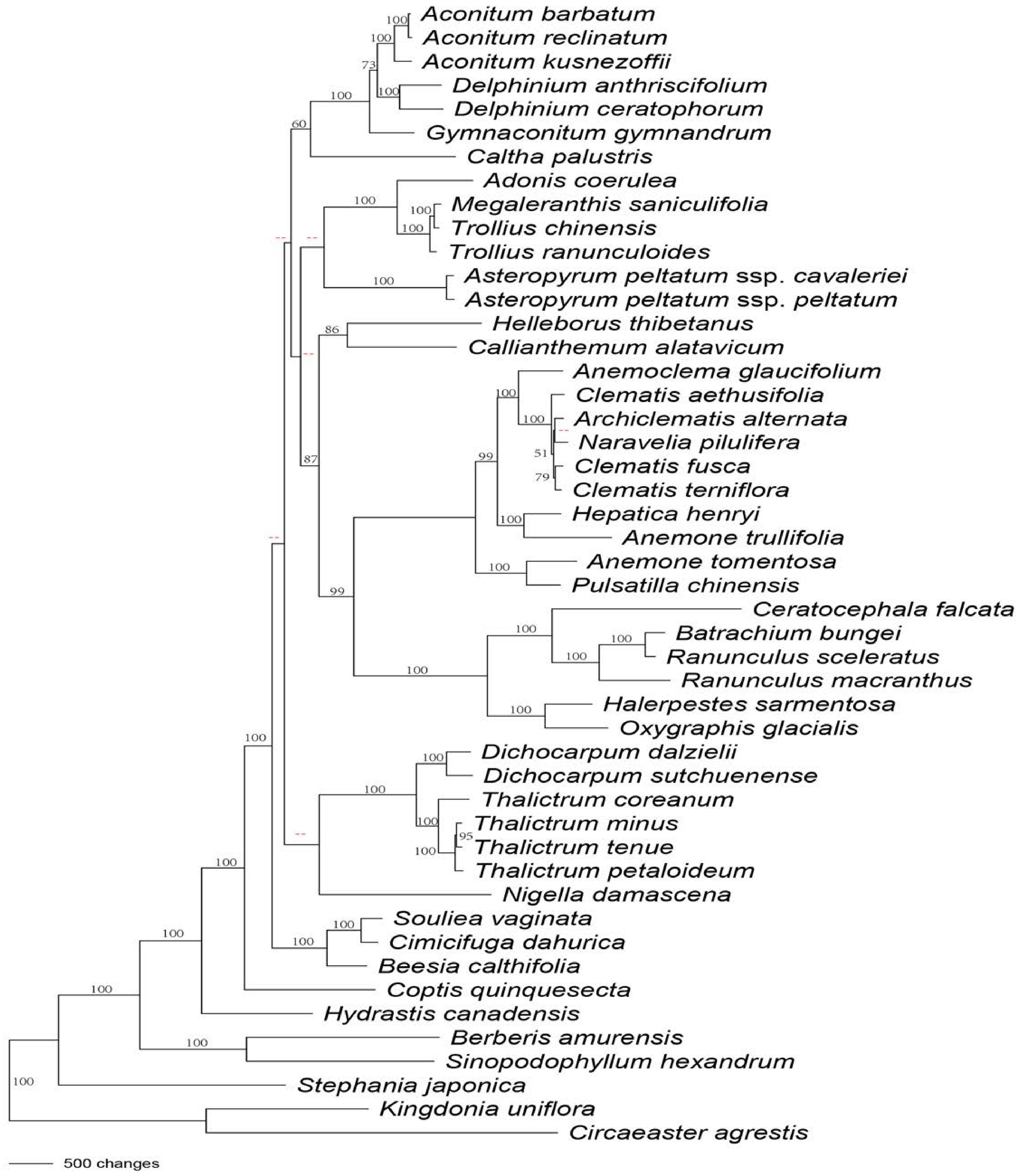

Of 31036 total characters:
All characters are of type 'unord'
All characters have equal weight
9560 characters are constant (proportion = 0.308029)
7395 variable characters are parsimony-uninformative
Number of parsimony-informative characters = 14081

Tree length = 58731
Consistency index (CI) = 0.5841
Homoplasy index (HI) = 0.4159
CI excluding uninformative characters = 0.5066
HI excluding uninformative characters = 0.4934
Retention index (RI) = 0.6781
Rescaled consistency index (RC) = 0.3961

Supplementary Figure S4 (continue)

Cp Intron

Paup-MP

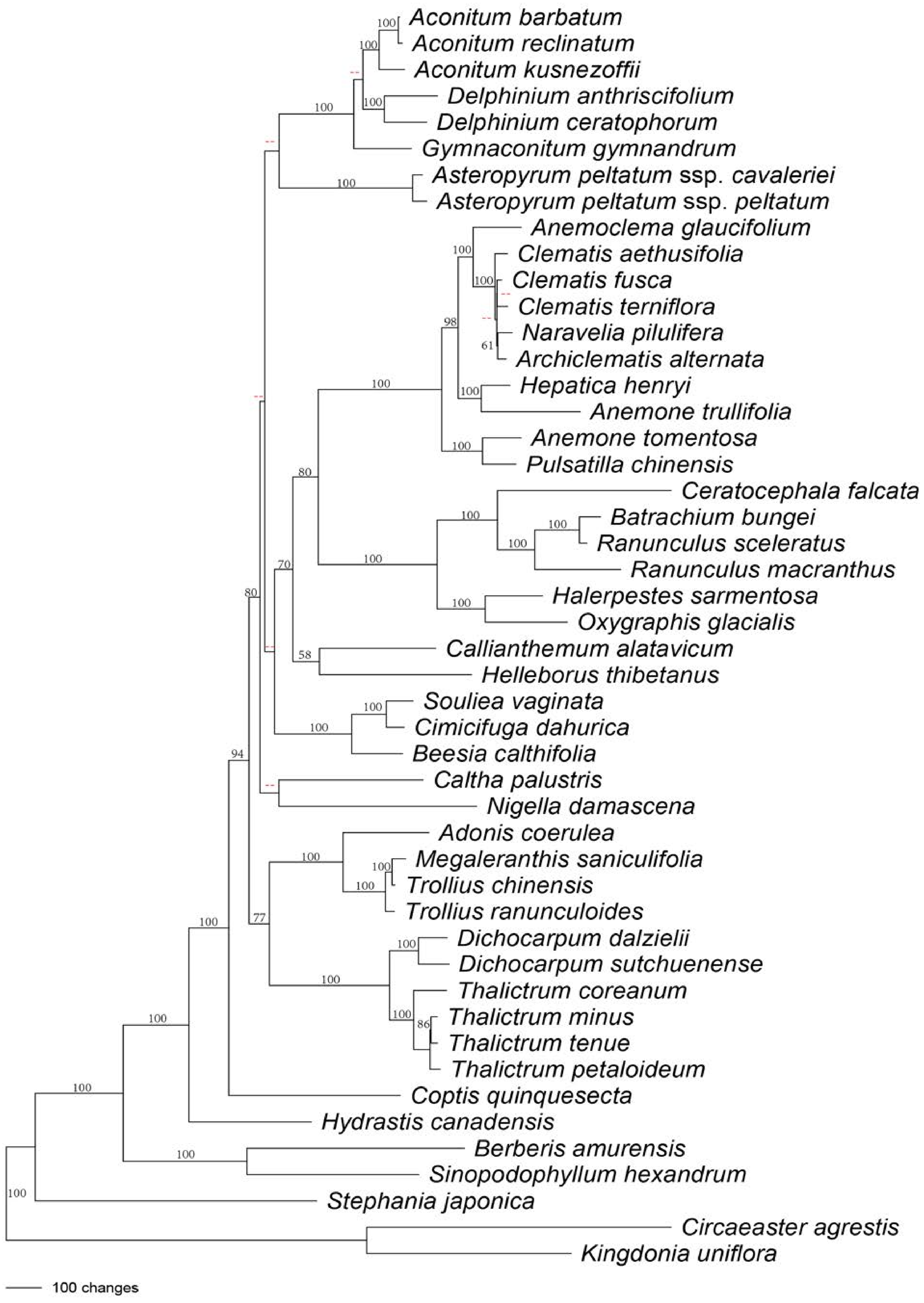

Of 14373 total characters:
All characters are of type 'unord'
All characters have equal weight
7618 characters are constant (proportion = 0.530022)
2776 variable characters are parsimony-uninformative
Number of parsimony-informative characters = 3979

Tree length = 15648
Consistency index (CI) = 0.6182
Homoplasy index (HI) = 0.3818
CI excluding uninformative characters = 0.5181
HI excluding uninformative characters = 0.4819
Retention index (RI) = 0.7007
Rescaled consistency index (RC) = 0.4332

Supplementary Figure S4 (continue)

Cp LSC

Paup-MP

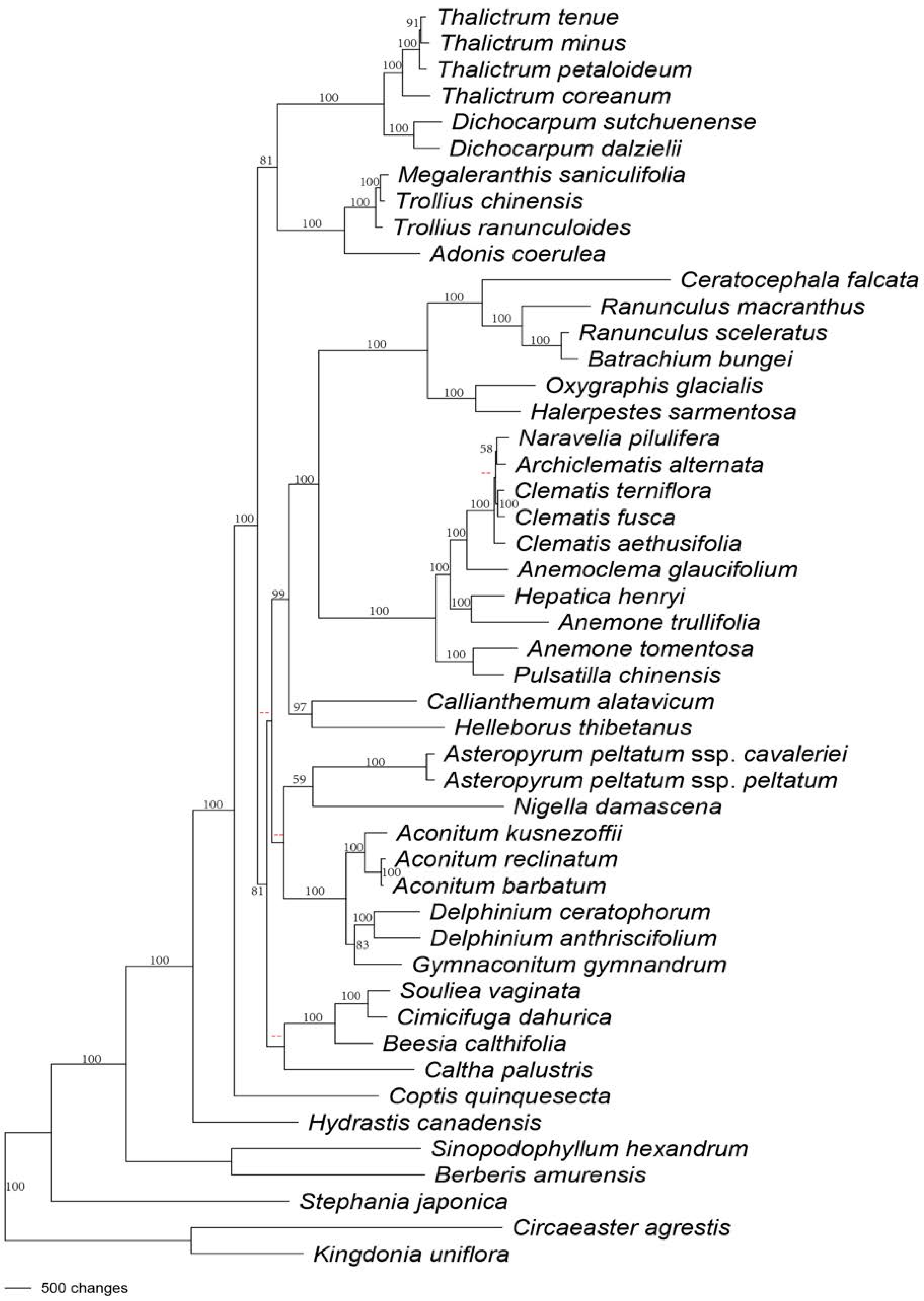

Of 77968 total characters:
All characters are of type 'unord'
All characters have equal weight
41042 characters are constant (proportion = 0.526395)
12885 variable characters are parsimony-uninformative
Number of parsimony-informative characters = 24041

Tree length = 93269
Consistency index (CI) = 0.5850
Homoplasy index (HI) = 0.4150
CI excluding uninformative characters = 0.5027
HI excluding uninformative characters = 0.4973
Retention index (RI) = 0.6891
Rescaled consistency index (RC) = 0.4031

Supplementary Figure S4 (continue)

Cp SSC

Paup-MP

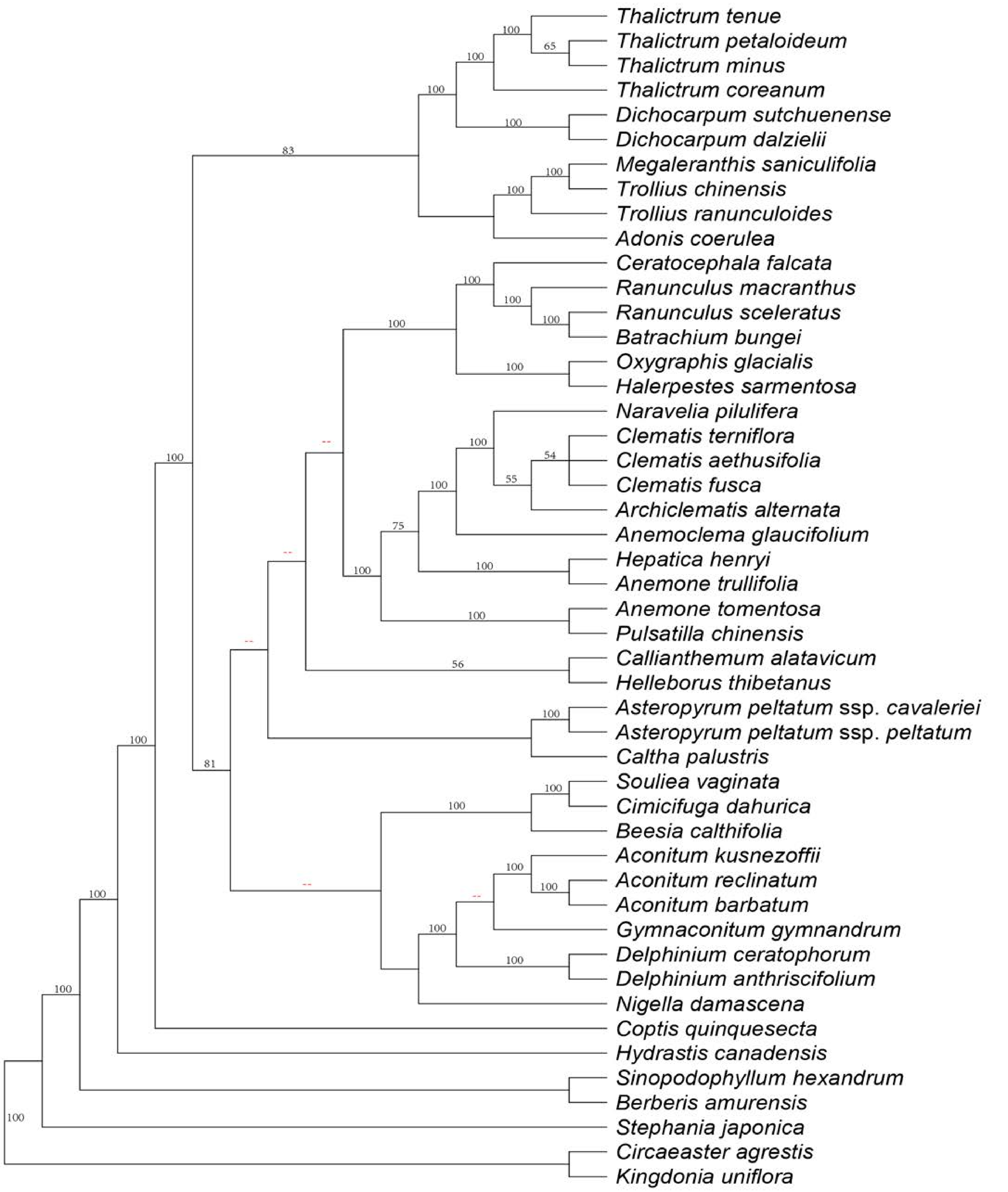

Of 18551 total characters:
All characters are of type 'unord'
All characters have equal weight
7521 characters are constant (proportion = 0.405423)
3385 variable characters are parsimony-uninformative
Number of parsimony-informative characters = 7645

Tree length = 32922
Consistency index (CI) = 0.5409
Homoplasy index (HI) = 0.4591
CI excluding uninformative characters = 0.4728
HI excluding uninformative characters = 0.5272
Retention index (RI) = 0.6496
Rescaled consistency index (RC) = 0.3514
2 trees

Supplementary Figure S4 (continue)

Cp IR

Paup-MP

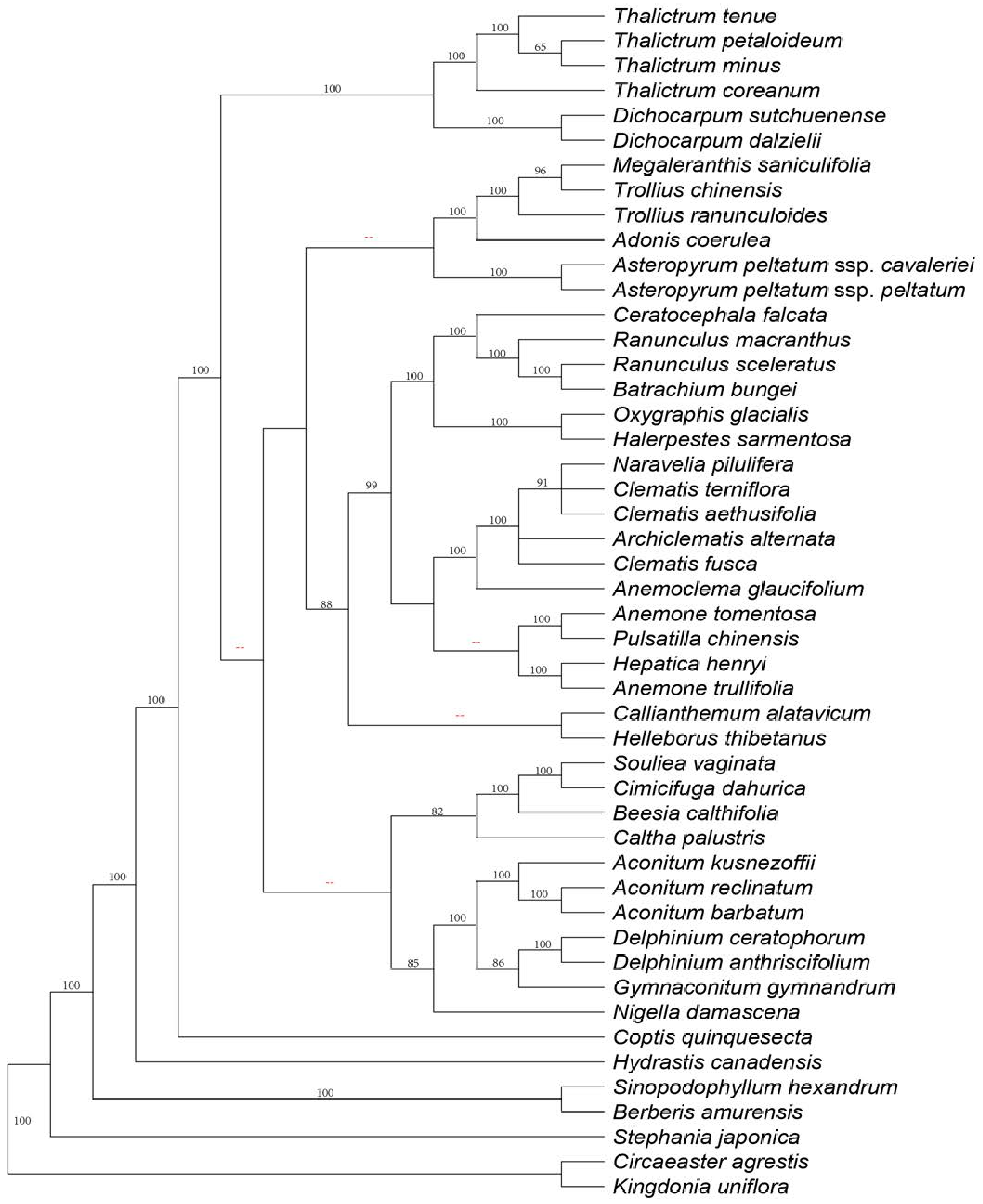

Of 24318 total characters:
All characters are of type 'unord'
All characters have equal weight
18605 characters are constant (proportion = 0.765071)
3647 variable characters are parsimony-uninformative
Number of parsimony-informative characters = 2066

Tree length = 8245
Consistency index (CI) = 0.8018
Homoplasy index (HI) = 0.1982
CI excluding uninformative characters = 0.6168
HI excluding uninformative characters = 0.3832
Retention index (RI) = 0.7851
Rescaled consistency index (RC) = 0.6295
3 trees
