# Supplementary material for: Structural variation of the complete chloroplast genome and plastid phylogenomics of the genus Asteropyrum (Ranunculaceae)
Source: Sci Rep. 2019 Oct 25;9:15285. doi: 10.1038/s41598-019-51601-2 (PMC6814708; doi:10.1038/s41598-019-51601-2)

Supplementary Figure S1

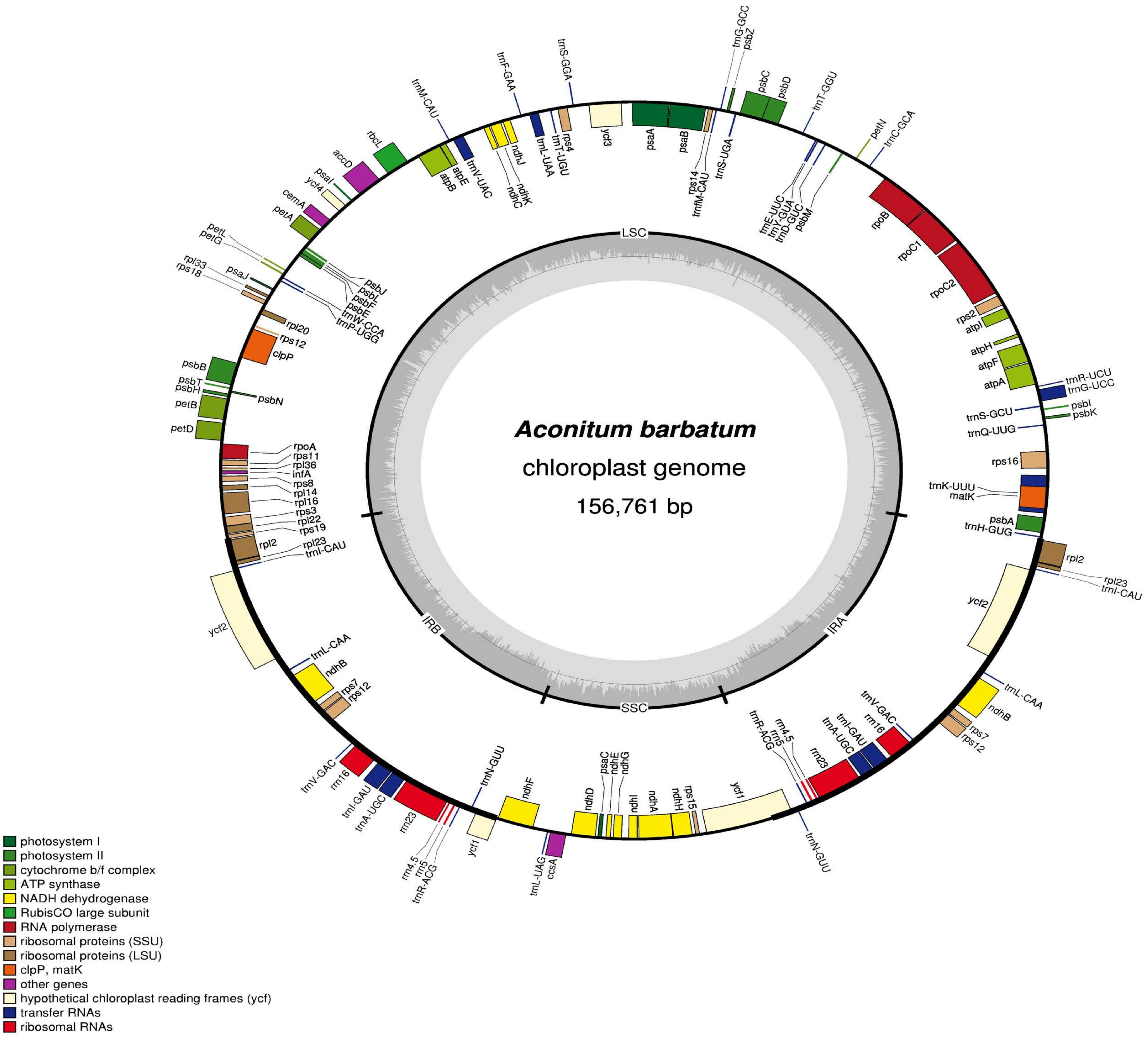

Supplementary Figure S1 (continue)

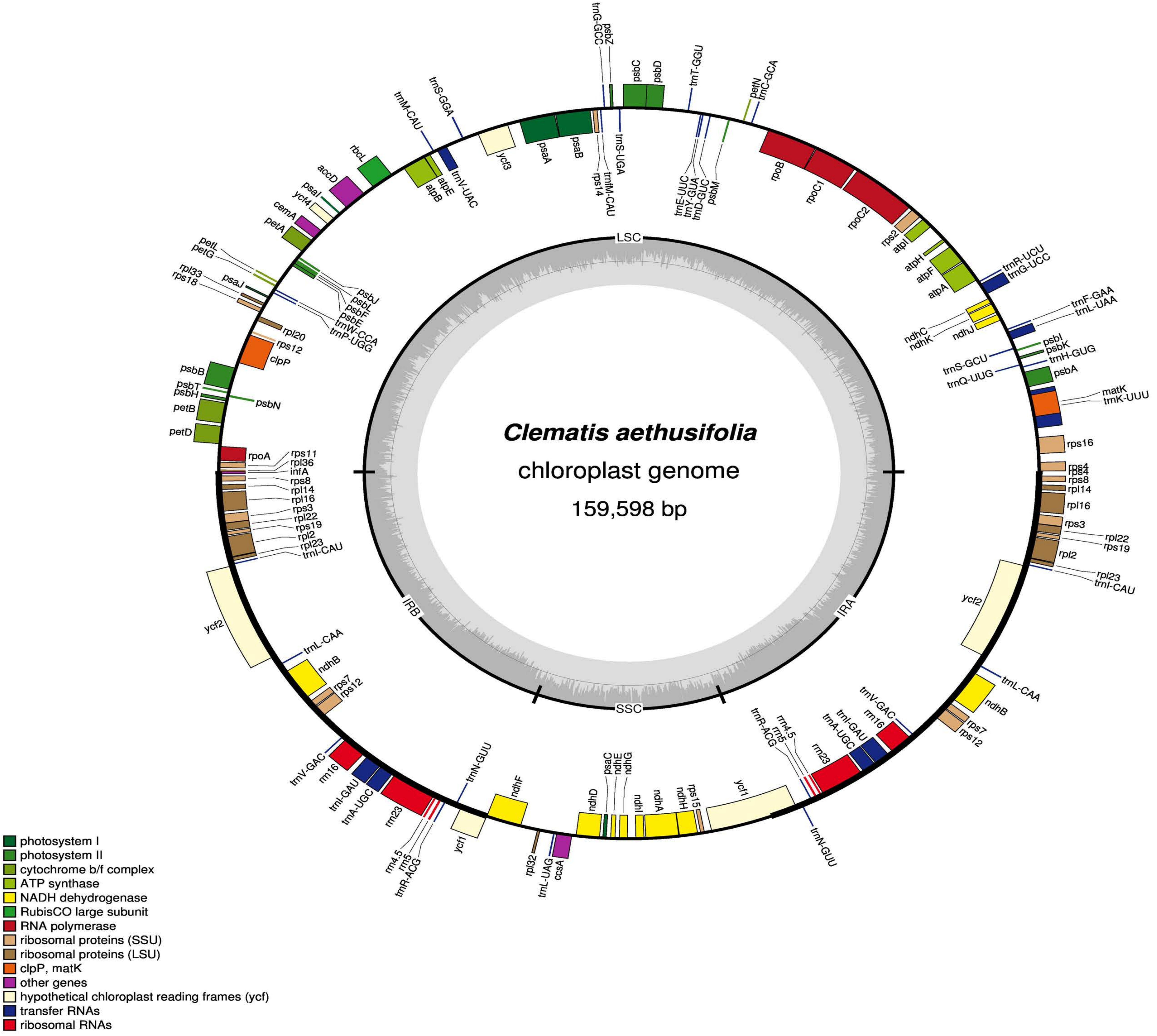

Supplementary Figure S1 (continue)

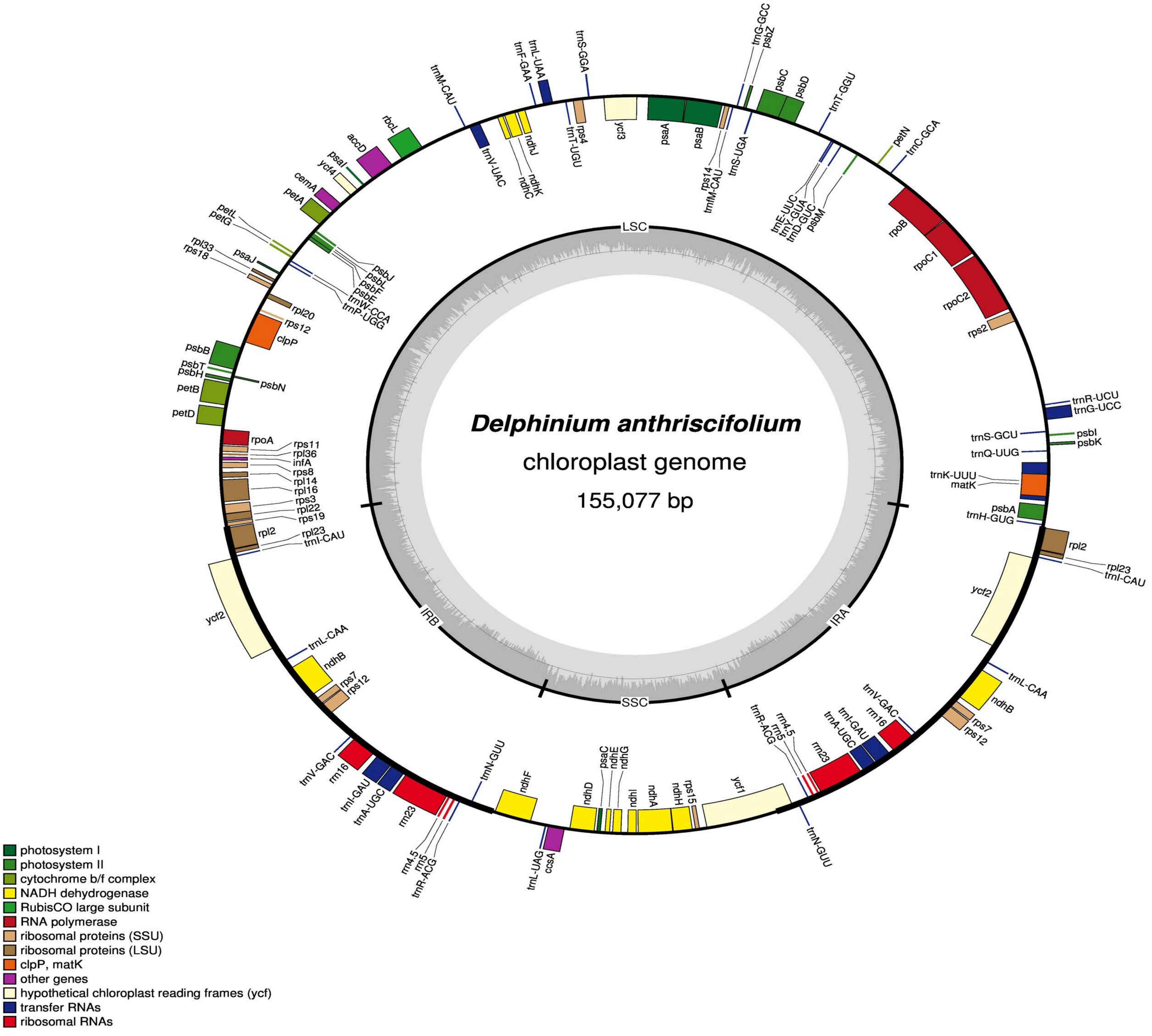

Supplementary Figure S1 (continue)

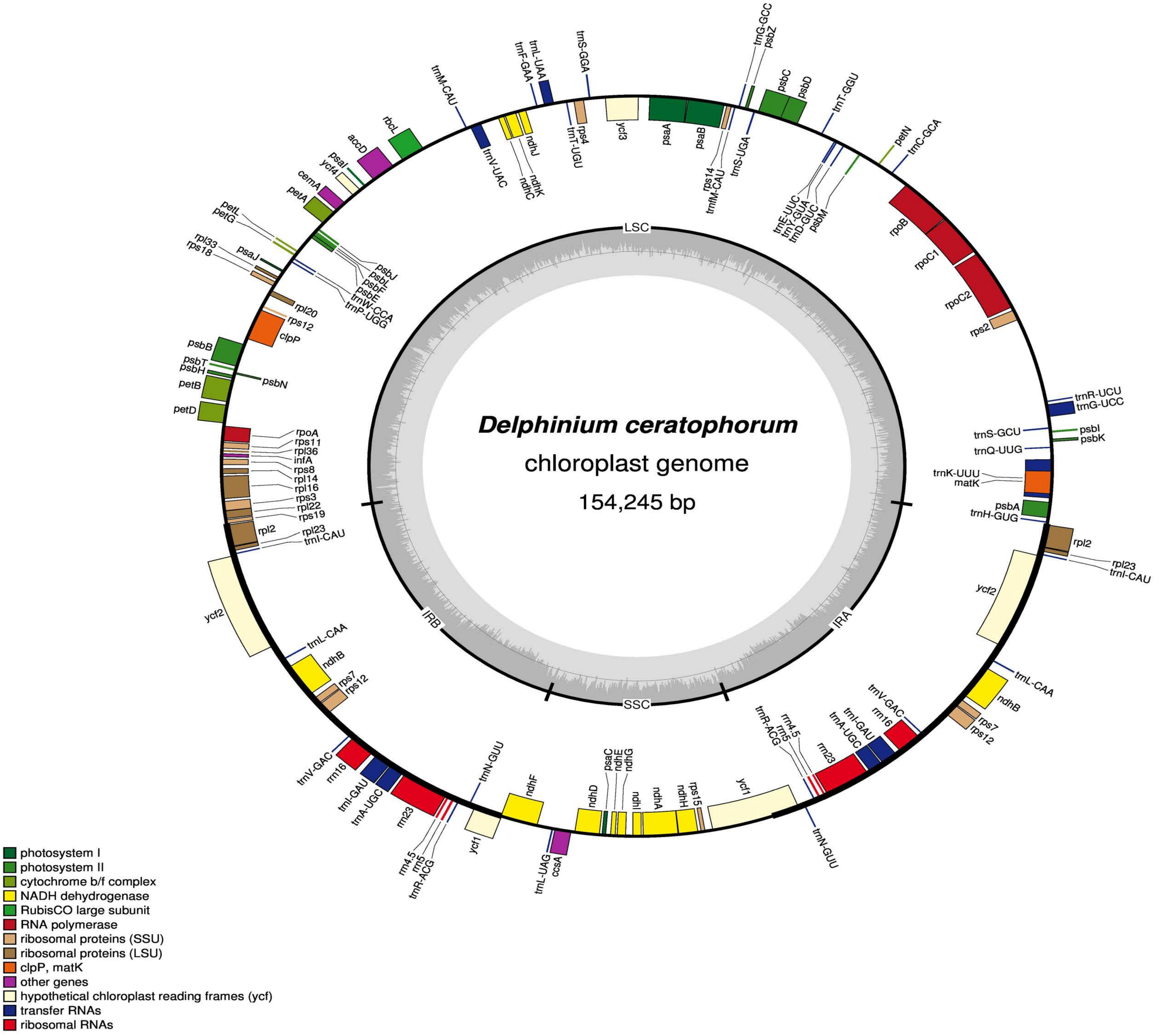

Supplementary Figure S1 (continue)

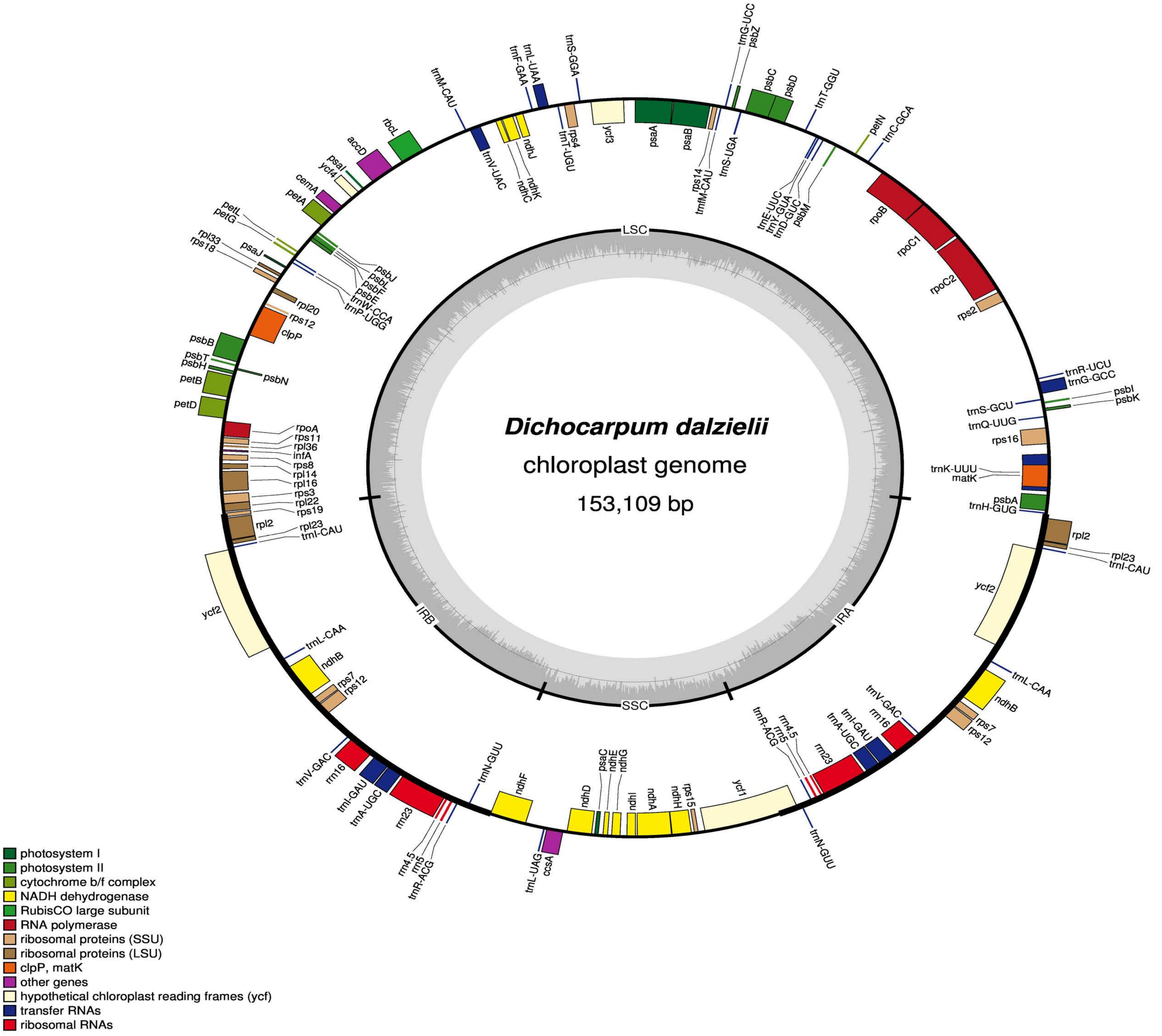

Supplementary Figure S1 (continue)

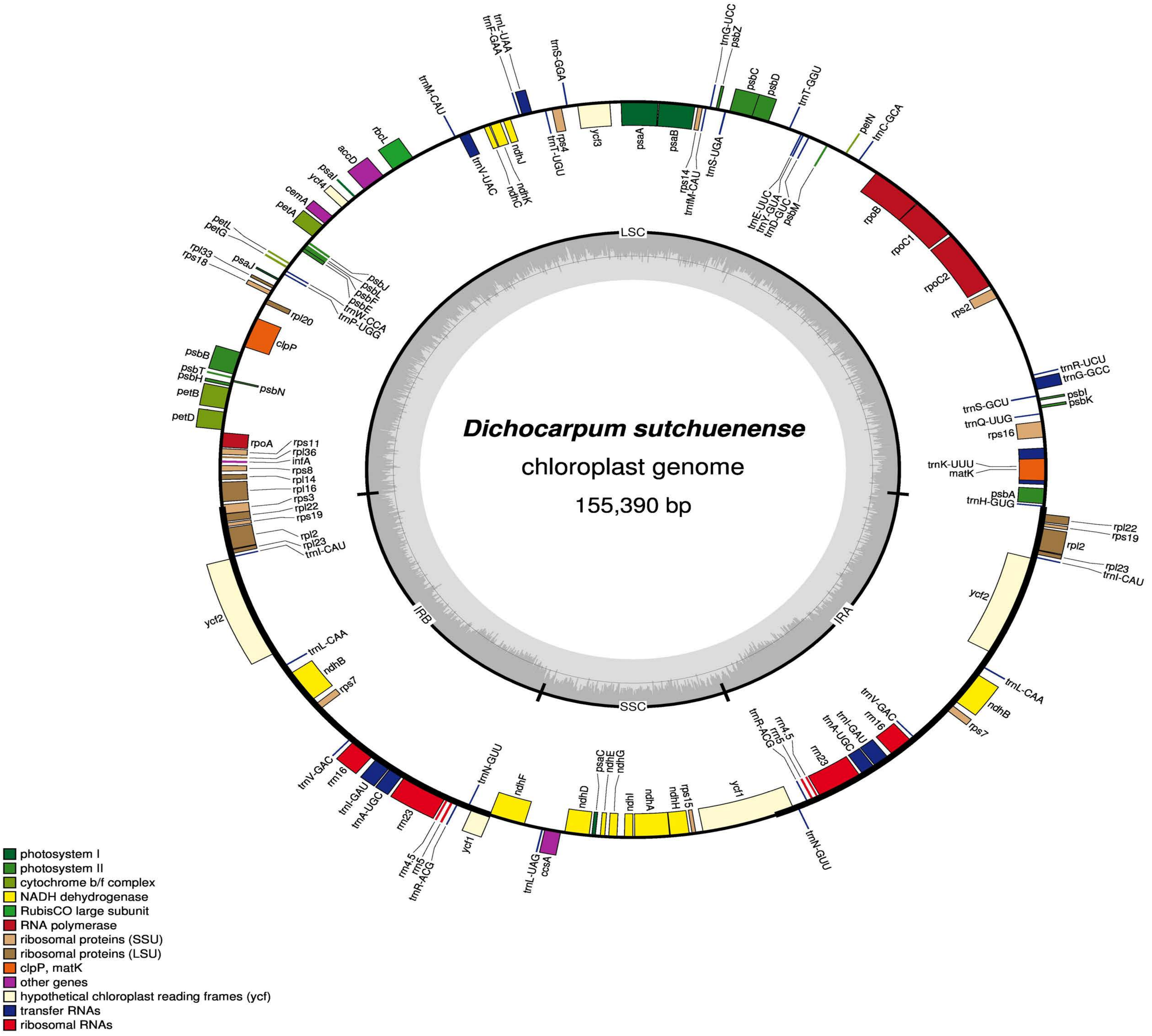

Supplementary Figure S1 (continue)

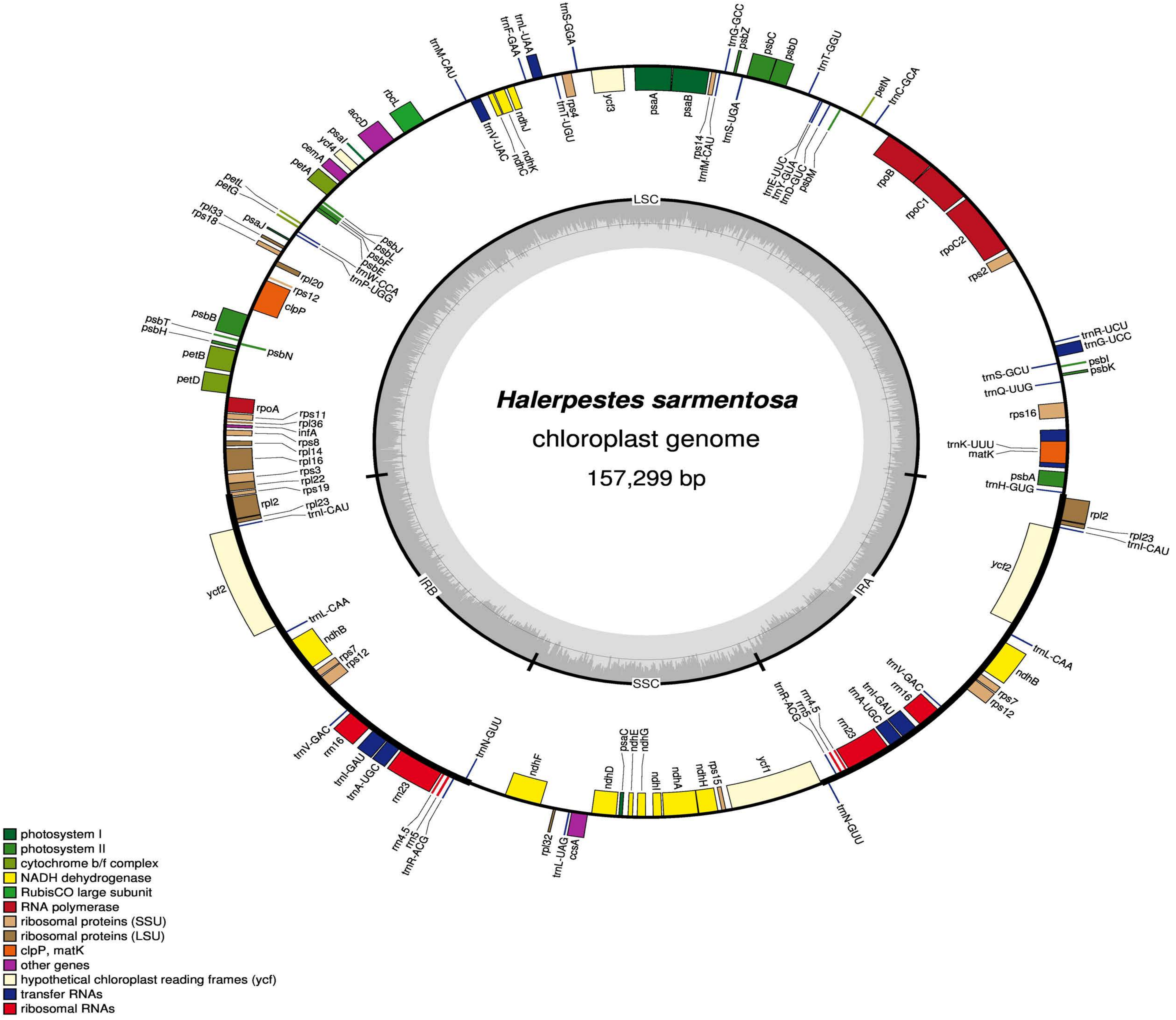

Supplementary Figure S1 (continue)

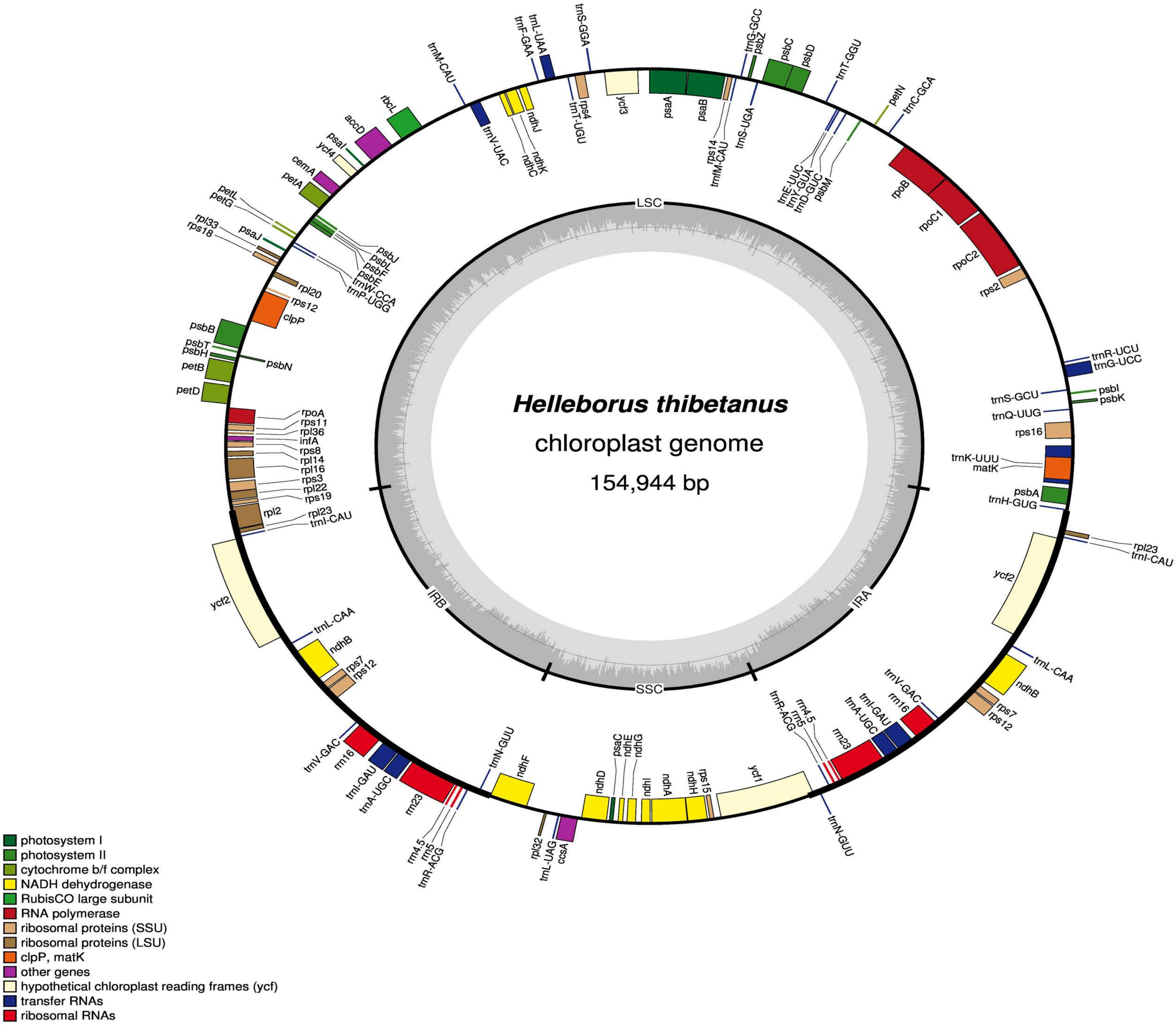

Supplementary Figure S1 (continue)

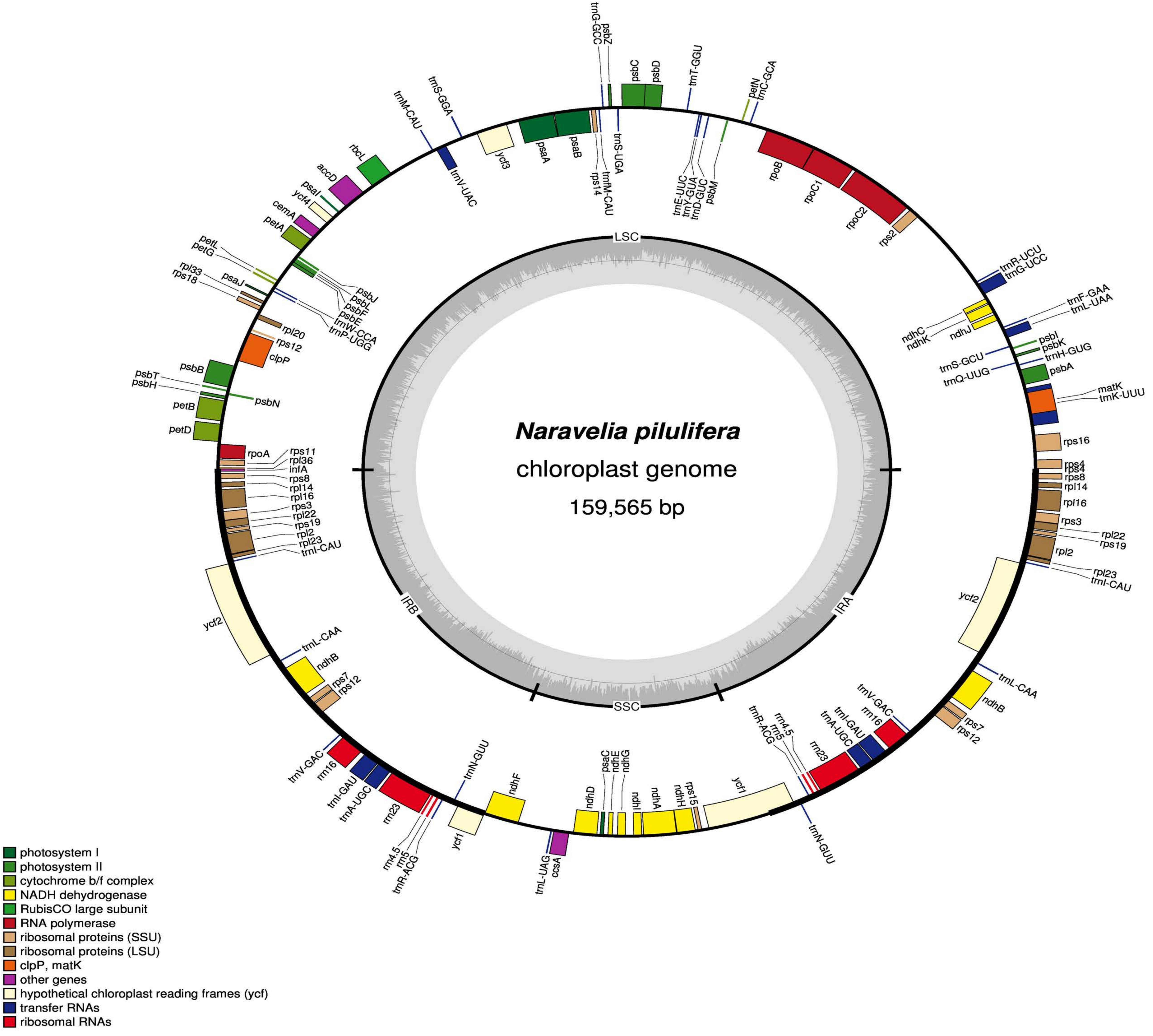

Supplementary Figure S1 (continue)

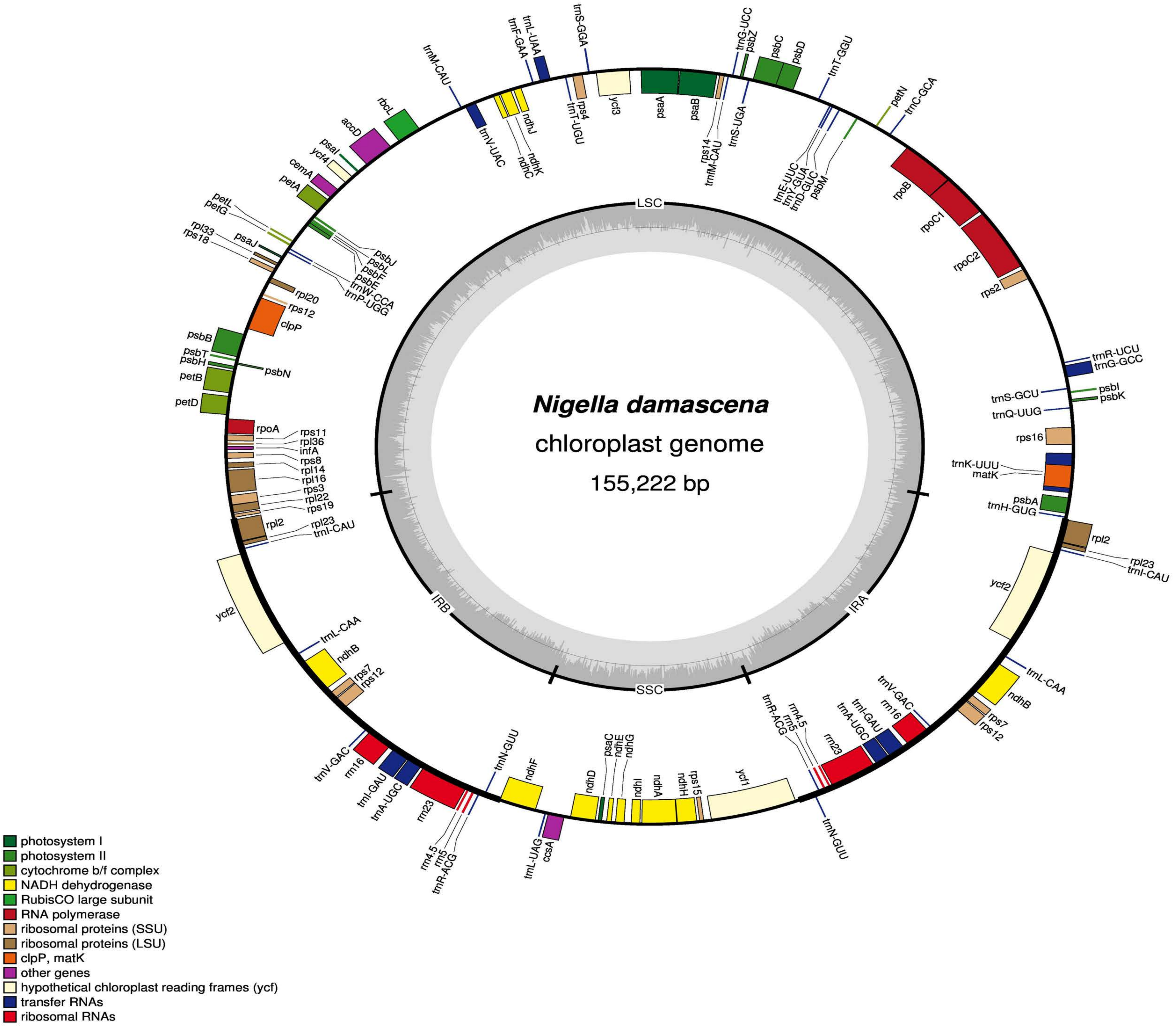

Supplementary Figure S1 (continue)

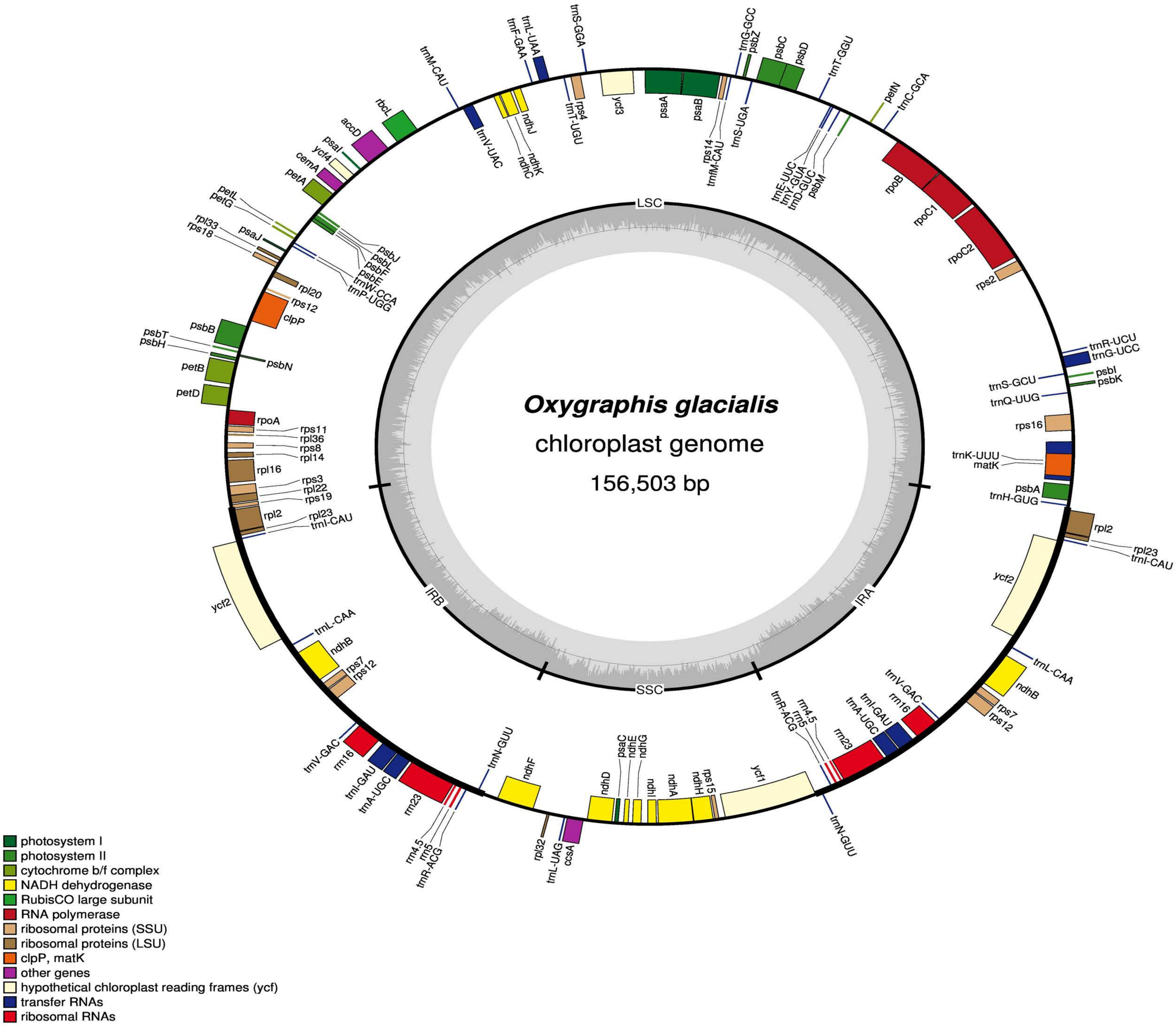

Supplementary Figure S1 (continue)

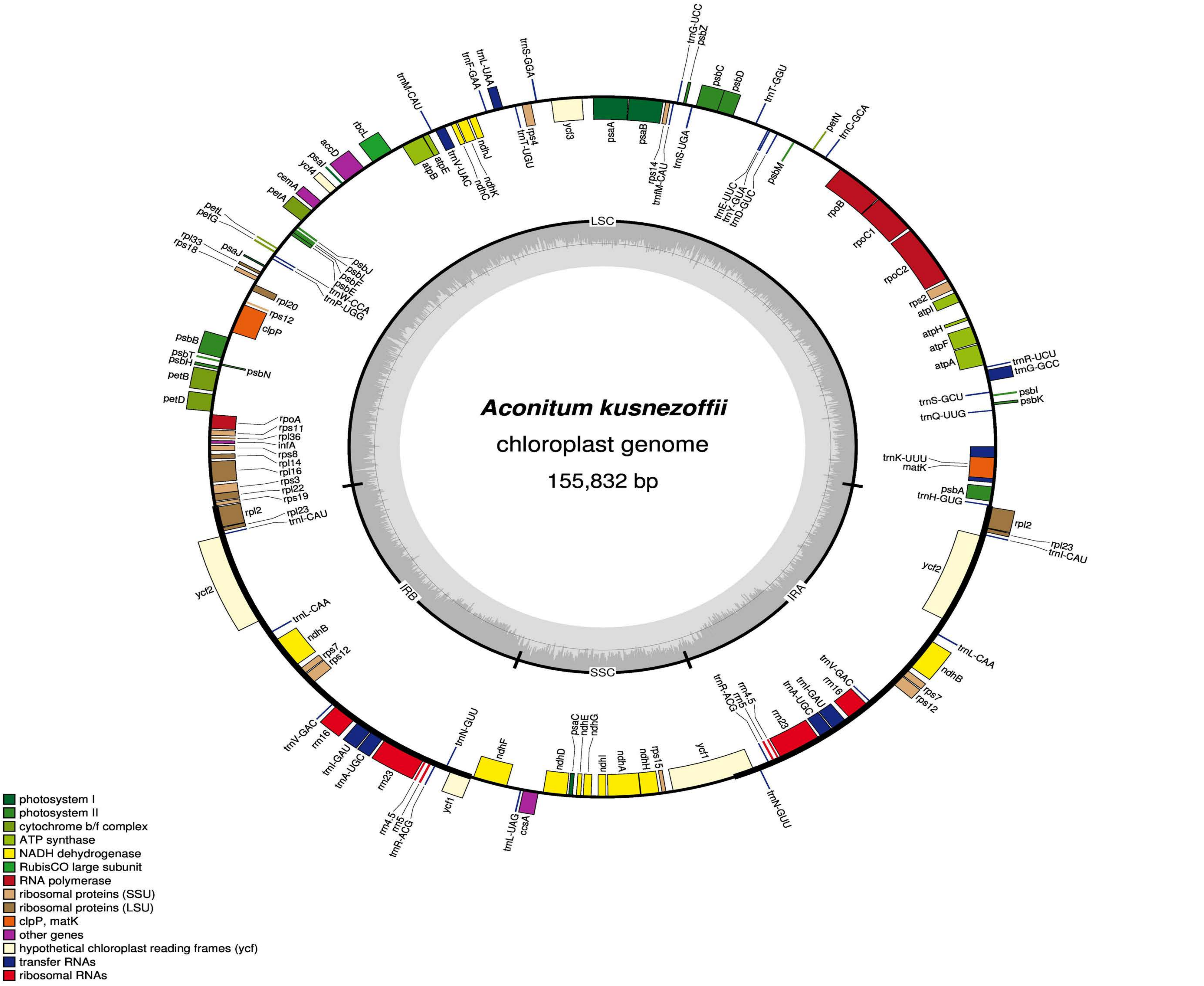

Supplementary Figure S1 (continue)

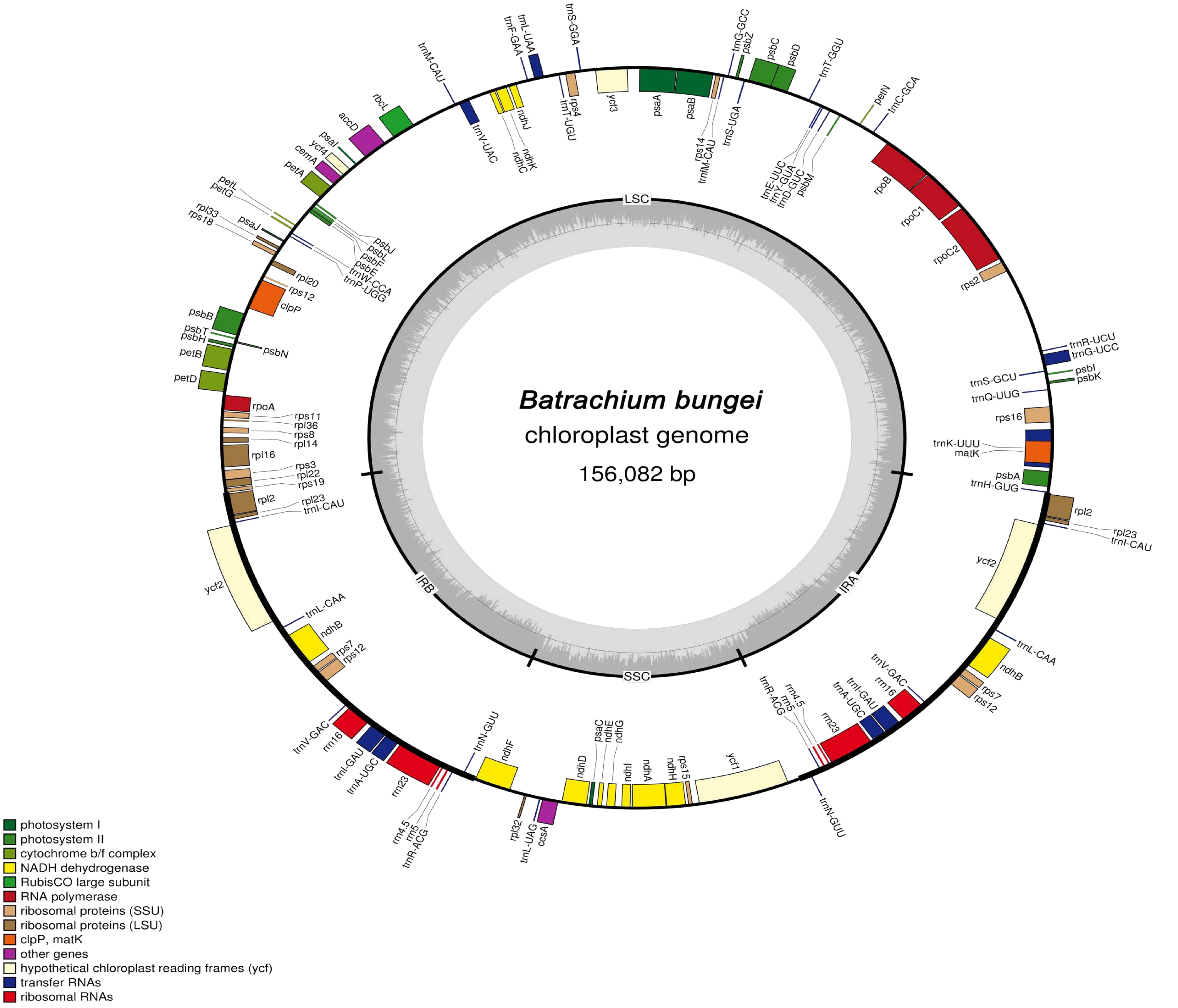

Supplementary Figure S1 (continue)

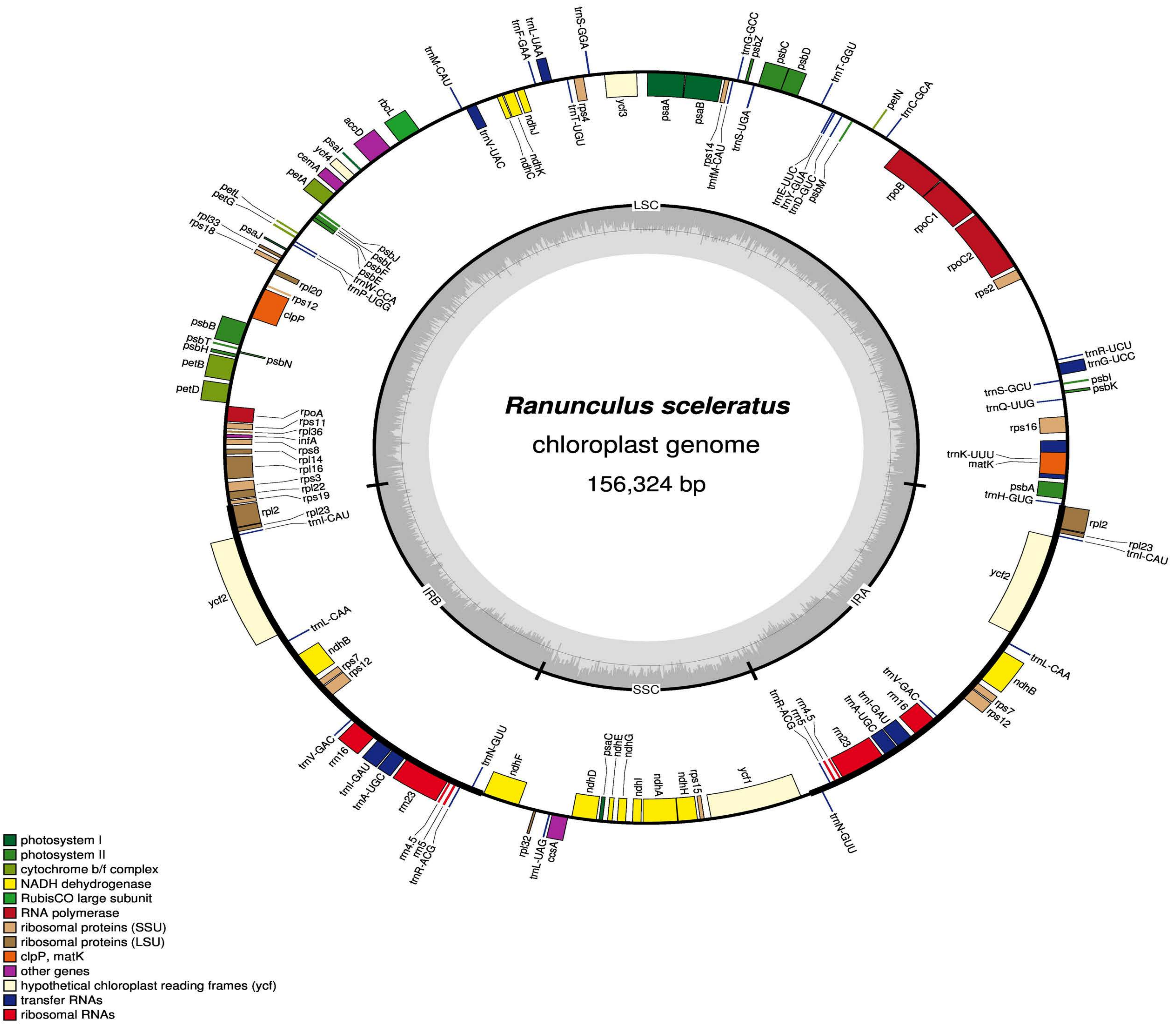

Supplementary Figure S1 (continue)

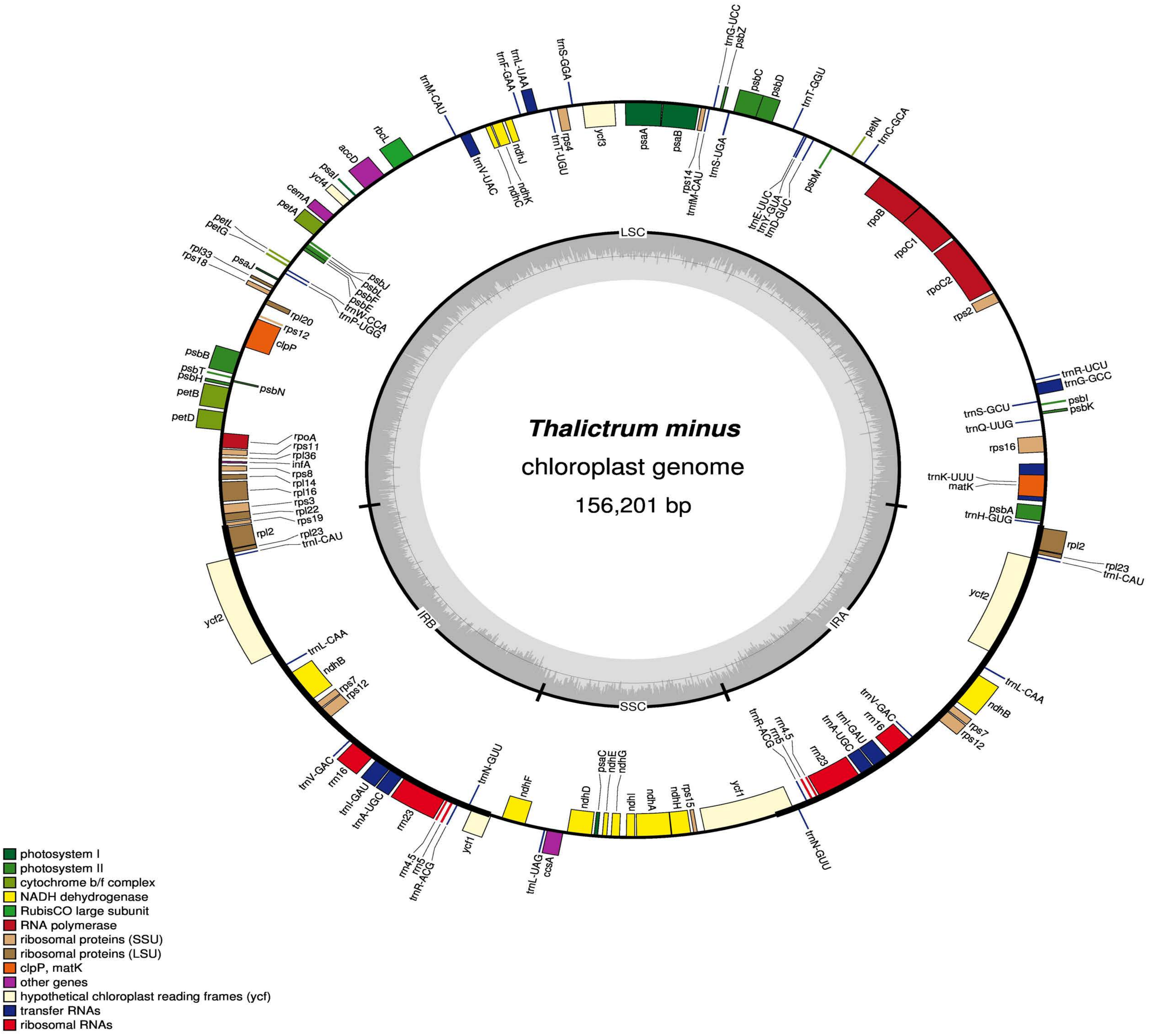

Supplementary Figure S1 (continue)

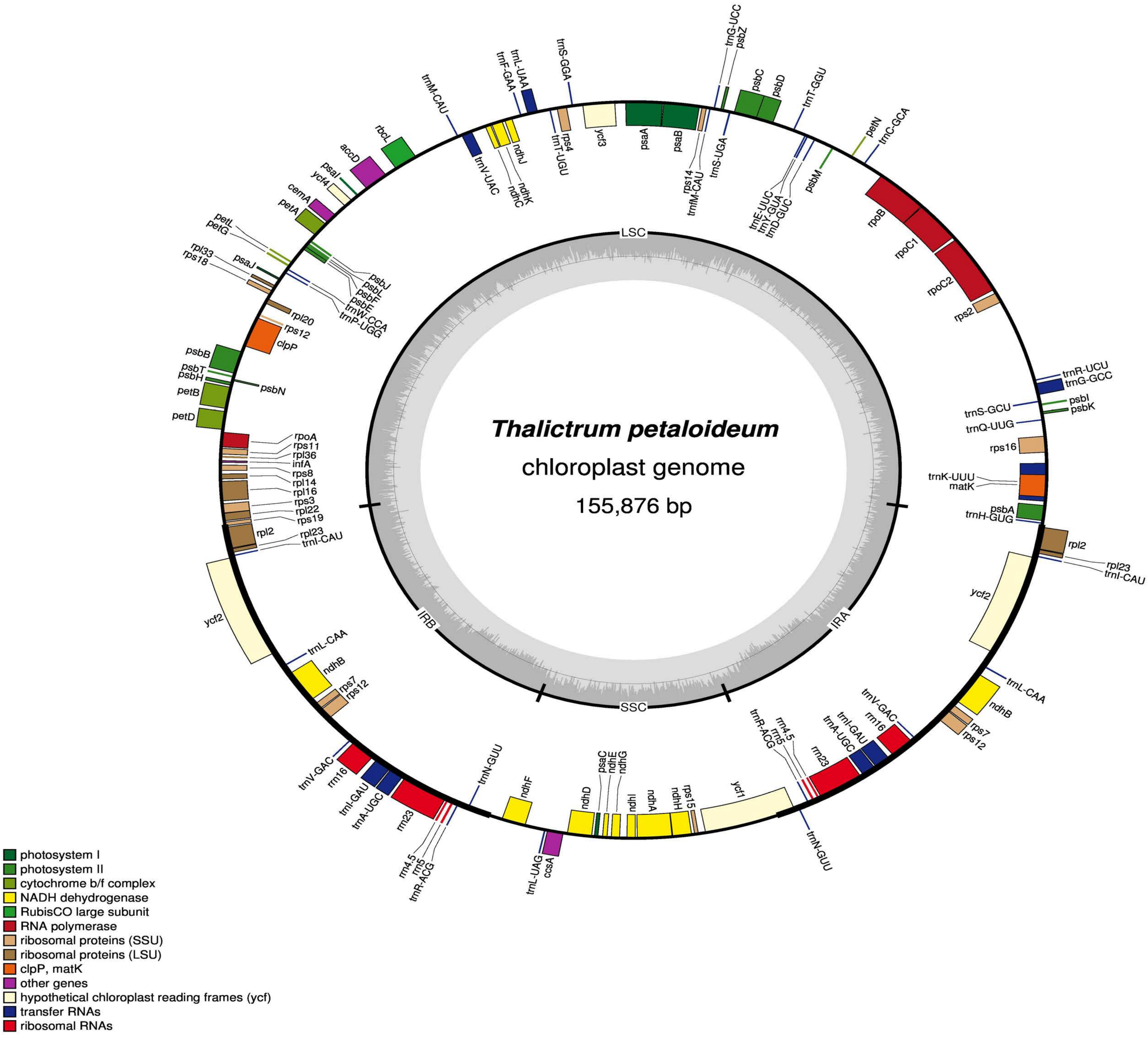

Supplementary Figure S1 (continue)

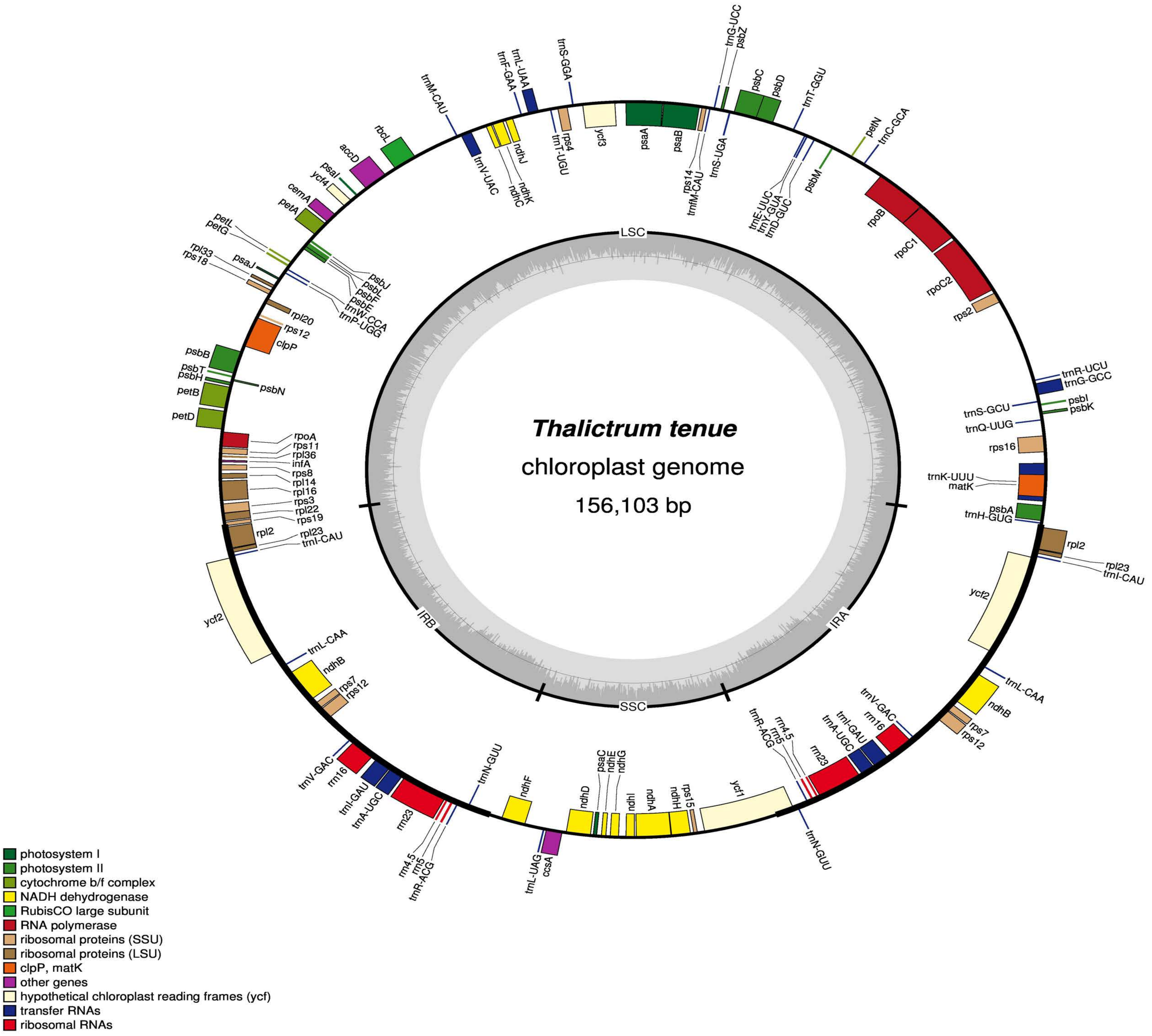

Supplementary Figure S1 (continue)

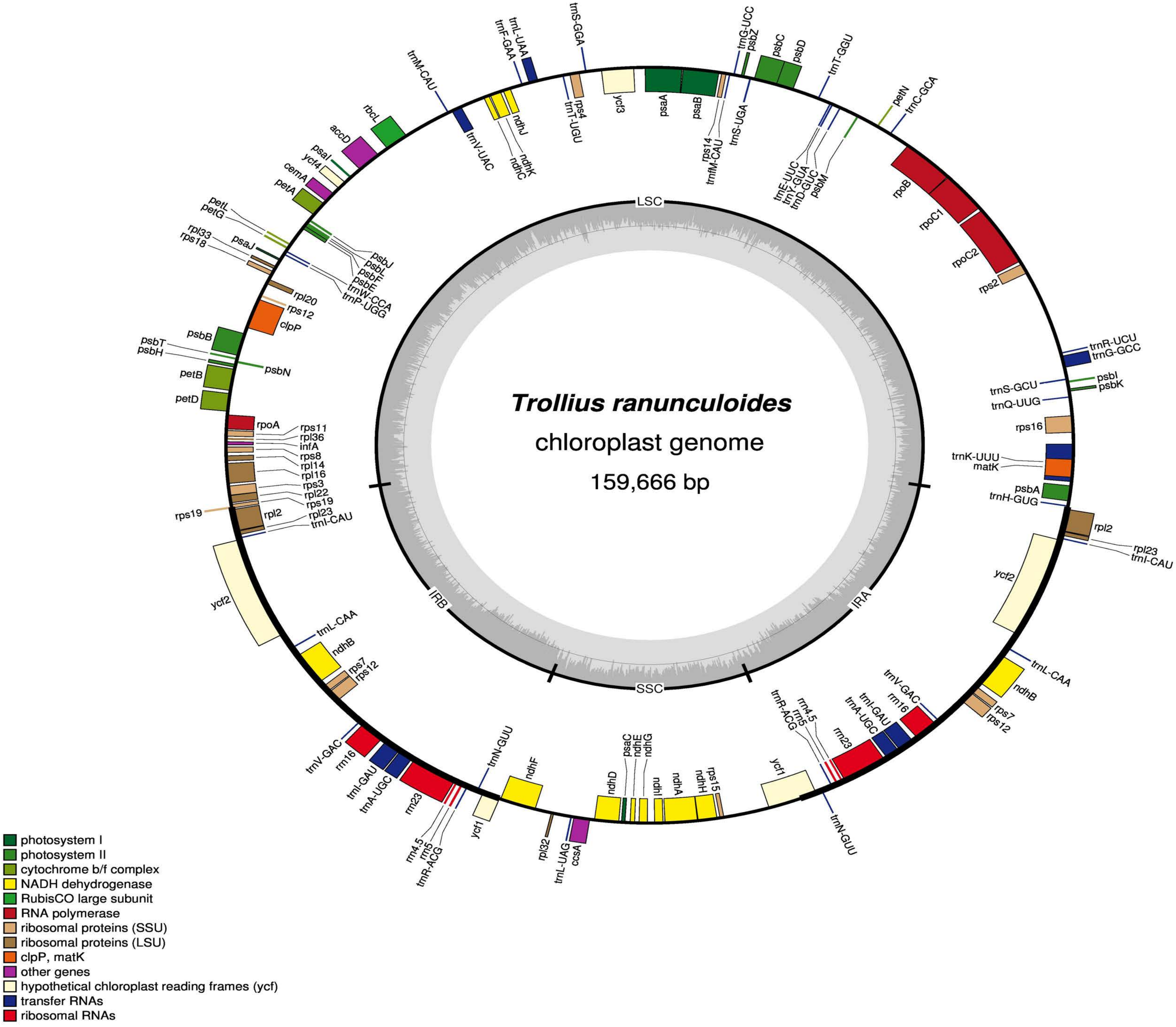

Supplementary Figure S1 (continue)

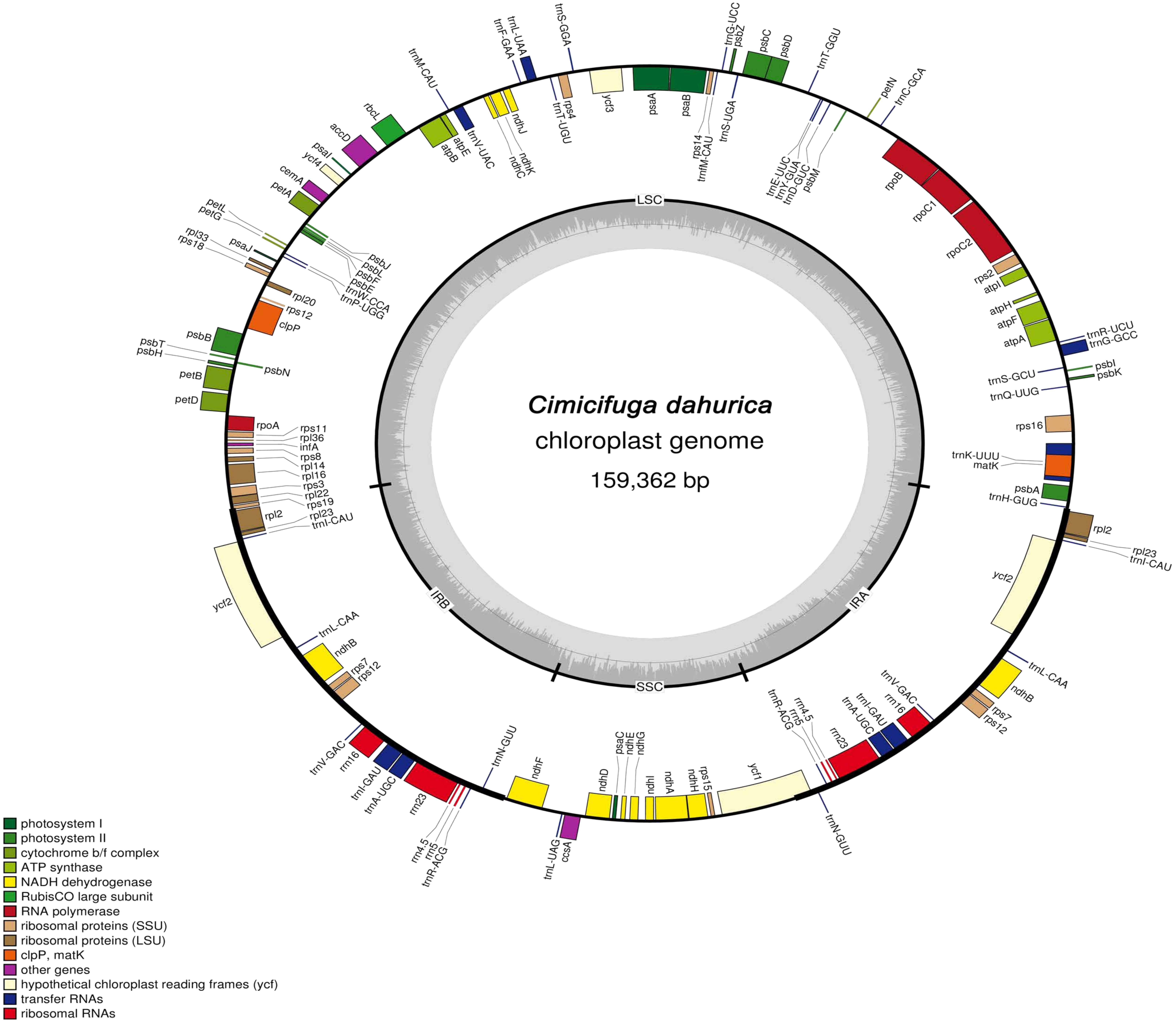

Supplementary Figure S1 (continue)

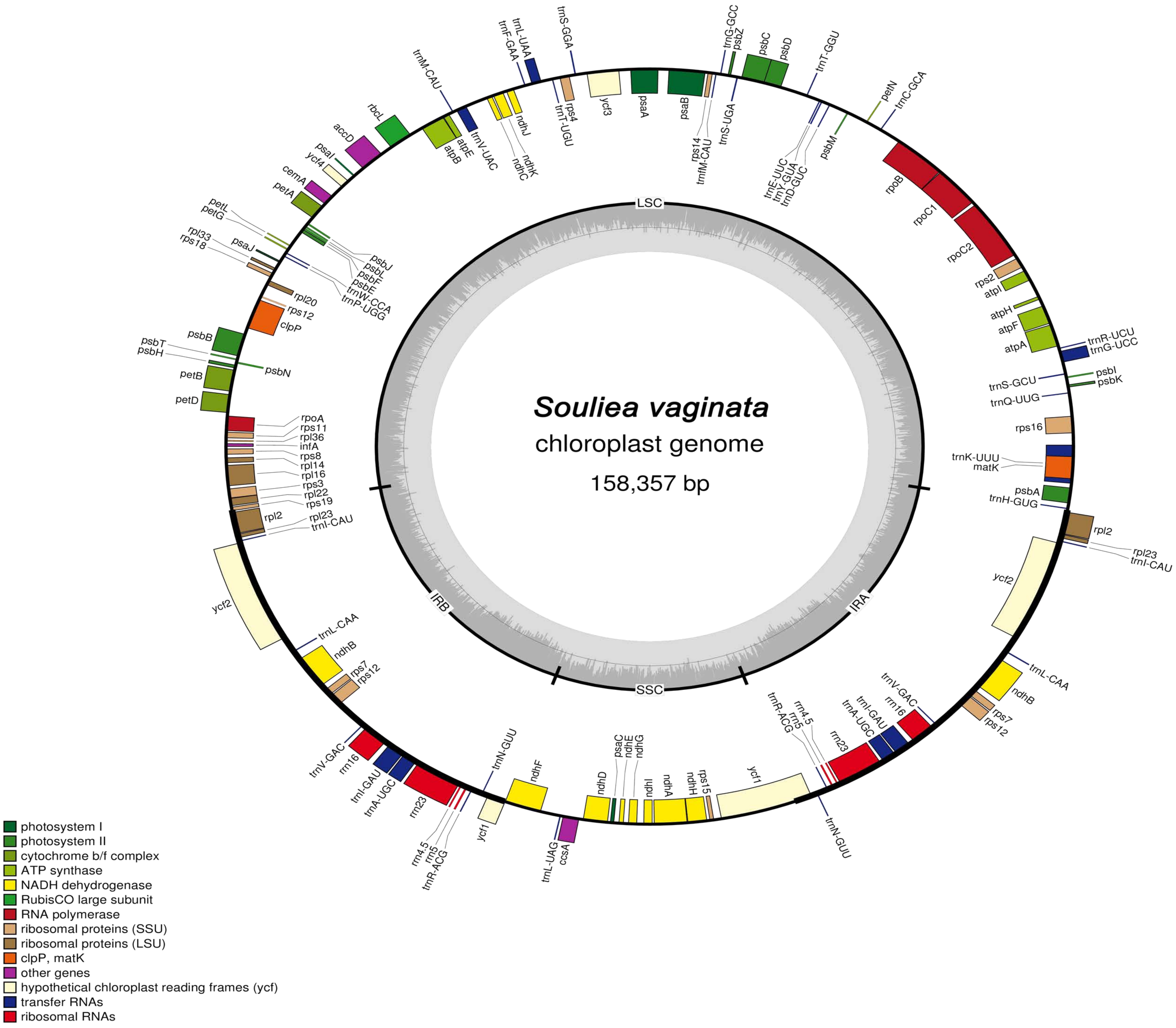

Supplementary Figure S1 (continue)

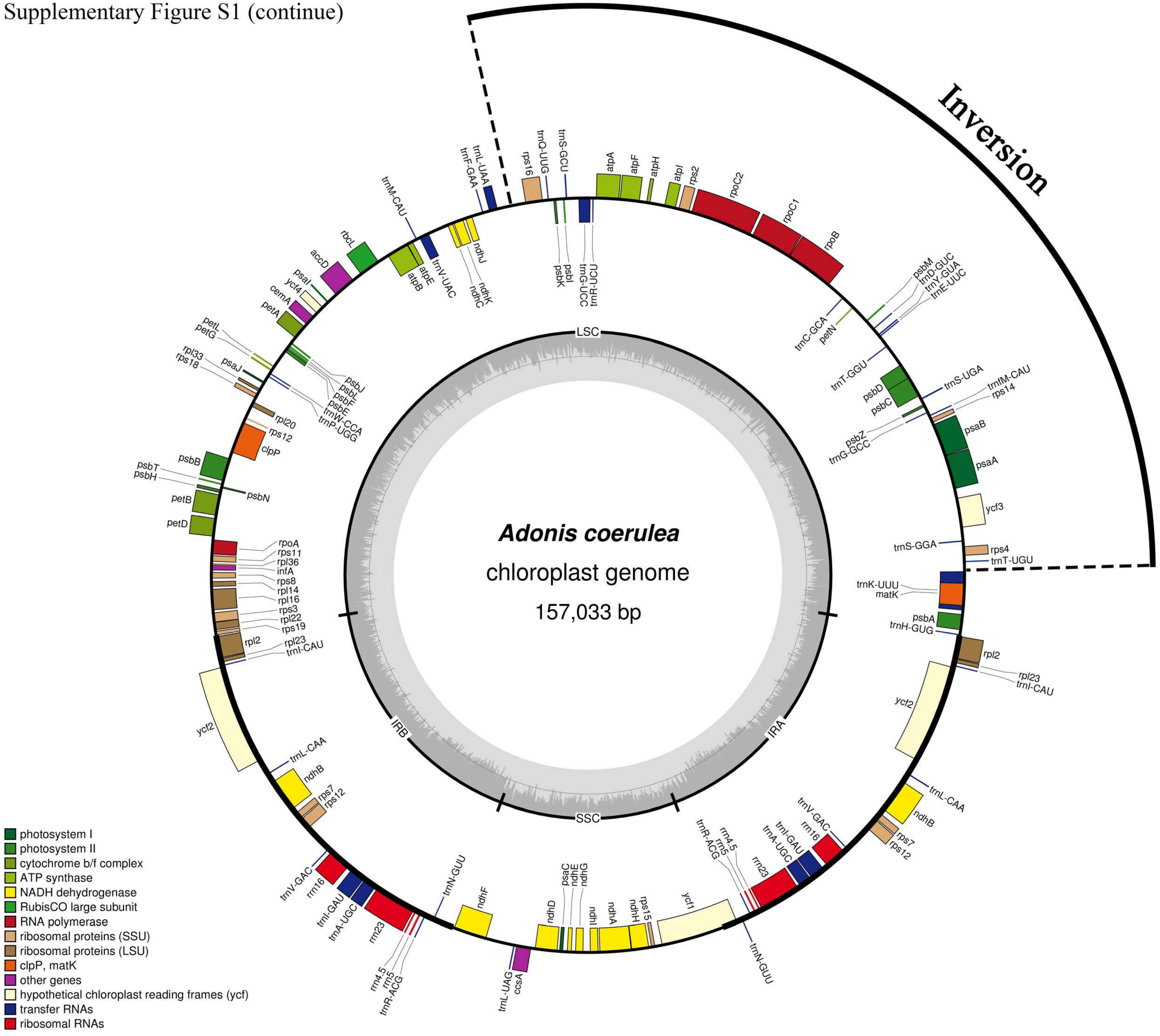

Supplementary Figure S1 (continue)

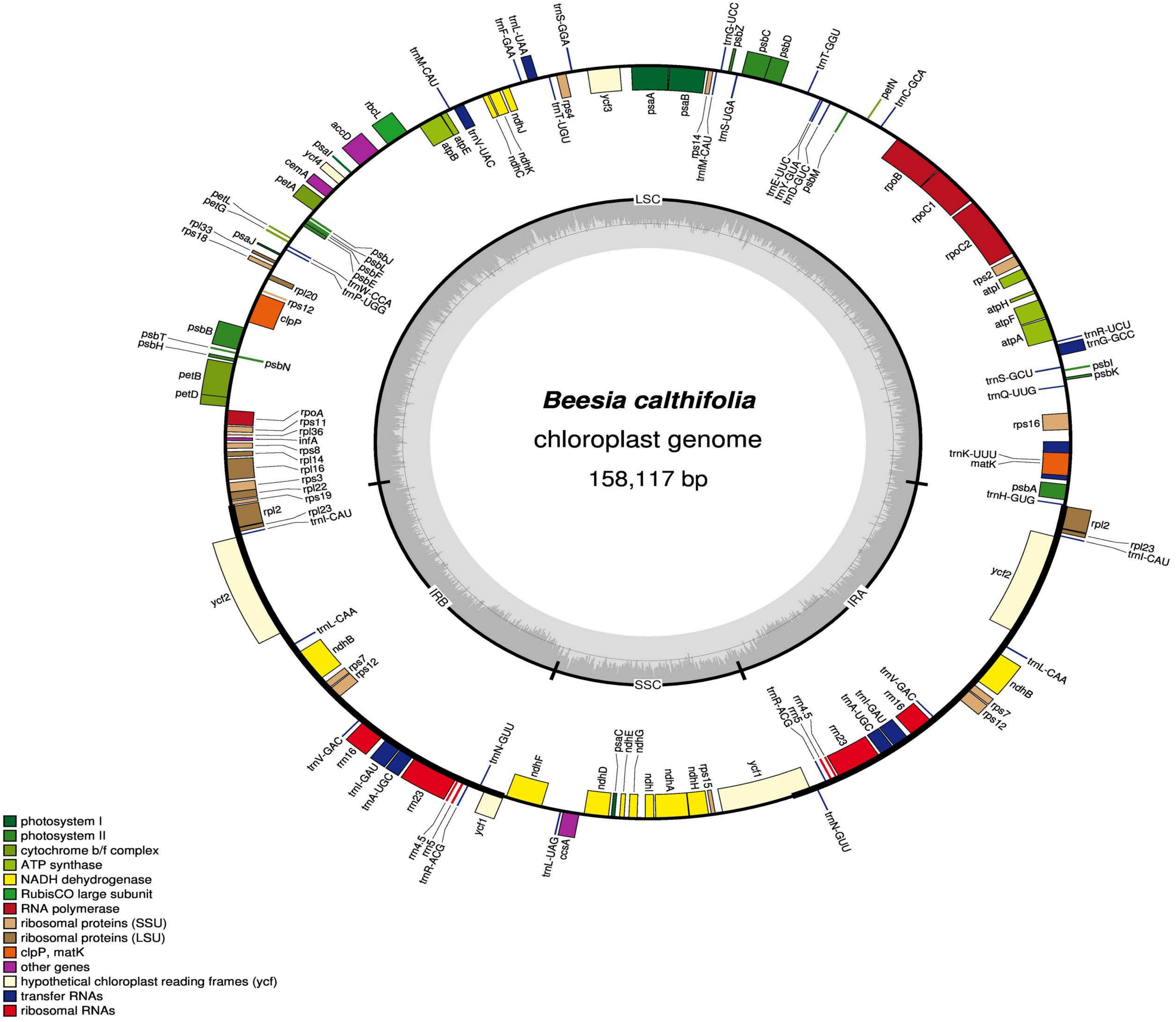

Supplementary Figure S1 (continue)

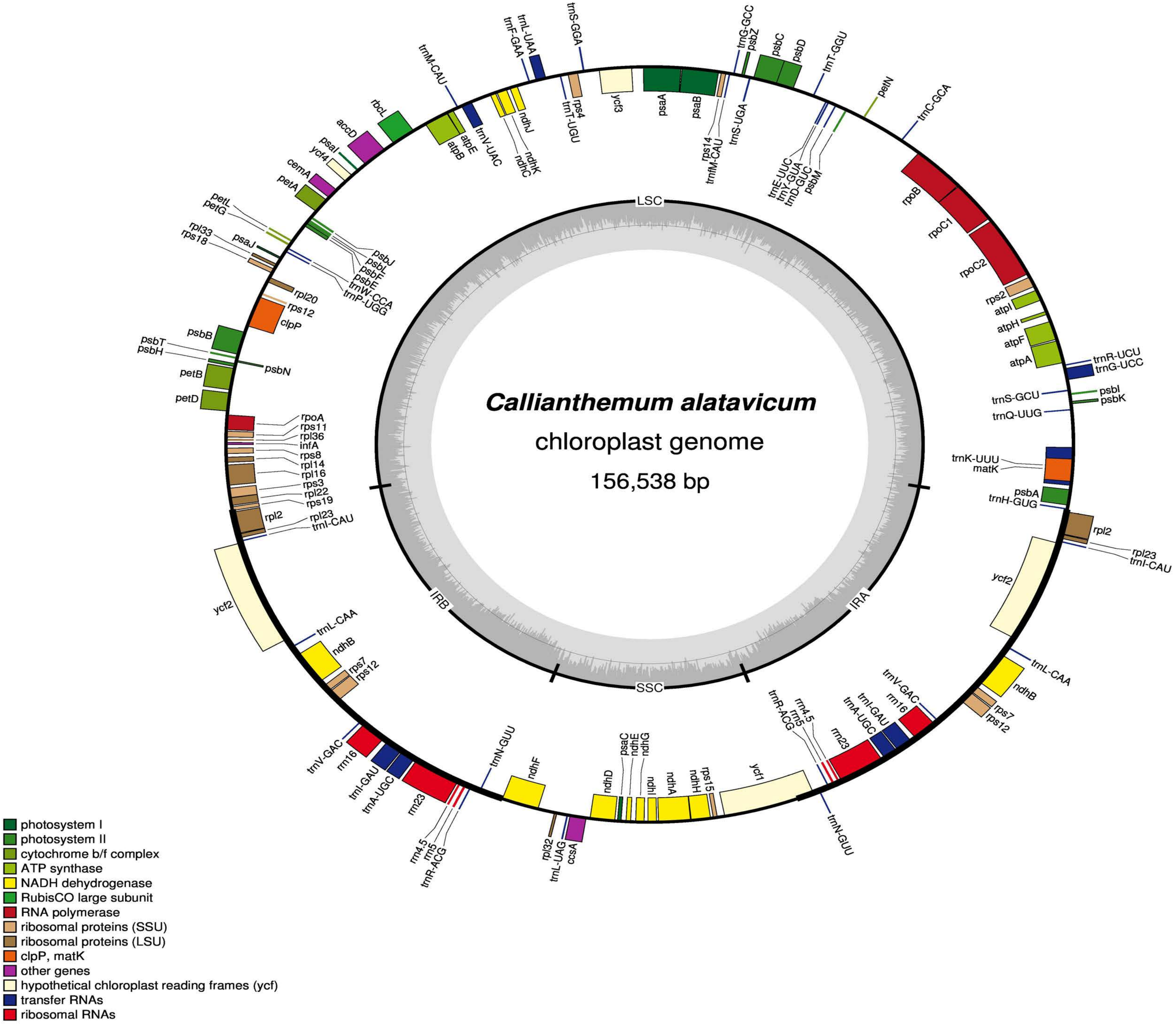

Supplementary Figure S1 (continue)

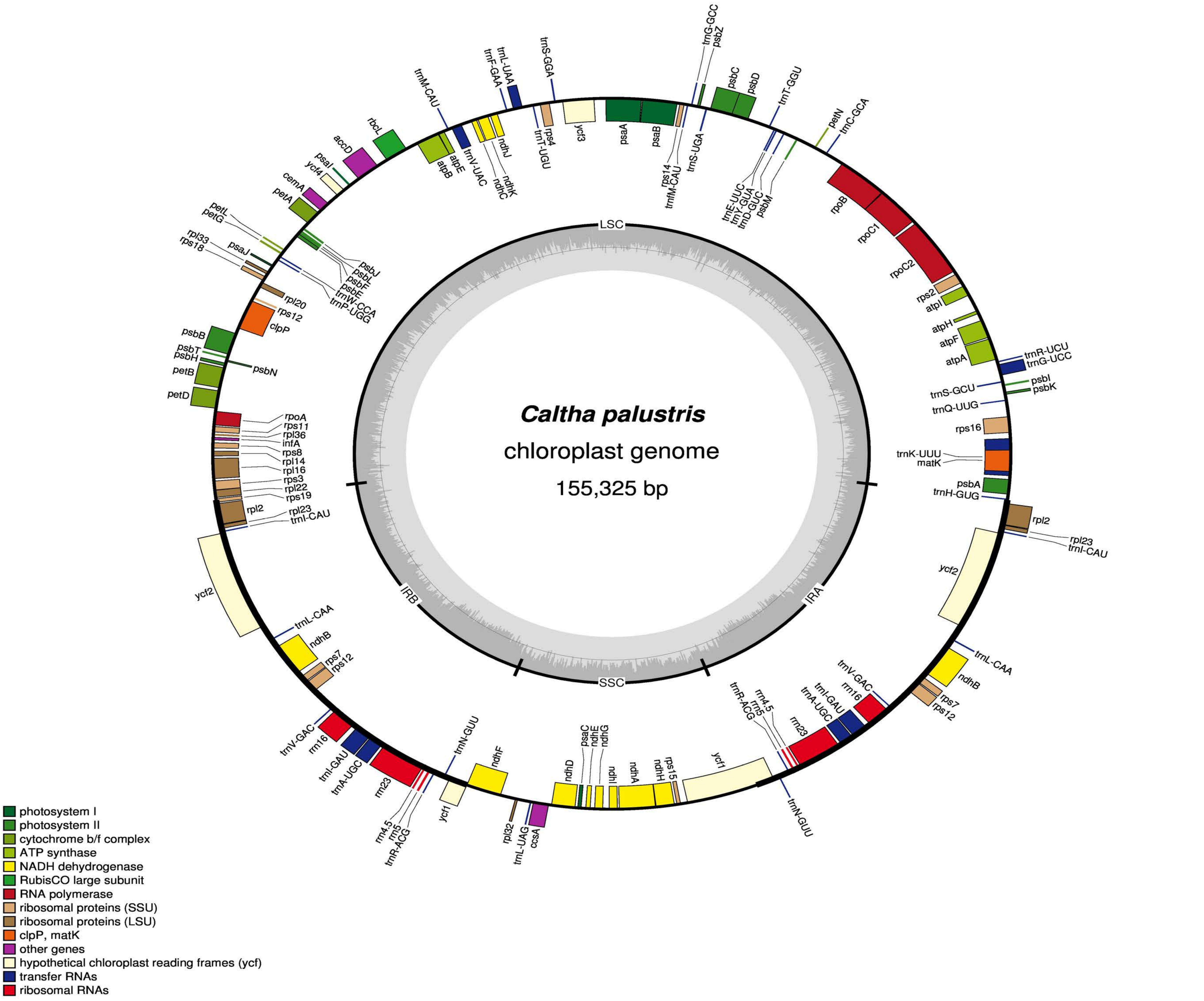

Supplementary Figure S1 (continue)

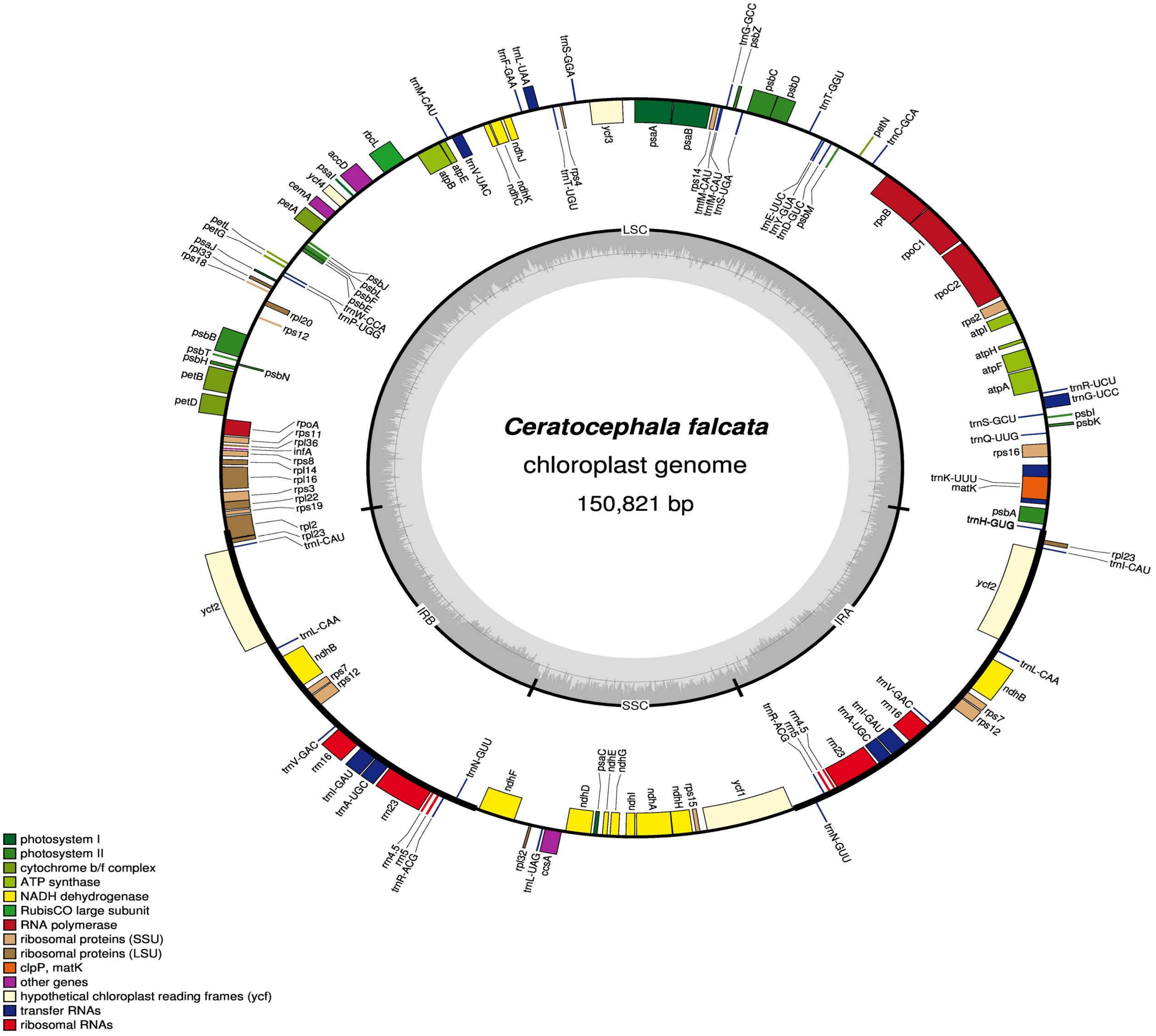

Supplement: Supplementary file 2 — Supplementary dataset [file 41598_2019_51601_MOESM2_ESM.zip › Supplementary dataset/Supplementary Figure S1.pdf]
