# Supplementary material for: Structural variation of the complete chloroplast genome and plastid phylogenomics of the genus Asteropyrum (Ranunculaceae)
Source: Sci Rep. 2019 Oct 25;9:15285. doi: 10.1038/s41598-019-51601-2 (PMC6814708; doi:10.1038/s41598-019-51601-2)
Supplement: Supplementary file 2 — Supplementary dataset [file 41598_2019_51601_MOESM2_ESM.zip › Supplementary dataset/Supplementary Figure S4.pdf]

Supplementary Figure S4

Complete cp genome

Mrbayes

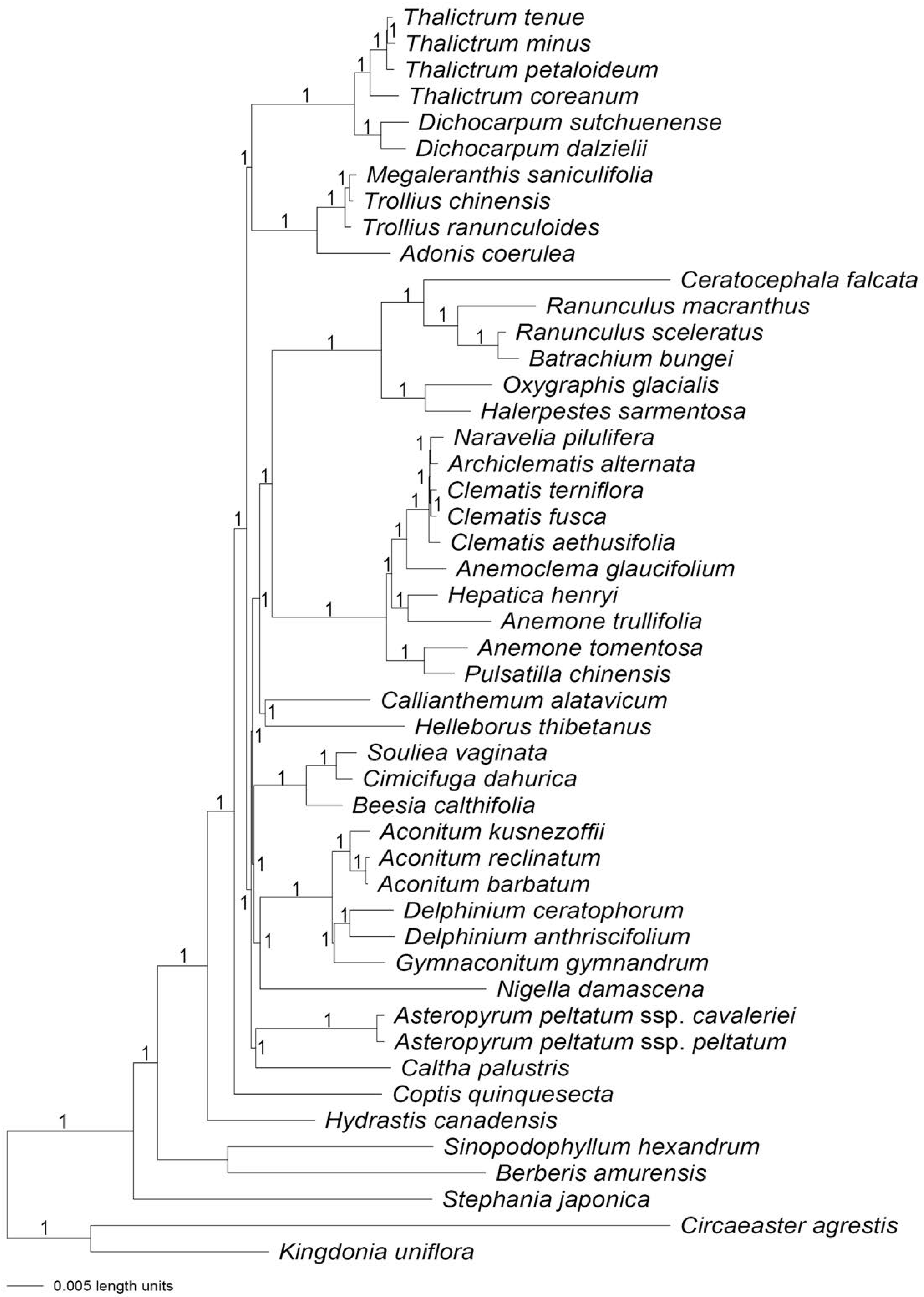

Supplementary Figure S4 (continue)

Cp CDs

Mrbayes

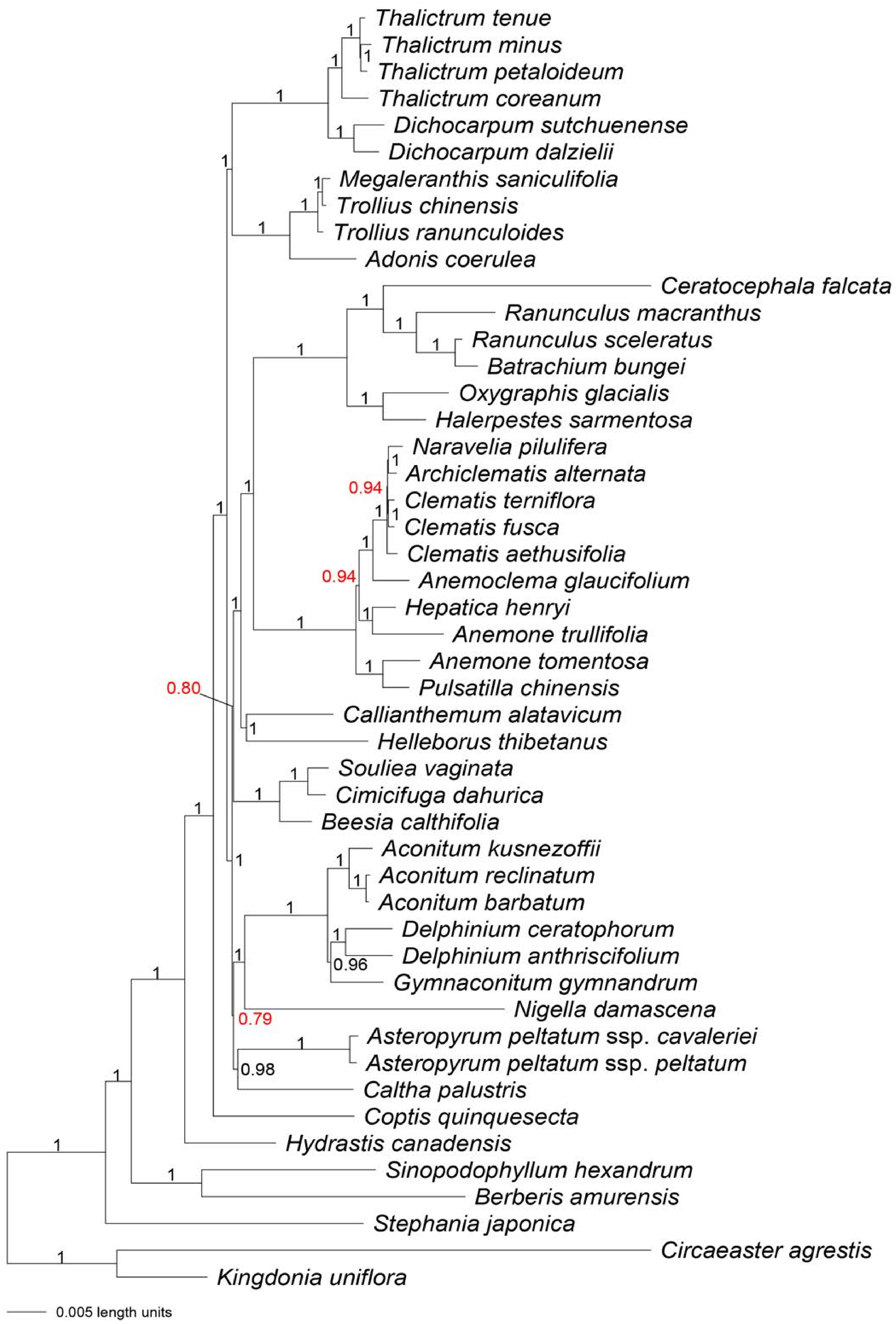

Supplementary Figure S4 (continue)

Cp IGS

Mrbayes

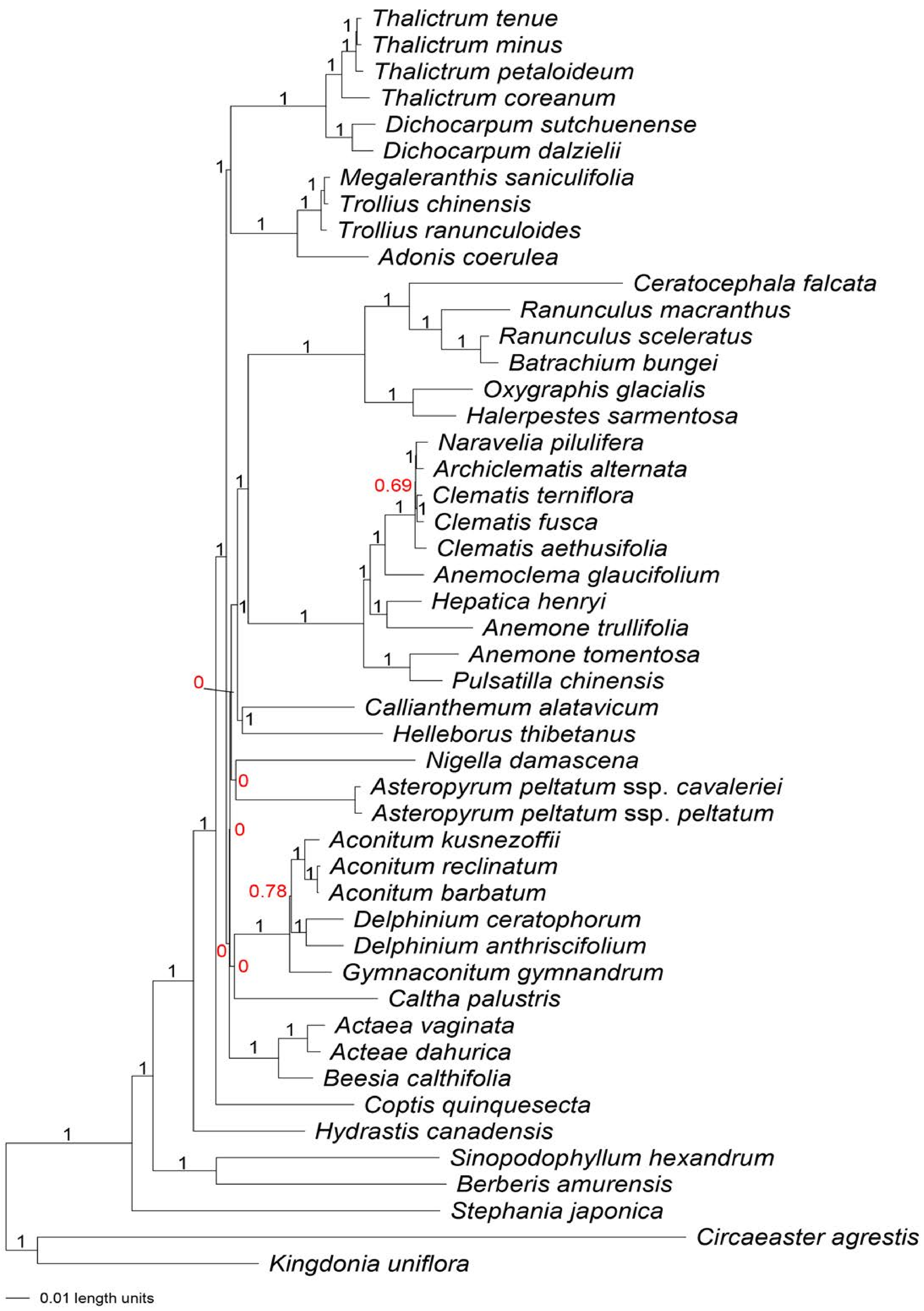

Supplementary Figure S4 (continue)

Cp Intron

Mrbayes

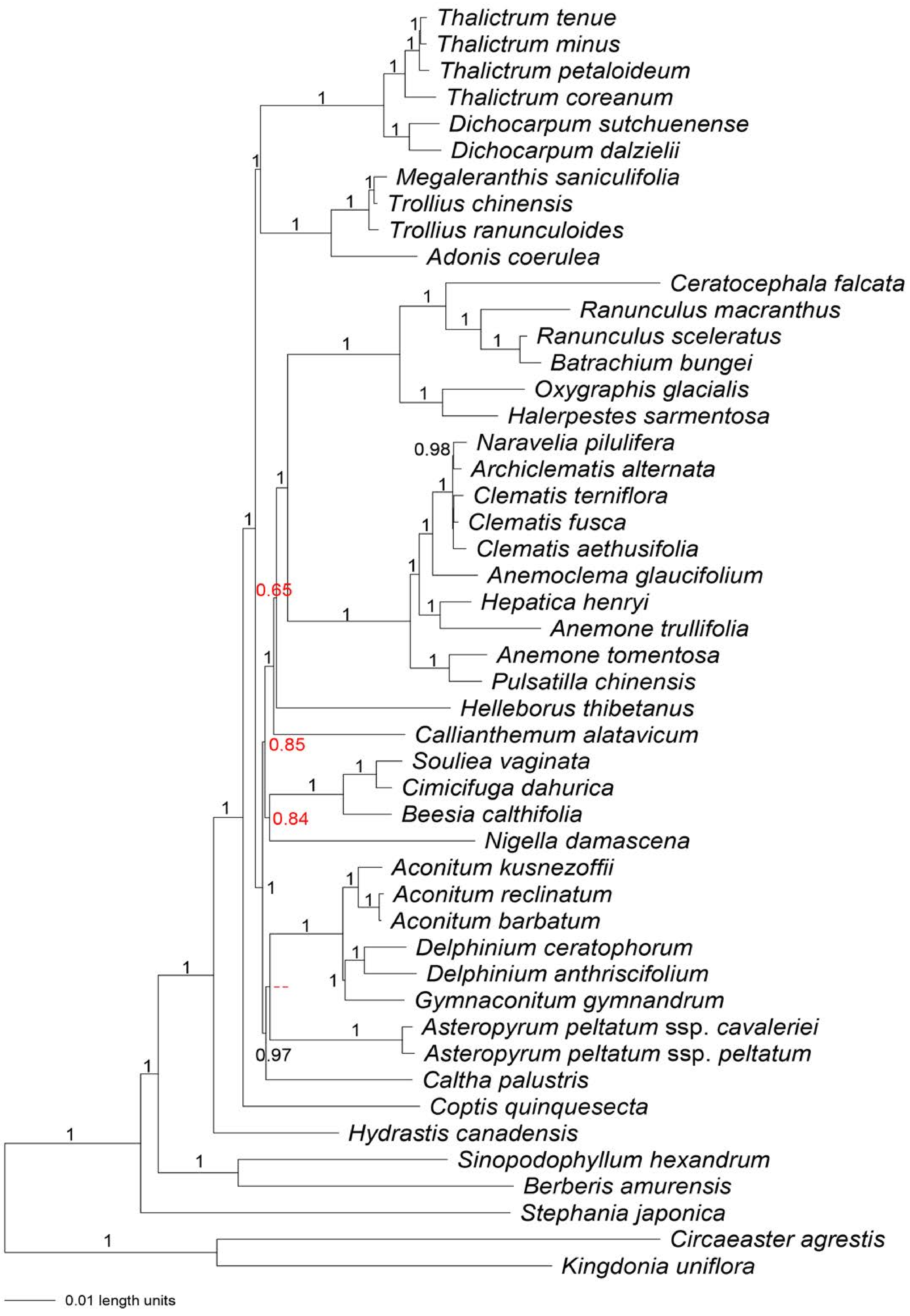

Supplementary Figure S4 (continue)

Cp LSC

Mrbayes

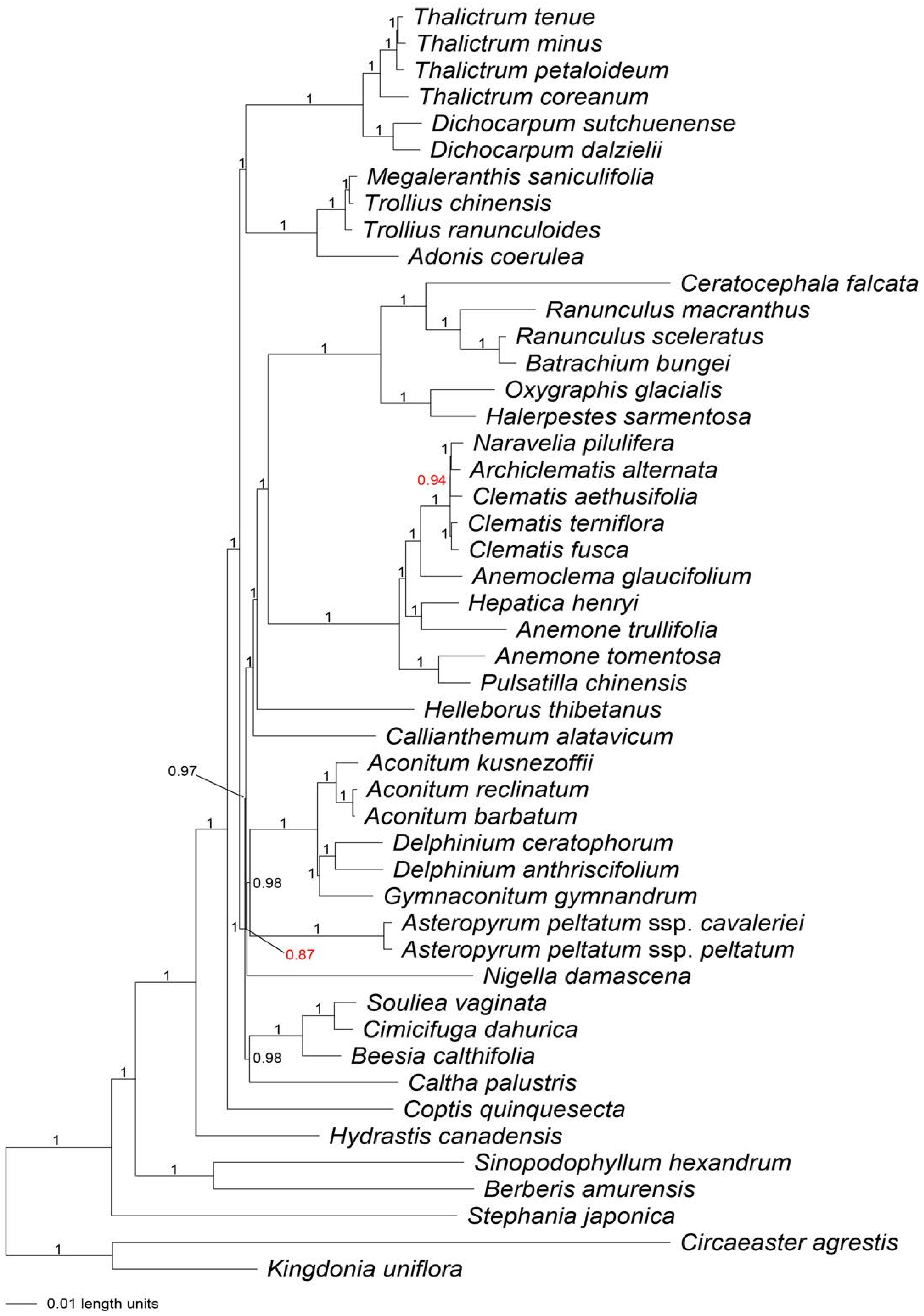

Supplementary Figure S4 (continue)

Cp SSC

Mrbayes

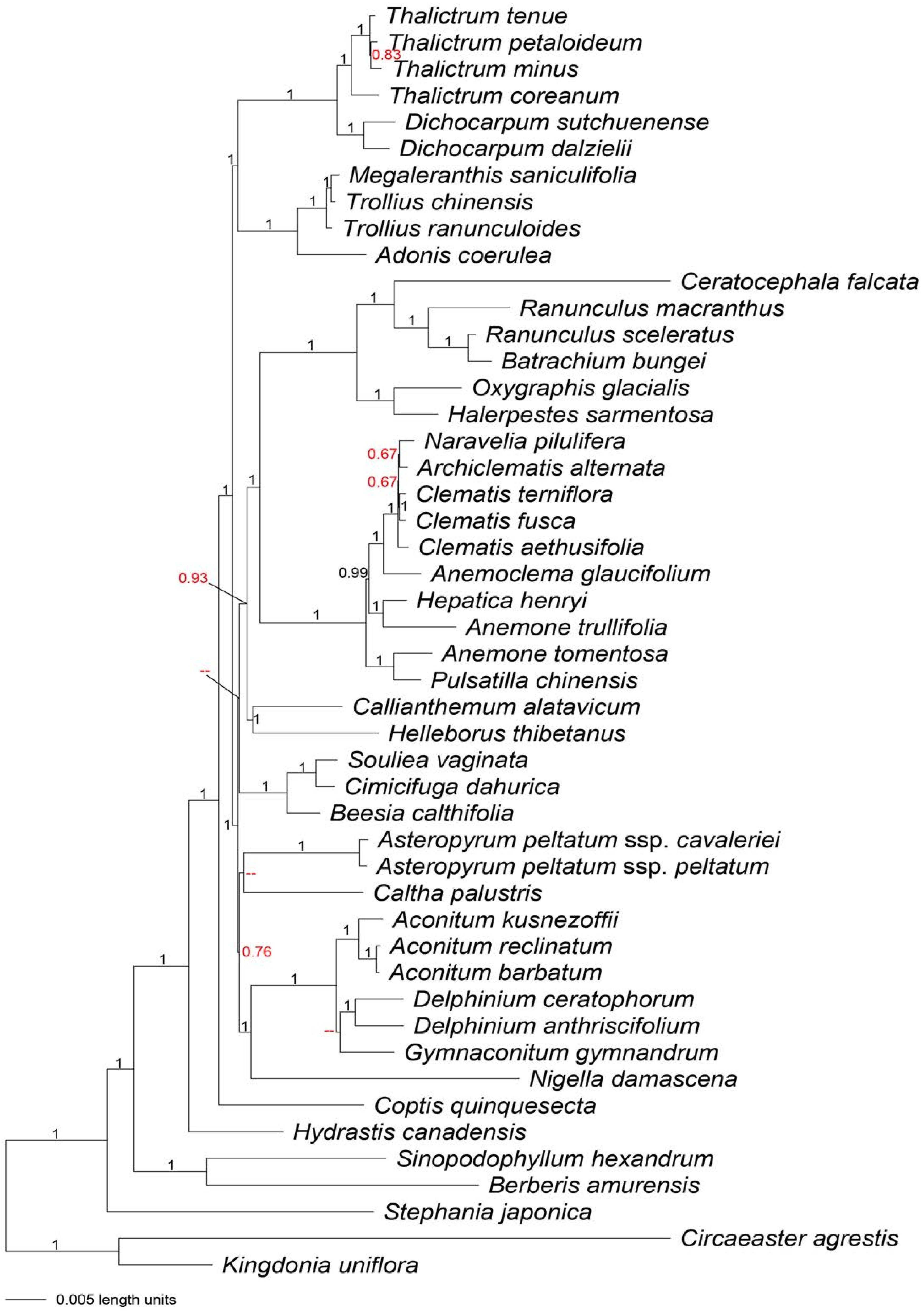

Supplementary Figure S4 (continue)

Cp IR

Mrbayes

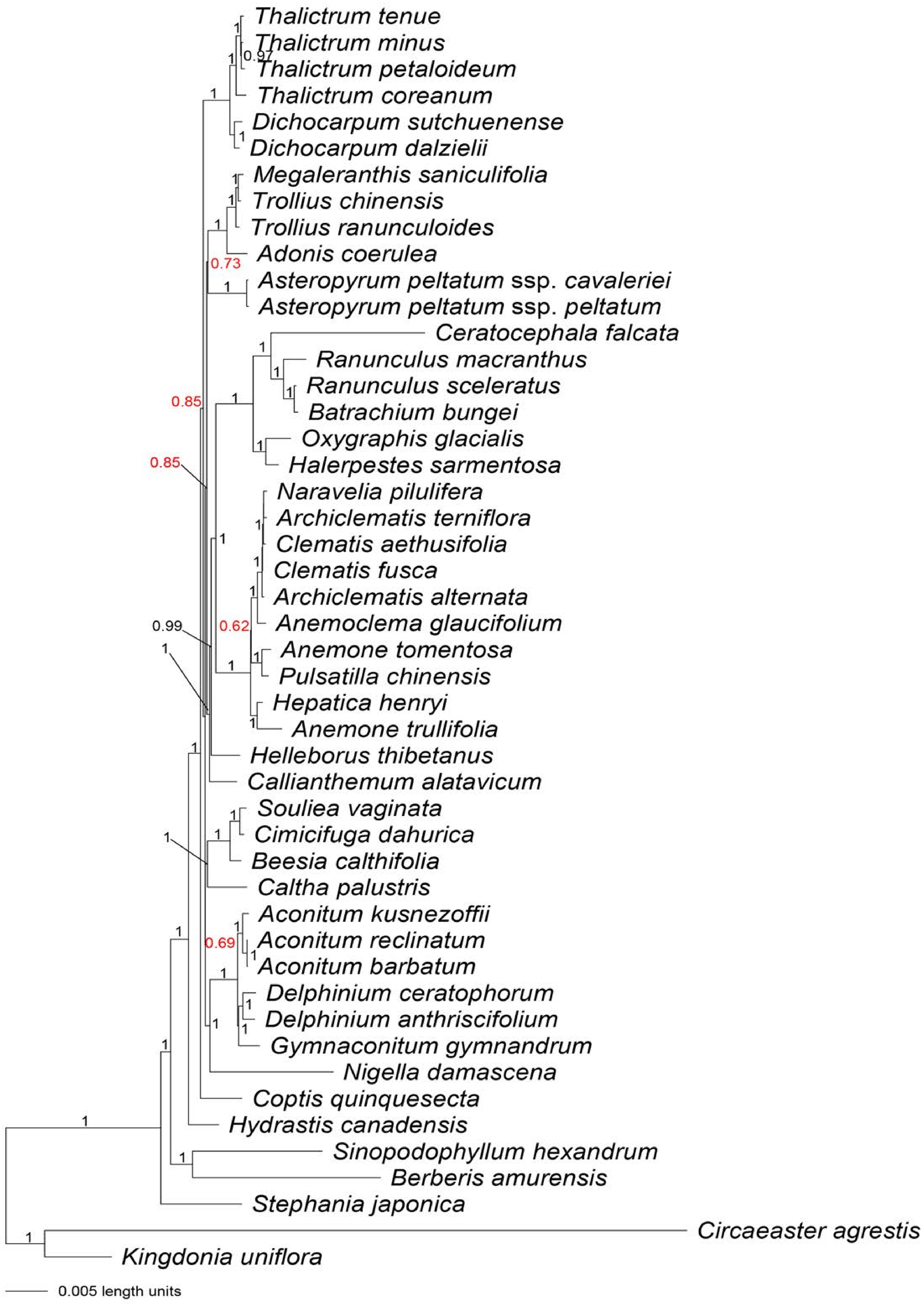

Supplementary Figure S4 (continue)

Complete cp genome

Raxml

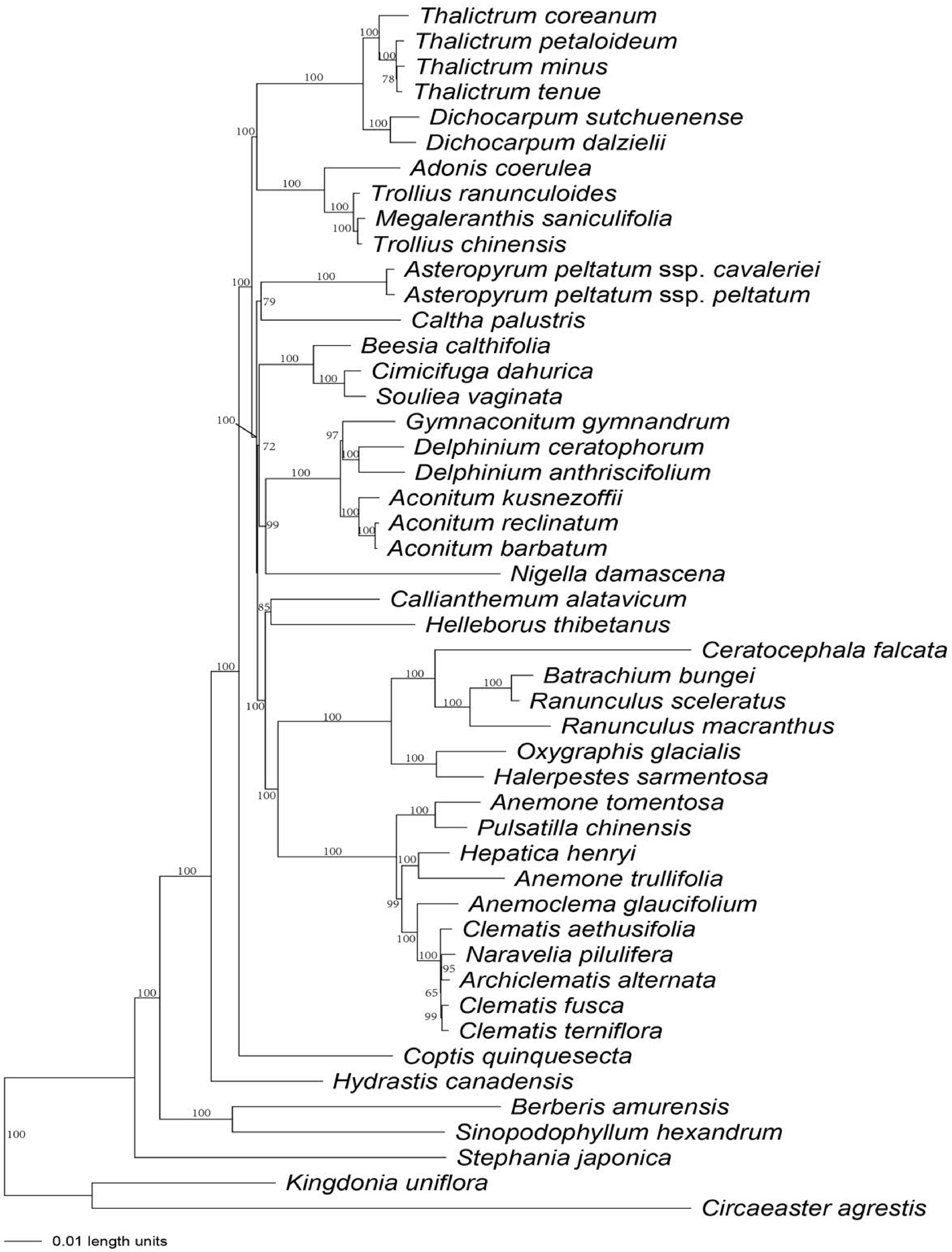

Supplementary Figure S4 (continue)

Cp CDs

Raxml

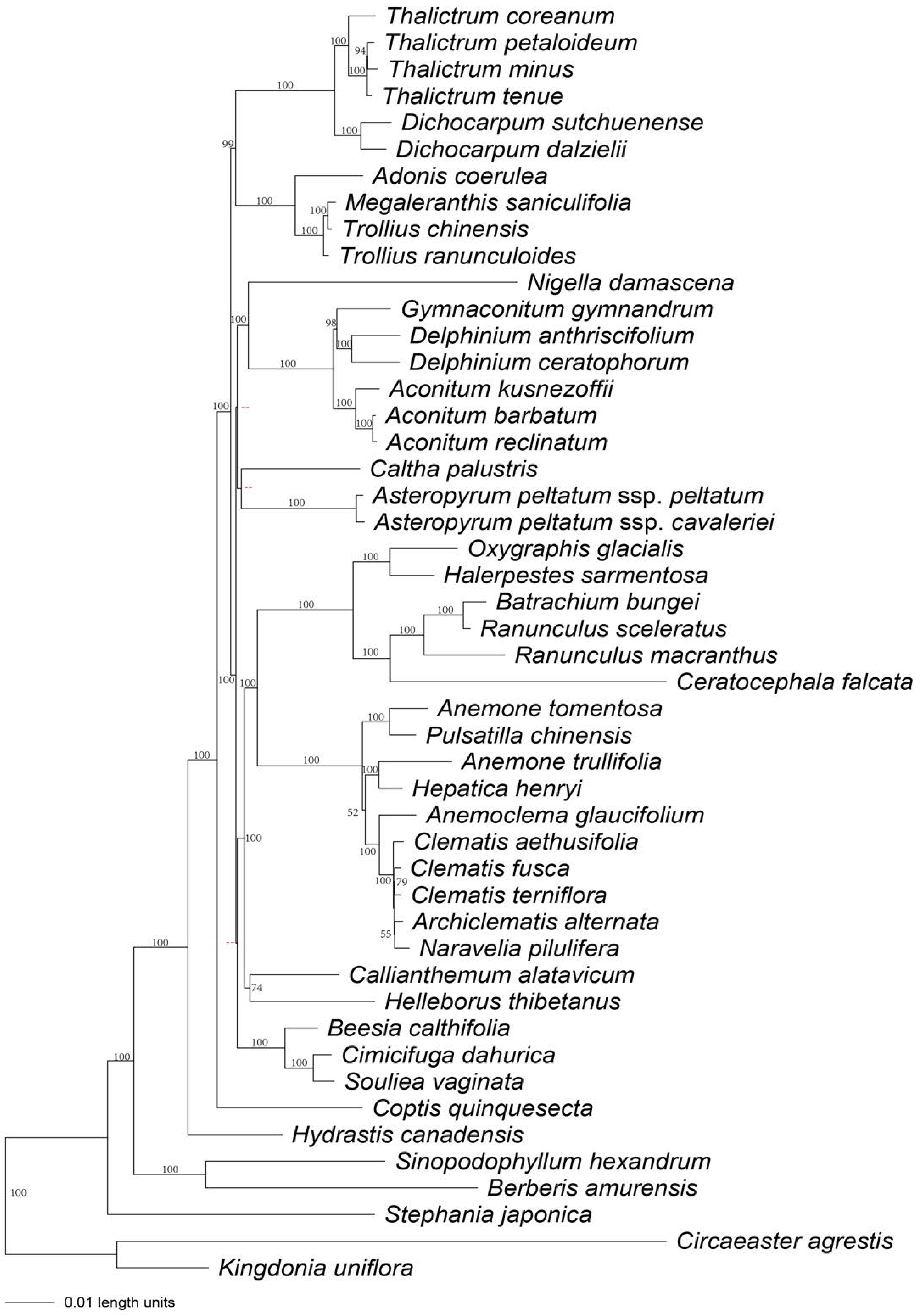

Supplementary Figure S4 (continue)

Cp IGS

Raxml

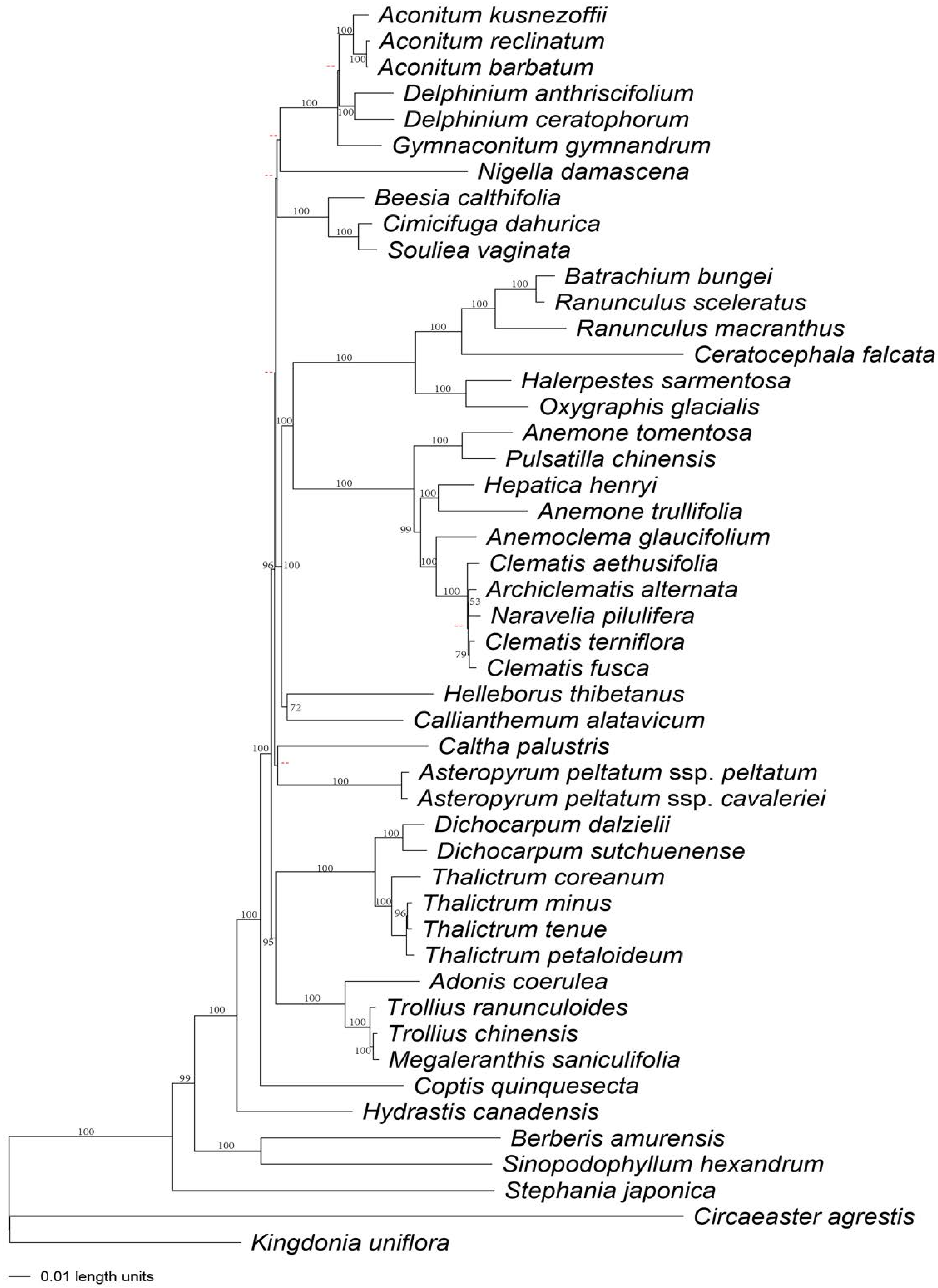

Supplementary Figure S4 (continue)

Cp Intron

Raxml

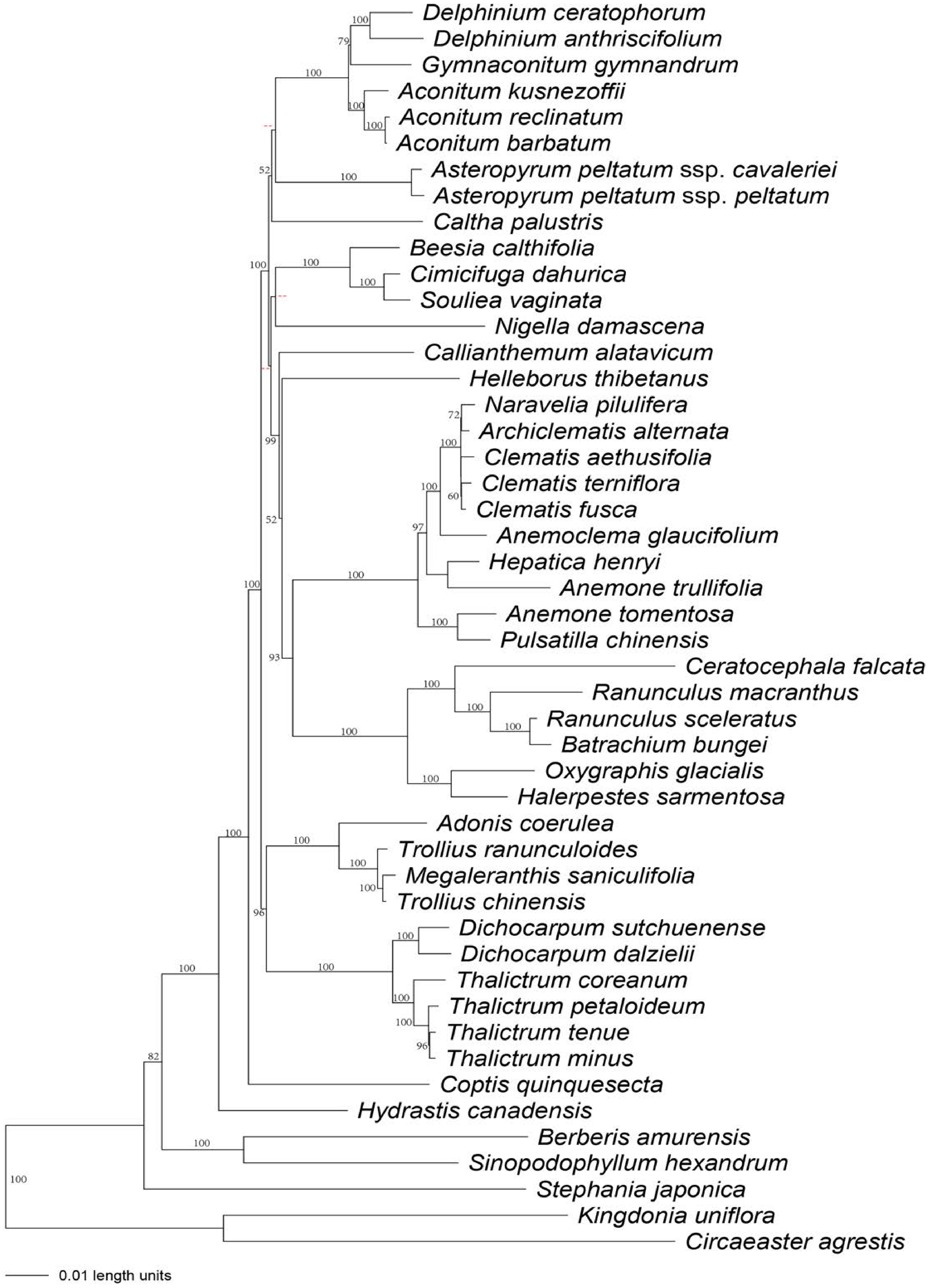

Supplementary Figure S4 (continue)

Cp LSC

Raxml

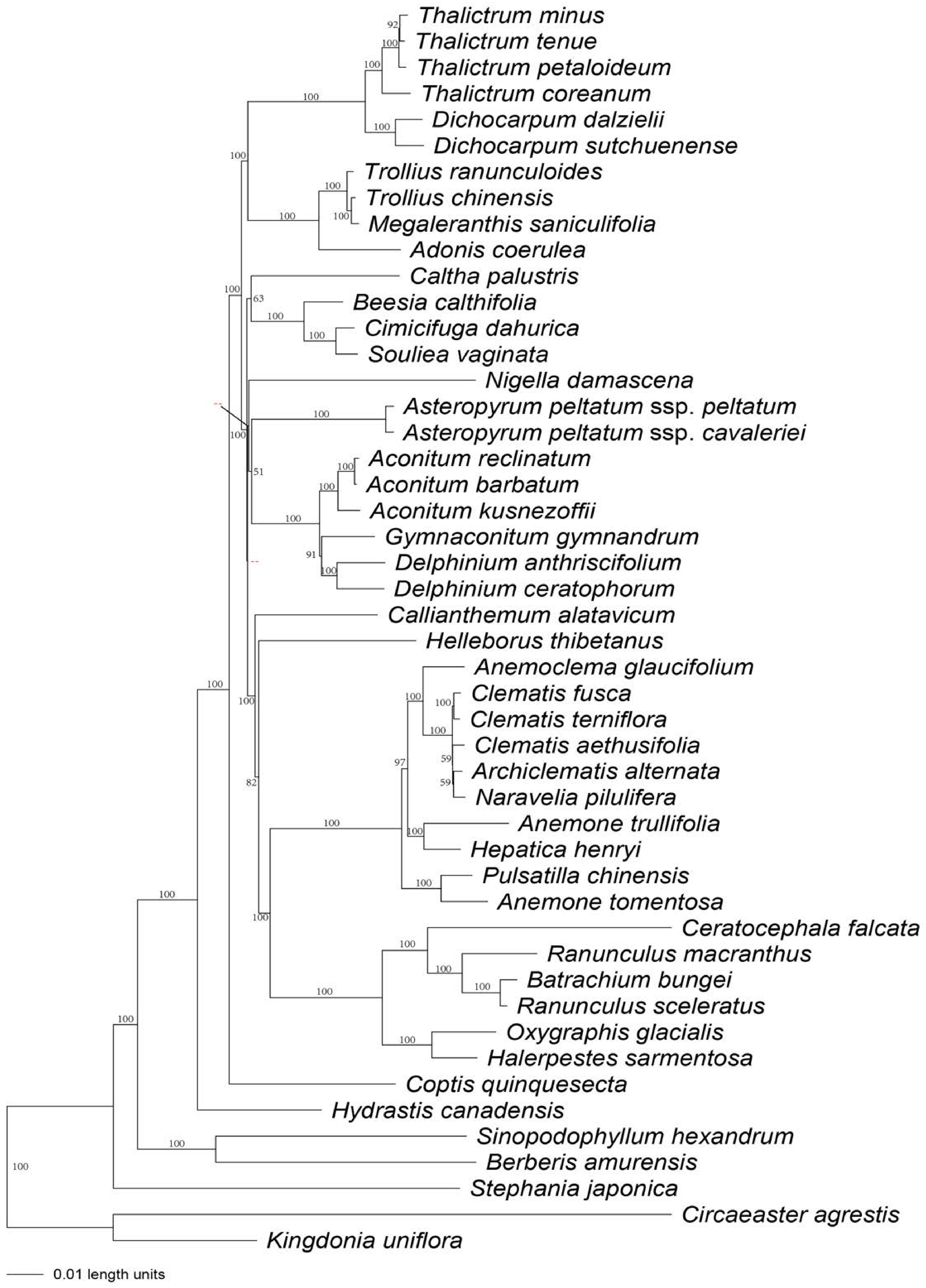

Supplementary Figure S4 (continue)

Cp SSC

Raxml

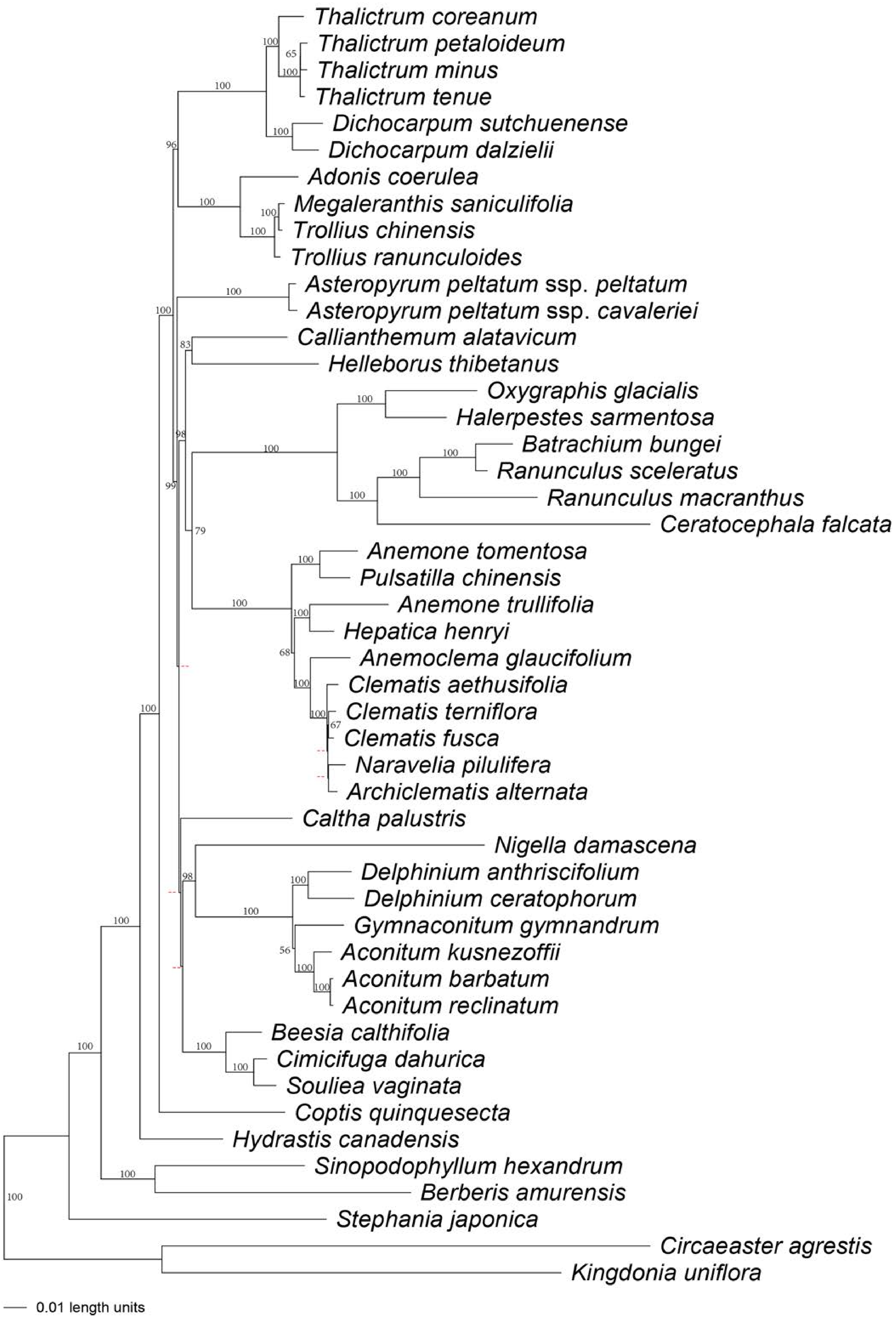

Supplementary Figure S4 (continue)

Cp IR

Raxml

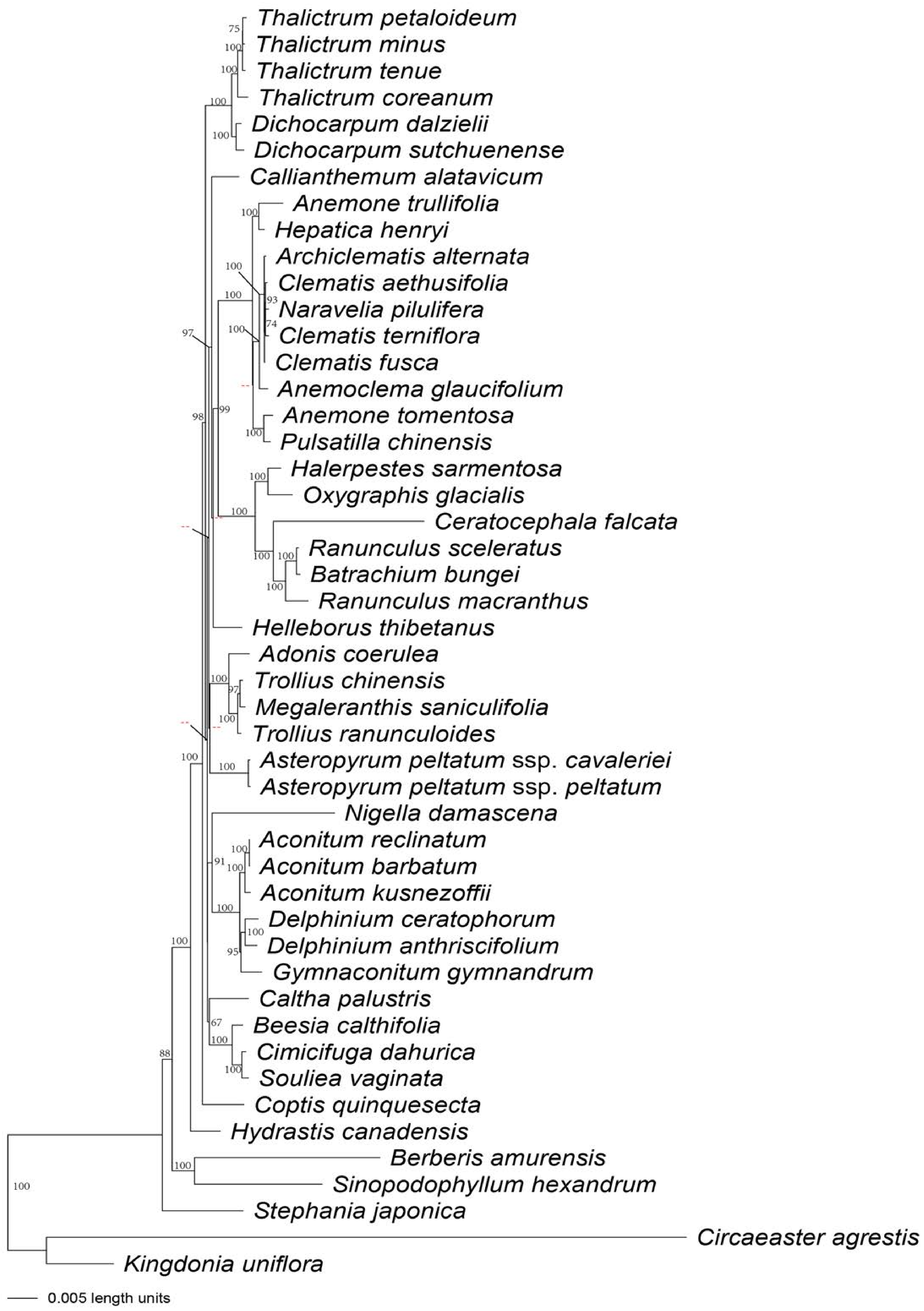

Supplementary Figure S4 (continue)

Complete cp genome

Paup-MP

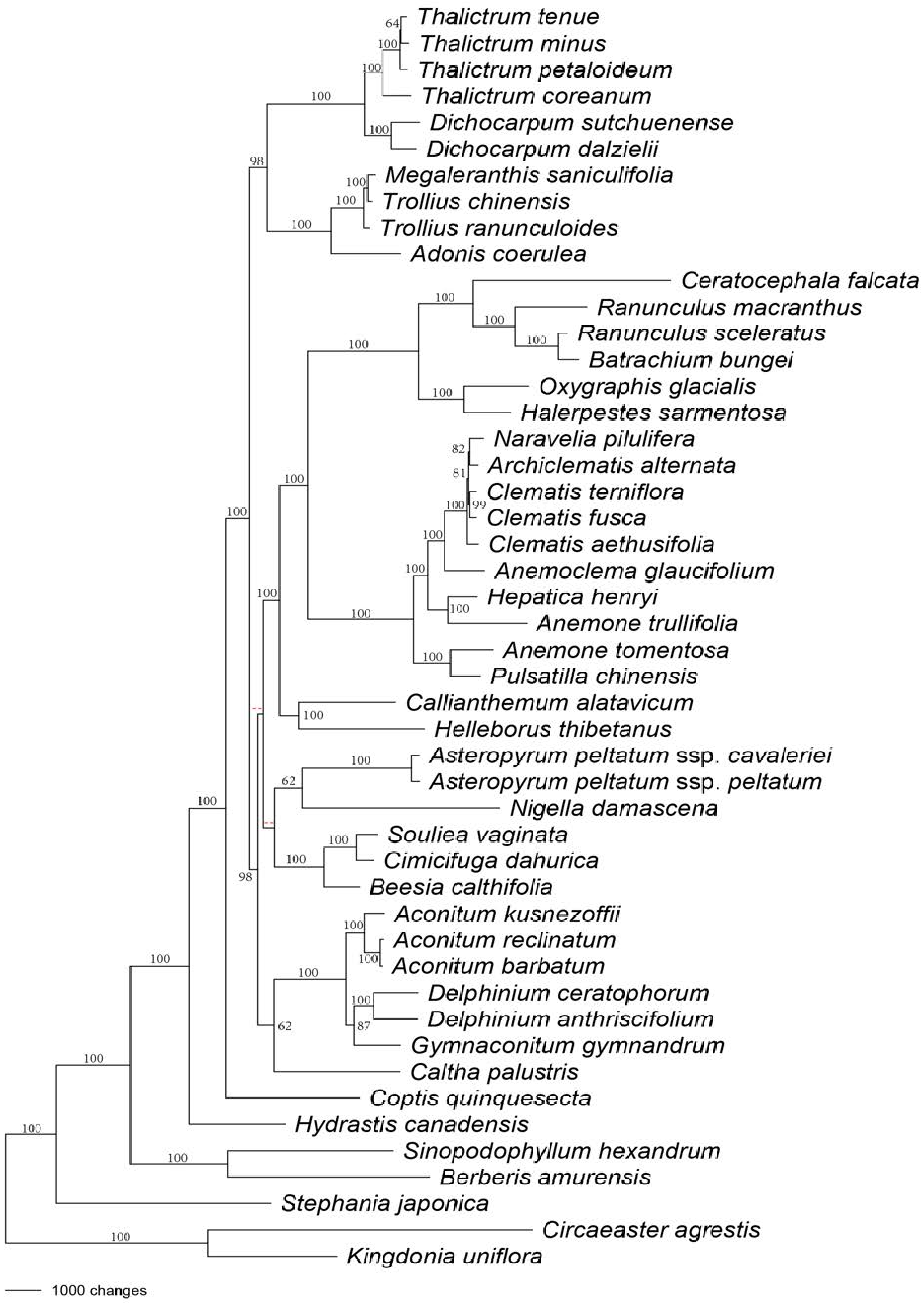

Of 120181 total characters:
All characters are of type 'unord'
All characters have equal weight
67209 characters are constant (proportion = 0.559231)
19708 variable characters are parsimony-uninformative
Number of parsimony-informative characters = 33264

Tree length = 132930
Consistency index (CI) = 0.5860
Homoplasy index (HI) = 0.4140
CI excluding uninformative characters = 0.4976
HI excluding uninformative characters = 0.5024
Retention index (RI) = 0.6825
Rescaled consistency index (RC) = 0.4000

Supplementary Figure S4 (continue)

Cp CDs

Paup-MP

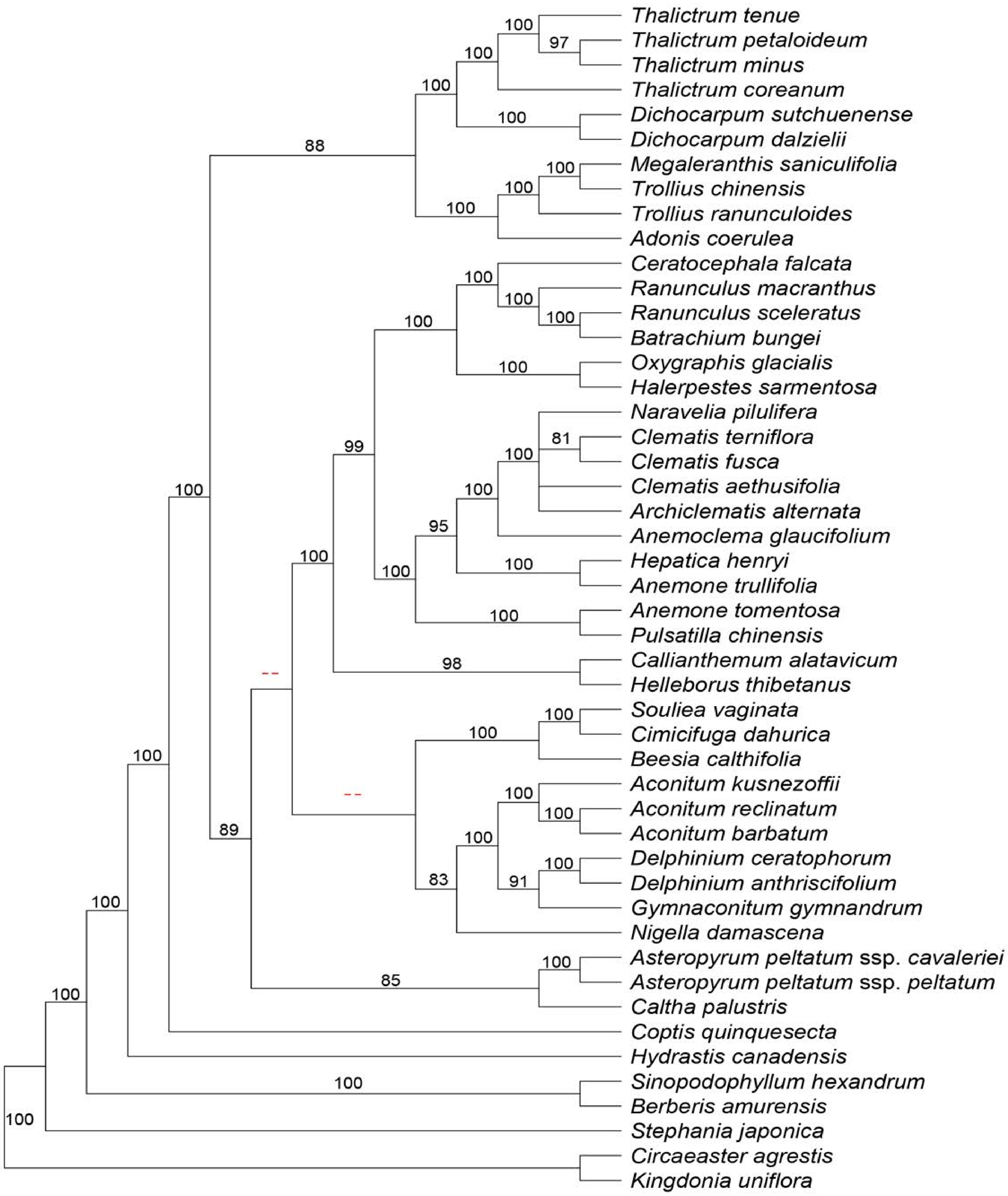

Of 74772 total characters:
All characters are of type 'unord'
All characters have equal weight
50031 characters are constant (proportion = 0.669114)
9537 variable characters are parsimony-uninformative
Number of parsimony-informative characters = 15204

Tree length = 58454
Consistency index (CI) = 0.5803
Homoplasy index (HI) = 0.4197
CI excluding uninformative characters = 0.4839
HI excluding uninformative characters = 0.5161
Retention index (RI) = 0.6834
Rescaled consistency index (RC) = 0.3966
2 trees

Supplementary Figure S4 (continue)

Cp IGS

Paup-MP

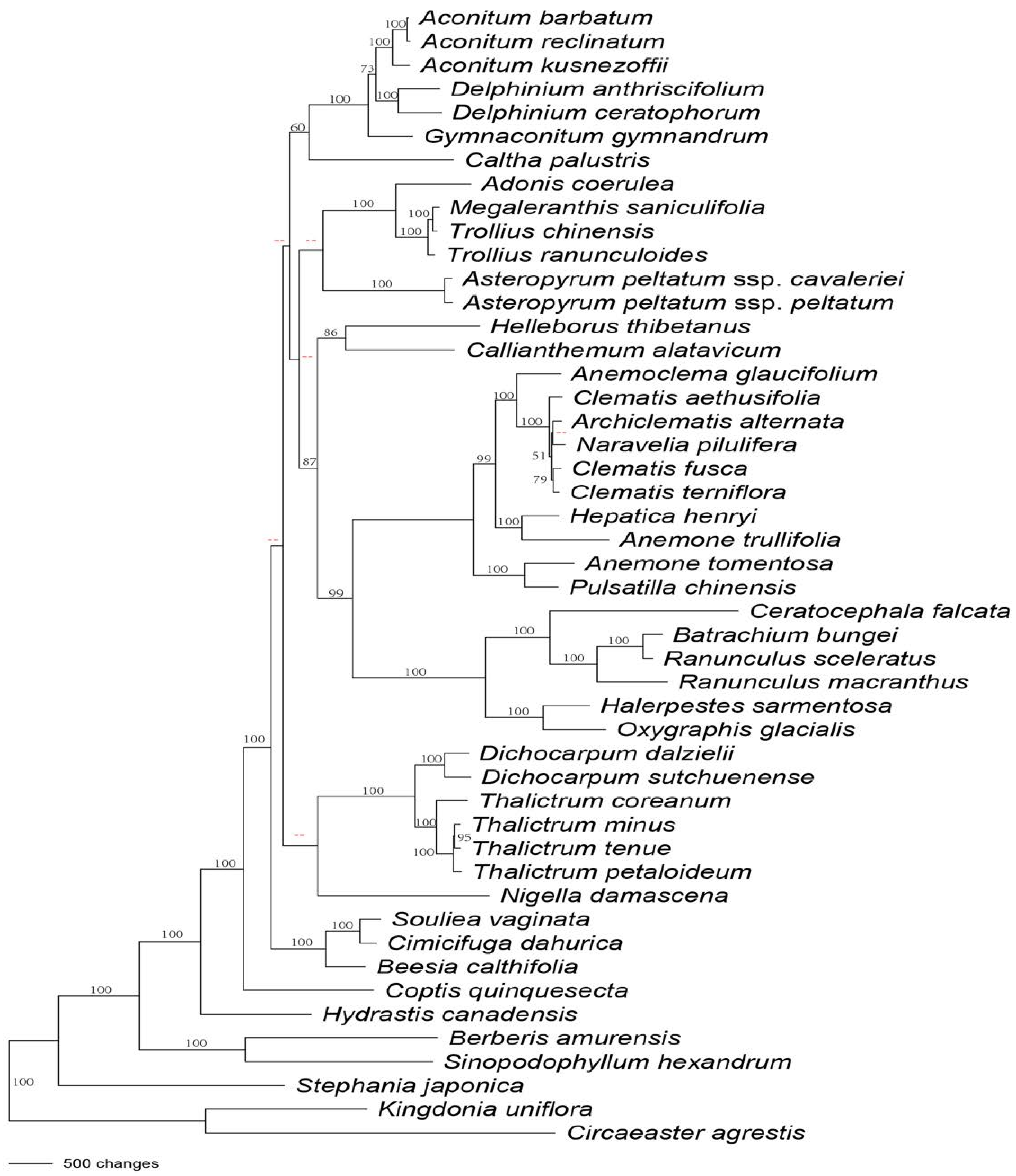

Of 31036 total characters:
All characters are of type 'unord'
All characters have equal weight
9560 characters are constant (proportion = 0.308029)
7395 variable characters are parsimony-uninformative
Number of parsimony-informative characters = 14081

Tree length = 58731
Consistency index (CI) = 0.5841
Homoplasy index (HI) = 0.4159
CI excluding uninformative characters = 0.5066
HI excluding uninformative characters = 0.4934
Retention index (RI) = 0.6781
Rescaled consistency index (RC) = 0.3961

Supplementary Figure S4 (continue)

Cp Intron

Paup-MP

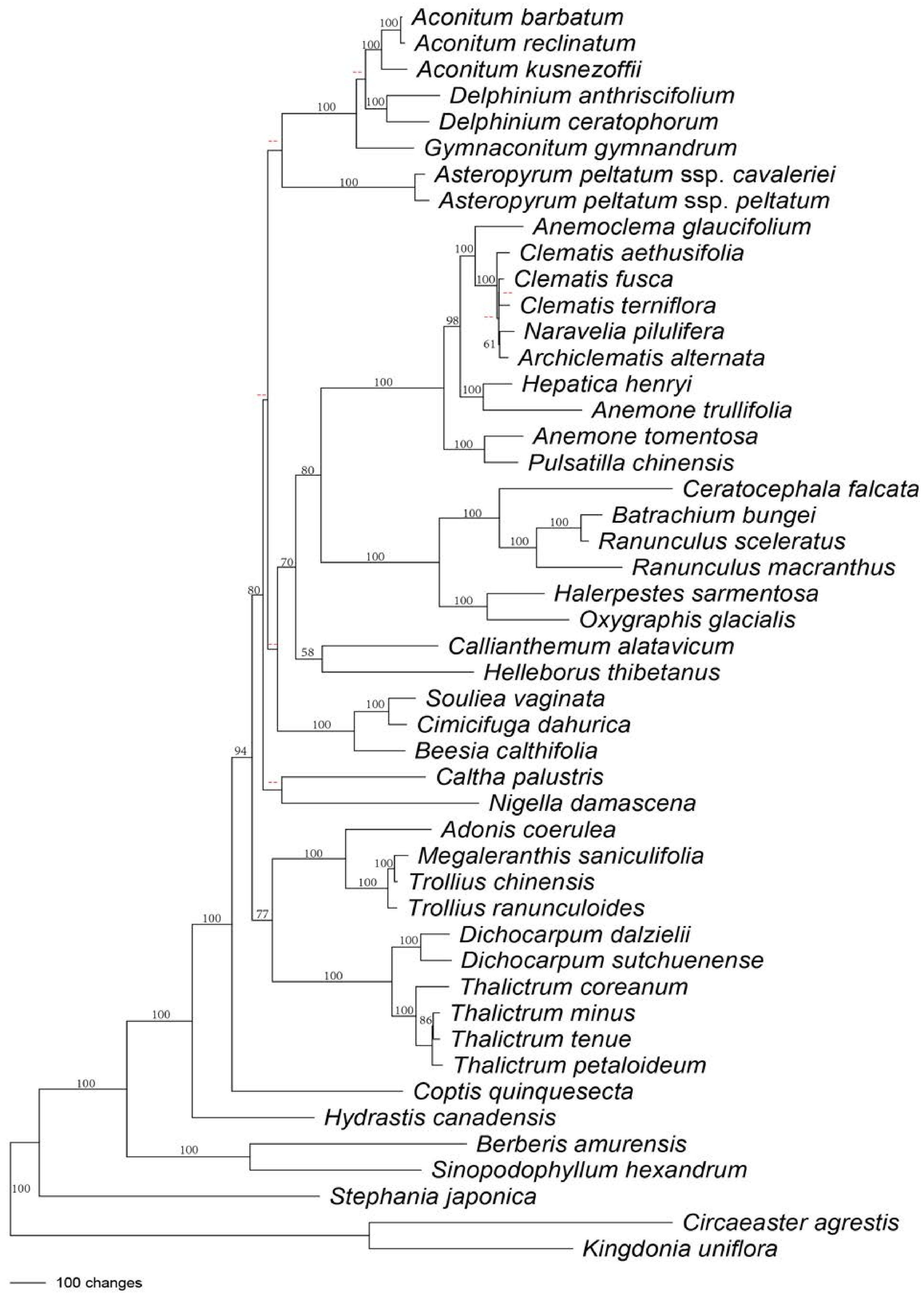

Of 14373 total characters:
All characters are of type 'unord'
All characters have equal weight
7618 characters are constant (proportion = 0.530022)
2776 variable characters are parsimony-uninformative
Number of parsimony-informative characters = 3979

Tree length = 15648
Consistency index (CI) = 0.6182
Homoplasy index (HI) = 0.3818
CI excluding uninformative characters = 0.5181
HI excluding uninformative characters = 0.4819
Retention index (RI) = 0.7007
Rescaled consistency index (RC) = 0.4332

Supplementary Figure S4 (continue)

Cp LSC

Paup-MP

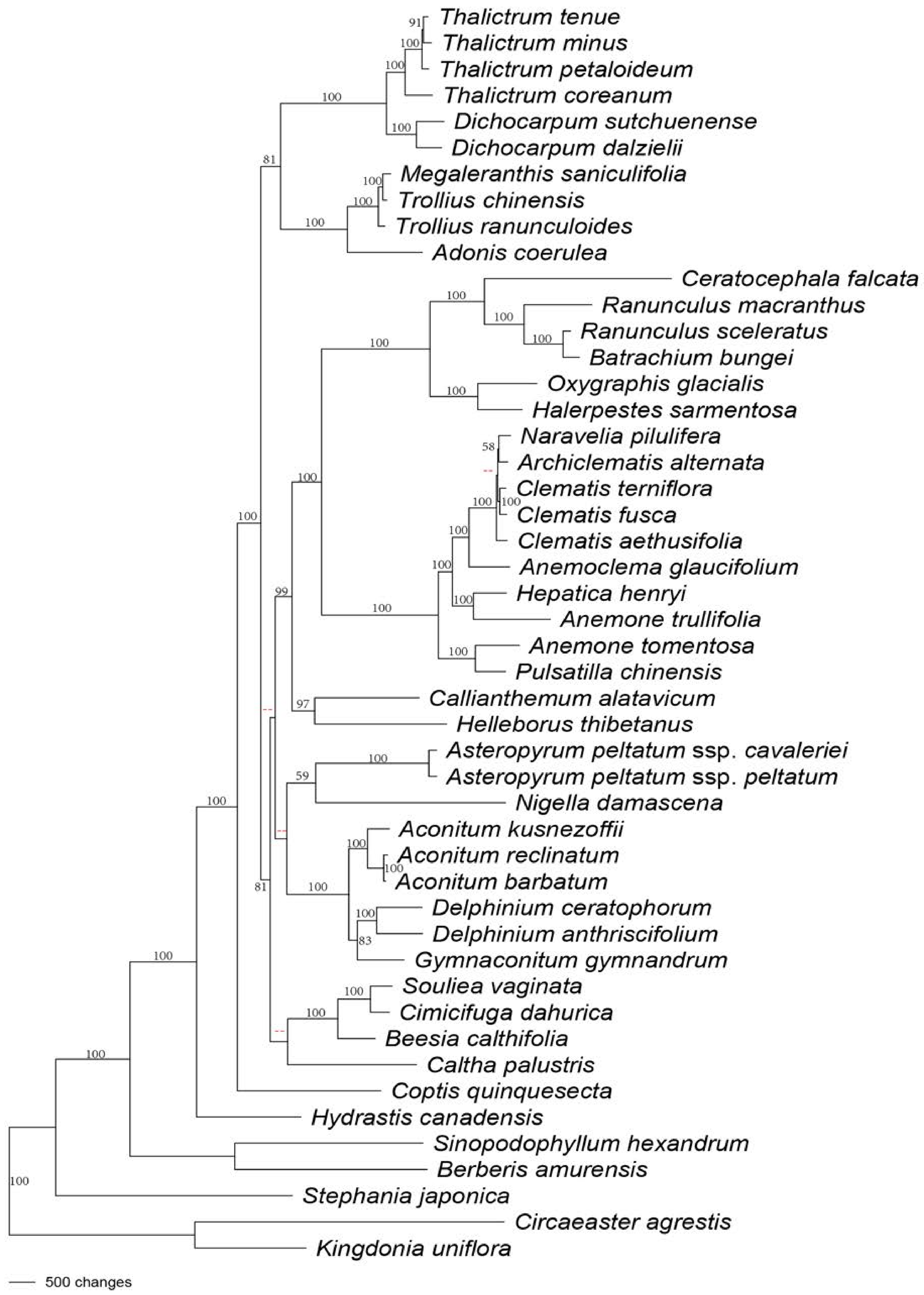

Of 77968 total characters:
All characters are of type 'unord'
All characters have equal weight
41042 characters are constant (proportion = 0.526395)
12885 variable characters are parsimony-uninformative
Number of parsimony-informative characters = 24041

Tree length = 93269
Consistency index (CI) = 0.5850
Homoplasy index (HI) = 0.4150
CI excluding uninformative characters = 0.5027
HI excluding uninformative characters = 0.4973
Retention index (RI) = 0.6891
Rescaled consistency index (RC) = 0.4031

Supplementary Figure S4 (continue)

Cp SSC

Paup-MP

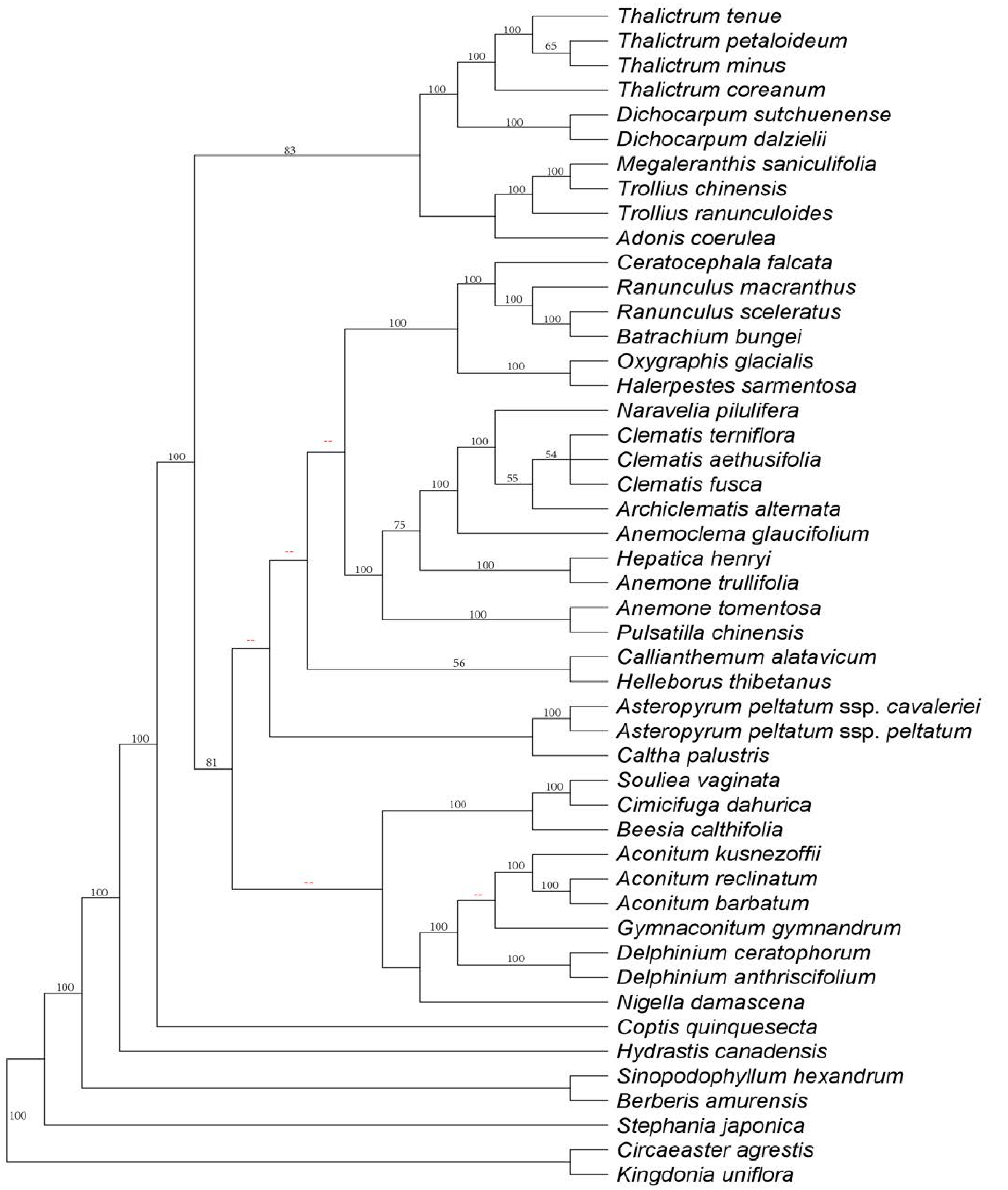

Of 18551 total characters:
All characters are of type 'unord'
All characters have equal weight
7521 characters are constant (proportion = 0.405423)
3385 variable characters are parsimony-uninformative
Number of parsimony-informative characters = 7645

Tree length = 32922
Consistency index (CI) = 0.5409
Homoplasy index (HI) = 0.4591
CI excluding uninformative characters = 0.4728
HI excluding uninformative characters = 0.5272
Retention index (RI) = 0.6496
Rescaled consistency index (RC) = 0.3514
2 trees

Supplementary Figure S4 (continue)

Cp IR

Paup-MP

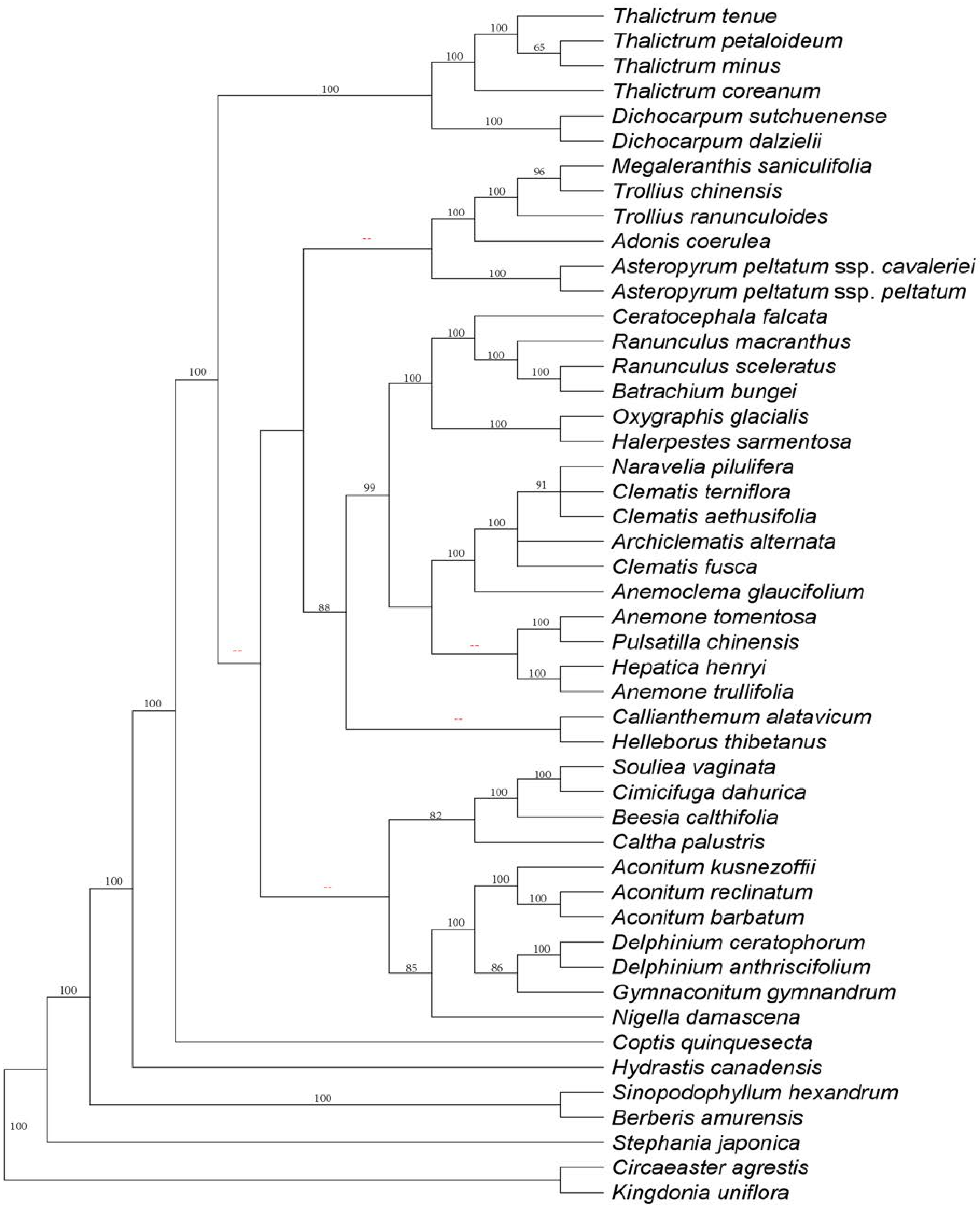

Of 24318 total characters:
All characters are of type 'unord'
All characters have equal weight
18605 characters are constant (proportion = 0.765071)
3647 variable characters are parsimony-uninformative
Number of parsimony-informative characters = 2066

Tree length = 8245
Consistency index (CI) = 0.8018
Homoplasy index (HI) = 0.1982
CI excluding uninformative characters = 0.6168
HI excluding uninformative characters = 0.3832
Retention index (RI) = 0.7851
Rescaled consistency index (RC) = 0.6295
3 trees
