# Supplementary material for: Structural variation of the complete chloroplast genome and plastid phylogenomics of the genus Asteropyrum (Ranunculaceae)
Source: Sci Rep. 2019 Oct 25;9:15285. doi: 10.1038/s41598-019-51601-2 (PMC6814708; doi:10.1038/s41598-019-51601-2)
Supplement: Supplementary file 2 — Supplementary dataset [file 41598_2019_51601_MOESM2_ESM.zip › Supplementary dataset/Supplementary Table S1.docx]

**Supplementary Table S1** Information regarding plastid genome sequence assembly for all newly sequenced Ranunculaceae species.

| **Species** | **Clean data (Gb)** | **Clean reads** | **Filtered cp reads** | **Contigs before iteration map** | **Contigs after iteration map** | **Length of contigs** | **PCR primer for gap filling** |
| --- | --- | --- | --- | --- | --- | --- | --- |
| *Aconitum barbatum* | 2.3 | 6344204 | 51642 | 2 | 2 | 69256+61414 | 20abF:TACCGAACTGAACTAAGAGCGC; 20abR:AGTCAAGAACAAAACAAAGAAGGGG |
| *Aconitum kuznezoffii* | 3.2 | 8826707 | 72148 | 2 | 1 | 129650 |  |
| *Adonis coerulea* | 2.3 | 6349523 | 51914 | 4 | 2 | 47911+82932 | 102rps16lF:CCCTGGAGACGCCCTTGTAT; 102rps16lR:GGGACGAGCTTGCTTCTTGA |
| *Batrachium bungei* | 2.4 | 6619957 | 54112 | 5 | 1 | 130830 |  |
| *Beesia calthifolia* | 3.1 | 8556436 | 69987 | 4 | 2 | 54030+76587 | 103psblcF:AGAAGAAATAATACTCTACCCCGATCT; 103psblcR:ATCTCTTTCACCAGGCCTCG |
| *Callianthemum alatavicum* | 5.6 | 15449964 | 125266 | 3 | 1 | 130660 |  |
| *Caltha palustris* | 2.6 | 7177618 | 58442 | 5 | 2 | 6017+122672 | 46trnQKF:CCAAAACCCGTTGCCTTACC; 46trnQKR:GGCTAAGAGCAAAAAGAACAAAGG |
| *Ceratocephala falcata* | 3 | 8271707 | 66872 | 6 | 1 | 126756 |  |
| *Cimicifuga dahurica* | 2.4 | 6622797 | 53583 | 4 | 1 | 132890 |  |
| *Clematis aethusifolia* | 2.8 | 7720067 | 62559 | 1 | 1 | 128657 |  |
| *Delphinium ceratophorum* | 2.7 | 7444709 | 60392 | 2 | 1 | 127785 |  |
| *Delphinium anthriscifolium* | 3.5 | 9656901 | 78466 | 1 | 1 | 129200 |  |
| *Dichocarpum dalzielii* | 2.1 | 5788694 | 47418 | 2 | 1 | 126674 |  |
| *Dichocarpum sutchuenense* | 3.3 | 9105961 | 73902 | 2 | 1 | 127868 |  |
| *Halerpestes sarmentosa* | 2.4 | 6623494 | 53898 | 5 | 1 | 132342 |  |
| *Helleborus thibetanus* | 2.9 | 7999831 | 65434 | 1 | 1 | 130045 |  |
| *Naravelia pilulifera* | 3 | 8272986 | 67427 | 1 | 1 | 128559 |  |
| *Nigella_damascena* | 2.2 | 6068894 | 49725 | 2 | 1 | 130155 |  |
| *Oxygraphis glacialis* | 6.2 | 17104519 | 139004 | 2 | 1 | 131509 |  |
| *Ranunculus sceleratus* | 3.6 | 9930175 | 80925 | 4 | 1 | 131122 |  |
| *Souliea vaginata* | 3.1 | 8548566 | 69272 | 2 | 1 | 131924 |  |
| *Thalictrum minus* | 2.3 | 6343047 | 51845 | 3 | 1 | 129819 |  |
| *Thalictrum petaloideum* | 3.2 | 8832639 | 71601 | 2 | 1 | 129496 |  |
| *Thalictrum tenue* | 3.2 | 8825760 | 71851 | 2 | 1 | 129699 |  |
| *Trollius ranunculoides* | 2.3 | 6343823 | 51663 | 3 | 1 | 133266 |  |
