# Supplementary material for: Structural variation of the complete chloroplast genome and plastid phylogenomics of the genus Asteropyrum (Ranunculaceae)
Source: Sci Rep. 2019 Oct 25;9:15285. doi: 10.1038/s41598-019-51601-2 (PMC6814708; doi:10.1038/s41598-019-51601-2)
Supplement: Supplementary file 2 — Supplementary dataset [file 41598_2019_51601_MOESM2_ESM.zip › Supplementary dataset/Supplementary Table S2.docx]

**Supplementary Table S2** Summary of the plastome features of newly sequenced Ranunculaceae species.

| Category | *Aconitum barbatum* | *Aconitum kuznezoffii* | *Adonis coerulea* | *Batrachium bungei* | *Beesia calthifolia* |
| --- | --- | --- | --- | --- | --- |
| Total cp genome size (bp) | 156761 | 155832 | 157033 | 156082 | 158117 |
| Length of large single copy region (bp) | 87638 | 86339 | 86545 | 85436 | 87564 |
| Length of inverted repeat region (bp) | 26090 | 26282 | 26087 | 25352 | 26500 |
| Length of small single copy region (bp) | 16943 | 16929 | 18314 | 19942 | 17553 |
| Coding size (bp) | 99572 | 99504 | 99738 | 101265 | 100586 |
| Intron size (bp) | 15405 | 14592 | 15451 | 14760 | 15409 |
| Spacer size (bp) | 41784 | 41736 | 41844 | 40057 | 42122 |
| Total GC content (%) | 38.0 | 38.1 | 37.9 | 37.8 | 38.2 |
| GC content of LSC (%) | 36.0 | 36.3 | 36.1 | 36.0 | 36.4 |
| GC content of IR (%) | 43.0 | 43.0 | 43.1 | 43.5 | 43.2 |
| GC content of SSC (%) | 32.8 | 32.7 | 31.3 | 31.3 | 32.4 |
| Total number of genes | 112 | 111 | 112 | 112 | 112 |
| Number of protein encoding genes | 78(comparing to *Amborella*, lack rpl32) | 77(lack rps16 and rpl32) | 78(lack rpl32) | 78(lack infA) | 78(lack rpl32) |
| Number of tRNA genes | 30 | 30 | 30 | 30 | 30 |
| Number of rRNA genes | 4 | 4 | 4 | 4 | 4 |
| Number of genes duplicated in IR | 17 | 17 | 17 | 17 | 17 |

**Supplementary Table S2** Summary of the plastome features of newly sequenced Ranunculaceae species (continued).

| Category | *Callianthemum alatavicum* | *Caltha palustris* | *Ceratocephala falcata* | *Cimicifuga dahurica* | *Clematis aethusifolia* |
| --- | --- | --- | --- | --- | --- |
| Total cp genome size (bp) | 156538 | 155325 | 150821 | 159362 | 159598 |
| Length of large single copy region (bp) | 86901 | 84123 | 83576 | 88465 | 79518 |
| Length of inverted repeat region (bp) | 25978 | 26421 | 24165 | 26572 | 31041 |
| Length of small single copy region (bp) | 17681 | 18360 | 18915 | 17753 | 17998 |
| Coding size (bp) | 99964 | 100845 | 97081 | 100862 | 104607 |
| Intron size (bp) | 14127 | 15515 | 13731 | 15180 | 15313 |
| Spacer size (bp) | 42447 | 38965 | 40009 | 43320 | 39678 |
| Total GC content (%) | 38.2 | 38.1 | 38.4 | 38.1 | 37.9 |
| GC content of LSC (%) | 36.5 | 36.4 | 36.8 | 36.3 | 36.3 |
| GC content of IR (%) | 43.2 | 43.0 | 43.7 | 43.1 | 42.0 |
| GC content of SSC (%) | 31.9 | 32.0 | 32.3 | 32.3 | 31.4 |
| Total number of genes | 112 | 113 | 113 | 112 | 112 |
| Number of protein encoding genes | 78(lack rps16) | 79 | 78(lack infA) | 78(lack rpl32) | 79 |
| Number of tRNA genes | 30 | 30 | 31(double trnfM) | 30 | 29(lack trnT-UGU) |
| Number of rRNA genes | 4 | 4 | 4 | 4 | 4 |
| Number of genes duplicated in IR | 17 | 17 | 16 | 17 | 23 |

**Supplementary Table S2** Summary of the plastome features of newly sequenced Ranunculaceae species (continued).

| Category | *Delphinium ceratophorum* | *Delphinium anthriscifolium* | *Dichocarpum dalzielii* | *Dichcarpum sutchuenense* |
| --- | --- | --- | --- | --- |
| Total cp genome size (bp) | 154245 | 155077 | 153109 | 155390 |
| Length of large single copy region (bp) | 84801 | 85871 | 82694 | 82716 |
| Length of inverted repeat region (bp) | 26560 | 25977 | 26535 | 27622 |
| Length of small single copy region (bp) | 16324 | 17252 | 17345 | 17430 |
| Coding size (bp) | 99225 | 99334 | 100441 | 101626 |
| Intron size (bp) | 14549 | 14608 | 14485 | 14584 |
| Spacer size (bp) | 40471 | 41135 | 38183 | 39180 |
| Total GC content (%) | 38.3 | 38.1 | 38.5 | 38.4 |
| GC content of LSC (%) | 36.3 | 36.2 | 36.8 | 36.6 |
| GC content of IR (%) | 43.0 | 43.2 | 43.2 | 43.0 |
| GC content of SSC (%) | 32.9 | 32.6 | 32.4 | 32.2 |
| Total number of genes | 111 | 111 | 112 | 112 |
| Number of protein encoding genes | 77(lack rpl32, rps16) | 77(lack rpl32, rps16) | 78(lack rpl32) | 78(lack rpl32) |
| Number of tRNA genes | 30 | 30 | 30 | 30 |
| Number of rRNA genes | 4 | 4 | 4 | 4 |
| Number of genes duplicated in IR | 17 | 17 | 17 | 19 |

**Supplementary Table S2** Summary of the plastome features of newly sequenced Ranunculaceae species (continued).

| Category | *Halerpestes sarmentosa* | *Helleborus thibetanus* | *Naravelia pilulifera* | *Nigella damascena* | *Oxygraphis glacialis* |
| --- | --- | --- | --- | --- | --- |
| Total cp genome size (bp) | 157299 | 154944 | 159513 | 155222 | 156503 |
| Length of large single copy region (bp) | 85663 | 85962 | 79312 | 87107 | 86298 |
| Length of inverted repeat region (bp) | 25057 | 24999 | 31054 | 25167 | 25094 |
| Length of small single copy region (bp) | 21522 | 18984 | 18093 | 17781 | 20017 |
| Coding size (bp) | 100315 | 100363 | 104592 | 99821 | 100283 |
| Intron size (bp) | 15609 | 15133 | 17025 | 15502 | 15497 |
| Spacer size (bp) | 41375 | 39448 | 37896 | 39899 | 40723 |
| Total GC content (%) | 37.9 | 37.8 | 37.9 | 38.8 | 37.8 |
| GC content of LSC (%) | 36.1 | 35.9 | 36.3 | 37.2 | 35.9 |
| GC content of IR (%) | 43.4 | 43.4 | 42.0 | 43.5 | 43.3 |
| GC content of SSC (%) | 32.2 | 31.6 | 31.2 | 33.5 | 31.8 |
| Total number of genes | 112 | 113 | 112 | 112 | 112 |
| Number of protein encoding genes | 78(lack infA) | 79 | 79 | 78(lack rpl32) | 78(lack infA) |
| Number of tRNA genes | 30 | 30 | 29(lack trnT-UGU) | 30 | 30 |
| Number of rRNA genes | 4 | 4 | 4 | 4 | 4 |
| Number of genes duplicated in IR | 17 | 16 | 23 | 17 | 17 |

**Supplementary Table S2** Summary of the plastome features of newly sequenced Ranunculaceae species (continued).

| Category | *Ranunculus sceleratus* | *Souliea vaginata* | *Thalictrum minus* | *Thalictrum petaloideum* | *Thalictrum tenue* | *Trollius ranunculoides* |
| --- | --- | --- | --- | --- | --- | --- |
| Total cp genome size (bp) | 156324 | 158357 | 156201 | 155876 | 156103 | 159666 |
| Length of large single copy region (bp) | 85835 | 87686 | 85685 | 85326 | 85507 | 88194 |
| Length of inverted repeat region (bp) | 25302 | 26533 | 26482 | 26480 | 26504 | 26500 |
| Length of small single copy region (bp) | 19885 | 17605 | 17579 | 17590 | 17588 | 18472 |
| Coding size (bp) | 101072 | 100187 | 99844 | 99854 | 99890 | 98114 |
| Intron size (bp) | 15648 | 15151 | 15328 | 15268 | 15341 | 15430 |
| Spacer size (bp) | 39604 | 43019 | 41029 | 40754 | 40872 | 46122 |
| Total GC content (%) | 37.9 | 38.0 | 38.4 | 38.4 | 38.4 | 38.0 |
| GC content of LSC (%) | 36.1 | 36.2 | 36.6 | 36.6 | 36.6 | 36.3 |
| GC content of IR (%) | 43.5 | 43.0 | 43.2 | 43.2 | 43.2 | 43.1 |
| GC content of SSC (%) | 31.7 | 32.3 | 32.5 | 32.5 | 32.4 | 31.8 |
| Total number of genes | 112 | 112 | 112 | 112 | 112 | 113 |
| Number of protein encoding genes | 78(lack infA) | 78(lack rpl32) | 78(lack rpl32) | 78(lack rpl32) | 78(lack rpl32) | 79 |
| Number of tRNA genes | 30 | 30 | 30 | 30 | 30 | 30 |
| Number of rRNA genes | 4 | 4 | 4 | 4 | 4 | 4 |
| Number of genes duplicated in IR | 17 | 17 | 17 | 17 | 17 | 17 |
