# Supplementary material for: Structural variation of the complete chloroplast genome and plastid phylogenomics of the genus Asteropyrum (Ranunculaceae)
Source: Sci Rep. 2019 Oct 25;9:15285. doi: 10.1038/s41598-019-51601-2 (PMC6814708; doi:10.1038/s41598-019-51601-2)
Supplement: Supplementary file 2 — Supplementary dataset [file 41598_2019_51601_MOESM2_ESM.zip › Supplementary dataset/Supplementary Table S4.docx]

**Supplementary Table S4** *Asteropyrum* materials used for IR-SC boundary checking.

| Species | Sample locality | No. of individuals | Voucher (Herbarium) |
| --- | --- | --- | --- |
| *Asteropyrum peltatum* ssp. *peltatum* | Medog, Xizang, China | 1 | *X.X. Zhu* s.n. (KUN) |
| *A. peltatum* ssp. *peltatum* | Baoxing, Sichuan, China | 2 | *L. Xie* 2015-BX024 (BJFC) |
| *A. peltatum* ssp. *cavaleriei* | YiBin, Sichuan, China | 2 | *L. Xie* 2014-YB016 (BJFC) |
| *A. peltatum* ssp. *cavaleriei* | Dujiangyan, Sichuan, China | 1 | *L. Xie* 2012-DJ002 (BJFC) |
| *A. peltatum* ssp. *cavaleriei* | Nanchuan, Chongqing, China | 1 | *L. Xie* 2006-CQ041 (BJFC) |
| *Intermediate form* | YiBin, Sichuan, China | 1 | *L. Xie* 2014-YB019 (BJFC) |
