# Supplementary material for: Structural variation of the complete chloroplast genome and plastid phylogenomics of the genus Asteropyrum (Ranunculaceae)
Source: Sci Rep. 2019 Oct 25;9:15285. doi: 10.1038/s41598-019-51601-2 (PMC6814708; doi:10.1038/s41598-019-51601-2)
Supplement: Supplementary file 2 — Supplementary dataset [file 41598_2019_51601_MOESM2_ESM.zip › Supplementary dataset/Supplementary Table S5.docx]

**Supplementary Table S5** Primers designed for IR-SC boundary checking using Sanger sequencing.

| **Position** | **Region length** | **Primer name** | **Primers** |
| --- | --- | --- | --- |
| ssp. *cavaleriei*: rps11-trnH | 1008 bp | RIR2F | CAGCCGCAAATGTGATTCGT |
|  |  | RIR2R | ATTGTGAATCCACCATGCGC |
| ssp. *peltatum*: rpoA-trnH | 487 bp | 92RIR2F | CCAATCAGAATTGCCTTCCAGG |
|  |  | 92RIR2R | ATTGTGAATCCACCATGCGC |
