# Supplementary figures and images for: Structural variation of the complete chloroplast genome and plastid phylogenomics of the genus Asteropyrum (Ranunculaceae)
Source: Sci Rep. 2019 Oct 25;9:15285. doi: 10.1038/s41598-019-51601-2 (PMC6814708; doi:10.1038/s41598-019-51601-2)

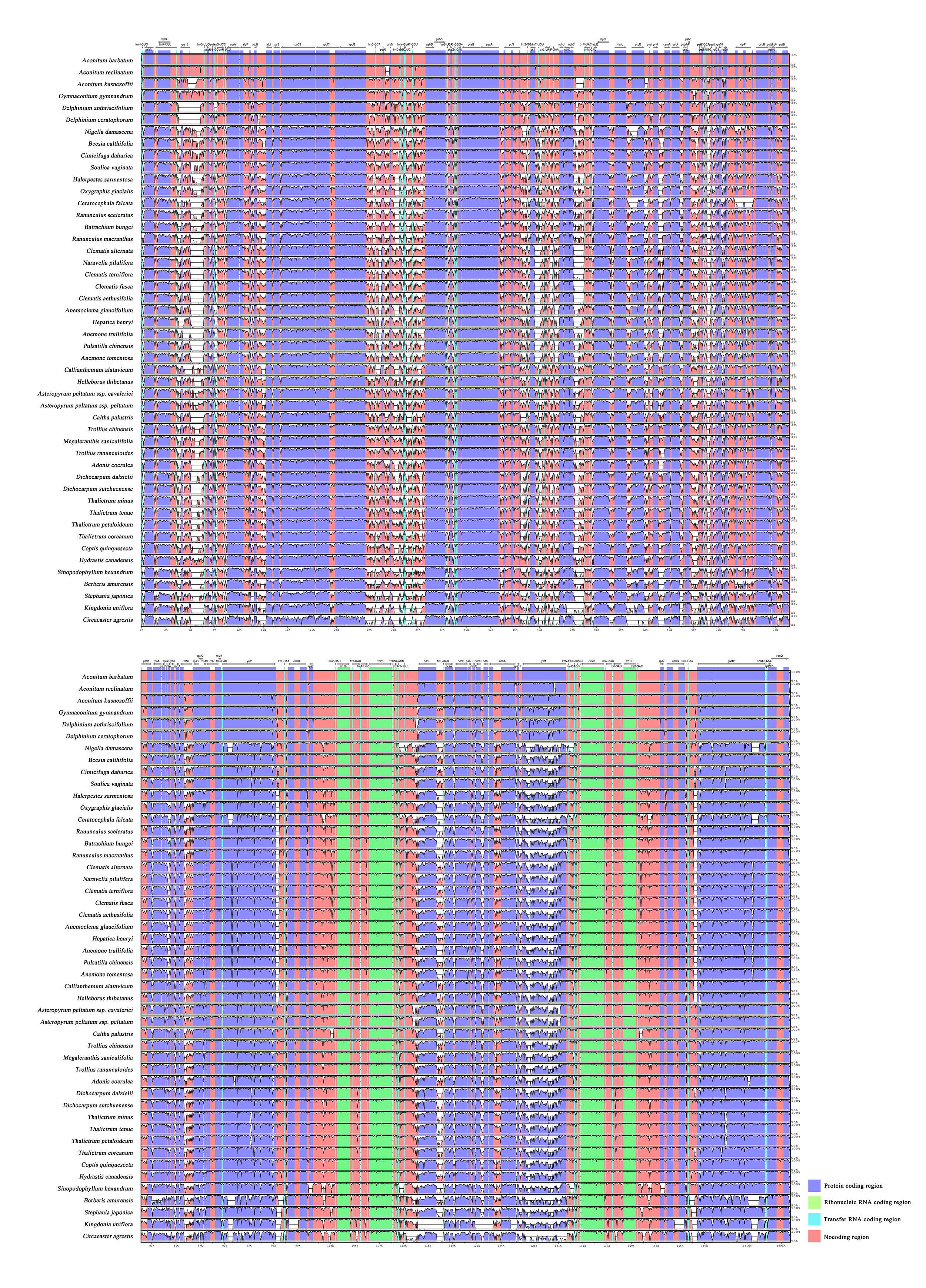

Supplement: Supplementary file 2 — Supplementary dataset [file 41598_2019_51601_MOESM2_ESM.zip › Supplementary dataset/Supplementary Figure S2.jpg]

Supplementary Figure S3

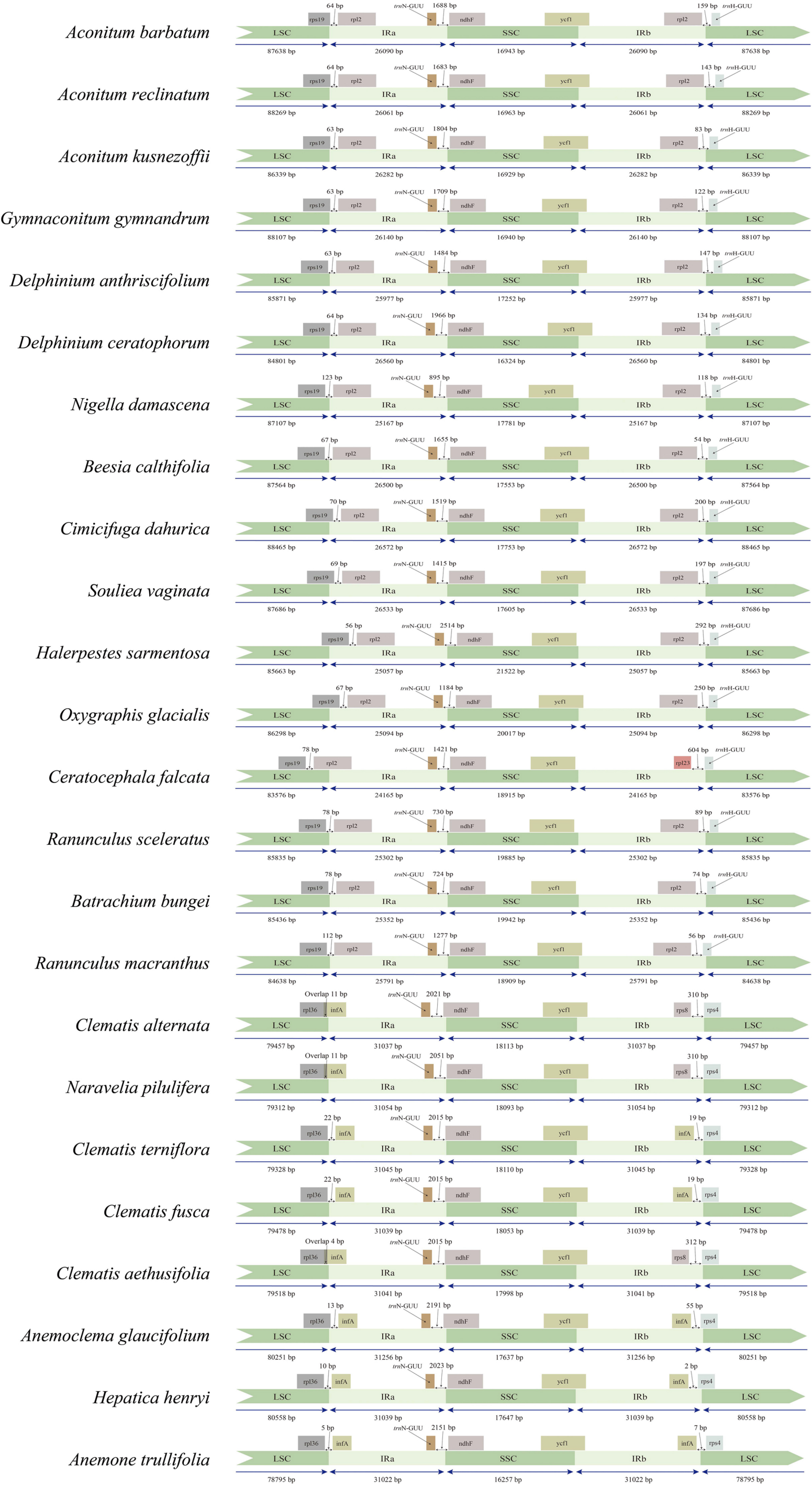

## Supplementary Figure S3 (continue)

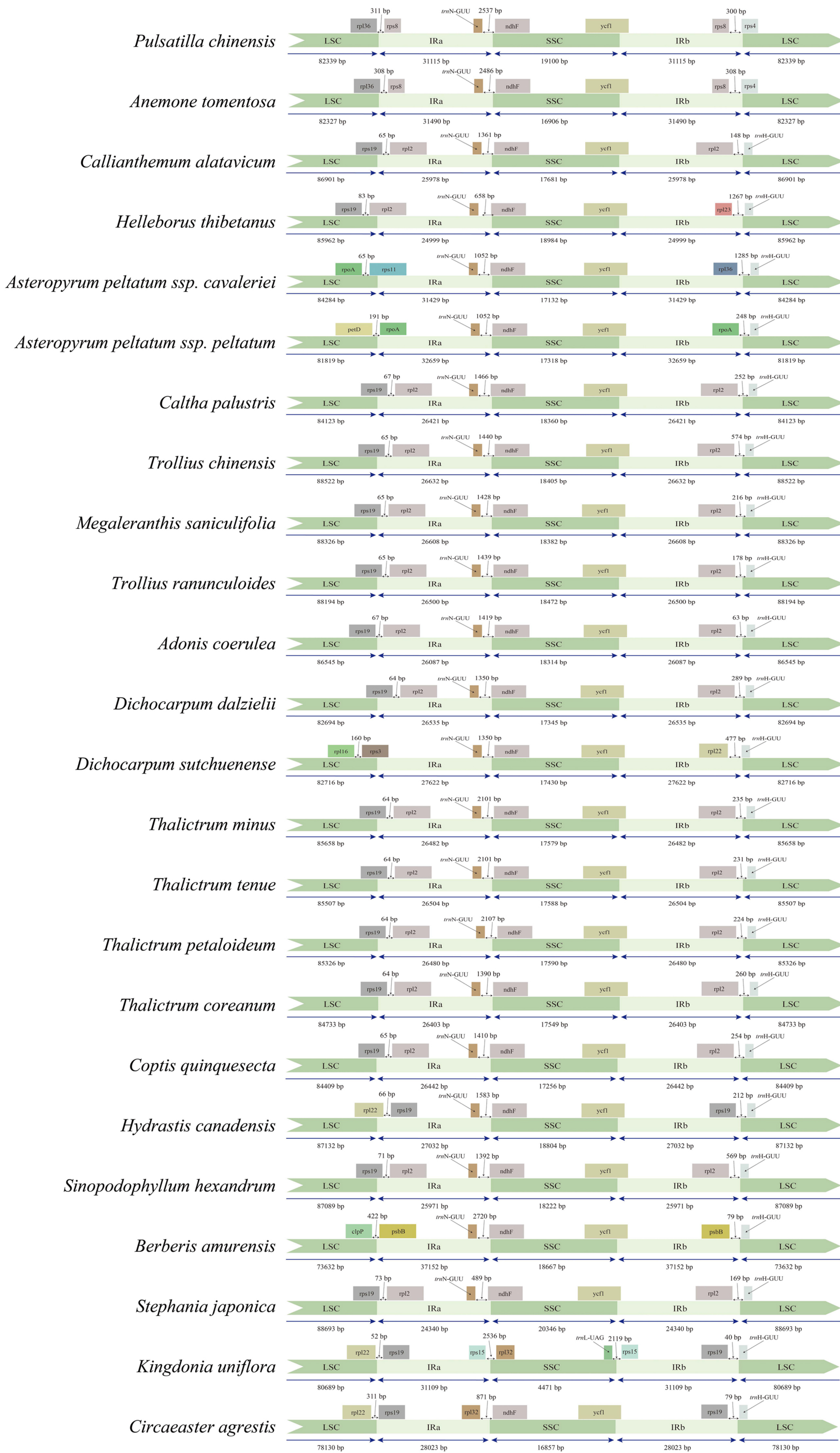

Supplement: Supplementary file 2 — Supplementary dataset [file 41598_2019_51601_MOESM2_ESM.zip › Supplementary dataset/Supplementary Figure S3.pdf]
